# Naïve pluripotent-like characteristics of non-tumorigenic Muse cells isolated from human amniotic membrane

**DOI:** 10.1038/s41598-022-22282-1

**Published:** 2022-10-14

**Authors:** Eiji Ogawa, Yo Oguma, Yoshihiro Kushida, Shohei Wakao, Kana Okawa, Mari Dezawa

**Affiliations:** Department of Stem Cell Biology and Histology, Tohoku University Graduate School of Medicine, 2-1 Seiryo-Machi, Sendai, 980-8575 Japan

**Keywords:** Mesenchymal stem cells, Multipotent stem cells

## Abstract

Multilineage-differentiating stress-enduring (Muse) cells are non-tumorigenic pluripotent-like stem cells that exhibit triploblastic differentiation and self-renewability at the single-cell level, and are collectable as pluripotent surface marker SSEA-3(+) from the bone marrow (BM), peripheral blood, and organ connective tissues. SSEA-3(+) cells from human amniotic membrane mesenchymal stem cells (hAMSCs) were compared with hBM-Muse cells. Similar to hBM-Muse cells, hAMSC-SSEA-3(+) cells expressed pluripotency genes (*OCT3/4*, *NANOG,* and *SOX2*), differentiated into triploblastic cells from a single cell, self-renewed, and exhibited non-tumorigenicity. Notably, however, they exhibited unique characteristics not seen in hBM-Muse cells, including higher expression of genes related to germline- and extraembryonic cell-lineages compared with those in hBM-Muse cells in single-cell RNA-sequencing; and enhanced expression of markers relevant to germline- (*PRDM14, TFAP2C, and NANOS3*) and extraembryonic cell- (*CDX2*, *GCM1*, and *ID2*) lineages when induced by cytokine subsets, suggesting a broader differentiation potential similar to naïve pluripotent stem cells. t-SNE dimensionality reduction and Gene ontology analysis visualized hAMSC-SSEA-3(+) cells comprised a large undifferentiated subpopulation between epithelial- and mesenchymal-cell states and a small mesenchymal subpopulation expressing genes relevant to the placental formation. The AM is easily accessible by noninvasive approaches. These unique cells are a potentially interesting target naïve pluripotent stem cell-like resource without tumorigenicity.

## Introduction

Recent advances in stem cell biology suggest that cells from the human amniotic membrane (hAM) include a unique stem cell population similar to epiblasts, which exhibit properties relevant to the early stage of development^1^. Unlike other types of embryonic tissues, the AM is formed from amnioblasts derived from pluripotent epiblasts prior to gastrulation, a phase of cell fate determination, and therefore maintain a broad differentiation potential^1^. The AM is suggested to contain residual epiblast-like pluripotent cells based on the recently proposed “Stem Cell Left Behind Theory”^1^. In addition, some aspects of hAM cells are similar to embryonic stem (ES) cells because amnioblasts differentiate in the blastocyst stage. Indeed, in addition to osteogenic, chondrogenic, and adipogenic differentiation, human amniotic membrane mesenchymal stem cells (hAMSCs)^2^ are suggested to differentiate into cells of all 3 germ layers by treatment with cytokine subsets^3^.

Besides their broad differentiation potential, hAM cells have several practical advantages for clinical application. First, the hAM is usually discarded as medical waste following delivery and is easily procured without additional invasive procedures. As such, hAM cells are inexpensive to obtain and, unlike embryo- or fetus-derived stem cells, their collection does not pose ethical problems. Second, hAM cells have less age- or environmental-associated DNA damage compared with adult stem cells^1^. Third, hAM cells are immunotolerant due to their weak expression of MHC class I and the lack of MHC class II expression^4^.One of the roles of the AM is to protect the fetus from maternal immune recognition, and thus hAM cells are suitable for allogeneic cell transplantation. Fourth, the AM has immunosuppressive and anti-inflammatory effects, making hAM cells applicable for inflammatory diseases^5^. On the basis of these properties, intracameral hAMSC injection is suggested to induce an immunosuppressive, anti-inflammatory, and anti-fibrotic environment that promotes corneal wound healing^6^, and increasing evidence indicates that hAMSCs may provide an alternative therapeutic approach for skin injury^7^.

Muse cells, isolated from the bone marrow (BM), blood, and organ connective tissues, express pluripotency genes, including *OCT3/4* (also known as *POU5F1*), *NANOG,* and SRY-box transcription factor2 (*SOX2*), and can be isolated as cells positive for stage-specific embryonic antigen-3 (SSEA-3), a representative pluripotent stem cell surface marker^8^. Muse cells display pluripotent-like properties because they can differentiate into cells of ectodermal-, endodermal-, and mesodermal-lineages both in vitro^8^ and in vivo^9–11^, and have the ability to self-renew at a single-cell level^8^. They also exhibit stress tolerance^12,13^. Consistent with the fact that they reside in adult normal tissues, Muse cells have low telomerase activity and do not form teratomas when injected into the testis^8,14^; thus, Muse cells have low safety concerns. Allogenic Muse cells do not induce immune rejection due to a specific immune privilege system similar to the placenta, represented by human leukocyte antigen (HLA)-G expression^8,15^. In addition, Muse cells selectively home to sites of damage via the sphingosine-1-phosphate (S1P)-S1P receptor 2 axis in vivo^10^ where they spontaneously differentiate into tissue-specific cells according to the microenvironment to replace damaged/apoptotic cells and contribute to tissue regeneration when infused into the bloodstream^8–11^. Therefore, Muse cells do not require gene introduction for rendering pluripotency, nor do they require induction for differentiating into target cell types prior to clinical use. Based on these unique properties, clinical trials for their application in acute myocardial infarction^16^, stroke, epidermolysis bullosa^17^, spinal cord injury, neonatal cerebral palsy, and amyotrophic lateral sclerosis (ALS) are in progress, all based on intravenous administration of donor-derived Muse cells without HLA-matching tests or long-term immunosuppressant treatment^18^.

Pluripotency is largely classified as two phases^19^ : naive (representing newly segregated pre-implantation epiblast and rodent naïve ES cell state) and primed (representing the post-implantation epiblast cell state)^20^. The two states are distinguished by several characters including reactivity to cytokines and X chromosomal activities, but more importantly, only naïve pluripotent stem cells are capable of differentiating not only into triploblastic-lineages but also into germ-line cells and/or extraembryonic-lineage cells^21,22^. However, typical examples of naïve pluripotent stem cells, as represented by mouse ES cells, have tumorigenic proliferative activity^20^.

In this study, we focused on the AM, which is an attractive cell source for regenerative therapy, expecting to identify cells comparable to Muse cells. We also compared the characteristics of those cells with hBM-Muse cells, the human Muse cells used most frequently for studies^8^, and found that hAM-derived Muse cells are naïve pluripotent-like without tumorigenic proliferative activity.

## Results

### Characterization of SSEA-3(+) cells from human AMSCs

**Figure 1:**
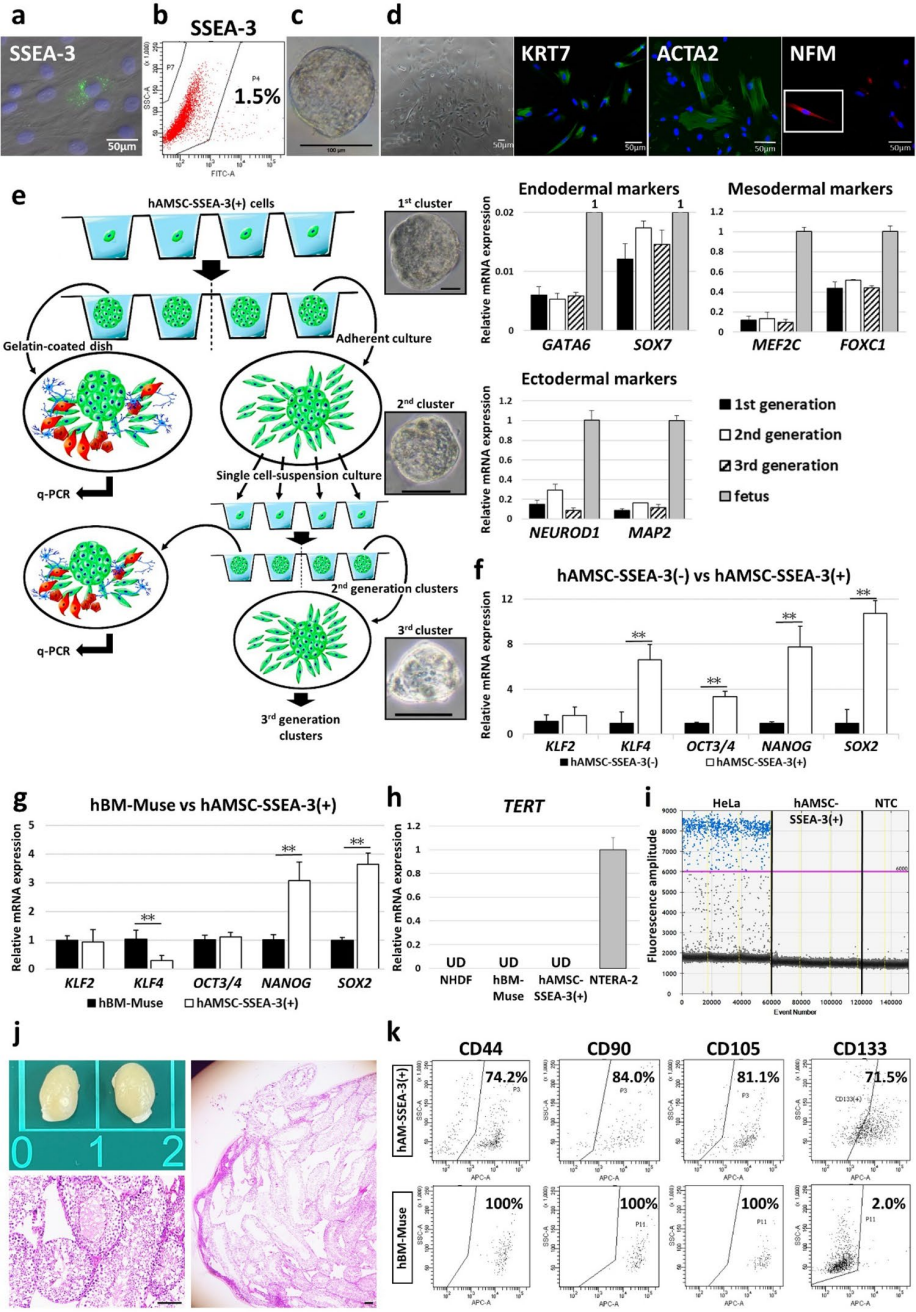
Characterization of SSEA-3(+) cells from human AMSCs. (**a**) Immunocytochemistry for SSEA-3 in hAMSCs (bar = 50 μm). (**b**) Example of SSEA-3(+) cells in hAMSCs in cell sorting. (**c**) Clusters were formed in single-cell suspension culture from hAMSC-SSEA-3(+) cells (bar = 100 µm). (**d**) Phase contrast image of cells expanded from the cluster in a gelatin-coated adherent culture dish and immunocytochemistry for KRT7, ACTA2, and NFM in the expanded cells (bars = 50 μm). (**e**) hAMSC-SSEA-3(+) cells demonstrated a capacity for self-renewal. Schematic diagram outlines experiments that validated self-renewal ability of hAMSC-SSEA-3(+) cells. *GATA6* and *SOX7* (endodermal), *MEF2C* and *FOXC1* (mesodermal), and *NEUROD1* and *MAP2* (ectodermal) gene expression was detected in qPCR from cells expanded from each of the clusters from the first to third generations (bars = 50 µm). (**f**) Expression of pluripotency-related genes in hAMSC-SSEA-3(−) and hAMSC-SSEA-3(+) cells (normalized by beta-actin (*ACTB*)). Values of hAMSC-SSEA-3(−) cells were set as 1. *p < 0.05; **p < 0.01. (**g**) Expression of pluripotency-related genes in hBM-Muse and hAMSC-SSEA-3(+) cells (normalized by *ACTB*). Values of hBM-Muse cells were set as 1. *p < 0.05; **p < 0.01. (**h**) Expression of *TERT* in normal human dermal fibroblasts (NHDF), hBM-Muse cells, hAMSC-SSEA-3(+) cells, and NTERA-2 measured by qPCR (normalized by *ACTB*). Values of NTERA-2 were set as 1. UD = under limits of detection. (**i**) Number of telomerase products in HeLa cells, hAMSC-SSEA-3(+) cells and non-template control (NTC) measured by ddPCR. Each dot on the ddPCR output represents a unique droplet that is either positive or negative for a fluorescent signal whose threshold was 6000. (**j**) Transplantation of hAMSC-SSEA-3(+) cells into SCID mouse testis did not form teratoma after 4 months (bar = 100 µm). (**k**) Expression of mesenchymal markers (CD44, CD90, and CD105) and CD133 in hAMSC-SSEA-3(+) and hBM-Muse cells.

Cells positive for SSEA-3 were identified using immunohistochemistry in hAMSCs in adherent culture (Fig. 1a). Fluorescence-activated cell sorting (FACS) analysis revealed the presence of SSEA-3(+) cells in hAMSCs at concentrations of ~ 1.5% (Fig. 1b).

When hAMSC-SSEA-3(+) cells were transferred to single-cell suspension culture, each cell proliferated to form a cluster whose morphology was similar to that of ES-cell–derived embryoid bodies formed in suspension culture at days 7–10, similar to previous reports^10,15^ (Fig. 1c). Importantly, none of the hAMSCs-SSEA-3(−) cells formed clusters in single-cell suspensions. When these single-cell–derived clusters were individually transferred onto gelatin-coated dishes and cultured without cytokine induction for 10–14 days, the cells expanded from the cluster and proliferated (Fig. 1d). Among the expanded cells, we identified cells positive for keratin7 (KRT7; endodermal marker), smooth muscle actin alpha 2 (ACTA2; mesodermal marker), and neurofilament medium chain (NFM; ectodermal marker) (Fig. 1d). Thus, SSEA-3(+) cells are suggested to have the ability to spontaneously generate cells representative of all 3 germ layers from a single cell.

To examine self-renewability, hAMSC-SSEA-3(+) cells were subjected to single-cell suspension culture to generate first-generation clusters. Half of the clusters were transferred onto gelatin-coated adherent culture media, maintained, and analyzed by quantitative-polymerase chain reaction (qPCR) for the expression of endodermal- (*GATA6* and *SOX7*), mesodermal- (myocyte enhancer factor 2C [*MEF2C*] and forkhead box C1 [*FOXC1*]), and ectodermal-lineage markers (neuronal differentiation 1 [*NEUROD1*] and microtubule associated protein 2 [*MAP2*]) (Fig. 1e). The remaining clusters were each transferred onto a non-coated adherent culture dish and allowed to proliferate for 7–10 days, after which they underwent a second-round of single-cell suspension culture to generate second-generation clusters. This experimental cycle was repeated until the third generation. Consequently, the expression of markers for each germ layer was identified in the first- to third-generation clusters by qPCR (Fig. 1e).

Somatic stem cells are known to randomly repeat symmetric and asymmetric cell divisions during proliferation. In single cell suspension culture, one of the two daughter cells of hAMSC-SSEA-3(+) cell expressed NUMB, the molecule known to influence on the cell fate of somatic stem cells by being asymmetrically segregated during cell division (Supplementary Fig. 1a)^23^.

Expression of genes related to pluripotency was analyzed by qPCR, comparing SSEA-3(+) with SSEA-3(−) cells from hAMSCs. hAMSC-SSEA-3(+) cells exhibited significantly higher (*p* < 0.01) expression of Kruppel-like factor 4 (*KLF4*), *OCT3/4*, *NANOG*, and *SOX2* compared with hAMSC-SSEA-3(−) cells, while *KLF2* was similar between the hAMSC SSEA-3(+) and SSEA-3(−) cells (Fig. 1f).

Comparison of hAMSC-SSEA-3(+) cells with hBM-Muse cells collected as SSEA-3(+) from hBM-MSCs as reported previously^8^ revealed significantly higher expression of *NANOG* and *SOX2* in hAMSC-SSEA-3(+) cells than in hBM-Muse cells (both *p* < 0.01), and lower expression of *KLF4* (*p* < 0.01), but similar levels of *KLF2* and *OCT3/4* between them (Fig. 1g).

Telomerase is an enzyme that indicates tumorigenic proliferative activity and comprises 3 core components. Telomerase reverse transcriptase (*TERT*), which is the catalytic subunit in telomerase, is the most important core component for tumorigenic proliferative activity^24,25^. In qPCR, *TERT* was under the limits of detection in normal human dermal fibroblasts (NHDF), hBM-Muse, and hAMSC-SSEA-3(+) cells, while its signal was detectable in pluripotent human embryonal carcinoma (NTERA-2) (Fig. 1h). Therefore, the *TERT* expression level in hAMSCs is comparable to that in NHDF and hBM-Muse cells (Fig. 1h). Telomerase enzyme activity and telomere maintenance are almost universal features of tumorigenic cells^26^. The droplet-digital telomere repeat amplification protocol (ddTRAP) is a method that adapts the telomere-repeat amplification procedure (TRAP), one of the most common assays for measuring telomerase activity, to droplet-digital PCR (ddPCR) in order to improve the sensitivity, reproducibility, and throughput of telomerase activity^26^. ddTRAP output showed that HeLa cells (input of 100 cell equivalents) were telomerase-positive, while hAMSC-SSEA-3(+) cells (input of 100 cell equivalents) and the non-template control (NTC) were telomerase-negative (Fig. 1i).

Previous reports suggested that mouse testis transplanted into mouse ES cells formed teratoma containing three germ layer tissues after 8–10 weeks^14^. Human iPS cells also formed teratoma by 12 weeks^27^. We investigated whether hAMSC-SSEA-3(+) cell transplantation generates teratoma in SCID mice testis. Transplantation of 1 × 10^5^ hAMSC-SSEA-3(+) cells into the mouse testis did not form teratoma at 4 months after transplantation (Fig. 1j).

Surface marker expression was compared among hBM-Muse cells, hAMSC-SSEA-3(+) cells, and hAMSCs. The positivity ratio for each marker differed among them: all 3 markers were expressed in 100% of the hBM-Muse cells; in hAMSCs, CD44 was expressed in 98.3%, CD90 was expressed in 83.4%, and CD105 was expressed in 99.4% (Fig. 1k, Supplementary Fig. 1b). In hAMSC-SSEA-3(+) cells, CD44 was expressed in 74.2%, CD90 was expressed in 84.0%, and CD105 was expressed in 81.1%. Notably, CD133, which is expressed in ES cells and cancer stem cells (93.1% of NTERA-2 was positive as shown in Supplementary Fig. 1c)^28^, was 2.0% positive in hBM-Muse cells and 0.8% positive in hAMSCs, while it was as high as 71.5% in hAMSC-SSEA-3(+) cells (Fig. 1k, Supplementary Fig. 1c). Hematopoietic markers CD34 and CD45 were under the detection limit in all 3 types of cells (data not shown).

We investigated the possibility of contamination of other amniotic cell types such as epithelial cells into hAMSCs population. Amniotic epithelial cells are known to express epithelial surface marker CD326^29^. NTERA-2 as positive control exhibited ~ 88.3% of positivity for CD326 (Supplementary Fig. 1^1^). hAMSCs were, however, negative for CD326. Since hAMSCs were negative, hAMSC-SSEA-3(+) cells were also negative for CD326 (Supplementary Fig. 1d).

In summary, hAMSC-SSEA-3(+) cells exhibited the ability to differentiate into triploblastic lineage cells and to self-renew at the single-cell level (similar to embryonic stem cells), proliferated asymmetrically (similar to adult stem cells), expressed pluripotency-related gene and MSC surface markers, and did not form teratomas after transplantation in vivo for up to 4 months, similar to Muse cells isolated from the BM, dermis, peripheral blood, and adipose tissue^8,12,27,30–32^. Therefore, in the following text, we refer to hAMSC-SSEA-3(+) cells as ‘hAM-Muse cells’.

### SSEA-3(+) cells from mouse AMSCs

We isolated adherent cells from the AM of ICR mice at 12–15 days gestation (Supplementary Fig. 2a). These cells expressed mesenchymal markers CD44 (96.0%) and CD29 (95.3%) (Supplementary Fig. 2b). On the other hand, hematopoietic markers CD34 and CD45 were under the detection limit (data not shown). Therefore, we refer to mouse AM-adherent mesenchymal marker (+) cells as ‘mAMSCs’ in the following text^2^. FACS analysis revealed the presence of SSEA-3(+) cells in mAMSCs at ~ 0.4% (Supplementary Fig. 2c). Cells positive for SSEA-3 were also recognized in mAMSCs in adherent culture with immunocytochemistry and these cells proliferated to form single clusters in single-cell suspension culture at days 5–7 (Supplementary Fig. 2c).

SSEA-1 is a cell surface marker used to monitor the early stages of embryogenesis in mice and humans because it is expressed only in preimplantation mouse embryo beginning at the 8-cell stage and ES cells, but not in their differentiated derivatives^33^. Although only 0.2% of mAMSCs were SSEA-1(+) (Supplementary Fig. 2b), nearly half (47.8%) of mAMSC-SSEA-3(+) cells were SSEA-1(+) (Supplementary Fig. 2c).

Expression of genes related to pluripotency was analyzed by qPCR, and compared between SSEA-3(+) with SSEA-3(−) cells from mAMSCs. SSEA-3(+) cells exhibited significantly higher expression of *Oct3/4* (*p* < 0.05), *Nanog* (*p* < 0.01), *Sox2* (*p* < 0.01), *Klf4* (*p* < 0.05), *Rex1* (*p* < 0.05), and *Ssea-1* (*p* < 0.01) compared with mAMSC-SSEA-3(−) cells (Supplementary Fig. 2d). Analysis of mAMSC-SSEA-3(+), mAMSC-SSEA-3(−), and mouse adipose (mAD)-Muse cells for the expression of *Prdm14*, *Blimp1* (also known as *Prdm1*), and transcription factor AP-2 gamma (*Tfap2c*), all of which are germ cell-related markers^34^, revealed significantly higher levels of these markers in mAMSC-SSEA-3(+) cells than in the other 2 types of cells (Supplementary Fig. 2e). Immunohistochemistry of the mAM showed that SSEA-3(+) cells mainly located in the epithelial layer, but not in the mesenchymal area (Supplementary Fig. 2f.).

### Comparison between hAM- and hBM-Muse cells by scRNA-seq

**Figure 2:**
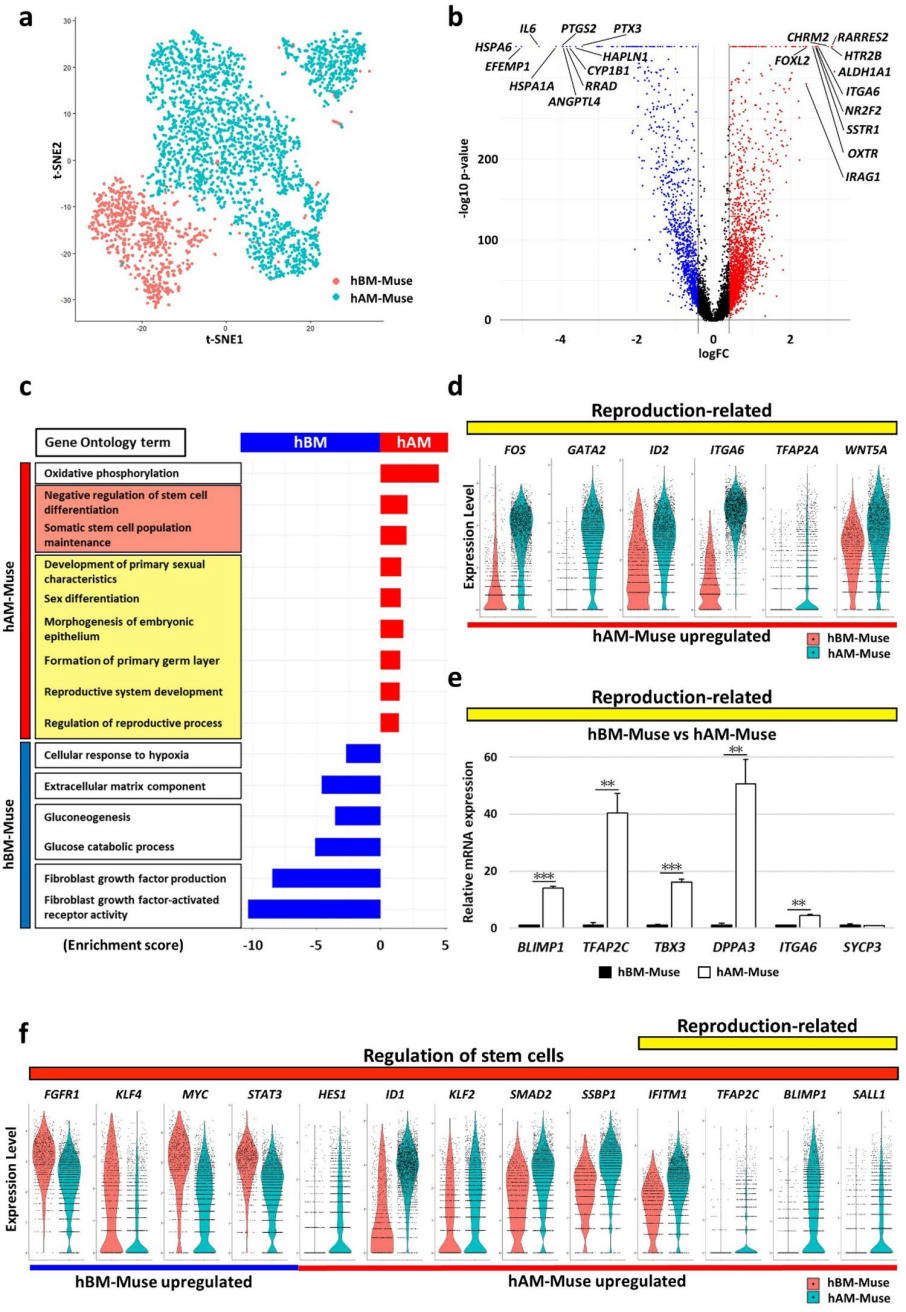
Comparison of hAM- and hBM-Muse cells in scRNA-seq. (**a**) t-SNE plot of hAM- and hBM-Muse cells. (**b**) Volcano plot displaying gene expression levels in hAM-Muse cells compared with those in hBM-Muse cells. Significantly differentially expressed genes were defined as those with a fold-change > 1.5 times or < 0.67 times in hAM-Muse cells compared with hBM-Muse cells, with *p* < 0.05. Upregulated genes are shown in red, downregulated genes are shown in blue, and the black lines represent the boundary for identification of upregulated or downregulated genes based on the *p*-value and fold-change. Top 10 differentially expressed genes in hAM- and hBM-Muse cells are shown. (**c**) GO analysis; upregulated genes in hAM- (red) and hBM-Muse cells (blue) are listed. Enrichment score of each term is shown. (**d**) Violin plot showing the expression levels of reproduction-related genes in hBM- (red) and hAM-Muse cells (blue). (**e**) Expression levels of reproduction-related genes in hBM- and hAM-Muse cells were confirmed by qPCR (normalized by *ACTB*). Values of hBM-Muse cells were set as 1. *p < 0.05; **p < 0.01; ***p < 0.001. (**f**) Violin plot showing the expression levels of genes related to regulation of stem cells in hBM- (red) and hAM-Muse cells (blue).

hAM- and hBM-Muse cells were analyzed by scRNA-seq. The hBM-Muse cells were prepared from 1 batch (834 cells) and the hAM-Muse cells from 2 batches, 1 from males (1312 cells) and the other from females (1420 cells). We used t-distributed stochastic neighbor embedding (t-SNE) dimensionality reduction to visualize the relationship between the 2 cell types (Fig. 2a). hAM- (blue cluster) and hBM-Muse (red cluster) cells were assigned to different clusters (Fig. 2a). Each cluster contained all periods of the cell cycle; G1, G2 and M, and S at proportions of 48%, 37%, and 15% (hAM-Muse cells) and 81%, 7%, and 12% (hBM-Muse cells) (Supplementary Fig. 3).

Volcano plot revealed that 3421 genes were significantly altered (fold-change [FC] > 1.5, *p* < 0.05) between hAM- and hBM-Muse cells; 2183 genes in hAM-Muse cells were upregulated compared with hBM-Muse cells (red dots) and 1238 genes in hAM-Muse cells were downregulated compared with the hBM-Muse cells (blue dots) (Fig. 2b, Supplementary Table 1). We identified the top 10 gene examples that were significantly different; the upregulated genes in hAM-Muse cells were aldehyde dehydrogenase 1 family member A1 (*ALDH1A1*), retinoic acid receptor responder 2 (*RARRES2*), 5-hydroxytryptamine receptor 2B (*HTR2B*), cholinergic receptor muscarinic 2 (*CHRM2*), integrin subunit alpha 6 (*ITGA6*), nuclear receptor subfamily 2 group F member 2 (*NR2F2*), somatostatin receptor 1 (*SSTR1*), oxytocin receptor (*OXTR*), *FOXL2*, and inositol 1,4,5-triphosphate receptor associated 1 (*IRAG1*); and the downregulated genes in hAM-Muse cells were heat shock protein family A member 6 (*HSPA6*), epidermal growth factor (EGF) containing fibulin extracellular matrix protein 1 (*EFEMP1*), interleukin 6 (*IL6*), *HSPA1A*, prostaglandin-endoperoxide synthase 2 (*PTGS2*), angiopoietin like 4 (*ANGPTL4*), *RRAD*, cytochrome P450 family 1 subfamily B member 1 (*CYP1B1*), hyaluronan and proteoglycan link protein 1 (*HAPLN1*), and pentraxin 3 (*PTX3*) (Fig. 2b).

Gene ontology (GO) analysis showed that hAM-Muse cells were enriched for genes involved in specific functions, such as oxidative phosphorylation, stem cell regulation (red highlighted in Fig. 2c), and reproduction (yellow highlighted in Fig. 2c). In contrast, upregulated genes in hBM-Muse cells, equal to downregulated genes in hAM-Muse cells, contained cellular response to hypoxia, extracellular matrix component, gluconeogenesis, and fibroblast growth factor-production and -receptor activity (Fig. 2c).

Violin plots of the expression levels of selected reproduction-related markers between hAM- and hBM-Muse cells are shown in Fig. 2d. Expression of genes related to placentation, such as *FOS*, *GATA2*, inhibitor of DNA binding 2 (*ID2*), and *ITGA6*, as well as genes involved in the formation of the embryonic epithelium and primary germ layer such as *TFAP2A* and *WNT5A* were upregulated in hAM-Muse cells compared with hBM-Muse cells (Fig. 2d). We also examined the expression of representative germ cell-related marker genes by qPCR. Expression of *BLIMP1* (*p* < 0.001), *TFAP2C* (*p* < 0.01), T-box transcription factor 3 (*TBX3*) (*p* < 0.001), developmental pluripotency associated 3 (*DPPA3*) (*p* < 0.01), and *ITGA6* (*p* < 0.01) was significantly higher in hAM-Muse cells than in hBM-Muse cells (Fig. 2e). Both hAM- and hBM-Muse cells expressed synaptonemal complex protein 3 (*SYCP3*) at similar levels (Fig. 2e).

Violin plots of genes related to stemness and/or undifferentiated state, such as fibroblast growth factor receptor 1 (*FGFR1*), *KLF4*, *MYC*, and signal transducer and activator of transcription 3 (*STAT3*), revealed higher expression of these genes in hBM-Muse cells, and higher expression of *HES1*, *ID1*, *KLF2*, *SMAD2*, and single-stranded DNA binding protein 1 (*SSBP1*) in hAM-Muse cells (Fig. 2f). Notably, expression of interferon induced transmembrane protein 1 (*IFITM1*), *TFAP2C*, *BLIMP1*, and spalt like transcription factor 1 (*SALL1*), relevant to germ cells, was substantially higher in hAM-Muse cells and very low in hBM-Muse cells (Fig. 2f). The higher expression of *ITGA6*, *BLIMP1*, and *TFAP2C* in hAM-Muse cells than in hBM-Muse cells was confirmed in both scRNA-seq and qPCR analyses (Fig. 2d–f).

Signaling pathways analyzed with the Kyoto Encyclopedia of Genes and Genomes (KEGG) revealed that the oxidative phosphorylation pathway was significantly affected in hAM-Muse cells (Supplementary Figs. 4, 5a), while the glycolysis/gluconeogenesis (Supplementary Fig. 4), hypoxia-inducible factor 1 signaling (Supplementary Fig. 5b), and extracellular matrix receptor interaction (Supplementary Fig. 6) pathways were significantly affected in hBM-Muse cells.

### Subpopulations in hAM-Muse cells

**Figure 3:**
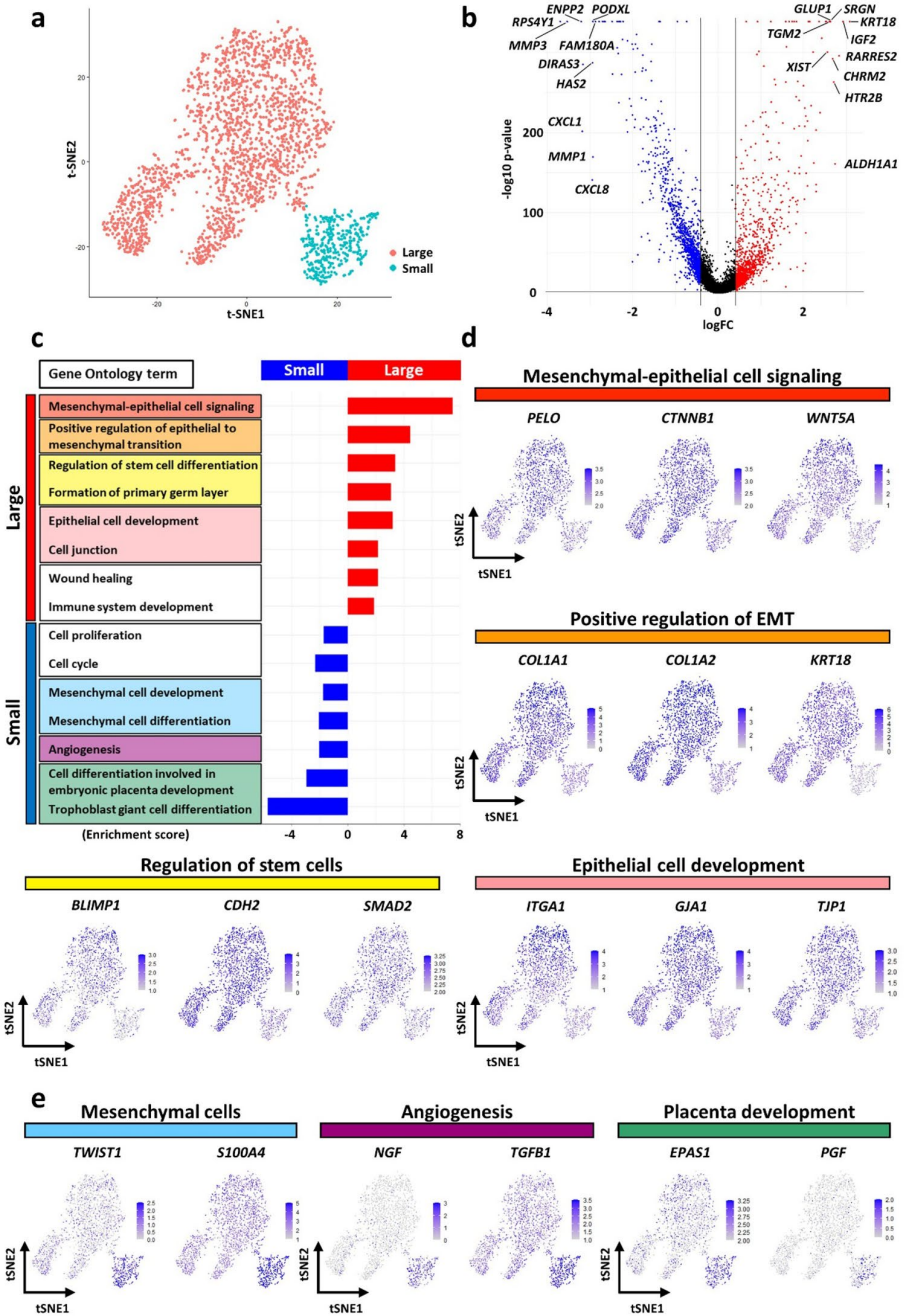
Subpopulation analysis in hAM-Muse cells by scRNA-seq. (**a**) t-SNE plot of the large (red) and small (blue) subpopulations in hAM-Muse cells. (**b**) Volcano plot displaying gene expression levels in the large subpopulation compared with the small subpopulation. Significantly differentially expressed genes were defined as those with a fold-change > 1.5 times or < 0.67 times in the large subpopulation compared with the small subpopulation, with *p* < 0.05. Upregulated genes are shown in red, downregulated genes are shown in blue, and the black lines represent the boundary for identification of upregulated or downregulated genes based on the *p*-value and fold-change. Top 10 differentially expressed genes in the large and small subpopulations are shown. (**c**) GO analysis; upregulated genes in the large (red) and small subpopulation (blue) are listed. Enrichment score of each term is shown. (**d**) t-SNE plots showing the expression levels of representative genes related to M-E cell signaling, positive regulation of EMT, regulation of stem cells, and epithelial cell development. Each term was upregulated in the large subpopulation. (**e**) t-SNE plots showing the expression levels of representative genes related to angiogenesis, mesenchymal cells, and placenta development. Each term was upregulated in the small subpopulation.

When hAM-Muse cells were further analyzed in scRNA-seq by t-SNE plot, they were separated into large (red clusters; 73.7%) and small (blue clusters; 26.3%) subpopulations (Fig. 3a). Each cluster contained cells in all periods of the cell cycle; G1, G2 and M, and S at proportions of 59%, 29%, and 12% (large subpopulation), and 8%, 66%, and 26% (small subpopulation), respectively (Supplementary Fig. 7a). Volcano plots revealed that 2530 genes were significantly altered (FC > 1.5, *p* < 0.05) between the large and small subpopulations; 1249 genes were upregulated in the large subpopulation (red dots) and 1281 genes were upregulated in the small subpopulation (blue dots) (Fig. 3b, Supplementary Table 2).

We identified the top 10 genes that showed significant differences in expression levels; in the large subpopulation, *KRT18*, insulin like growth factor 2 (*IGF2*), *RARRES2*, *ALDH1A1*, *HTR2B*, *CHRM2*, serglycin (*SRGN*), *GULP1*, X inactive specific transcript (*XIST*), and transglutaminase 2 (*TGM2*) were upregulated; and in the small subpopulation, ribosomal protein S4 Y-linked 1 (*RPS4Y1*), matrix metallopeptidase 3 (*MMP3*), ectonucleotide pyrophosphatase 2 (*ENPP2*), C-X-C motif chemokine ligand 1 (*CXCL1*), distinct subgroup of the Ras family member 3 (*DIRAS3*), *CXCL8*, hyaluronan synthase 2 (*HAS2*), podocalyxin like (*PODXL*), *MMP1*, and family with sequence similarity 180 member A (*FAM180A*) were upregulated (Fig. 3b).

GO analysis demonstrated that the large subpopulation was enriched for genes involved in specific functions such as mesenchymal-epithelial (M-E) cell signaling (red in Fig. 3c), positive regulation of epithelial to mesenchymal transition (EMT, orange), regulation of stem cell differentiation and formation of primary germ layer (yellow), epithelial cell development and cell junction (pink), and wound healing and immune system development (Fig. 3c). In contrast, the functions of the upregulated genes in the small subpopulation were related to cell proliferation and cell cycle, mesenchymal cell development and differentiation (blue), angiogenesis (purple), and placenta development (green) (Fig. 3c).

We visualized the differences in gene expression between the 2 subpopulations using t-SNE as representative genes of each term (Fig. 3d). *PELO*, catenin beta 1 (*CTNNB1*), and *WNT5A*, which are related to M-E cell signaling, were upregulated in the large subpopulation. *PELO* is particularly related to the mesenchymal-to-epithelial transition (MET)^35^. *CTNNB1* inhibits tight junctions in epithelial cells and induces them to be more mobile with looser junctions like the mesenchymal phenotype^36^. *WNT5A* promotes epithelial cells inhibiting the *CTNNB1* pathway and proliferation^37^. Collagen type I alpha 1 chain (*COL1A1*), *COL1A2*, and *KRT18* are involved in EMT, particularly epithelial phenotype markers^38^. *BLIMP1*, *CDH2,* and *SMAD2* relate to stem cell differentiation regulation^39–41^. *BLIMP1* is also essential for early primordial germ cell (PGC) development and relevant to the primary germ layer formation^39^. *ITGA1* expression is higher in epithelial cells, and gap junction protein alpha 1 (*GJA1*) and tight junction protein 1 (*TJP1*) are cell junction components^38^.

*TWIST1* and S100 calcium binding protein A4 (*S100A4*) are related to EMT, and are mesenchymal phenotype markers^38^. Representative genes related to angiogenesis, which were upregulated in the small subpopulation, are nerve growth factor (*NGF*) and *TGFB1*. Although *NGF,* which is also known as neurotrophin, is also suggested to promote angiogenesis^42^ (Fig. 3e). Endothelial PAS domain protein 1 (*EPAS*) is expressed in the endothelial blood vessel cells in the umbilical cord and placental growth factor (*PGF*) is involved in placental development^43^.

These results suggest the potential of the large subpopulation to commit to epithelial lineage cells and a more undifferentiated state, in contrast to the small subpopulation, which was more related to a mesenchymal state and is enriched for genes relevant to the placental formation.

We also used t-SNE to visualize the relationship between female- and male-origin hAM-Muse cells. hAM-Muse cells from males and females were contained in the same cluster, and not separated each other (Supplementary Fig. 7b). Only 15 genes were significantly altered (FC > 1.25, *p* < 0.05) between them; 14 genes were upregulated in male-origin and 1 gene was upregulated in female-origin cells (Supplementary Table 3). GO analysis demonstrated that the genes upregulated in male-origin cells were involved in specific functions, such as placenta development, particularly placenta blood vessel and embryonic placenta development (Supplementary Fig. 7c). Representative genes of placenta development are shown in Violin plots; *WNT2*, *FOS*, *IGFBP5*, *JUNB*, suppressor of cytokine signaling 3 (*SOCS3*), and *ZFP36* (Supplementary Fig. 7d).

### In vitro differentiation into germ cell-lineage marker (+) cells

**Figure 4:**
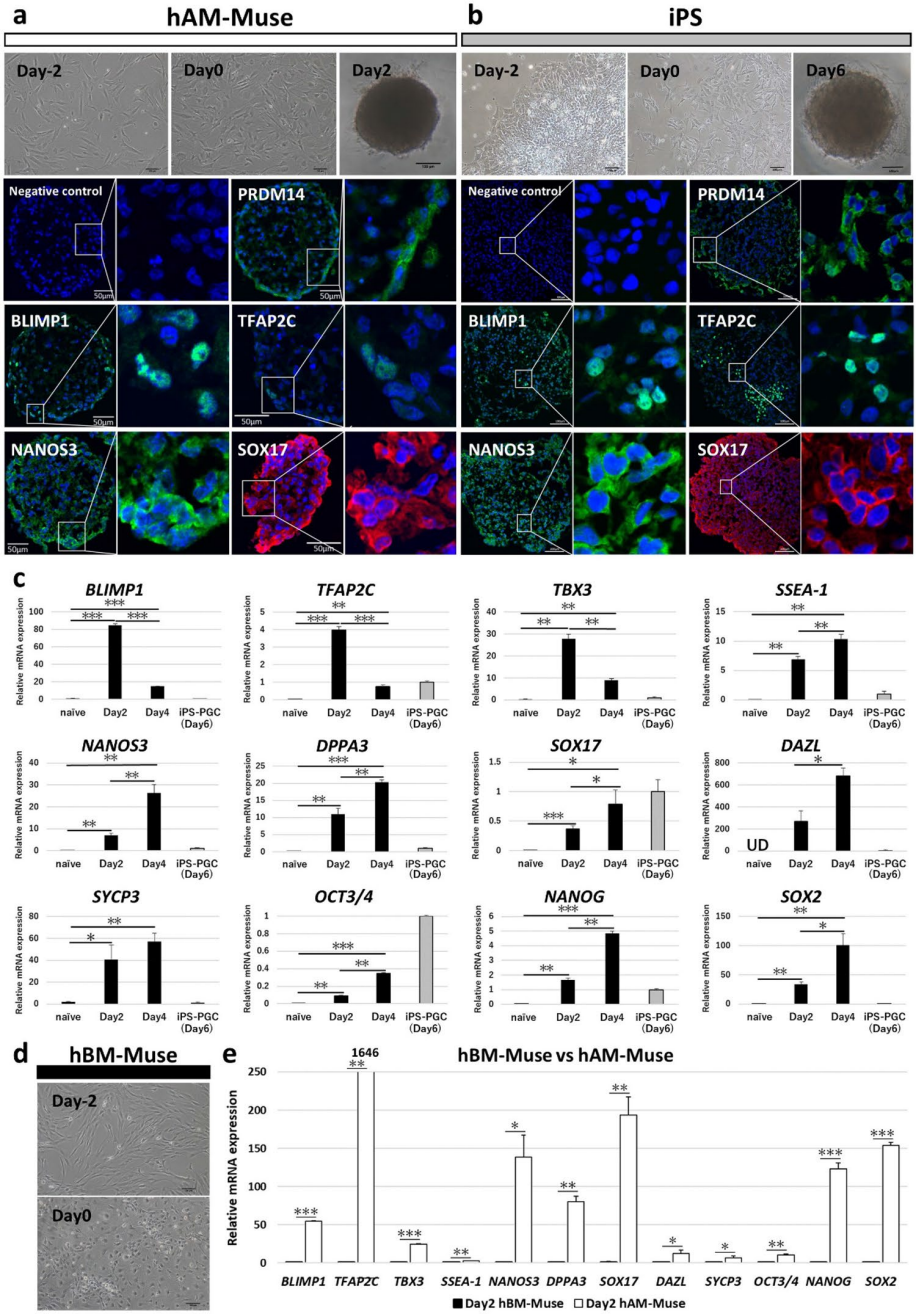
In vitro differentiation of hAM-Muse cells into germ cell-lineage marker (+) cells. (**a**, **b**) iMeLC and hPGCLC induction in hAM-Muse (**a**) and iPS (**b**) cells. Aggregations of hAM-Muse and iPS cells were subjected to immunocytochemistry for germ cell-lineage markers (PRDM14, BLIMP1, TFAP2C, NANOS3, and SOX17). (Immunocytochemistry in hAM-Muse cells, bars = 50 µm; others, bars = 100 µm). (**c**) Expression of germ cell- and pluripotency-related genes in naïve hAM-Muse cells, day 2 and day 4 after hPGCLC induction and iPS cells at day 6 after hPGCLC induction (normalized by *ACTB*). Values of induced iPS cells were set as 1. *p < 0.05; **p < 0.01; ***p < 0.001; UD = under detected. (**d**) hBM-Muse cells after iMeLC induction. (bars = 100 µm). (**e**) Expression of germ cell- and pluripotency-related genes compared between hBM- with hAM-Muse cells at day 2 after induction (normalized by *ACTB*). Values of induced hBM-Muse cells were set as 1. *p < 0.05; **p < 0.01; ***p < 0.001.

hAM-Muse and human induced pluripotent stem (iPS) cells (as positive control) were cultured in medium containing activin A and a WNT-agonist with minor modifications for 2 days in adherent culture to differentiate the cells into incipient mesoderm-like cells (iMeLCs). After iMeLC induction, the cells were cultured in bone morphogenetic protein 4 (BMP4), leukemia inhibitory factor, stem cell factor, EGF, Rho-associated coiled-coil forming kinase (ROCK) inhibitor on suspension culture to differentiate them into human primordial germ cell-like (hPGCLCs), as reported previously^44,45^. After hPGCLC induction, both hAM-Muse (at day 2) and iPS cells (at day 6) became positive for PRDM14, essential for PGC specification (especially in mice^34,46^) in immunocytochemistry. hAM-Muse and iPS cells also expressed other PGC markers, such as BLIMP1, TFAP2C, NANOS3, and SOX17, in immunocytochemistry (Fig. 4a,b). qPCR revealed that hAM-Muse cells expressed significantly higher levels of early PGC markers such as *BLIMP1* and *TFAP2C*^44^, and *TBX3* (activating the promoter of *SOX17*), compared with naïve hAM-Muse cells at day 2 and day 4 after hPGCLC induction (*BLIMP1*; both day 2 and 4 *p* < 0.001, *TFAP2C*; *p* < 0.001 and *p* < 0.01, *TBX3*; both *p* < 0.01), and expression was significantly higher at day 2 than at day 4 (*BLIMP1* and *TFAP2C*; *p* < 0.001, *TBX3*; *p* < 0.01; Fig. 4c). In contrast, *SSEA-1*, *NANOS3*, *DPPA3*, and *SOX17*, which are suggested to be only slightly expressed after these early PGC markers^34,44^, and the middle-to-late PGC markers such as deleted in azoospermia like (*DAZL*), were more highly expressed at day 4 than at day 2 of induction (*SSEA-1*, *NANOS3* and *DPPA3*; *p* < 0.01, *SOX17* and *DAZL*; *p* < 0.05) (Fig. 4c). Synaptonemal complex protein 3 (*SYCP3*)^34,44^ expression also tended to be higher at day 4 than at day 2 of induction. Expression of key pluripotency genes *OCT3/4*, *NANOG*, and *SOX2* was significantly increased^34^ at day 2 and day 4 of induction compared with naïve hAM-Muse cells (*OCT3/4* and *NANOG*; *p* < 0.01 and *p* < 0.001, *SOX2*; both *p* < 0.01; Fig. 4c).

hBM-Muse cells treated with the same method at day 0 exhibited a different morphology than hAM-Muse cells and iPS cells at day 0 (Fig. 4d). The gene expression of germ cell- and pluripotency-related markers at day 2 after hPGCLC induction was significantly lower in hBM-Muse cells than in hAM-Muse cells (Fig. 4e).

### In vitro differentiation into extraembryonic-marker (+) cells

**Figure 5:**
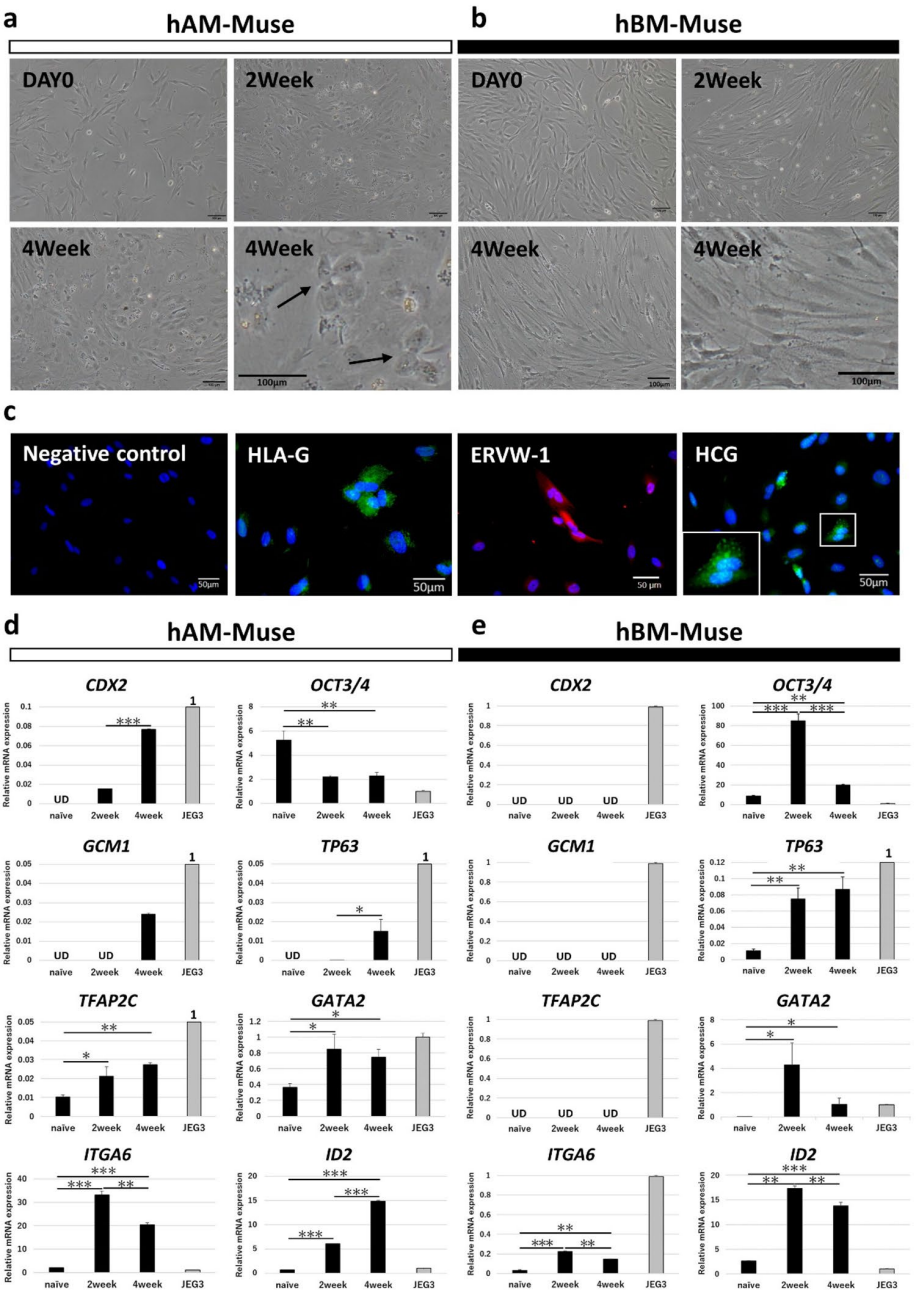
In vitro differentiation of hAM-Muse cells into extraembryonic-marker (+) cells. (**a**, **b**) Extraembryonic-lineage induction of hAM- (**a**) and hBM-Muse cells (**b**). hAM-Muse cells at 4 weeks after induction (a) assumed a different morphology than naïve cells and exhibited fusion-like cells (arrows; bars = 100 µm). (**c**) Immunocytochemistry for extraembryonic-markers (HLA-G, ERVW-1, and HCG) in hAM-Muse cells at 4 weeks after induction (bars = 50 µm). In HCG, fusion of the two cells was observed (see high magnification). (**d**, **e**) Expression of extraembryonic-related genes in naïve hAM- (**d**) and hBM-Muse (**e**), 2 and 4 weeks after induction and in JEG3 (normalized by *ACTB*). Values of JEG3 were set as 1. *p < 0.05; **p < 0.01; ***p < 0.001; UD, under detected.

For extraembryonic-lineage induction, hAM-Muse and hBM-Muse cells were cultured in medium containing BMP4 (100 ng/mL), SB431542, and SU5402, according to previous reports with minor modifications for 4 weeks^47,48^. hAM-Muse cells developed a high nucleus-cytoplasm ratio, and cell–cell adhesion and membrane fusion were observed at 4 weeks (Fig. 5a). These morphologic features were similar to cytotrophoblasts (CT), the progenitors of syncytiotrophoblasts and extravillous trophoblasts^49,50^. On the other hand, the morphology of the hBM-Muse cells after induction slightly changed compared with the cells in naïve state while CT-like cells were not identified even at 4 weeks (Fig. 5b).

hAM-Muse cells were observed to adhere to the culture medium and were immunohistochemically positive for HLA-G, which is expressed in placental CT; endogenous retrovirus group W member 1 envelope (ERVW-1), which has an important role in placentation as well as glycoprotein hormones; and alpha polypeptide (HCG), produced in the placenta (Fig. 5c). Fusion of hAM-Muse cells was also observed (Fig. 5c, see HCG). Naïve hAM-Muse cells were negative for these markers (Supplementary Fig. 7e).

In qPCR, *CDX2* (trophoblast stem [TS] cell marker^48^), originally under the detection limit in naïve hAM-Muse cells, was newly expressed at 2 weeks and expression was significantly higher at 4 weeks than at 2 weeks (*p* < 0.001; Fig. 5d). The primary function of *CDX2* is to block *OCT3/4* in the trophectoderm, the first differentiated cell lineage in mammalian embryogenesis, which triggers the commitment of embryonic cells to the trophectoderm lineage^51^. Thus, once *CDX2* is expressed, *OCT3/4* is suppressed, and both are thus mutually exclusively expressed in the 2 different cell lineages, the inner cell mass and trophectoderm^51^. As the induction progressed in hAM-Muse cells, *CDX2* increased and *OCT3/4* significantly decreased compared with naïve hAM-Muse cells at 2 and 4 weeks (both *p* < 0.01) (Fig. 5d). *GCM1* (placenta-specific transcription factor involved in the placenta development) and *TP63* (undifferentiated trophoblast cell marker^52^), were newly expressed either at 2 or 4 weeks and *TP63* was significantly increased at 4 weeks compared with that at 2 weeks (*p* < 0.05; Fig. 5d). Expression of *TFAP2C* and *GATA2* (both accelerate the differentiation of trophoblast and TS lineages^53^), as well as *ITGA6* (CT marker) and *ID2* (undifferentiated placenta marker), all originally expressed at low levels in naïve hAM-Muse cells, was significantly increased at 2 weeks compared with the naïve state (*TFAP2C* and *GATA2*; *p* < 0.05, *ITGA6* and *ID2*; *p* < 0.001), and the higher level was maintained at 4 weeks (Fig. 5d). In contrast to hAM-Muse cells, expression of *CDX2*, *GCM1*, *TFAP2C* was consistently under the limits of detection in hBM-Muse cells until 4 weeks, while expression of *TP63* (*p* < 0.01), *GATA2* (*p* < 0.05), *ITGA6* (*p* < 0.001), and *ID2* (*p* < 0.01) was significantly increased at 2 weeks compared with cells in the naïve state (Fig. 5e). Furthermore, in contrast to hAM-Muse cells, *OCT3/4* was significantly upregulated in hBM-Muse cells at 2 weeks compared with cells in the naïve state (*p* < 0.001; Fig. 5e).

## Discussion

**Figure 6:**
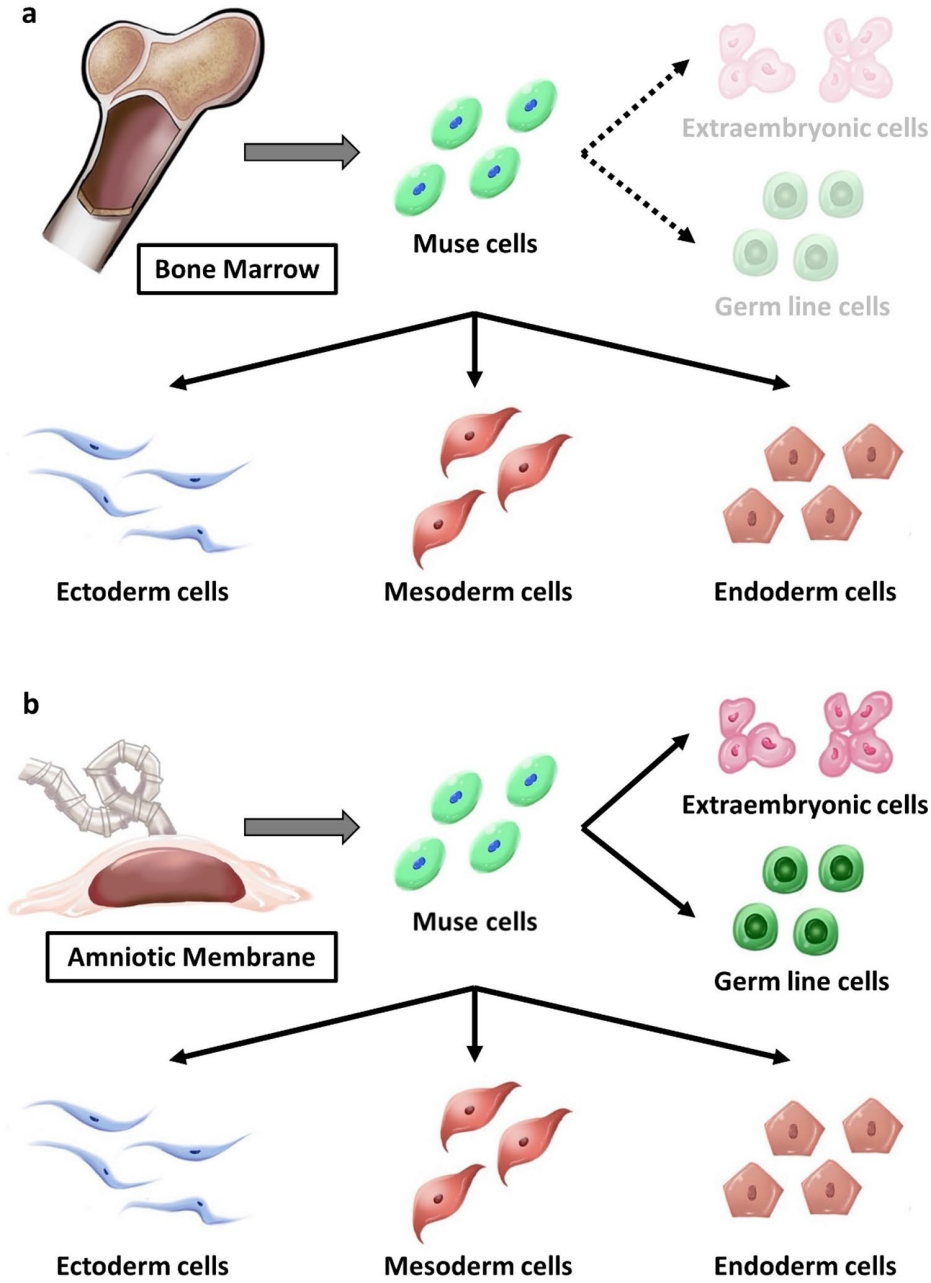
Differentiation potential of hBM- and hAM-Muse cells. (**a**, **b**) Schematic images of the differentiation range of hBM- (**a**) and hAM-Muse cells (**b**). Both hBM- and hAM-Muse cells differentiated into cells representative of all 3 germ layers, but hBM-Muse cells exhibited a partial response to germline and extraembryonic cell inductions (**a**), while hAM-Muse cells are suggested to have a broader differentiation potential into not only triploblastic-lineages, but also germline- and extraembryonic-lineages (**b**).

The new findings of this study are as follows:

1. hAM-Muse cells, isolated from human hAMSCs as SSEA-3(+) cells, expressed pluripotency markers, and were capable of generating cells representative of all 3 germ layers from a single cell and self-renewing. They exhibited low telomerase activity and did not form teratoma in vivo until 4 months. These properties are similar to those of Muse cells isolated from the BM, dermis, peripheral blood, and adipose tissue^8,12,27,30–32^.
2. On the other hand, more than 70% of hAM-Muse cells were positive for CD133, which was expressed in only ~ 2.0% of hBM-Muse cells. The hBM-Muse cells were 100% positive for mesenchymal markers such as CD44, CD90 and CD105, while hAM-Muse cells were positive for those markers at ~ 70%–80%. In addition, hAM-Muse cells expressed germ cell-lineage and placental-lineage markers when treated with germ-cell and placental induction systems, while hBM-Muse cells responded to a lesser extent than hAM-Muse cells.
3. Thus, although hAM- and hBM-Muse cells both shared pluripotent-like properties, the surface marker expression and potential differentiation range differed between them. Because hAM-Muse cells exhibited potential for differentiation into germ-cell and placental lineages, compared with hBM-Muse cells, they were more like naïve pluripotent stem cells that differentiate into germ-line cells and/or extraembryonic cell-lineage cells in addition to triploblastic-lineage cells^21,22^ (Fig. 6a,b).
4. scRNA-seq demonstrated that hAM-Muse cells comprised 2 major subpopulations. The large subpopulation seemed to be in a state between the epithelial- and mesenchymal-cell states, while the small subpopulation was in a more mesenchymal state and was enriched for genes relevant for placental formation.

### Comparison between hAM- and hBM-Muse cells

The hAM-Muse cells had several unique features compared with hBM-Muse cells.

Gene expression levels related to pluripotency differed. qPCR and scRNA-seq demonstrated that *NANOG*, *SOX2*, *KLF2*, and *SMAD2* levels were significantly higher in hAM-Muse cells compared with hBM-Muse cells. In addition, the metabolic system is suggested to differ between them; genes related to oxidative phosphorylation were expressed at higher levels in hAM-Muse cells (Supplementary Fig. 5a) but those related to glycolysis and hypoxia-inducible factor 1 signaling pathway were expressed at higher levels in hBM-Muse cells (Supplementary Figs. 4, 5b).

Interestingly, the metabolic activity of totipotent stem cells is reported to shift to oxidative phosphorylation where ATP is produced by the electron transport chain in mitochondria rather than to glycolysis due to the low activities of the rate-limiting enzymes hexokinase and phosphofructokinase 1^54^. This tendency is also recognized in primordial germ cells, primary oocytes, and Sertoli cells^55^. In contrast to those cells, adult stem cells such as MSCs depend on glycolysis rather than oxidative phosphorylation for protection against active oxygen-induced damage^56,57^. Besides, the metabolism of naïve pluripotent stem cells is more dependent on oxidative phosphorylation and that of primed pluripotent stem cells on glycolysis^22^. Quiescent hematopoietic stem cells activate hypoxia-inducible factor 1α, which in turn promotes glycolysis and prevents pyruvate oxidation by suppressing pyruvate dehydrogenase complex^54^. In these respects, hAM-Muse cells are more similar to totipotent stem cells than to adult stem cells, and in contrast, hBM-Muse cells are more similar to typical human adult stem cells than to totipotent stem cells, in terms of metabolic activity.

### Differentiation potential of hAM- and hBM-Muse cells

In the present study, hAM-Muse cells were suggested to differentiate not only into 3 germ layers cells, but also into germ cell- and extraembryonic-lineages, and thus have broader differentiation potential than hBM-Muse cells (Fig. 6a,b).

Naïve hAM-Muse cells expressed markers related to germ line cells at levels higher than those in naïve hBM-Muse cells. hAM-Muse cells also responded efficiently to the PGC induction^44^ compared with hBM-Muse cells; after induction, hAM-Muse cells expressed higher levels of genes relate to germ cells compared with hBM-Muse cells (Fig. 4c,e).

The origination of human PGCs remains under debate^58^. In mouse and swine, PGCs are suggested to originate in a specific region of the peri-implantation embryo called the posterior proximal epiblast^59^, where the AM originates before gastrulation. In cynomolgus macaques (Macaca fascicularis; ‘cyno’), PGCs are first specified in an extraembryonic tissue called the amnion and possibly the posterior epiblast^45^. In this manner, the AM is strongly related to the origin of PGCs and is also continuous with epiblasts in mouse and swine. Indeed, epiblasts play an important role in germ cell induction; in germ cell induction in iPS and ES cells, they differentiate initially into epiblast-like cells (EpiLCs) and/or iMeLCs, induced from epiblasts during early development^44^. It is possible that residual PGC-like cells and/or cells that produce essential factors for the development and maintenance of PGCs remain in the AM. If these cells join hAM-Muse cells as a subpopulation, they may support the expression of genes involved in germline cells in hAM-Muse cells.

hBM-Muse cells did not exhibit such differentiational changes, and some of extraembryonic markers were under the detection limit until 4 weeks after induction. With regard to extraembryonic differentiation, hAM-Muse cells differentiated into CT-like cells and newly expressed or showed significant upregulation of extraembryonic markers compared with the naïve state in immunocytochemistry and qPCR. There is a possibility that these differentiation-related marker expressions were owing to trophoblasts/villi cells contaminated into hAM-Muse cells and not by differentiation of hAM-Muse cells. However, such contamination is unlikely because multiple genes involved in trophoblasts/villi cells (HLA-G, ERVW-1, HCG, *CDX2*, *GCM1* and *TP63*) were under the detection in naïve hAM-Muse cells. Thus, hAM-Muse cells, are suggested to have higher potential to differentiate into extraembryonic cells compared with hBM-Muse cells.

In scRNA-seq, hAM-Muse cells obtained from human males were enriched for genes related to placentation compared with those obtained from human females. Parthenogenetic and androgenetic embryos have 2 sets of maternal and paternal genomes. Parthenogenetic embryos can develop up to the 25-somite stage, but with very limited development of extraembryonic tissues^60,61^. On the other hand, trophoblast development is very much active in androgenetic embryos compared with parthenogenetic embryos, while the development of androgenetic embryos is substantially suppressed^60–62^. These results imply that the maternal genomes are more responsible for the differentiation of embryos and the paternal genomes are more responsible for the differentiation of trophoblast cells^63^. Paternal genes in germ cells are also deeply involved in placental development. Activation of more than one active paternal chromosome causes hyperplasia of the placenta in mouse embryogenesis^64^. Therefore, the paternal X chromosome is preferentially inactivated in the extraembryonic region in mice, and this process is termed paternal X-inactivation^65,66^.

As hAM-Muse cells exhibit germ cell-like characteristics, it is conceivable that hAM-Muse cells of male origin have a high placental potential and are thus enriched for genes related to placentation. This hypothesis however, must be examined by in-depth analysis of multiple different batches of hAM-Muse cells.

### Two different subpopulations of hAM-Muse cells

ScRNA-seq analysis separated hAM-Muse cells into a large group and a small group. The large subpopulation was enriched for genes involved in interactions with epithelium and mesenchymal cells, such as mesenchymal-epithelial cell signaling and EMT. In addition, genes related to epithelial development and basal membrane integrity, were also expressed more highly in the large subpopulation compared with the small subpopulation. Therefore, the large subpopulation is suggested to have the potential to commit to epithelial lineage cells. Previous transmission electron microscopy observations showed that hAMSCs, unlike general MSCs, had a hybrid epithelial-mesenchymal ultrastructural phenotype; epithelial characteristics included non-intestinal-type surface microvilli, intracytoplasmic lumina lined with microvilli, and intercellular junctions, while mesenchymal features included rough endoplasmic reticulum profiles. Lipid droplets, and well-developed foci of contractile filaments with dense bodies^67^. Therefore, hAMSCs are assumed to include cells that were originally converted from amniotic epithelial cells to mesenchymal cells by EMT^68^. Since hAMSCs were negative for CD326 (Supplementary Fig. 1d), the contamination of epithelial cells into hAMSCs was unlikely. Consequently, the epithelial-like properties seen in the large subpopulation might be due to the presence of such mesenchymal cells in a state between the epithelial- and mesenchymal-cell states. The fact that the 10–20% of hAM-Muse cells were negative for mesenchymal markers might be relevant to their uniqueness. A recent study suggested that the intermediate cellular state between the fully mesenchymal and fully epithelial states is necessary for the maintenance of pluripotency in the epiblast^69^. GO analysis showed that the large subpopulation was enriched for genes related to the regulation of stem cell differentiation and formation of the primary germ layer. In contrast to the large subpopulation, the small subpopulation was more in a mesenchymal state and enriched for genes relevant to placental formation. This might be because the early amnion was in contact with trophoblasts and interacted with the placenta during the first 10–11 days of development before the formation of the extraembryonic mesoderm (later the chorionic cavity)^70^.

### Significances of AM-Muse cells in the future

As a new source of Muse cells, AM-Muse cells are useful because they have several practical advantages for clinical application including availability and non-additional invasive. Despite being non-tumorigenic, hAM-Muse cells were suggested to have potential like naïve pluripotent stem cells (differentiating into 3 germ layers, germ cell- and extraembryonic-lineages cells) and epiblast (being in a state between the epithelial- and mesenchymal-cell states). Thus, hAM-Muse cells might be a population of unique cells from the hAMSCs that were reported to be similar to epiblast and exhibit properties relevant to the early stage of development. This character implicated that hAM-Muse cells could be useful as a germ cell model without ethical issues and tumorigenic, and helpful to identify the origination of human PGCs. Furthermore, we demonstrated the presence of SSEA-3(+) cells in the mouse amniotic membrane and that the characteristics of mouse AMSC-SSEA-3(+) cells were similar to those of hAM-Muse cells. The functionality of hAM-Muse cells and mAM-Muse cells in vivo is an interesting point to be investigated in the future.

## Limitations

Further studies are needed to determine whether AM-Muse cells also show the ability to differentiate into germline- and extraembryonic-lineage cells in vivo. If AM-Muse cells have the ability to differentiate into functional germline cells and extraembryonic cells, that will be a beneficial to basic research as well as to clinical application.

## Methods and materials

### Animals

ICR mice were used in this study. All animals were treated according to the regulations of the Standards for Human Care and Use of Laboratory Animals of Tohoku University. The animal experiments were approved by the Animal Care and Experimentation Committee of Tohoku University Graduate School of Medicine (permission No. 103-2).

The study is reported in accordance with ARRIVE guidelines.

### Human AMSCs culture

Human AMSCs (hAMSCs) were purchased from Cellular Engineering Technologies (Coralville, IA, USA). Cells were cultured at 37 °C in 5% CO_2_ in alpha modified Eagle’s medium (alpha-MEM; MilliporeSigma, St. Louis, MO, USA) containing 10% fetal bovine serum (FBS; HyClone, Logan, UT, USA), 1% GlutaMAX (Invitrogen, Carlsbad, CA, USA), 1 ng/mL basic fibroblast growth factor (Miltenyi Biotec, Bergisch Gladbach, Germany), and 0.1 mg/mL kanamycin sulfate (Invitrogen). The cells were subcultured when they reached 90% confluence. For all experiments, hAMSCs were used at passages 4 to 9.

### Mouse AMSCs culture

The detailed method of the primary culture of mouse AMSCs (mAMSCs) isolated from mouse placenta is described in the Supplementary Methods. Cells were maintained in MesenPure medium (VERITAS, Tokyo, Japan) at 37 °C in 5% CO_2_, and culture medium was exchanged every other day to remove hematopoietic cells. The cells were subcultured when they reached 80% confluence. For all experiments, mAMSCs were used at passages 1 to 3.

### Preparation of human and mouse AMSCs and Muse cells

SSEA-3(+) human and mouse AMSCs were collected from human AMSCs and mouse AMSCs as SSEA-3(+) cells by FACS as described in the Supplementary Methods.

### Single-cell suspension culture

AMSC-SSEA-3(+) cells were cultured in single-cell suspension culture in poly-HEMA–coated dishes, as previously described^71^. Single cells were plated in individual wells of 96-well plates after limiting dilution of the collected cells with alpha-MEM medium containing 10% FBS. The actual number of cells deposited in each well was determined by visual inspection using a phase-contrast microscope, and empty wells or wells with more than 1 cell were excluded from analysis.

### Spontaneous differentiation of clusters in vitro

After 7–10 days of single-cell suspension culture, single clusters of hAMSC-SSEA-3(+) cells were picked up with a glass micropipette and transferred onto a gelatin-coated culture dish. After another 7–10 days of incubation, clusters were subjected to immunocytochemistry.

### Immunocytochemistry

Immunocytochemistry was performed as previously described^71^. Cells expanded from a single hAMSC-SSEA-3(+) cell-derived cluster were grown in gelatin-coated dishes. Cells were fixed with 4% paraformaldehyde (PFA) in 0.01 M phosphate buffered saline (PBS). Primary antibodies used in this study were described in the Supplementary Methods. All primary antibodies were diluted in PBS/0.1% bovine serum albumin (BSA) solution and incubated overnight at 4 °C. Following treatment with primary antibodies, the cells were washed 3 times with PBS and incubated for 1 h at room temperature with PBS/0.1% BSA containing secondary antibodies either of FITC-, Alexa-488-, Alexa-568, or Alexa-594-labeled conjugated anti-rabbit IgG, anti-goat IgG, anti-mouse IgG, anti-mouse IgM, or anti-rat IgM (1:100; Jackson ImmunoResearch, West Grove, PA, USA). Nuclei were identified by 4’,6-diamidino-2-phenylindole (DAPI) staining (1:1000; MilliporeSigma). Cells were then washed 3 times with PBS. Images were acquired with a confocal laser scanning microscope (CS-1; Nikon, Tokyo, Japan).

Mouse AM was fixed with 4% PFA, embedded in OCT compound, and then cut into 10-µm-thick cryosections. For staining, samples were washed with PBS; incubated with 20% Block Ace (skim milk; Yukijirushi), 5% BSA, and 0.3% Triton X-100 in PBS at room temperature for 30 min; and then incubated overnight at 4 °C with primary antibodies diluted in antibody diluent solution for immunohistochemistry (0.02 M PBS supplemented with 5% Block Ace, 1% BSA, and 0.3% Triton X-100). The primary antibody used was SSEA-3 (Millipore, MAB4303, 1:100). After 3 washes with PBS, the slides were incubated with anti-rat IgM (Jackson Immunoresearch, 1:200) antibody conjugated with Alexa-594 under the presence of DAPI in the antibody diluents for 2 h at room temperature. Samples were inspected with a Nikon C1si confocal microscope system.

### Evaluation for cell self-renewal

Self-renewal of hAMSC-SSEA-3(+) cells was examined as previously described^71^. Briefly, hAMSC-SSEA-3(+) cells isolated by FACS were grown in single-cell suspensions after limiting dilution to generate the first-generation cluster. After 7–10 days of the suspension culture, first-generation clusters were transferred onto an adherent culture without coating for expansion. After another 7 days, expanded cells were collected by trypsinization and subjected to the second-round of single-cell suspension culture after limiting dilution to form second-generation clusters. This cycle was repeated for up to third-generation clusters.

### Quantitative PCR (qPCR)

Total RNA was collected using the NucleoSpin® RNA XS (Macherey–Nagel, Duren, Germany), and cDNA was synthesized using Oligo(dT)20 primers (Invitrogen) and SuperScript® III reverse transcriptase (Invitrogen). DNA was amplified with the Applied Biosystems 7500 Fast real-time PCR system according to the manufacturer’s instructions. Data were processed by using the ΔΔCT method^72^. Details regarding the use of the primers are described in the Supplementary Methods.

### Droplet digital-telomere repeat amplification protocol (ddTRAP)

Telomerase extension reaction was measured by ddTRAP^26^. Whole cell lysates were prepared and added to an extension mix containing the telomerase substrate primer (TS primer) and a mixture of deoxynucleoside triphosphates. The extension reaction was heat-inactivated, and the products were then amplified by PCR in the presence of the reverse primer ACX and the forward primer TS to amplify the telomerase-extended substrates. The PCR products were detected using Evagreen® dsDNA binding dye in the QX100/200 droplet digital PCR reader. HeLa cells were used as positive control.

### Single-cell RNA-sequencing (scRNA-seq)

Single-cell capture and cDNA synthesis were performed based on the TAS-seq protocol using Rhapsody Single-Cell Analysis System (Becton Dickinson, Franklin Lakes, NJ, USA)^73^. All libraries were sequenced using Novaseq 6000 (Illumina, San Diego, CA, USA). After adapter trimming and filtering, sequenced reads were mapped to reference RNA (build GRCh38 release-101) using bowtie2-2.4.2^74^. The sequencing and initial analyses were performed by ImmunoGeneTeqs (Tokyo, Japan). Seurat R package v3.2.2 was used for the following analyses^75^. Low-quality cells with < 5000 expressed genes and < 1 or > 10% mitochondrial genes and genes that were detected in fewer than 3 cells were excluded. The Seurat "SCtransform" function performed normalization and regressed out the percent of mitochondrial genes^76^. Unsupervised clustering was performed by the Seurat "FindClusters" function. Principal component analysis was performed using the Seurat "RunPCA" function, and the top 30 principal components were used to generate t-SNE plot using the Seurat "RuntSNE" function^77^. We used the Seurat "FindMarkers" function with the MAST algorithm to identify differentially expressed genes^78^. The Database for Annotation, Visualization, and Integrated Discovery (DAVID: http://david.abcc.ncifcrf.gov) was used for the GO analysis^79^. Supplemental Information provides detailed experimental procedures.

### In vitro differentiation into germline-lineage marker (+) cells

The previously described method of differentiation into germline cells was used^44^ with minor modifications. hAM-Muse cells (10,000 cells/cm^2^) were cultured in alpha-MEM containing 10% FBS, 1% GlutaMaX, 0.1 mg/mL kanamycin sulfate, 50 ng/mL Activin A (014-23961, Wako, Osaka, Japan), 3 µM GSK3 inhibitor (039–20,831, Wako), and 10 µM ROCK inhibitor (030-24021, Wako) on adherent culture for 2 days (iMeLC induction). After 2 days of iMeLC induction, the cells were cultured in alpha-MEM containing 10% FBS, 1% GlutaMaX, 0.1 mg/mL kanamycin sulfate, 200 ng/mL BMP4 (022-17071, Wako), 1000 U/mL leukemia inhibitory factor (LIF1005, Milipore), 100 ng/mL stem cell factor (197-15511, Wako), 50 ng/mL (EGF; 059-07873, Wako), and 10 µM ROCK inhibitor on suspension culture using low-cell-binding V-bottom 96-well plates (3000 cells/well) and formed aggregations at 37 °C in 5% CO_2_. Aggregations of hAM-Muse cells after 2 or 4 days were subjected to qPCR. Aggregations after 4 days were fixed with 4% PFA in 0.01 M PBS, embedded in OCT compound, and cut into 8-µm-thick cryosections. These cryosections were subjected to immunocytochemistry as described above. Human iPS cells were induced by the same method for 6 days and used as a positive control.

### In vitro differentiation into extraembryonic-lineage marker (+) cells

Extraembryonic differentiation was performed according to the previously described method^47,48^ with minor modifications. hAM-Muse cells (10,000 cells/cm^2^) were cultured in alpha-MEM containing 5% FBS, GlutaMaX (100×), 0.1 mg/mL kanamycin sulfate, BMP4 (100 ng/mL), SB431542 (20 µM; 031-24291, Wako), and SU5402 (20 µM; 197-16731, Wako). Cell cultures were maintained at 37 °C in 5% CO_2_. hAM-Muse cells after 2 or 4 weeks were subjected to qPCR. hAM-Muse cells after 4 weeks were fixed with 4% PFA in 0.01 M PBS and were subjected to immunocytochemistry. JEG3 purchased from American Type Culture Collection was used as positive control.

### Statistical analysis

All experiments were conducted with at least 3 biological replicates. Results are presented as mean ± standard deviation (SD) of 3 independent experiments. *P*-values of the difference were determined by *t*-test using Microsoft Excel. A difference was considered significant when the *p*-value was less than 0.05.

## Supplementary Information

Supplementary Information 1.

Supplementary Information 2.

## Data Availability

The datasets used and/or analysed during the current study available from the corresponding author on reasonable request.
