# Supplementary material for: Naïve pluripotent-like characteristics of non-tumorigenic Muse cells isolated from human amniotic membrane
Source: Sci Rep. 2022 Oct 14;12:17222. doi: 10.1038/s41598-022-22282-1 (PMC9568515; doi:10.1038/s41598-022-22282-1)
Supplement: Supplementary file 1 — Supplementary Information 1. [file 41598_2022_22282_MOESM1_ESM.docx]

**Supplementary Method**

**Isolation of mouse AMSCs.**

Embryos were obtained from an ICR mouse at 12–15 days gestation and the non-amniotic tissues were removed. Isolated amniotic tissues were finely chopped using scissors in 0.1% collagenase-dispase (MilliporeSigma) in alpha-MEM and 10% serum. The tissue pieces and cell dissociation solution together were incubated and agitated (37°C, 100 min-1, 30 min) with a water bath shaker. Broken pieces of tissues were filtered with a strainer and centrifuged at 400g for 5 min. The supernatant was discarded and culture medium was added to loosen the cell pellet by gentle pipetting and plated into dishes.

**Fluorescence-activated cell sorting.**

Confluent AMSCs were used for cell sorting. Cells were collected by trypsin-EDTA (0.25%) treatment, centrifuged, and resuspended in fluorescence-activated cell sorting (FACS) buffer (PBS containing 0.5% BSA and 2 mM EDTA) ^1^ at a concentration of 1×10⁶cells/100 mL. Cells were incubated in FACS buffer containing 15% human serum for 20 min. After 2 successive washes with FACS buffer, cells were incubated with anti-SSEA-3 antibody (1:200; Thermo Fisher Scientific) for 1 h at 4°C. Cells were then washed 3 times with FACS buffer, followed by fluorescein isothiocyanate (FITC)-conjugated anti-rat IgM (1:100; Jackson Immuno Research, West Grove, USA) for 1 h at 4°C. After 3 consecutive washes in FACS buffer, cells were sorted into SSEA-3(+) and SSEA-3(-) cells (Muse and non-Muse cells) by Special Order Research Products FACSAriaII (Becton Dickinson, Franklin Lakes, NJ, USA) using a low stream speed. This ensured a high level of cell survival and the highest purity of the sorted cells via the 4-way purity sorting mode, as previously described ^1^. SSEA-3(+)-Muse cells (labeled with FITC) were analyzed by flow cytometry for the expression of cell surface antigens CD326 (APC), CD324 (APC), CD44 (APC), CD90 (APC), CD105 (APC), CD133 (APC), CD34 (PE), and CD45 (APC) (Becton Dickinson). BM Muse cells were used as a positive control for CD44, CD90, and CD105. NTERA-2 (pluripotent human embryonal carcinoma) cell line was used as a positive control for CD326, CD324 and CD133. Mouse amniotic membrane cells were sorted for TER-119 (APC) and CD45 (APC) to remove hematopoietic cells. Double-negative cells indicate MSCs sorted as SSEA-3(+) and SSEA-3(-) (Muse and non-Muse cells). SSEA-3 (+)-Muse cells (labeled with FITC) were analyzed by flow cytometry for the expression of cell surface antigens SSEA-1 (APC), CD29 (APC), CD44 (APC), CD34 (PE), and CD45 (APC) (Becton Dickinson). The F9 (mouse teratocarcinoma) cell line was used as a positive control for SSEA-1.

**Immunocytochemistry.**

Primary antibodies used in this study were SSEA-3 (1:100; Thermo Fisher Scientific, Waltham, MA, USA), KRT7 (1:100; MilliporeSigma), ACTA2 (1:100; Lab Vision), NFM (1:100; Millipore, Burlington, MA, USA), NUMB (1:100; ab4147, abcam, Cambridge, England), PRDM14 (1:100; ab187881, abcam), BLIMP1 (1:100; ab198287, abcam), TFAP2C (1:2000; ab76007, abcam), NANOS3 (1:200; ab70001, abcam), SOX17 (1:100; Af1924, R&D Systems, Minneapolis, MN, USA), HLA-G (1:100; ab7758, abcam), ERVW-1 (1:250; ab71115, abcam), HCG (1:50; MAB4169, R&D Systems).

**Quantitative PCR (qPCR).**

The following primers were used: *GATA6* (Hs00232018m1), *SOX7* (Hs00846731s1), *MEF2C* (Hs00231149m1), *FOXC1* (Hs00559473s1), *NEUROD1* (Hs01922995s1), *MAP2* (Hs00258900m1), *KLF2* (Hs00360439g1), *KLF4* (Hs00358836m1), *OCT3/4* (Hs03005111g1), *NANOG* (Hs04399610g1), *SOX2* (Hs01053049s1), *TERT* (Hs00972565m1), *BLIMP1* (Hs00153357m1), *TFAP2C* (Hs00231476m1), *TBX3* (Hs00195612m1), *DPPA3* (Hs01931905g1), *ITGA6* (Hs01041008m1), *SYCP3* (Hs00538146m1), SSEA-1 (Hs01106466s1), *NANOS3* (Hs00928455s1), *SOX17* (Hs00751752s1), *DAZL* (Hs00154706m1), *CDX2* (Hs01078080m1), *GCM1* (Hs00961601m1), *TP63* (Hs00978343m1), *GATA2* (Hs00231119m1), *ID2* (Hs04187239m1), *ACTB* (Hs99999903m1), *Oct3/4* (Mm03053917g1), *Nanog* (Mm02384862g1), *Sox2* (Mm03053810s1), *Klf4* (Mm00516104m1), *Rex1* (Mm03053975g1), *Ssea-1* (Mm00487448s1), *Prdm14* (Mm01237814m1), *Blimp1* (Mm00476128m1), *Tfap2c* (Mm00493473m1), and *Actb* (Mm02619580g1).

**Transplantation into immune-deficient mice testes.**

hAM-Muse cells (1*10^5^ cells) were suspended in PBS and injected using glass micropipette into the testes of 8-week-old CB17/Icr-Prkdc scid/CrlCrlj (SCID) mice (n=3). Mice were sacrificed for analysis 4 months after injection. For negative control, testes were injected with PBS (n=3) and were sacrificed 4 months after injection. Tissues were fixed with 4% paraformaldehyde in 0.01M PBS and 3-µm-thick paraffin sections and analyzed by HE staining.

**Single-cell RNA-sequencing data processing.**

AM-Muse cells and BM-Muse cells were collected using FACS-AriaII (Becton Dickinson) as described above. Single-cell capture and cDNA synthesis were performed based on the TAS-seq protocol using Rhapsody Single-Cell Analysis System (Becton Dickinson) ^2^. All libraries were sequenced using Novaseq 6000 (Illumina, San Diego, CA, USA). Pair-end Fastq files of samples were processed as follows. Adapter trimming of sequencing data was performed using cutadapt 2.10 ^3^. Filtered reads were chunked into 16 parts for parallel processing using the Seqkit 0.9.0 ^4^. Filtered cell barcode reads were annotated based on a Python script provided by Becton Dickinson. Associated cDNA reads were mapped to reference RNA (build GRCh38 release-101) using bowtie2-2.4.2 by the following parameters: "-p 2 -D 20 -R 3 -N 0 -L 8 -i S,1,0.75 –norc –seed 656565 –reorder" ^5^. Then, cell barcode information of each read was added to the bowtie2-mapped BAM files and read counts of each gene in each cell barcode were counted using mawk. The resulting count data were converted to genes x cells matrix file, and the inflection threshold of the knee-plot was detected by DropletUtils package in R ver. 3.6.3 ^6,7^. In addition, we further estimated background beads using the DropletUtils "emptyDrops" function.

Demultiplexing procedures were processed as follows. For assignment of each tag to each cell barcode, read counts of each tag in each valid cell barcode, which is defined by the cDNA matrix, were extracted from the tag/cell barcode expression matrix. The sum of the total read counts of each tag was normalized to the minimum sum count of each tag, and log2 fold-change between the first-most tag counts and second-most tag counts within each cell barcode. The fold-change ascending order ranked the cell barcode of each cell, and the top 3.28% cells were identified as doublets, which were theoretically detectable doublets calculated by Poisson's distribution based on the number of loaded cells, total Rhapsody well number, and the number of tags used. Finally, the remaining cell barcodes were assigned to the first-most counted tags. The sequencing and sequencing data processing were performed by ImmunoGeneTeqs (Tokyo, Japan).

**Data analyses of single-cell RNA sequencing.**

The Seurat R package v3.2.2 in R ver. 4.0.2 was used for filtering, normalization, dimensionality reduction, differentially expressed gene (DEG) detection, and cell-cycle analysis ^8,9^. Cells outside thresholds of >5000 expressed genes and 1%–10% mitochondrial genes were considered low-quality cells and excluded. Genes that were detected in fewer than 3 cells were removed. The Seurat "SCtransform" function performed normalization, and the percent of mitochondrial genes was regressed out ^10^. Unsupervised clustering was performed by the Seurat "FindClusters" function with the parameter "dim = 30, resolution = 0.5". To visualize cell-to-cell relationships, principal component analysis dimensionality reduction was performed using the Seurat "RunPCA" function, and the top 30 principal components were used to generate a t-SNE plot using the Seurat "RuntSNE" function with the following parameter "dims = 30" ^11^. The Seurat "FindMarkers" function was used with the MAST algorithm to identify DEGs ^12^. Genes with a fold-change greater than 1.5 or smaller than 0.67 and p ≤ 0.05 were considered to be upregulated or downregulated, respectively (in male- and female-origin hAM-Muse cells, the upregulation and downregulation thresholds were fold-change greater than 1.25 or smaller than 0.80). The Database for Annotation, Visualization, and Integrated Discovery [DAVID: <http://david.abcc.ncifcrf.gov> (2021 Update)] was used for the GO analysis ^13^ on February 8^th^, 2022. All GO terms listed in bar chart were extracted from results "Functional Annotation Chart" function. The enrichment scores of each GO term were calculated in DAVID. The Seurat "CellCycleScoring" function was used to determine a cell cycle score for each cell according to G2/M and S phase marker gene expression ^14^. The cell cycle phase was decided on the basis of this score.

**Reference**

1 Kuroda, Y. *et al.* Isolation, culture and evaluation of multilineage-differentiating stress-enduring (Muse) cells. *Nat Protoc* **8**, 1391-1415, doi:10.1038/nprot.2013.076 (2013).

2 Chen, C. Y. *et al.* Combining an Alarmin HMGN1 Peptide with PD-L1 Blockade Results in Robust Antitumor Effects with a Concomitant Increase of Stem-Like/Progenitor Exhausted CD8(+) T Cells. *Cancer Immunol Res* **9**, 1214-1228, doi:10.1158/2326-6066.CIR-21-0265 (2021).

3 Martin, M. Cutadapt removes adapter sequences from high-throughput sequencing reads. *2011* **17**, 3, doi:10.14806/ej.17.1.200 (2011).

4 Shen, W., Le, S., Li, Y. & Hu, F. SeqKit: A Cross-Platform and Ultrafast Toolkit for FASTA/Q File Manipulation. *PLoS One* **11**, e0163962, doi:10.1371/journal.pone.0163962 (2016).

5 Langmead, B., Trapnell, C., Pop, M. & Salzberg, S. L. Ultrafast and memory-efficient alignment of short DNA sequences to the human genome. *Genome Biol* **10**, R25, doi:10.1186/gb-2009-10-3-r25 (2009).

6 Griffiths, J. A., Richard, A. C., Bach, K., Lun, A. T. L. & Marioni, J. C. Detection and removal of barcode swapping in single-cell RNA-seq data. *Nature Communications* **9**, 2667, doi:10.1038/s41467-018-05083-x (2018).

7 Lun, A. T. L. *et al.* EmptyDrops: distinguishing cells from empty droplets in droplet-based single-cell RNA sequencing data. *Genome Biology* **20**, 63, doi:10.1186/s13059-019-1662-y (2019).

8 Stuart, T. *et al.* Comprehensive Integration of Single-Cell Data. *Cell* **177**, 1888-+, doi:10.1016/j.cell.2019.05.031 (2019).

9 <R A Language and Enviroment for Statistical Computing.pdf>.

10 Hafemeister, C. & Satija, R. Normalization and variance stabilization of single-cell RNA-seq data using regularized negative binomial regression. *Genome Biol* **20**, 296, doi:10.1186/s13059-019-1874-1 (2019).

11 Jamieson, A. R. *et al.* Exploring nonlinear feature space dimension reduction and data representation in breast Cadx with Laplacian eigenmaps and t-SNE. *Med Phys* **37**, 339-351, doi:10.1118/1.3267037 (2010).

12 Finak, G. *et al.* MAST: a flexible statistical framework for assessing transcriptional changes and characterizing heterogeneity in single-cell RNA sequencing data. *Genome Biol* **16**, 278, doi:10.1186/s13059-015-0844-5 (2015).

13 Huang, D. W., Sherman, B. T. & Lempicki, R. A. Systematic and integrative analysis of large gene lists using DAVID bioinformatics resources. *Nature Protocols* **4**, 44-57, doi:10.1038/nprot.2008.211 (2009).

14 Tirosh, I. *et al.* Dissecting the multicellular ecosystem of metastatic melanoma by single-cell RNA-seq. *Science* **352**, 189-196, doi:10.1126/science.aad0501 (2016).

**
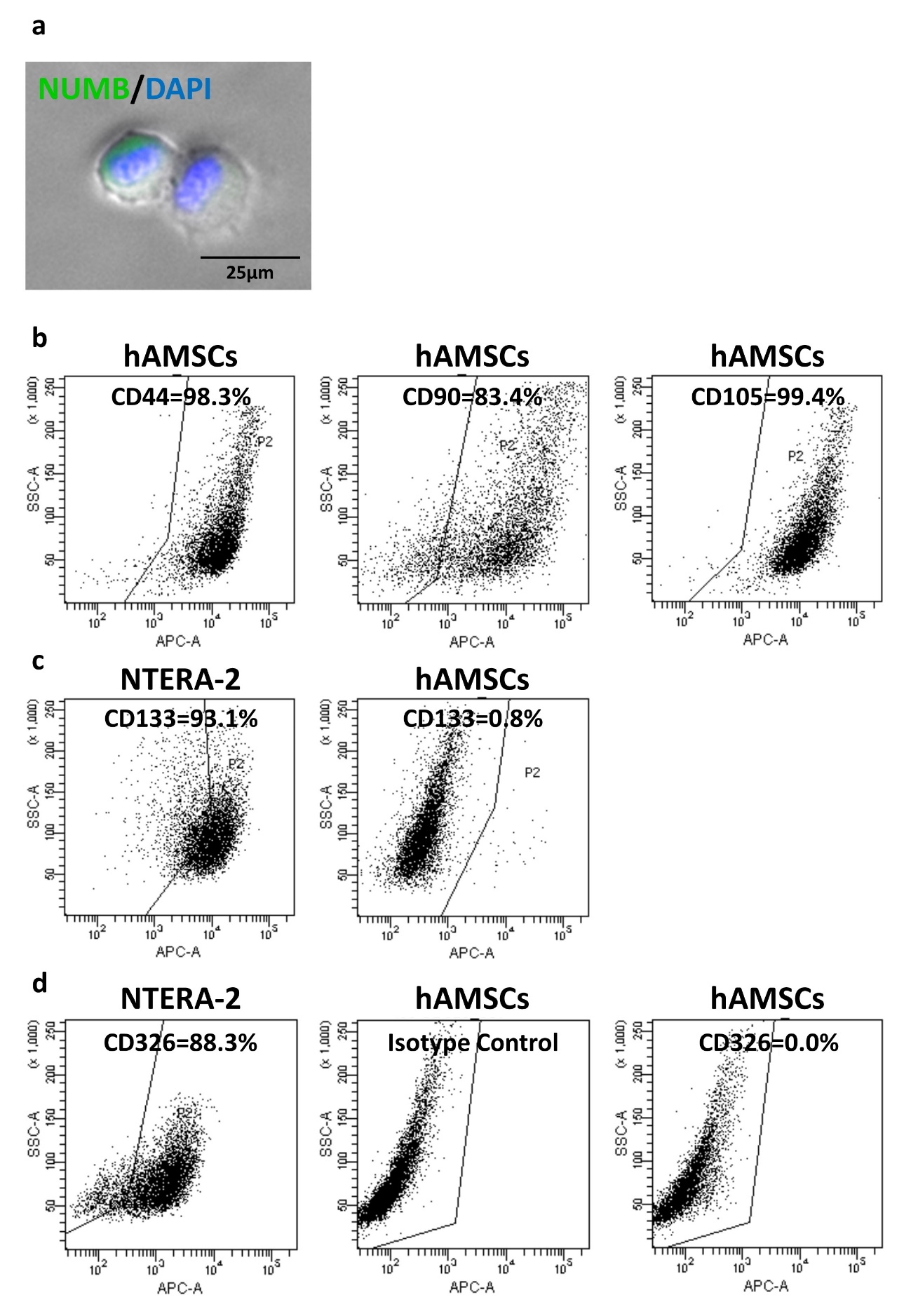
**

**Supplementary Figure 1.**

(a) Immunocytochemistry for NUMB (green signal) in hAMSC-SSEA-3(+) cells (bar = 25 µm).

(b) Expression of mesenchymal markers (CD44, CD90, and CD105) in hAMSCs.

(c) Expression of CD133 in NTERA-2 and hAMSCs.

(d) Expression of CD326 in NTERA-2 and hAMSCs.

**
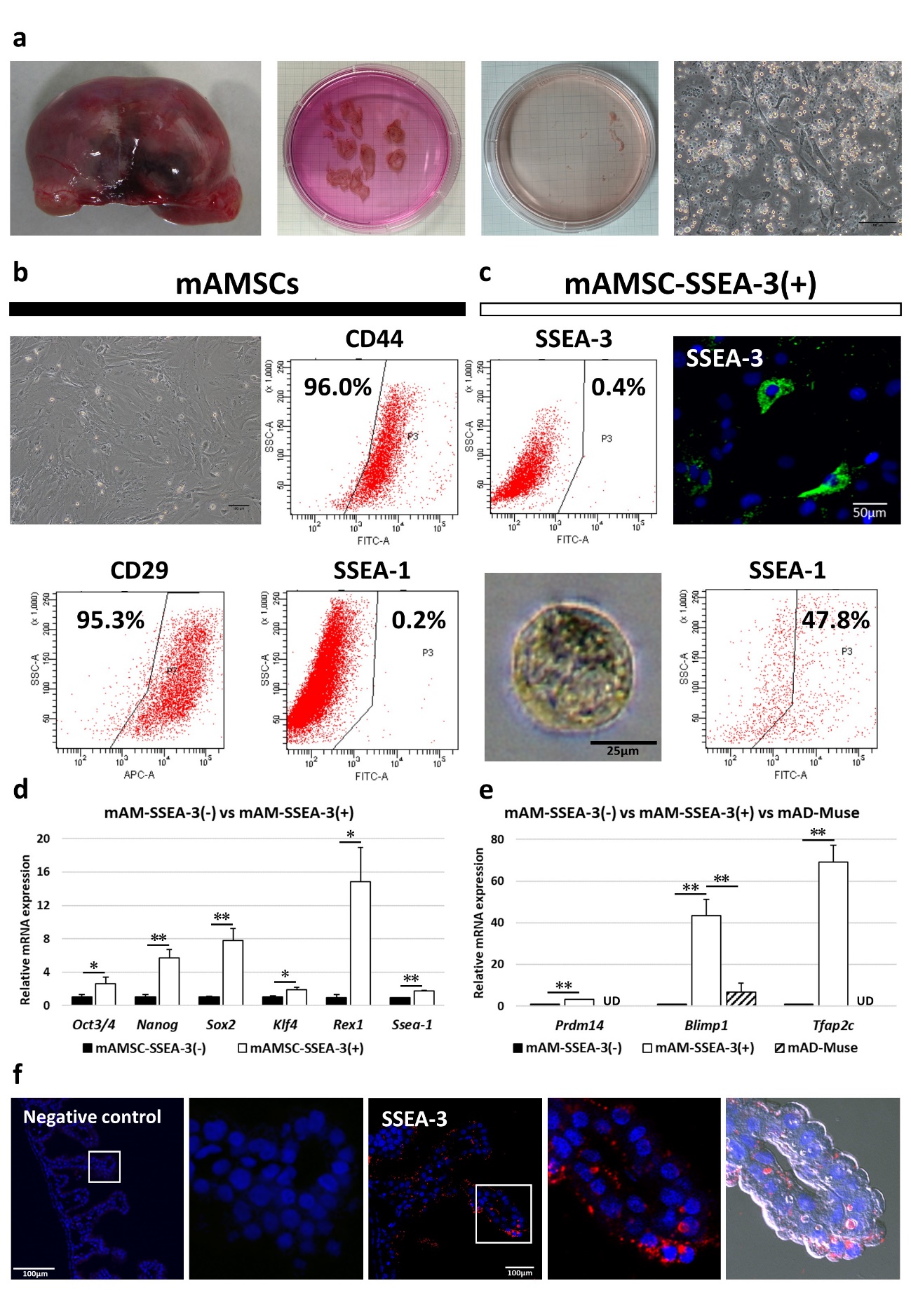
**

**Supplementary Figure 2.**

(a) The mAM cell isolation procedure. Embryos of ICR mouse, isolated amniotic tissues, plating amniotic tissue, and mAM-plastic-adherent cells (primary culture; bar = 100 µm).

(b) mAM plastic-adherent cells (passage 2). Expression of mesenchymal markers (CD44, CD29) and SSEA-1 in mAM plastic-adherent cells (bar = 100 µm).

(c) Example of SSEA-3(+) cells among mAMSCs. FACS analysis and immunocytochemistry showed the presence of mAMSC-SSEA-3(+) cells (bar = 50 µm). Clusters formed in single-cell suspension culture from mAMSC-SSEA-3(+) cells (bar = 25 µm). Expression of SSEA-1 in mAMSC-SSEA-3(+) cells.

(d) Expression of pluripotency-related genes in mAMSC-SSEA-3(-) and SSEA-3(+) (normalized by *ACTB*) cells. Values of mAMSC-SSEA-3(-) cells were set as 1. *p<0.05; **p<0.01.

(e) Expression of reproduction-related genes in mAMSC-SSEA-3(-), SSEA-3(+), and mAD-Muse cells (normalized by *ACTB*). Values of mAMSC-SSEA-3(-) cells were set as 1. *p<0.05; **p<0.01; UD=under detection limits.

(f) SSEA-3(+) cells were detected in the mouse AM by immunohistochemistry (bars = 100 µm).


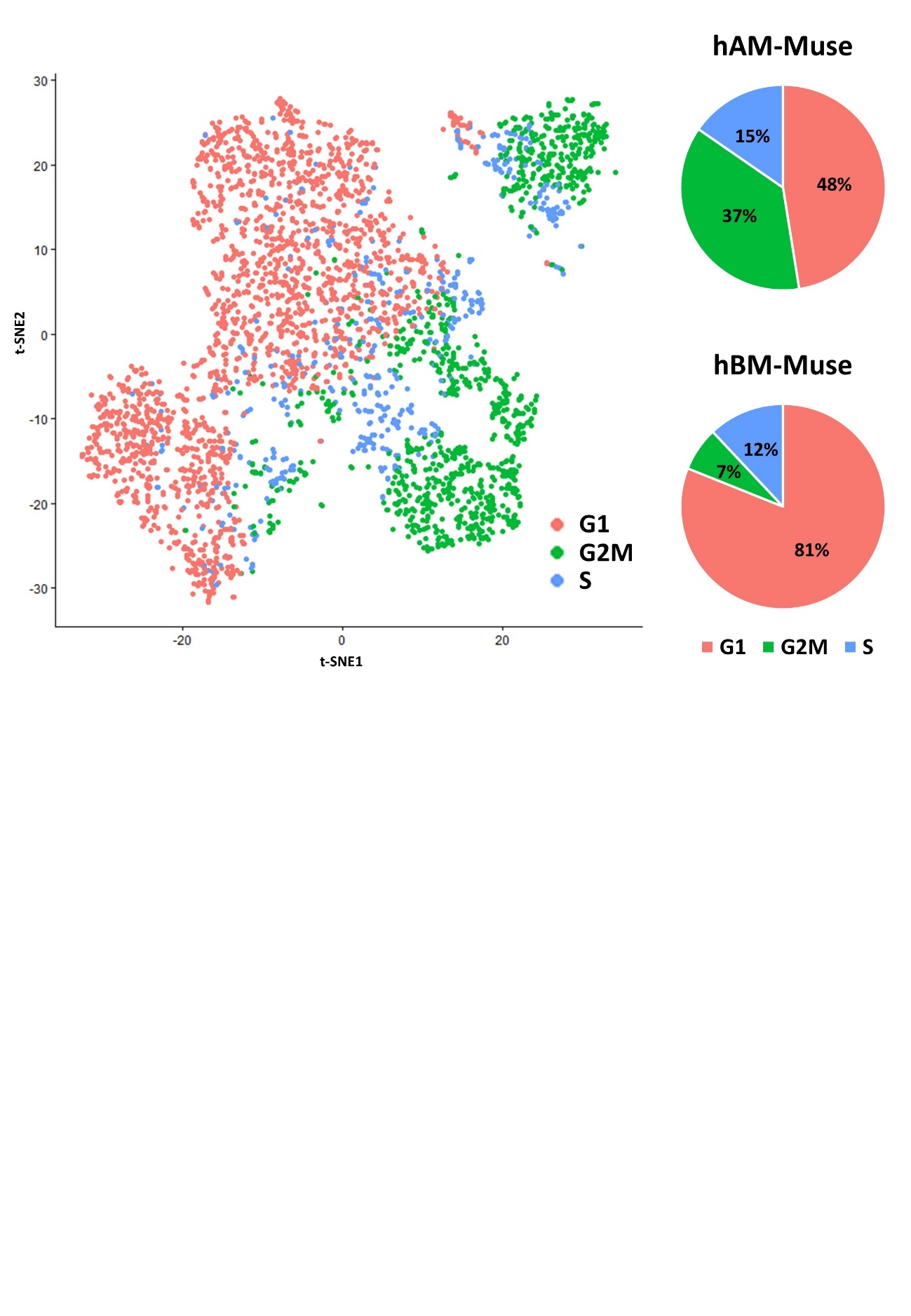
**Supplementary Figure 3.**

t-SNE plot of the cell cycle (G1, red; G2M, green; S, blue) in hAM- and hBM-Muse cells and the proportion of cells in each cell cycle.

**
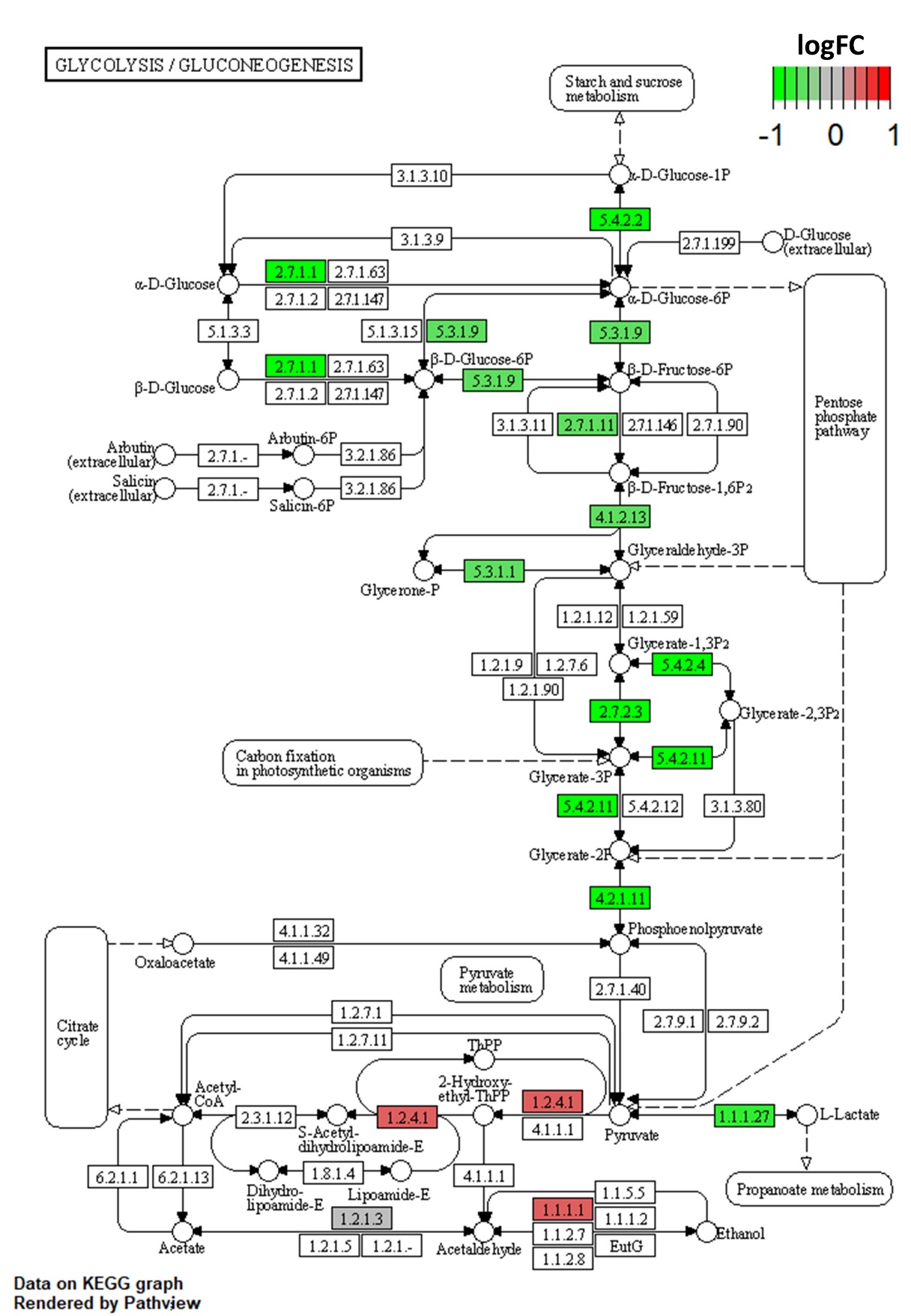
**

**Supplementary Figure 4.**

A portion of the KEGG pathway map of glycolysis/gluconeogenesis (map00010). Red and green colors indicate increased hAM- and hBM-Muse cell gene expression, respectively, as indicated by the colored scales.

**
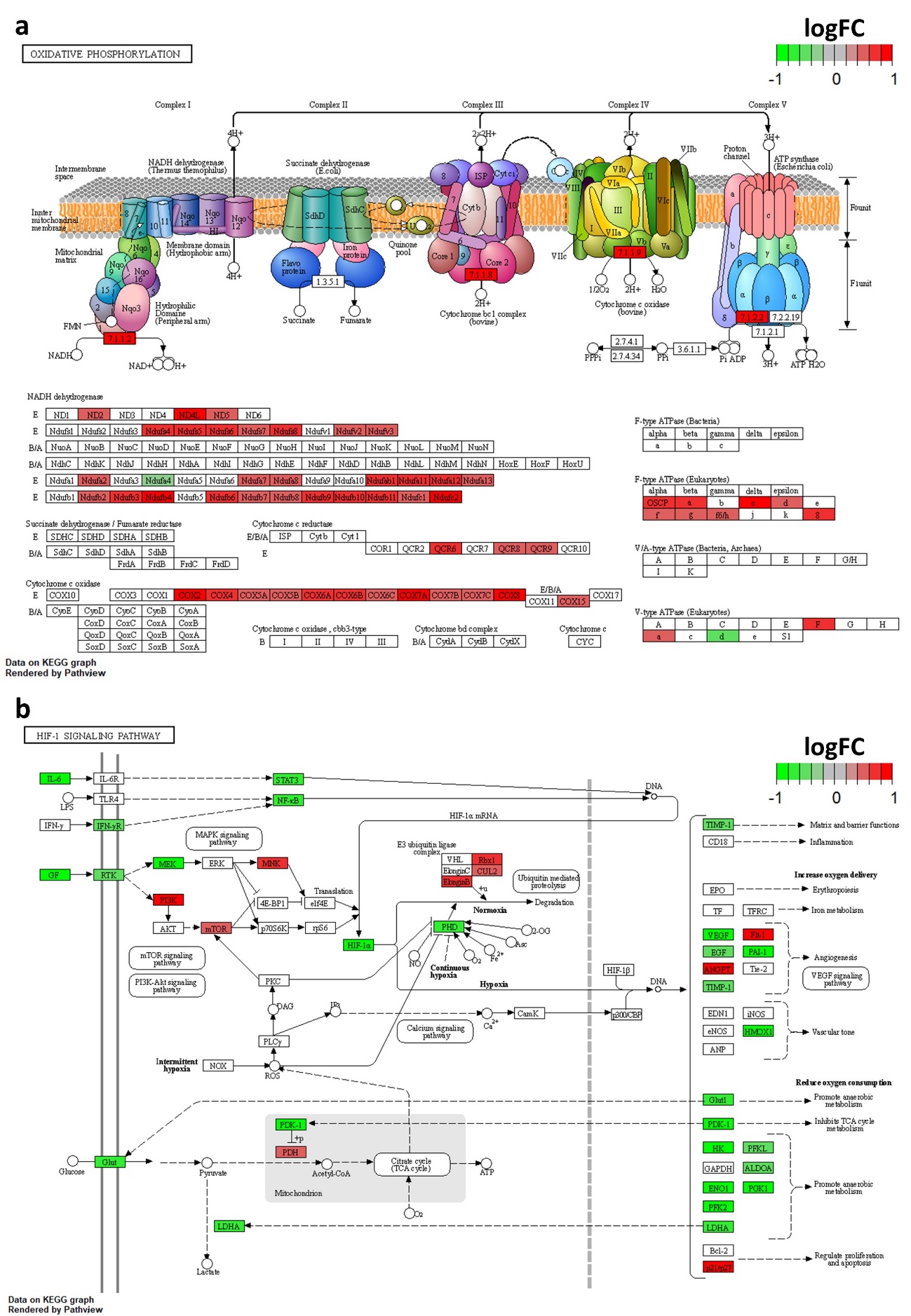
**

**Supplementary Figure 5.**

(a) A portion of the KEGG pathway map of oxidative phosphorylation (map00190). Red and green colors indicate increased hAM- and hBM-Muse cell gene expression, respectively, as indicated by the colored scales.

(b) A portion of the KEGG pathway map of HIF-1 signaling pathway (map04066). Red and green colors indicate increased hAM- and hBM-Muse cell gene expression, respectively, as indicated by the colored scales.


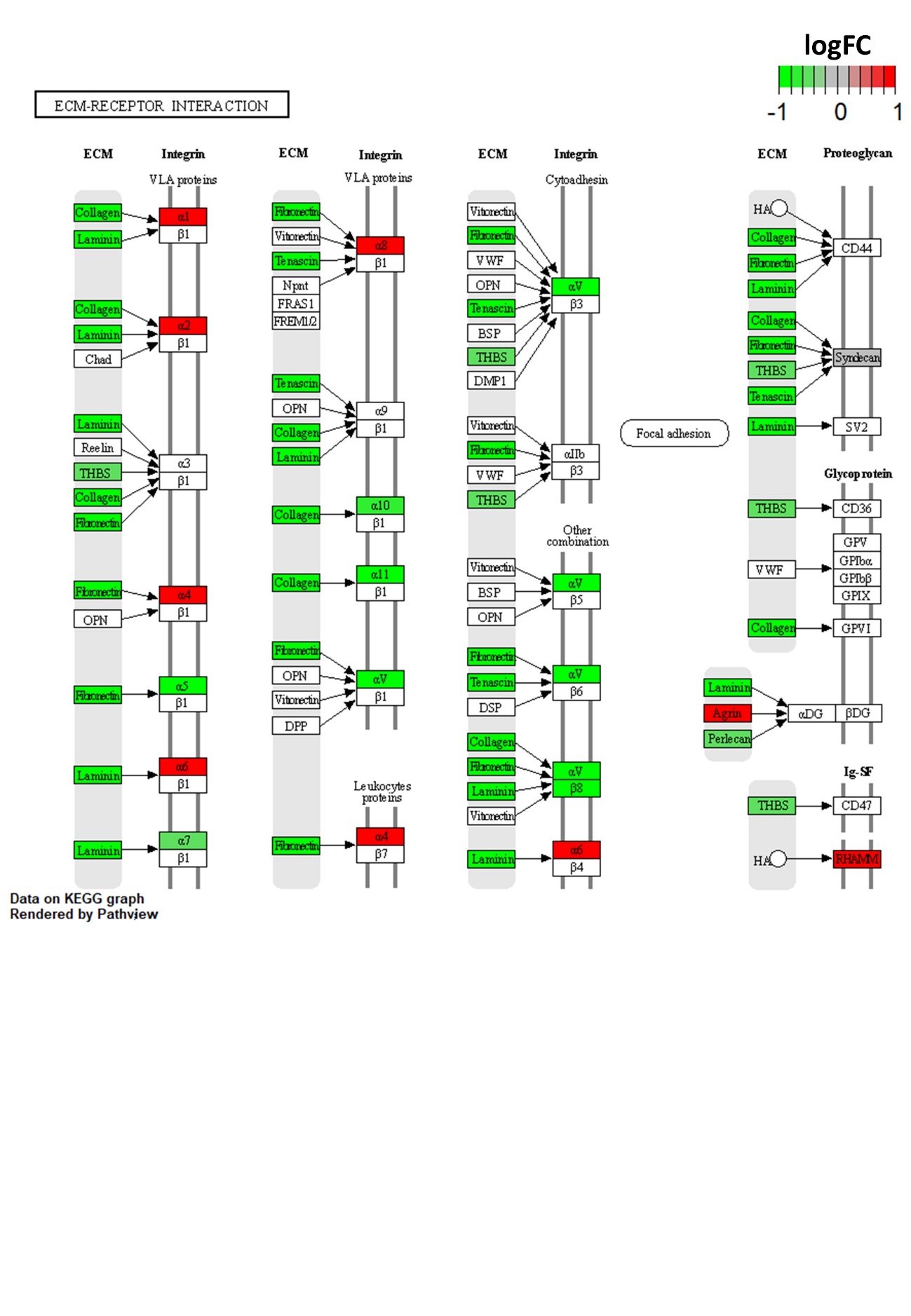


**Supplementary Figure 6.**

A portion of the KEGG pathway map of ECM-receptor interaction (map04512). Red and green colors indicate increased hAM- and hBM-Muse cell gene expression, respectively, as indicated by the colored scales.


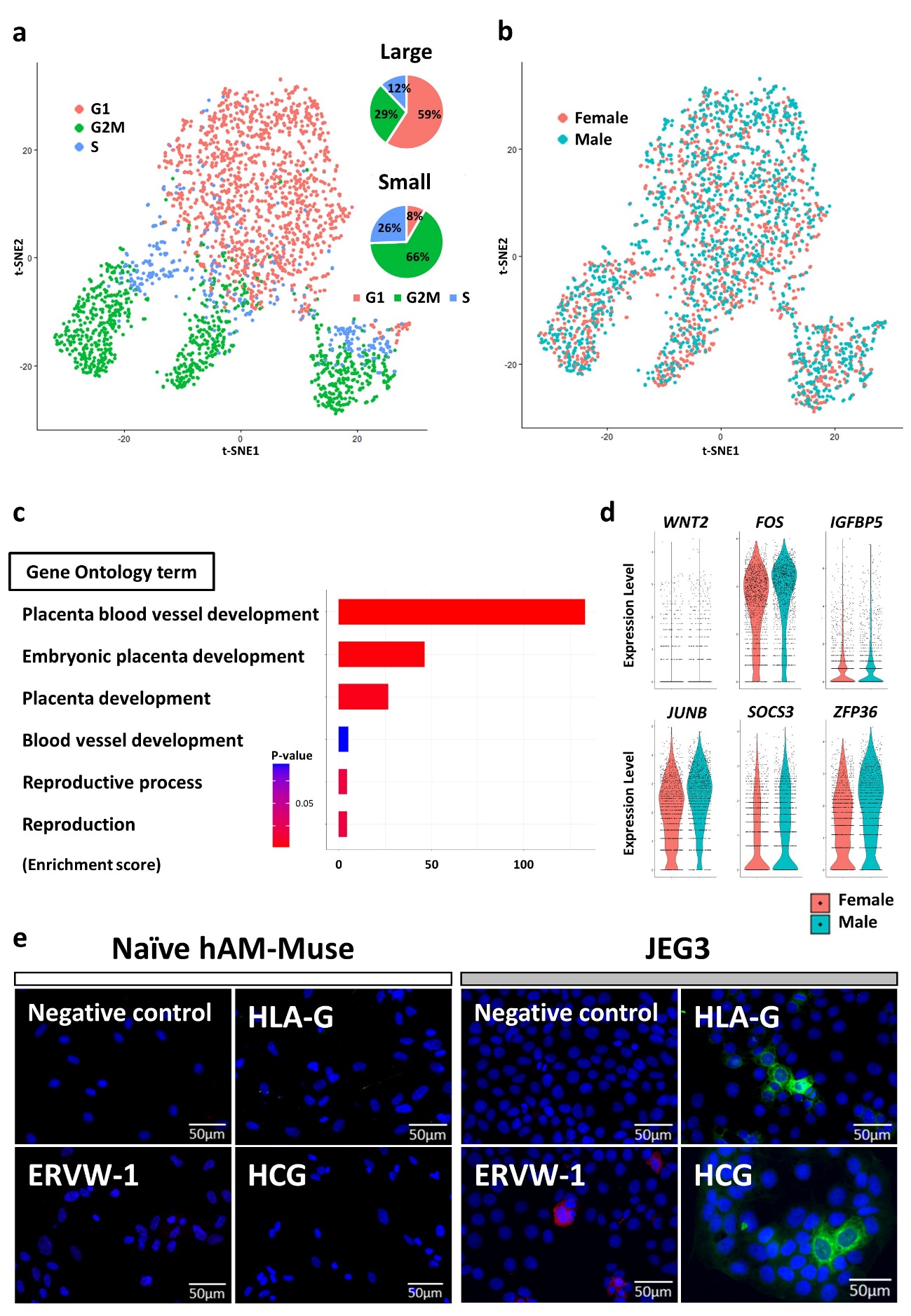


**Supplementary Figure 7.**

(a) t-SNE plot of the cell cycle (G1, red; G2M, green; S, blue) in large and small subpopulations and the proportion of cells in each cell cycle.

(b) t-SNE plot of female- (red) and male- (blue) origin hAM-Muse cells.

(c) Enriched GO terms associated with placentation in male-origin hAM-Muse cells. Bar chart shows the enrichment score in each term. Color scales indicate the adjusted p-values.

(d) Expression levels of genes related to placentation in female- (red) and male- (blue) origin hAM-Muse cells.

(e) Immunocytochemistry for HLA-G, ERVW-1 and HCG in naïve hAM-Muse cells and JEG3 (bar = 50 µm).
