# Supplementary material for: Naïve pluripotent-like characteristics of non-tumorigenic Muse cells isolated from human amniotic membrane
Source: Sci Rep. 2022 Oct 14;12:17222. doi: 10.1038/s41598-022-22282-1 (PMC9568515; doi:10.1038/s41598-022-22282-1)
Supplement: Supplementary file 2 — Supplementary Information 2. [file 41598_2022_22282_MOESM2_ESM.pdf]

Supplementary Table. 1

Upregulated genes in hAM-Muse cells than in hBM-Muse cells

GeneSymbol	p-value	avg_logFC	FC	pct.1	pct.2	p_val_adj
ALDH1A1	0	3.1556311	23.46784	0.722	0.041	2.21E-304
RARRES2	0	3.0928622	22.04007	0.838	0.126	0
HTR2B	0	3.0773964	21.70183	0.792	0.041	0
CHRM2	0	2.9894357	19.87446	0.822	0.124	0
ITGA6	0	2.7264142	15.27801	0.999	0.717	0
NR2F2	0	2.6896814	14.72698	0.978	0.785	0
SSTR1	0	2.6516789	14.17782	0.96	0.323	0
OXTR	0	2.5879408	13.30235	0.975	0.548	0
FOXL2	0.00E+00	2.4274522	11.32998	0.856	0.031	0.00E+00
IRAG1	1.8E-293	2.4144635	11.18377	0.725	0.065	5.58E-289
CLEC2B	0	2.3962139	10.98152	0.913	0.09	0
MASP1	0	2.3363274	10.34318	0.868	0.144	0
FILIP1L	0.00E+00	2.2507708	9.495052	0.948	0.47	0.00E+00
PCDH10	1.78E-199	2.2416986	9.4093	0.606	0.065	5.61E-195
NR2F1	5.51E-252	2.2315692	9.314471	0.824	0.289	1.74E-247
OLR1	4.2E-272	2.2135024	9.1477	0.649	0.024	1.31E-267
TRHDE	0	2.1349138	8.456317	0.85	0.197	0
MXRA5	0	2.1206978	8.336953	0.963	0.342	0
GATA2	0	2.1191623	8.324161	0.913	0.237	0
CITED2	0	2.0784097	7.99175	0.998	0.85	0
S100A4	0	2.0662695	7.895315	0.986	0.629	0
SPON2	0.00E+00	2.0424484	7.709462	0.967	0.405	0.00E+00
GULP1	2.40E-307	2.0362107	7.661523	0.872	0.418	7.55E-303
SYTL5	1.93E-225	2.0149563	7.5004	0.54	0.002	6.10E-221
IFIT3	4.42E-300	2.0040597	7.419115	0.829	0.174	1.40E-295
GLRX	6.05E-298	1.9850176	7.279175	0.975	0.948	1.91E-293
VAT1L	1.07E-287	1.9671554	7.150308	0.706	0.041	3.36E-283
GBP4	1.50E-217	1.9195336	6.817778	0.692	0.149	4.74E-213
TNFSF4	9.1E-172	1.901966	6.699052	0.549	0.055	2.86E-167
NES	0.00E+00	1.8973312	6.668075	0.939	0.284	0.00E+00
SYNPO2	1.3E-204	1.8901297	6.620227	0.824	0.385	3.98E-200
GREM1	0	1.8809919	6.560008	0.999	0.848	0
ITGA2	0.00E+00	1.8754039	6.523454	0.964	0.612	0.00E+00
HAND2	4.79E-283	1.8731731	6.508917	0.72	0.06	1.51E-278
MITF	5.45E-236	1.8611783	6.43131	0.728	0.137	1.72E-231
PTN	2.74E-287	1.8374466	6.280481	0.876	0.291	8.65E-283
ESAM	1.37E-216	1.8223832	6.186585	0.551	0.012	4.31E-212

MMP11	7.44E-64	1.8115531	6.119945	0.355	0.084	2.35E-59
ST6GALNAC5	7.51E-218	1.781038	5.936015	0.611	0.048	2.37E-213
REN	2.86E-81	1.7783849	5.920287	0.227	0	9.01E-77
FOXL2NB	3.34E-223	1.7775307	5.915232	0.607	0.042	1.05E-218
IGF2	2.29E-259	1.7740588	5.894731	0.872	0.513	7.22E-255
ARMC9	1.25E-236	1.7569895	5.794965	0.888	0.532	3.93E-232
OTULINL	7.57E-247	1.7566225	5.792839	0.612	0.019	2.39E-242
SCN9A	1E-191	1.739122	5.692343	0.725	0.215	3.21E-187
PAMR1	0.00E+00	1.7303815	5.642806	0.957	0.452	0.00E+00
SERPINB7	6.40E-261	1.7279162	5.628912	0.859	0.281	2.02E-256
HOXD11	1.63E-246	1.7268581	5.622959	0.716	0.098	5.14E-242
CPED1	8.92E-292	1.7219878	5.59564	0.897	0.323	2.81E-287
OGFRL1	7.25E-256	1.6780445	5.355074	0.952	0.662	2.29E-251
ITGA1	1.43E-199	1.6660072	5.291	0.955	0.734	4.50E-195
ADGRG6	4.60E-199	1.6651065	5.286236	0.703	0.156	1.45E-194
SEMA3A	3.52E-269	1.6512251	5.213363	0.867	0.299	1.11E-264
AR	4.90E-230	1.6503998	5.209062	0.628	0.046	1.55E-225
CLEC14A	9.48E-131	1.6492483	5.203067	0.426	0.029	2.99E-126
ACTG2	3.56E-31	1.6393334	5.151734	0.289	0.151	1.12E-26
MT-RNR1	0.00E+00	1.6331299	5.119874	1	1	0.00E+00
ERAP2	2.39E-206	1.631455	5.111306	0.702	0.157	7.55E-202
IFIT1	3.8E-196	1.630984	5.108899	0.617	0.071	1.18E-191
FZD2	0.00E+00	1.6243714	5.075228	0.944	0.45	0.00E+00
BTBD3	1.6E-249	1.6203114	5.054664	0.878	0.335	5.06E-245
CTSC	0.00E+00	1.6197885	5.052022	0.997	0.941	0.00E+00
HOXD10	1.89E-294	1.6190304	5.048193	0.767	0.092	5.96E-290
PCDH18	2.35E-239	1.5822173	4.865733	0.927	0.535	7.40E-235
ASPM	3.66E-77	1.5699969	4.806634	0.677	0.398	1.15E-72
PRDM1	1.05E-194	1.5639343	4.777581	0.719	0.168	3.31E-190
ADAM33	5.17E-285	1.5553322	4.73666	0.812	0.152	1.63E-280
MMP3	1.46E-162	1.5478623	4.701409	0.942	0.59	4.60E-158
HOXA11	1.06E-212	1.5352478	4.642476	0.861	0.363	3.36E-208
KIF20A	8.9E-105	1.5159275	4.553642	0.628	0.245	2.82E-100
ADM	0.00E+00	1.5141087	4.545368	0.981	0.627	0.00E+00
NUAK1	2.4E-198	1.5062119	4.509616	0.852	0.404	7.62E-194
FBLN5	1.76258217	1.500409	4.483522	0.949	0.463	5.56E-307
AC132217.2	6.51E-226	1.493082	4.450792	0.815	0.291	2.05E-221
TOR4A	7.58E-233	1.4857965	4.418483	0.69	0.089	2.39E-228
PSG4	1.37E-147	1.4755705	4.37353	0.702	0.233	4.33E-143
XAF1	4.9E-204	1.4672723	4.337388	0.794	0.276	1.54E-199

MYLK	2.25407515	1.4664674	4.333898	0.999	0.966	7.10957843865577e-3
F3	3.08E-158	1.4647942	4.326653	0.793	0.351	9.73E-154
DKK1	4.33E-173	1.4640387	4.323385	0.974	0.866	1.37E-168
DLGAP5	2.48E-88	1.4551658	4.285194	0.599	0.27	7.84E-84
SGIP1	9.60E-208	1.4518676	4.271084	0.683	0.106	3.03E-203
PLAT	4.01E-177	1.4513128	4.268715	0.946	0.668	1.27E-172
ACTA2	5.99E-200	1.4436704	4.236216	0.997	0.917	1.89E-195
FOXF1	1.99E-185	1.4412591	4.226013	0.556	0.042	6.26E-181
MKX	1.12E-124	1.4385966	4.214777	0.451	0.049	3.55E-120
NCAM1	8.93E-138	1.4246877	4.15656	0.501	0.065	2.82E-133
FOS	8.22E-269	1.4241219	4.154208	0.936	0.579	2.59E-264
PTTG1	1.13E-131	1.4224831	4.147406	0.85	0.635	3.57E-127
ISG15	7.50E-231	1.4042236	4.072364	0.838	0.339	2.37E-226
CENPF	1.13E-66	1.402617	4.065826	0.724	0.536	3.56E-62
AK5	6.55E-259	1.40137	4.060759	0.928	0.47	2.06E-254
STAMBPL1	1.04E-186	1.3887216	4.009721	0.746	0.217	3.27E-182
PCDH7	3.72E-72	1.385653	3.997435	0.312	0.047	1.17E-67
TCIM	1.64E-132	1.3854877	3.996775	0.366	0.004	5.18E-128
RGCC	1.21E-58	1.3792854	3.972062	0.494	0.206	3.81E-54
SHROOM3	1.26E-131	1.3750963	3.955458	0.631	0.182	3.96E-127
PDE5A	2.57E-151	1.3736992	3.949935	0.766	0.353	8.09E-147
CENPE	1.63E-55	1.3658623	3.919101	0.588	0.369	5.14E-51
EMX2	1.3E-235	1.3605343	3.898276	0.707	0.095	4.05E-231
GNG11	0.00E+00	1.3584891	3.890311	0.998	0.909	0.00E+00
KIF20B	1.85E-68	1.3545629	3.875067	0.617	0.343	5.85E-64
CDC20	3.86E-68	1.3520542	3.865357	0.639	0.411	1.22E-63
IGFBP5	8.24E-07	1.345923	3.841731	0.473	0.376	2.60E-02
SYNGR1	4.82E-162	1.3376843	3.81021	0.671	0.182	1.52E-157
IER5L	2.43E-250	1.3328986	3.792019	0.793	0.174	7.68E-246
HMGB2	1.01E-78	1.3308845	3.784389	0.862	0.651	3.19E-74
GNG4	3.66E-157	1.3299579	3.780884	0.549	0.088	1.16E-152
FAM43A	7.31E-103	1.3291236	3.777731	0.577	0.2	2.31E-98
LMOD1	2.77E-129	1.3242486	3.75936	0.635	0.186	8.75E-125
PRICKLE1	5.78E-146	1.3211729	3.747814	0.643	0.171	1.82E-141
PRKAR2B	9E-155	1.3198428	3.742833	0.57	0.089	2.83E-150
STMN1	0.00E+00	1.3167162	3.731149	0.999	0.92	0.00E+00
CHN1	1.11E-155	1.3150522	3.724945	0.742	0.259	3.49E-151
QPRT	1.24E-107	1.3136234	3.719627	0.555	0.183	3.93E-103
NFKBIZ	2.94E-175	1.313161	3.717907	0.876	0.5	9.26E-171
UBE2C	3.92E-34	1.3114778	3.711655	0.63	0.486	1.24E-29

FLT1	5.65E-135	1.3111059	3.710275	0.624	0.193	1.78E-130
GAS2L3	1.84E-73	1.3060164	3.691439	0.535	0.225	5.82E-69
LINC02844	4.21E-136	1.301787	3.675859	0.369	0.002	1.33E-131
ARHGEF17	4.11E-197	1.2987064	3.664553	0.866	0.414	1.30E-192
CSRP2	5.65E-181	1.2972083	3.659068	0.921	0.628	1.78E-176
TTK	4.73E-57	1.2950907	3.651327	0.45	0.234	1.49E-52
KHDRBS3	1.2E-175	1.2904441	3.6344	0.677	0.145	3.73E-171
C12orf75	0.00E+00	1.2902375	3.63365	1	0.999	0.00E+00
AURKA	8.95E-65	1.2849855	3.614616	0.552	0.267	2.82E-60
SLC7A11	8.53E-209	1.284138	3.611553	0.967	0.857	2.69E-204
RHOBTB3	1.26E-216	1.2811829	3.600897	0.986	0.992	3.99E-212
CTSK	5.79E-186	1.2810791	3.600523	0.868	0.415	1.83E-181
KRT19	3.84E-107	1.2803988	3.598074	0.812	0.532	1.21E-102
CKAP2	1.23E-99	1.2787423	3.592119	0.73	0.369	3.88E-95
CARD16	1.13E-185	1.2775288	3.587763	0.539	0.035	3.56E-181
GBP2	9.5E-146	1.2764692	3.583963	0.704	0.234	3E-141
KANK2	0.00E+00	1.2726805	3.57041	0.998	0.926	0.00E+00
NDC80	1.01E-71	1.2677441	3.552829	0.58	0.273	3.17E-67
MME	4.12E-150	1.2663775	3.547977	0.779	0.329	1.30E-145
NID1	1.20E-303	1.2663427	3.547853	0.998	0.921	3.80E-299
A2M	1.23E-73	1.2634158	3.537484	0.239	0.007	3.89E-69
CKS1B	2.74E-112	1.2619807	3.532411	0.851	0.679	8.63E-108
TOP2A	9.41E-44	1.2614309	3.53047	0.773	0.652	2.97E-39
HOXA11-AS	3.56E-152	1.2563466	3.512565	0.555	0.082	1.12E-147
MGARP	4.63E-116	1.25391	3.504017	0.532	0.118	1.46E-111
LRRC17	1.82E-107	1.2508797	3.493415	0.656	0.296	5.73E-103
PBX1	3.69E-188	1.2490367	3.486982	0.866	0.417	1.17E-183
HOXA10	1.14E-173	1.248473	3.485017	0.924	0.604	3.60E-169
GPNMB	7.95E-134	1.2477359	3.482449	0.619	0.161	2.51E-129
TRBC2	2.4E-113	1.2454134	3.474371	0.463	0.073	7.43E-109
FARP1	0.00E+00	1.2445412	3.471342	0.997	0.91	0.00E+00
TRNP1	3.19E-130	1.2399051	3.455286	0.77	0.384	1.01E-125
CCDC14	6.75E-161	1.2369362	3.445042	0.821	0.388	2.13E-156
ITGA4	7.00E-306	1.2362254	3.442594	0.989	0.813	2.21E-301
ID1	2.57E-281	1.2241041	3.401118	0.985	0.749	8.11E-277
CDKN3	9.50E-81	1.221751	3.393124	0.659	0.398	3.00E-76
HSBP1L1	1.90E-169	1.2209977	3.390569	0.735	0.251	6.00E-165
IFIT2	1.02E-94	1.2183799	3.381704	0.369	0.042	3.21E-90
TRHDE-AS1	4.02E-128	1.2167477	3.376189	0.428	0.035	1.27E-123
FRY	8.01E-96	1.2158652	3.373211	0.421	0.072	2.53E-91

CEP55	1.33E-73	1.2155209	3.37205	0.629	0.355	4.20E-69
PHTF2	4.70E-263	1.2151057	3.37065	0.972	0.711	1.48E-258
NUSAP1	2.04E-74	1.2107852	3.356119	0.646	0.375	6.43E-70
RBP1	7.40E-71	1.2096529	3.352321	0.487	0.2	2.33E-66
RBM24	3.00E-157	1.207212	3.344148	0.831	0.38	9.45E-153
CCNA2	7.72E-57	1.2051953	3.337411	0.639	0.433	2.44E-52
DIO2	3.01E-34	1.2037909	3.332727	0.264	0.078	9.48E-30
AFF3	7.29E-92	1.1984579	3.315001	0.47	0.125	2.30E-87
MTCL1	1.27E-137	1.1905726	3.288964	0.688	0.227	4.02E-133
TGM2	1.27E-140	1.1891878	3.284413	0.964	0.926	4.01E-136
DEPDC1	3.39E-61	1.1875036	3.278886	0.573	0.313	1.07E-56
PPME1	1.42E-61	1.1869383	3.277032	0.937	0.936	4.49E-57
RARB	4.32E-115	1.1859398	3.273762	0.36	0.014	1.36E-110
KIF23	2.16E-72	1.18387	3.266993	0.715	0.44	6.81E-68
PRDX6	0.00E+00	1.1832807	3.265068	1	1	0.00E+00
UBALD2	3.96E-140	1.1804941	3.255983	0.811	0.421	1.25E-135
CCNB1	5.41E-64	1.1785736	3.249735	0.767	0.548	1.71E-59
ZNF516	3.83E-149	1.178508	3.249522	0.666	0.174	1.21E-144
TPX2	2.13E-72	1.1771588	3.245141	0.78	0.556	6.71E-68
TMSB4X	0.00E+00	1.1762849	3.242306	1	1	0.00E+00
HSPB6	1.18E-212	1.1750269	3.23823	0.979	0.842	3.73E-208
BUB1	1.06E-59	1.1701017	3.22232	0.515	0.251	3.34E-55
ZNF804A	6.52E-103	1.1688235	3.218204	0.366	0.032	2.06E-98
HAND2-AS1	3.59E-117	1.166877	3.211946	0.578	0.213	1.13E-112
PODXL	1.64E-66	1.1659823	3.209074	0.624	0.318	5.16E-62
PSG5	7.14E-83	1.1627206	3.198624	0.793	0.486	2.25E-78
GSDME	1.23E-157	1.1493491	3.156138	0.913	0.649	3.89E-153
SBF2-AS1	6.10E-78	1.1487622	3.154286	0.505	0.189	1.92E-73
KNL1	2.85E-60	1.1487541	3.15426	0.512	0.27	8.98E-56
CDCA8	2.04E-48	1.145485	3.143966	0.428	0.21	6.43E-44
PSRC1	1.00E-72	1.1434245	3.137494	0.46	0.14	3.16E-68
MKI67	1.67E-56	1.1394247	3.12497	0.659	0.416	5.28E-52
MFAP4	3.31E-72	1.1381793	3.121081	0.576	0.253	1.04E-67
PLK1	2.96E-55	1.1380145	3.120566	0.561	0.339	9.34E-51
KIF2C	5.26E-52	1.1343087	3.109023	0.461	0.233	1.66E-47
GRIK2	6.04E-115	1.1335217	3.106578	0.611	0.188	1.91E-110
PIMREG	1.50E-88	1.1214994	3.069453	0.59	0.23	4.75E-84
MIR503HG	1.98E-135	1.1212459	3.068675	0.659	0.187	6.26E-131
SGO2	1.92E-55	1.1199154	3.064595	0.54	0.305	6.05E-51
IRS1	7.62E-165	1.1186389	3.060686	0.928	0.688	2.40E-160

GALNT6	9E-122	1.1129053	3.043187	0.754	0.366	2.83E-117
DKK3	0.00E+00	1.1118646	3.040022	1	0.974	0.00E+00
UACA	3.99E-267	1.1107521	3.036641	0.998	0.989	1.26E-262
TACC3	6.85E-64	1.1085403	3.029932	0.621	0.369	2.16E-59
HMMR	6.83E-52	1.1048242	3.018694	0.534	0.329	2.15E-47
PLEKHA2	1.42E-201	1.1029836	3.013143	0.973	0.793	4.49E-197
KANK1	2.5E-129	1.0985915	2.999938	0.756	0.362	7.95E-125
SLC38A2	0.00E+00	1.0982774	2.998996	1	0.971	0.00E+00
AP1S2	3.70E-220	1.0966473	2.994111	0.982	0.795	1.17E-215
MAP3K5	2.94E-127	1.0958241	2.991647	0.592	0.143	9.26E-123
ANO4	1.51E-128	1.0948172	2.988636	0.38	0.011	4.76E-124
AURKB	8.84E-48	1.0941235	2.986564	0.455	0.24	2.79E-43
TRERF1	4.04E-123	1.0931959	2.983795	0.638	0.193	1.27E-118
CDKN1B	1.50E-123	1.0917421	2.97946	0.802	0.408	4.72E-119
CENPA	1.41E-50	1.091706	2.979352	0.406	0.152	4.45E-46
CDCA3	8.45E-65	1.0909083	2.976977	0.571	0.283	2.66E-60
HACD4	9.78E-146	1.0897214	2.973445	0.776	0.331	3.09E-141
MIS18BP1	8.81E-112	1.086586	2.964137	0.751	0.371	2.78E-107
TMEM97	2.03E-95	1.0852024	2.960039	0.654	0.291	6.39E-91
HOXD9	9.38E-115	1.0848486	2.958992	0.492	0.094	2.96E-110
CKAP2L	9.39E-47	1.0839201	2.956246	0.5	0.33	2.96E-42
CCDC71L	2.25E-114	1.0831148	2.953866	0.934	0.751	7.09E-110
BHMT2	4.60E-100	1.0803234	2.945632	0.555	0.185	1.45E-95
PLBD1	2.10E-91	1.0782774	2.939612	0.371	0.046	6.63E-87
ECM1	2.00E-267	1.0756442	2.931881	0.993	0.809	6.30E-263
KIF14	2.54E-40	1.0735283	2.925684	0.435	0.24	8.02E-36
DRAM1	1.63E-190	1.0733167	2.925065	0.963	0.758	5.14E-186
NCAPG	1.70E-55	1.0716208	2.920108	0.567	0.344	5.37E-51
AGRN	2.11E-149	1.0709272	2.918084	0.839	0.46	6.66E-145
SYPL2	3.5E-113	1.0698292	2.914882	0.361	0.017	1.09E-108
CUTA	0.00E+00	1.0677771	2.908906	1	0.996	0.00E+00
ARHGEF2	1.10E-159	1.0649446	2.900678	0.884	0.486	3.47E-155
ARHGAP18	1.71E-194	1.0609319	2.889062	0.957	0.668	5.39E-190
RACGAP1	8.67E-64	1.0603778	2.887462	0.634	0.36	2.74E-59
UBE2T	2.71E-73	1.0599342	2.886181	0.673	0.393	8.53E-69
NEXN	6.34E-189	1.0558007	2.874276	0.975	0.89	2.00E-184
KRT18	7.04E-145	1.0551197	2.872319	0.936	0.831	2.22E-140
AC132217.1	5.11E-170	1.0543835	2.870205	0.706	0.194	1.61E-165
TWIST2	1.86E-186	1.0532331	2.866905	0.91	0.501	5.86E-182
CAVIN2	1.69E-119	1.0527397	2.865491	0.449	0.052	5.34E-115

PLPP2	2.07E-80	1.051935	2.863186	0.471	0.157	6.53E-76
PYCARD	4.74E-119	1.0506397	2.85948	0.748	0.381	1.49E-114
PHF19	6.67E-85	1.0490302	2.854881	0.725	0.433	2.10E-80
CENPU	3.27E-63	1.0476669	2.850992	0.549	0.276	1.03E-58
CENPW	2.69E-109	1.047087	2.849339	0.682	0.337	8.48E-105
LRCH2	1.43E-101	1.0417315	2.83412	0.414	0.055	4.52E-97
JPH2	6.54E-113	1.0417029	2.834039	0.531	0.124	2.06E-108
SPDL1	2.11E-84	1.0409953	2.832034	0.839	0.643	6.66E-80
CCNB2	1.12E-65	1.039768	2.828561	0.602	0.321	3.53E-61
FAM83D	8.63E-42	1.03966	2.828255	0.43	0.218	2.72E-37
PGR	6.59E-115	1.0396077	2.828107	0.308	0	2.08E-110
TSPAN14	3.44E-145	1.0386905	2.825515	0.913	0.658	1.08E-140
GTSE1	3.23E-51	1.0384019	2.824699	0.53	0.312	1.02E-46
TNFRSF19	1.18E-132	1.0375328	2.822245	0.865	0.506	3.73E-128
TEAD3	6.36E-133	1.0354426	2.816353	0.804	0.393	2.01E-128
CLDN11	1.04E-103	1.0325785	2.808298	0.879	0.68	3.29E-99
PRC1	7.56E-71	1.0317101	2.80586	0.754	0.546	2.38E-66
NDUFC2-KCT	0.00E+00	1.0298951	2.800772	0.989	0.819	0.00E+00
GFRA1	4.81E-51	1.025327	2.788007	0.364	0.12	1.52E-46
ZFP36L2	1.00E-188	1.0225987	2.780411	0.956	0.673	3.17E-184
CKS2	6.98E-38	1.0223986	2.779855	0.878	0.802	2.20E-33
NUF2	3.83E-45	1.022277	2.779516	0.474	0.266	1.21E-40
CCDC85A	1.00E-124	1.0217478	2.778046	0.484	0.066	3.17E-120
AMIGO2	1.43E-90	1.0216767	2.777849	0.886	0.719	4.52E-86
CHAC1	4.52E-70	1.0214991	2.777355	0.462	0.147	1.43E-65
CDK15	1.36E-88	1.0192821	2.771205	0.62	0.255	4.29E-84
KIF18A	1.63E-56	1.0154015	2.760471	0.5	0.233	5.14E-52
PTPRD	3.4E-97	1.014908	2.75911	0.319	0.014	1.07E-92
NDUFC2	0.00E+00	1.0141708	2.757076	1	0.995	0.00E+00
BIRC5	4.20E-83	1.01226	2.751813	0.822	0.61	1.33E-78
CAMK1G	4.45E-94	1.0118348	2.750643	0.349	0.03	1.40E-89
SEMA3D	4.08E-87	1.0102984	2.746421	0.359	0.058	1.29E-82
RFLNB	6.85E-214	1.0101685	2.746064	0.984	0.85	2.16E-209
SEMA6D	7.55E-65	1.0098935	2.745309	0.284	0.038	2.38E-60
LINC02762	4.68E-98	1.0084086	2.741235	0.562	0.191	1.48E-93
IRS2	3.43E-101	1.0064325	2.735823	0.782	0.45	1.08E-96
ANLN	6.30E-64	1.0060456	2.734765	0.77	0.572	1.99E-59
ZADH2	1.01E-132	1.0059794	2.734584	0.825	0.428	3.20E-128
FENDRR	9.13E-81	1.0049922	2.731886	0.334	0.046	2.88E-76
TNS3	7.99E-132	1.0021483	2.724128	0.905	0.659	2.52E-127

PIK3R1	4.60E-127	0.9997672	2.717649	0.813	0.426	1.45E-122
LACTB2	3.69E-114	0.9987916	2.714999	0.815	0.495	1.16E-109
SMAD9	4.34E-102	0.9977089	2.712061	0.658	0.263	1.37E-97
MEIS1	5.95E-113	0.9975587	2.711654	0.749	0.355	1.88E-108
CAMK2N1	5.46E-113	0.9937801	2.701427	0.765	0.394	1.72E-108
SEMA5A	3.12E-121	0.9936697	2.701129	0.892	0.596	9.84E-117
SAMD12	7.35E-76	0.9916543	2.69569	0.491	0.186	2.32E-71
RGS10	1.19E-141	0.986058	2.680646	0.91	0.692	3.75E-137
BDKRB2	3.02E-76	0.9856973	2.67968	0.352	0.058	9.51E-72
LACTB	1.73E-131	0.9851174	2.678126	0.931	0.723	5.46E-127
NLGN1	1E-95	0.9842264	2.675741	0.474	0.1	3.16E-91
CSTB	0.00E+00	0.9830475	2.672589	0.997	0.953	0.00E+00
PITX1	1.29E-40	0.9827353	2.671754	0.406	0.205	4.05E-36
PBK	2.23E-53	0.9822079	2.670346	0.581	0.326	7.03E-49
SHCBP1	2.27E-68	0.9818125	2.66929	0.673	0.409	7.15E-64
TOX	2.82E-89	0.9815797	2.668669	0.447	0.094	8.90E-85
HMGB3	6.45E-95	0.9813718	2.668114	0.81	0.522	2.04E-90
ARHGDIB	7.76E-75	0.9804199	2.665575	0.474	0.145	2.45E-70
SOCS5	3.52E-169	0.9798426	2.664037	0.95	0.661	1.11E-164
SMC2	1.04E-95	0.9780807	2.659347	0.847	0.583	3.29E-91
TROAP	4.54E-64	0.9779403	2.658974	0.514	0.225	1.43E-59
SKA3	1.11E-57	0.9758462	2.653412	0.522	0.296	3.51E-53
OSR2	3.72E-95	0.9750081	2.651189	0.456	0.089	1.17E-90
DYNLT3	2.80E-172	0.9748277	2.65071	0.994	0.974	8.84E-168
ISYNA1	8.34E-87	0.9733294	2.646742	0.584	0.223	2.63E-82
MT-ND4L	0	0.9727978	2.645335	1	1	0
TXN	0	0.9725826	2.644766	1	1	0
MYL6	0.00E+00	0.9715119	2.641936	1	1	0.00E+00
MGLL	1.65E-237	0.9713812	2.64159	0.995	0.898	5.19E-233
HERC2P2	4.42E-89	0.9710483	2.640711	0.572	0.211	1.39E-84
KIFC1	1.57E-49	0.9704066	2.639017	0.518	0.332	4.96E-45
DDIT3	1.86E-96	0.969321	2.636154	0.591	0.21	5.87E-92
SERTAD4	3.63E-66	0.9683066	2.633481	0.346	0.07	1.14E-61
ADRA1D	2.58E-77	0.9682203	2.633254	0.335	0.047	8.15E-73
COL13A1	3.18E-124	0.9669413	2.629888	0.689	0.251	1.00E-119
LPAR1	8.54E-146	0.9650384	2.624889	0.909	0.596	2.69E-141
CCNF	7.35E-43	0.9644182	2.623261	0.382	0.176	2.32E-38
KLF5	5.53E-78	0.9638408	2.621747	0.467	0.126	1.74E-73
FJX1	4.09E-98	0.9638166	2.621683	0.873	0.585	1.29E-93
KIF11	3.57E-42	0.963768	2.621556	0.541	0.336	1.13E-37

AASS	1.70E-100	0.9636981	2.621373	0.774	0.459	5.36E-96
MLLT3	1.44E-102	0.9630328	2.619629	0.764	0.441	4.53E-98
ABLIM3	6.70E-120	0.962523	2.618294	0.759	0.332	2.11E-115
ARHGAP11A	2.14E-54	0.9617008	2.616142	0.584	0.32	6.75E-50
PRR11	5.08E-71	0.9614544	2.615498	0.861	0.74	1.60E-66
MALL	3.17E-61	0.9612281	2.614906	0.661	0.376	9.99E-57
AC087721.2	1.61E-68	0.9588084	2.608586	0.548	0.266	5.09E-64
TSHZ1	1.04E-101	0.958539	2.607884	0.736	0.349	3.27E-97
MYO1D	9.89E-75	0.9571828	2.604349	0.464	0.139	3.12E-70
UCP2	2.07E-73	0.9559165	2.601053	0.341	0.055	6.54E-69
ARID3A	6.64E-125	0.9511361	2.588649	0.652	0.206	2.09E-120
TRIM59	3.26E-89	0.9503477	2.586609	0.779	0.48	1.03E-84
IFIT5	4.7E-122	0.9482113	2.581089	0.836	0.464	1.49E-117
TMSB10	0.00E+00	0.9457829	2.574828	1	1	0.00E+00
DBF4	1.74E-58	0.9456949	2.574602	0.565	0.32	5.49E-54
HMGB1	0.00E+00	0.945582	2.574311	1	1	0.00E+00
PAPSS2	1.1E-225	0.9451485	2.573196	0.999	0.998	3.58E-221
NEDD8	0.00E+00	0.9450144	2.57285	1	0.99	0.00E+00
PALLD	1.4E-145	0.9446356	2.571876	1	0.999	4.36E-141
FTL	0.00E+00	0.9429397	2.567518	1	1	0.00E+00
GPR37	1.14E-80	0.9427244	2.566965	0.3	0.025	3.59E-76
TACC1	9.60E-222	0.9427224	2.56696	0.997	0.964	3.03E-217
DHCR24	5.68E-118	0.941294	2.563296	0.905	0.664	1.79E-113
APOL6	1.58E-88	0.9403509	2.56088	0.824	0.541	4.97E-84
ZWINT	3.92E-82	0.939784	2.559429	0.696	0.347	1.24E-77
LGALS1	0.00E+00	0.9396457	2.559075	1	1	0.00E+00
NEDD1	1.87E-95	0.9393061	2.558206	0.815	0.507	5.89E-91
AC090409.1	9.84E-91	0.9389668	2.557338	0.451	0.101	3.10E-86
RAB3B	4.61E-299	0.9345495	2.546066	0.999	0.983	1.45E-294
SLC8A1	3.35E-122	0.9341057	2.544936	0.848	0.482	1.06E-117
KCNMB4	2.99E-68	0.932575	2.541044	0.259	0.023	9.42E-64
MRPL34	5.87E-121	0.9299405	2.534358	0.839	0.516	1.85E-116
TRPV2	1.9E-116	0.9290797	2.532178	0.698	0.261	6.13E-112
GPX4	0.00E+00	0.9288008	2.531472	1	0.998	0.00E+00
LPCAT2	7.66E-137	0.9262325	2.524978	0.918	0.659	2.42E-132
ETHE1	5.99E-139	0.9256082	2.523403	0.953	0.807	1.89E-134
GYPC	8.67E-114	0.9250706	2.522046	0.918	0.718	2.73E-109
CCDC68	3.64E-81	0.9236337	2.518425	0.377	0.064	1.15E-76
NRGN	9.97E-97	0.923202	2.517338	0.455	0.09	3.14E-92
NDUFB4	4.01966868	0.9214365	2.512898	0.997	0.904	1.26784370088202e-3

MSRB2	1.30E-129	0.9193609	2.507687	0.892	0.68	4.10E-125
S1PR1	6.82E-77	0.9188369	2.506374	0.641	0.305	2.15E-72
KNSTRN	6.00E-51	0.9176482	2.503396	0.533	0.278	1.89E-46
KIF22	6.31E-62	0.9167373	2.501117	0.651	0.361	1.99E-57
SNHG5	0.00E+00	0.9164443	2.500384	1	0.964	0.00E+00
LMNB1	5.52E-55	0.9148535	2.49641	0.551	0.284	1.74E-50
ANXA3	2.51E-70	0.9136367	2.493374	0.393	0.091	7.90E-66
SAMD11	2.07E-115	0.9133949	2.492771	0.504	0.095	6.53E-111
HMGB1P5	1.14E-222	0.9133739	2.492718	0.982	0.788	3.61E-218
SPC25	9.72E-52	0.9133506	2.49266	0.432	0.198	3.06E-47
ZNF22	1.53E-115	0.9133302	2.49261	0.866	0.543	4.84E-111
RIN2	7.44E-136	0.911134	2.487141	0.916	0.624	2.35E-131
H2AZ1	6.35E-150	0.9094384	2.482928	1	0.996	2.00E-145
GABARAPL1	1.43E-74	0.9092159	2.482375	0.883	0.841	4.51E-70
CSF2RB	9.69E-56	0.9087609	2.481246	0.243	0.028	3.06E-51
NHP2	2.15E-227	0.9080365	2.479449	0.993	0.965	6.79E-223
CEP57	3.52E-136	0.9071409	2.47723	0.888	0.534	1.11E-131
MACIR	1.24E-86	0.9061939	2.474885	0.862	0.598	3.91E-82
SMCHD1	5.85E-93	0.8981493	2.455055	0.852	0.59	1.85E-88
ARID5B	1.95E-121	0.8980251	2.45475	0.981	0.939	6.16E-117
NCAPD2	2.14E-64	0.8934395	2.44352	0.732	0.505	6.75E-60
KIF4A	5.38E-42	0.892907	2.442219	0.494	0.305	1.70E-37
NCAPH	9.92E-43	0.892792	2.441938	0.485	0.282	3.13E-38
ATF7IP	4.51E-103	0.8921057	2.440263	0.768	0.404	1.42E-98
PSMB9	4.38E-72	0.8892777	2.433371	0.716	0.49	1.38E-67
PABPC4L	7.02E-67	0.888708	2.431986	0.274	0.03	2.21E-62
TMEM60	4.30E-99	0.8885143	2.431514	0.774	0.412	1.36E-94
TLE4	6.29E-87	0.8884937	2.431464	0.777	0.441	1.98E-82
TMSB4XP6	4.60E-248	0.8866462	2.426976	0.928	0.544	1.45E-243
CHCHD10	4.03E-127	0.8862639	2.426049	0.773	0.372	1.27E-122
ATP2B1	2.56E-186	0.8862065	2.425909	0.999	0.983	8.09E-182
ITPRID2	1.76E-156	0.8835527	2.41948	0.993	0.963	5.54E-152
TMPO	2.26E-71	0.8832896	2.418844	0.86	0.685	7.14E-67
SMC4	1.90E-55	0.8827582	2.417559	0.924	0.882	6.01E-51
CASP1	8.76E-60	0.8781985	2.40656	0.409	0.124	2.76E-55
LINC01391	3.32E-110	0.8780155	2.40612	0.297	0	1.05E-105
SGO1	3.86E-42	0.8770937	2.403903	0.387	0.179	1.22E-37
RAD21	4.14E-170	0.8770884	2.40389	1	0.993	1.31E-165
TRIM2	4.18E-94	0.8766298	2.402788	0.807	0.534	1.32E-89
ECT2	9.05E-66	0.8759833	2.401235	0.731	0.441	2.86E-61

PRTFDC1	2.65E-90	0.8755348	2.400159	0.711	0.36	8.37E-86
AC007744.1	8.04E-114	0.8749171	2.398676	0.453	0.061	2.54E-109
EMX2OS	4.28E-85	0.8710912	2.389517	0.625	0.294	1.35E-80
NEK2	7.05E-40	0.8708575	2.388959	0.421	0.197	2.22E-35
ANAPC15	2.60E-122	0.8703387	2.387719	0.92	0.715	8.19E-118
ITGAE	2.33E-158	0.8695223	2.385771	0.957	0.791	7.35E-154
HOXA5	1.18E-77	0.8675148	2.380986	0.541	0.2	3.72E-73
INPP4B	2.74E-73	0.8661213	2.377671	0.668	0.342	8.64E-69
MT-CO2	0.00E+00	0.8658494	2.377024	1	1	0.00E+00
COL7A1	3.98E-79	0.8638619	2.372305	0.72	0.385	1.26E-74
SSX2IP	8.49E-91	0.8634512	2.371331	0.828	0.534	2.68E-86
DHRS7	1.59E-137	0.8630163	2.370299	0.963	0.859	5.00E-133
TMEM205	2.30E-95	0.8618609	2.367562	0.749	0.436	7.25E-91
TXNIP	1.27E-54	0.8617268	2.367245	0.667	0.387	4.00E-50
HJURP	7.77E-34	0.8609327	2.365366	0.413	0.255	2.45E-29
BST2	2.73E-31	0.8600573	2.363296	0.294	0.121	8.61E-27
CKAP5	2.59E-91	0.8597103	2.362476	0.92	0.719	8.17E-87
FAM111A	1.44E-69	0.8588537	2.360453	0.676	0.378	4.55E-65
BAX	1.63E-213	0.8587522	2.360214	0.997	0.954	5.14E-209
ST3GAL5	1.77E-54	0.8587292	2.36016	0.588	0.35	5.58E-50
PFDN5	0.00E+00	0.8581457	2.358783	1	0.999	0.00E+00
H1-0	2.01E-80	0.8581014	2.358678	0.87	0.62	6.34E-76
NR2F6	2.19E-115	0.8578237	2.358023	0.803	0.415	6.91E-111
FMN2	1.77E-98	0.8576868	2.3577	0.737	0.344	5.59E-94
RPL39L	5.48E-72	0.857028	2.356148	0.641	0.359	1.73E-67
HMGA1	0.00E+00	0.8568522	2.355734	1	0.998	0.00E+00
MICB	1.33E-85	0.8554926	2.352533	0.493	0.134	4.20E-81
CDH4	1.52E-62	0.8539366	2.348875	0.508	0.201	4.78E-58
HYI	1.20E-87	0.8524753	2.345445	0.855	0.646	3.79E-83
F10	4.85E-85	0.8524345	2.34535	0.303	0.02	1.53E-80
KRCC1	7.19E-124	0.8520958	2.344555	0.922	0.681	2.27E-119
CCDC50	2.60E-214	0.8516271	2.343457	0.998	0.984	8.19E-210
LSM2	2.30E-97	0.8516073	2.34341	0.855	0.653	7.25E-93
INA	1.95E-65	0.8505109	2.340842	0.257	0.023	6.15E-61
TCEAL7	2.08E-79	0.8494957	2.338467	0.467	0.124	6.57E-75
CFL2	4.40E-295	0.8490536	2.337434	1	0.984	1.39E-290
MELK	2.17E-61	0.8449313	2.327818	0.662	0.361	6.84E-57
L3MBTL3	4.20E-61	0.8432043	2.323801	0.495	0.195	1.32E-56
TMEM98	2.83E-148	0.8418641	2.320689	0.975	0.806	8.93E-144
UQCC3	4.72E-101	0.841613	2.320106	0.717	0.39	1.49E-96

H2AX	3.29E-43	0.8413746	2.319553	0.827	0.805	1.04E-38
STMN2	2.58E-21	0.8403128	2.317092	0.133	0.029	8.15E-17
C1orf35	1.38E-95	0.8398487	2.316017	0.634	0.263	4.37E-91
MGME1	7.24E-75	0.8393712	2.314911	0.687	0.368	2.28E-70
COX8A	0.00E+00	0.8390128	2.314081	0.999	0.982	0.00E+00
TBC1D2	1.99E-79	0.8389332	2.313897	0.743	0.416	6.28E-75
KATNAL1	4.64E-97	0.8388457	2.313695	0.861	0.588	1.46E-92
ETAA1	4.72E-81	0.8388299	2.313658	0.655	0.3	1.49E-76
FADS1	2.59E-143	0.8385878	2.313098	0.981	0.873	8.18E-139
IL7R	1.93E-48	0.838298	2.312428	0.594	0.35	6.08E-44
ZCRB1	2.3E-174	0.835728	2.306493	0.961	0.799	7.34E-170
NDUFA4	0.00E+00	0.8356563	2.306327	1	1	0.00E+00
TP53I3	3.04E-52	0.8355293	2.306034	0.862	0.698	9.58E-48
ID2	1.52E-116	0.8347876	2.304325	0.961	0.813	4.78E-112
TMEM159	4.20E-67	0.8338505	2.302166	0.801	0.576	1.32E-62
G2E3	1.86E-63	0.8329977	2.300204	0.767	0.549	5.88E-59
AP1M2	1.95E-88	0.8329411	2.300073	0.312	0.02	6.15E-84
ANGPT1	1.59E-103	0.832025	2.297967	0.955	0.813	5.03E-99
ITGA8	1.74E-36	0.8314793	2.296714	0.268	0.082	5.47E-32
ISCA2	1.42E-83	0.8294309	2.292014	0.759	0.47	4.48E-79
MRPL41	7.12E-197	0.8288238	2.290623	0.961	0.796	2.25E-192
TANC1	7.16E-73	0.828401	2.289655	0.728	0.426	2.26E-68
ACAT2	4.90E-101	0.8276984	2.288046	0.898	0.641	1.55E-96
GBP1	7.00E-95	0.8270328	2.286524	0.938	0.82	2.21E-90
NCKAP5	6.07E-45	0.8260914	2.284372	0.465	0.213	1.91E-40
IRF2BPL	7.26E-112	0.8258871	2.283906	0.802	0.409	2.29E-107
CYB5R2	1.28E-79	0.8246339	2.281046	0.522	0.169	4.04E-75
CCDC107	3.01E-92	0.8245396	2.28083	0.842	0.573	9.51E-88
ANAPC11	2.93E-273	0.824244	2.280156	0.998	0.983	9.23E-269
IRF1	1.58E-77	0.8237594	2.279052	0.766	0.452	5.00E-73
DYNLRB1	1.71E-261	0.8237044	2.278926	0.997	0.964	5.40E-257
NPC2	3.34E-146	0.8236059	2.278702	0.995	0.984	1.05E-141
BEX3	4.69E-271	0.8224848	2.276149	1	1	1.48E-266
CDK1	4.83E-25	0.821607	2.274151	0.538	0.362	1.52E-20
INSYN2B	5.00E-78	0.8204924	2.271618	0.645	0.285	1.58E-73
UCHL1	1.97E-131	0.8204913	2.271616	0.97	0.905	6.23E-127
HIP1	1.83E-120	0.8204116	2.271434	0.94	0.725	5.78E-116
JAZF1	4.11E-91	0.8181054	2.266202	0.889	0.693	1.30E-86
MOXD1	3.98E-58	0.8180746	2.266132	0.55	0.253	1.25E-53
LDB2	4.66E-81	0.8180446	2.266064	0.777	0.463	1.47E-76

MRPL54	2.81E-128	0.8162655	2.262037	0.878	0.628	8.86E-124
SNRPC	1.95E-209	0.813911	2.256717	1	0.992	6.14E-205
TRIM14	6.06E-72	0.8138365	2.256549	0.498	0.171	1.91E-67
ASF1A	1.64E-98	0.8128401	2.254301	0.802	0.438	5.18E-94
WNT5A	4.60E-61	0.8114973	2.251276	0.964	0.939	1.45E-56
NCAM2	2.00E-61	0.8106204	2.249303	0.418	0.127	6.30E-57
MAN2A1	4.65E-119	0.8095561	2.24691	0.96	0.839	1.47E-114
INPPL1	7.41E-100	0.8083695	2.244246	0.91	0.703	2.34E-95
REPIN1	2.53E-87	0.8074352	2.24215	0.817	0.502	7.98E-83
MGST1	2.89E-144	0.8073415	2.24194	0.936	0.993	9.12E-140
F8A1	9.96E-90	0.8068276	2.240788	0.663	0.289	3.14E-85
PARPBP	2.81E-50	0.8063651	2.239752	0.569	0.32	8.88E-46
FZD6	3.04E-101	0.8061403	2.239248	0.91	0.674	9.60E-97
SLC9A3R2	1.20E-139	0.8060199	2.238979	0.946	0.667	3.78E-135
NUDT2	9.96E-88	0.8059293	2.238776	0.784	0.47	3.14E-83
PTMA	0.00E+00	0.8031121	2.232478	1	1	0.00E+00
MOSPD2	1.63E-80	0.8031013	2.232454	0.837	0.573	5.13E-76
NECTIN3	5.59E-146	0.8029021	2.232009	0.994	0.946	1.76E-141
DTWD1	2.75E-103	0.8019599	2.229907	0.83	0.507	8.66E-99
CENPJ	7.65E-43	0.8018724	2.229712	0.492	0.255	2.41E-38
CYB5A	7.36E-101	0.8013144	2.228468	0.909	0.739	2.32E-96
TAF1B	1.12E-99	0.8009006	2.227546	0.787	0.42	3.52E-95
SCD	1.11E-78	0.7999172	2.225357	0.951	0.836	3.51E-74
EID1	0.00E+00	0.7987839	2.222836	1	1	0.00E+00
SLFN5	3.32E-96	0.7976466	2.220309	0.926	0.737	1.05E-91
JADE2	1.16E-89	0.7976412	2.220298	0.464	0.104	3.66E-85
CENPX	3.52E-106	0.7975584	2.220114	0.933	0.802	1.11E-101
MSMO1	2.14E-59	0.7973865	2.219732	0.839	0.624	6.75E-55
PDP2	1.33E-61	0.7973725	2.219701	0.581	0.299	4.19E-57
KPNA2	9.45E-38	0.7972369	2.2194	0.951	0.911	2.98E-33
TRPC4	1.33E-68	0.795276	2.215052	0.566	0.231	4.19E-64
APCDD1	3.03E-40	0.7944326	2.213185	0.218	0.04	9.55E-36
DBI	4.86E-178	0.7938886	2.211981	0.981	0.888	1.53E-173
CBR3	1.99E-54	0.7936777	2.211515	0.639	0.374	6.26E-50
IGF2BP1	6.51E-46	0.7934645	2.211043	0.438	0.213	2.05E-41
MRPL51	3.80E-231	0.7931991	2.210457	0.997	0.982	1.20E-226
S100A13	1.67E-212	0.7910858	2.20579	0.993	0.899	5.28E-208
FBLN1	2.30E-109	0.7910465	2.205703	0.943	0.747	7.26E-105
AC011295.1	1.09E-230	0.789562	2.202432	1	1	3.45E-226
BUB1B	7.75E-40	0.7893579	2.201982	0.433	0.206	2.44E-35

HOXA9	1.38E-82	0.7893318	2.201925	0.621	0.261	4.36E-78
SKA1	5.36E-38	0.7893061	2.201868	0.49	0.32	1.69E-33
PM20D2	5.88E-61	0.7888713	2.200911	0.456	0.168	1.85E-56
REV1	7.60E-67	0.7880785	2.199167	0.611	0.288	2.40E-62
RAD1	7.58E-89	0.7875195	2.197938	0.884	0.655	2.39E-84
TMEM154	5.69E-60	0.7874219	2.197723	0.549	0.259	1.79E-55
RTL10	2.38E-67	0.7869652	2.19672	0.501	0.183	7.52E-63
AC243919.1	5.20E-212	0.7866854	2.196105	0.999	1	1.64E-207
ZMYM3	1.62E-70	0.7861851	2.195007	0.55	0.211	5.11E-66
GTF3C6	3.24E-205	0.7859356	2.194459	0.995	0.972	1.02E-200
CARD9	5.02E-87	0.7853525	2.19318	0.588	0.225	1.58E-82
MT-ATP8	4.73E-259	0.7852643	2.192986	0.986	0.881	1.49E-254
PPM1K	1.79E-53	0.785006	2.19242	0.631	0.387	5.65E-49
H3-3A	0	0.7843161	2.190908	1	1	0
MTATP6P1	0.00E+00	0.7841449	2.190533	1	1	0.00E+00
ADCY9	8.95E-67	0.7840621	2.190352	0.586	0.276	2.82E-62
ARHGEF28	3.45E-48	0.7833689	2.188834	0.418	0.167	1.09E-43
CD248	2.51E-211	0.781251	2.184203	0.998	0.952	7.93E-207
FDX2	6.94E-81	0.7811232	2.183924	0.717	0.418	2.19E-76
NDFIP2	1.96E-102	0.7800916	2.181672	0.966	0.914	6.17E-98
TMEM126A	2.30E-111	0.7799665	2.181399	0.947	0.839	7.26E-107
C16orf87	4.60E-87	0.7788862	2.179044	0.804	0.492	1.45E-82
PNKD	2.35E-105	0.7784486	2.17809	0.858	0.616	7.41E-101
TRAM2	1.21E-183	0.7783302	2.177833	0.999	0.986	3.82E-179
PEG10	1.89E-39	0.7768388	2.174587	0.415	0.194	5.97E-35
RRM2B	4.04E-81	0.7768023	2.174508	0.895	0.703	1.27E-76
MRPS6	3.08E-128	0.7762889	2.173392	0.962	0.815	9.71E-124
DENND2B	2.19E-81	0.7761598	2.173111	0.813	0.513	6.91E-77
POC1A	2.94E-46	0.7757305	2.172178	0.444	0.185	9.26E-42
NOG	7.06E-65	0.7756744	2.172057	0.272	0.031	2.23E-60
PCDH9	6.84E-16	0.775358	2.171369	0.675	0.571	2.16E-11
POPDC3	8.38E-84	0.7751209	2.170855	0.802	0.493	2.64E-79
KIF15	1.68E-39	0.7746876	2.169914	0.367	0.151	5.31E-35
GMNN	5.15E-58	0.7746061	2.169737	0.693	0.424	1.63E-53
AC068831.6	3.76E-62	0.7744289	2.169353	0.642	0.363	1.19E-57
XPC	8.58E-77	0.7737174	2.16781	0.715	0.373	2.71E-72
PCLAF	6.74E-73	0.7730673	2.166401	0.853	0.703	2.13E-68
ARHGAP29	2.09E-65	0.7729389	2.166123	0.837	0.644	6.59E-61
PRNP	3.36E-164	0.7726145	2.16542	1	1	1.06E-159
RAD51AP1	6.35E-38	0.7725133	2.165201	0.447	0.225	2.00E-33

MDK	8.32E-68	0.772206	2.164536	0.835	0.606	2.62E-63
TSPAN9	2.27E-69	0.771968	2.164021	0.629	0.308	7.14E-65
FBXL5	2.91E-85	0.7717898	2.163635	0.784	0.457	9.17E-81
NSL1	8.51E-93	0.771552	2.163121	0.828	0.519	2.68E-88
AC010735.2	1.19E-57	0.7715225	2.163057	0.384	0.108	3.75E-53
NCALD	7.25E-56	0.7712754	2.162523	0.312	0.07	2.29E-51
HOXD8	8.49E-76	0.771262	2.162494	0.634	0.291	2.68E-71
HPF1	3.10E-73	0.7710152	2.16196	0.722	0.428	9.77E-69
HMGB1P6	1.32E-152	0.7708145	2.161526	0.897	0.548	4.16E-148
SNRNP25	6.71E-60	0.7702317	2.160267	0.765	0.588	2.12E-55
SNRPD2	2.57913403	0.7701923	2.160182	1	0.989	8.13E-306
PIF1	4.32E-39	0.7699513	2.159661	0.411	0.201	1.36E-34
TFB1M	8.78E-77	0.7694673	2.158616	0.58	0.222	2.77E-72
SOX4	3.30E-24	0.7693558	2.158375	0.798	0.765	1.04E-19
CDC25C	1.42E-34	0.7688577	2.157301	0.291	0.107	4.48E-30
DDX58	6.54E-57	0.7683441	2.156193	0.389	0.119	2.06E-52
CENPM	1.71E-50	0.7681816	2.155842	0.59	0.335	5.4E-46
TIMP2	0.00E+00	0.7678661	2.155163	1	1	0.00E+00
SPC24	2.87E-63	0.7678427	2.155112	0.552	0.247	9.04E-59
TCEAL1	1.57E-72	0.7669975	2.153291	0.757	0.474	4.96E-68
MRPL23	1.7E-156	0.7652117	2.149449	0.964	0.815	5.47E-152
MT-ATP6	0	0.7651325	2.149279	1	1	0
HINT1	0.00E+00	0.7645251	2.147974	1	1	0.00E+00
CPT1C	2.28E-62	0.7635826	2.145951	0.577	0.281	7.18E-58
FRMPD4	1.73E-63	0.7629221	2.144534	0.327	0.06	5.47E-59
NDUFB10	3.49E-176	0.7613155	2.141091	0.991	0.939	1.10E-171
ADGRA2	3.37E-94	0.7598564	2.137969	0.93	0.753	1.06E-89
CHAMP1	4.01E-79	0.7597512	2.137744	0.59	0.237	1.27E-74
LIMCH1	1.36E-49	0.7596244	2.137473	0.448	0.175	4.29E-45
MEIS2	2.34E-83	0.7584885	2.135047	0.863	0.595	7.39E-79
SKA2	2.36E-114	0.7584126	2.134885	0.944	0.763	7.46E-110
LRIG3	1.37E-56	0.7575751	2.133097	0.379	0.109	4.31E-52
SMIM29	4.73E-59	0.7575517	2.133048	0.707	0.462	1.49E-54
NDUFB6	1.43E-215	0.7573605	2.13264	0.998	0.978	4.51E-211
CIT	8.54E-48	0.7572187	2.132337	0.484	0.228	2.69E-43
GNB5	1.75E-83	0.7571717	2.132237	0.873	0.653	5.53E-79
PARP4	1.72E-82	0.7566119	2.131044	0.854	0.596	5.44E-78
CARHSP1	2.78E-109	0.7553059	2.128262	0.934	0.759	8.77E-105
DDX60L	5.06E-60	0.7549214	2.127444	0.612	0.312	1.60E-55
LINC01085	4.99E-66	0.7548853	2.127367	0.338	0.064	1.57E-61

RPL22L1	6.78E-167	0.7546469	2.12686	0.994	0.957	2.14E-162
TRIP13	1.80E-47	0.7546314	2.126827	0.592	0.347	5.67E-43
HAUS1	6.01E-79	0.7546217	2.126807	0.783	0.493	1.89E-74
CIP2A	6.20E-40	0.7534524	2.124321	0.597	0.378	1.95E-35
GCNT4	2.59E-57	0.7518994	2.121025	0.289	0.054	8.16E-53
CEP78	1.05E-54	0.7517206	2.120646	0.749	0.538	3.31E-50
FANCI	2.66E-51	0.751628	2.120449	0.63	0.4	8.38E-47
IGF2BP3	5.56E-41	0.7512643	2.119678	0.359	0.145	1.76E-36
DNMT1	1.58E-82	0.7510183	2.119157	0.916	0.752	4.97E-78
TPRKB	3.01E-70	0.7507568	2.118603	0.776	0.564	9.49E-66
LAMA5	6.07E-72	0.7497341	2.116437	0.348	0.064	1.91E-67
DHFR	1.43E-76	0.7481985	2.11319	0.889	0.707	4.51E-72
MKNK2	4.84E-70	0.7480193	2.112811	0.814	0.556	1.53E-65
NXN	8.00E-105	0.7477263	2.112192	0.914	0.646	2.52E-100
WFS1	2.60E-65	0.7473155	2.111325	0.76	0.501	8.20E-61
TNFAIP8L1	5.14E-49	0.7457652	2.108054	0.625	0.416	1.62E-44
PDGFB	2.93E-53	0.7450801	2.10661	0.171	0.004	9.26E-49
GSTP1	0.00E+00	0.7441013	2.104549	1	1	0.00E+00
NUCKS1	5.98E-242	0.7440872	2.10452	1	1	1.89E-237
CNTN3	1.48E-71	0.7426034	2.101399	0.335	0.052	4.68E-67
HNMT	4.77E-57	0.74244	2.101056	0.742	0.487	1.50E-52
PSME2	8.75E-191	0.7419199	2.099963	0.998	0.986	2.76E-186
BHLHE41	9.36E-53	0.7413962	2.098864	0.491	0.205	2.95E-48
RBX1	4.38E-226	0.7411507	2.098349	0.998	0.971	1.38E-221
AMOT	8.40E-50	0.7410525	2.098143	0.337	0.096	2.65E-45
FAT4	1.74E-52	0.740399	2.096772	0.566	0.287	5.48E-48
SPAG5	5.48E-31	0.7386556	2.09312	0.522	0.398	1.73E-26
GPX1	0.00E+00	0.7386167	2.093038	1	1	0.00E+00
CHCHD1	2.95E-132	0.7385002	2.092794	0.954	0.83	9.29E-128
LSM5	1.56E-96	0.738262	2.092296	0.878	0.689	4.91E-92
NDUFA12	8.89E-172	0.7379428	2.091628	0.99	0.92	2.81E-167
FRMD3	1.39E-41	0.7374849	2.090671	0.304	0.094	4.38E-37
MAP3K7CL	5.66E-53	0.7366314	2.088887	0.521	0.235	1.79E-48
SF3B5	8.46E-190	0.7364008	2.088405	0.996	0.994	2.67E-185
PLCB1	1.99E-64	0.7359403	2.087444	0.718	0.417	6.26E-60
MRPS31	3.50E-76	0.7356666	2.086873	0.585	0.251	1.10E-71
APH1B	8.98E-56	0.7352264	2.085954	0.587	0.311	2.83E-51
TRIB3	8.32E-50	0.7349539	2.085386	0.641	0.365	2.62E-45
METTL7A	3.06E-44	0.7346569	2.084767	0.48	0.239	9.66E-40
IFITM1	1.92E-95	0.7339758	2.083347	0.967	0.898	6.06E-91

CCDC81	8.99E-62	0.7334882	2.082332	0.275	0.038	2.84E-57
GTF2I	6.73E-192	0.7330235	2.081364	0.998	0.984	2.12E-187
NDC1	5.16E-53	0.73239	2.080046	0.632	0.36	1.63E-48
PLEK2	2.34E-48	0.7322387	2.079731	0.448	0.192	7.38E-44
MXD3	2.49E-32	0.7322164	2.079685	0.586	0.441	7.87E-28
XRRA1	8.02E-52	0.7307335	2.076603	0.492	0.218	2.53E-47
PTMS	9.13E-278	0.7305329	2.076187	1	1	2.88E-273
MRPL58	5.22E-80	0.7304158	2.075944	0.813	0.565	1.65E-75
LSM10	3.15E-76	0.73007	2.075226	0.847	0.635	9.94E-72
ZNF618	1.98E-72	0.7296812	2.074419	0.682	0.35	6.23E-68
AC007920.2	2.07E-67	0.7295924	2.074235	0.267	0.028	6.53E-63
LSM3	6.90E-115	0.7287772	2.072545	0.933	0.788	2.17E-110
SELENOM	6.13E-158	0.7285226	2.072017	0.999	0.995	1.93E-153
NDUFS5	1.83E-269	0.7278681	2.070661	1	0.993	5.78E-265
RANGAP1	5.18E-113	0.7276042	2.070115	0.956	0.785	1.63E-108
SLC7A2	3.74E-40	0.7273651	2.06962	0.226	0.047	1.18E-35
SMIM30	1.77E-120	0.7260207	2.06684	0.923	0.706	5.59E-116
TFAP2A	1.12E-16	0.7240536	2.062778	0.357	0.217	3.53E-12
RAI14	6.72E-143	0.724024	2.062717	0.998	0.995	2.12E-138
CROT	4.72E-55	0.7237638	2.06218	0.52	0.233	1.49E-50
C19orf53	5.54E-186	0.7230031	2.060612	0.992	0.933	1.75E-181
AKIP1	4.82E-82	0.72292	2.060441	0.863	0.609	1.52E-77
GREM2	1.29E-26	0.7227041	2.059996	0.533	0.361	4.08E-22
RPA3	1.05E-62	0.7226456	2.059876	0.699	0.432	3.30E-58
ABCC4	3.69E-62	0.7212247	2.056951	0.791	0.554	1.16E-57
RSRC1	3.73E-68	0.7206962	2.055864	0.666	0.361	1.18E-63
CYBA	2.46E-97	0.7199378	2.054305	0.912	0.809	7.75E-93
WEE1	2.27E-84	0.7196392	2.053692	0.718	0.356	7.16E-80
PHPT1	2.72E-189	0.7196323	2.053678	0.993	0.941	8.56E-185
TCF12	5.60E-117	0.7189123	2.0522	0.974	0.88	1.77E-112
CABIN1	3.13E-68	0.7187368	2.05184	0.665	0.338	9.88E-64
AC138811.2	1.16E-58	0.7176355	2.049581	0.876	0.763	3.66E-54
FLOT1	5.42E-197	0.7171306	2.048547	0.998	0.948	1.71E-192
JADE1	1.41E-75	0.7166889	2.047642	0.802	0.494	4.45E-71
AJUBA	3.51E-93	0.7165196	2.047295	0.833	0.489	1.11E-88
CIAO2B	6.40E-176	0.7156057	2.045425	0.992	0.964	2.02E-171
APOBEC3B	1.07E-53	0.7151045	2.0444	0.527	0.233	3.37E-49
HMCES	9.30E-59	0.7149546	2.044094	0.734	0.454	2.93E-54
MICU3	5.79E-50	0.7149432	2.04407	0.606	0.338	1.83E-45
FAM155A	6.48E-47	0.7147542	2.043684	0.61	0.354	2.05E-42

PCGF2	1.44E-68	0.7145304	2.043227	0.765	0.484	4.54E-64
WNK4	7.23E-49	0.7143301	2.042818	0.433	0.187	2.28E-44
HCFC1R1	6.27E-76	0.7140107	2.042165	0.777	0.505	1.98E-71
TBL1X	2.93E-72	0.7129842	2.04007	0.86	0.634	9.24E-68
NAPRT	1.14E-59	0.7115256	2.037097	0.51	0.201	3.60E-55
SSPN	1.07E-68	0.7097776	2.033539	0.862	0.646	3.38E-64
SNRPE	1.75E-165	0.709266	2.032499	0.98	0.866	5.52E-161
DZIP1	1.04E-80	0.7090842	2.032129	0.906	0.718	3.28E-76
MAML2	5.43E-60	0.7086349	2.031217	0.673	0.373	1.71E-55
SAMD9L	4.68E-45	0.7085414	2.031027	0.663	0.42	1.47E-40
RNASEH2A	2.85E-53	0.7083143	2.030565	0.717	0.476	8.99E-49
CRIM1	5.71E-199	0.7080004	2.029928	1	0.999	1.80E-194
SPHKAP	7.32E-52	0.7075135	2.02894	0.164	0.002	2.31E-47
PSIP1	1.27E-110	0.7071294	2.028161	0.977	0.861	4.02E-106
NDUFAB1	4.97E-160	0.7069018	2.027699	0.991	0.972	1.57E-155
MSRA	2.64E-60	0.7053167	2.024488	0.665	0.38	8.31E-56
PBX3	4.30E-52	0.7052424	2.024337	0.698	0.457	1.35E-47
GATA2-AS1	6.01E-89	0.7052041	2.02426	0.349	0.038	1.89E-84
COX7A1	3.53E-114	0.7051082	2.024066	0.577	0.151	1.11E-109
CDKN2D	1.67E-24	0.7049909	2.023828	0.553	0.406	5.26E-20
KIFBP	5.20E-94	0.7045815	2.023	0.938	0.781	1.64E-89
HDAC9	3.39E-44	0.7044082	2.022649	0.264	0.059	1.07E-39
ATP10D	1.46E-44	0.7040482	2.021921	0.66	0.44	4.61E-40
RTKN2	4.12E-42	0.7040296	2.021884	0.313	0.097	1.30E-37
ANKRD34A	2.38E-100	0.703596	2.021007	0.749	0.393	7.51E-96
OPN3	2.02E-53	0.7030769	2.019958	0.509	0.219	6.39E-49
OSBPL1A	6.83E-67	0.7028962	2.019593	0.82	0.586	2.15E-62
OIP5	4.39E-36	0.7025201	2.018834	0.38	0.159	1.38E-31
MAD2L1	2.18E-44	0.7020506	2.017886	0.682	0.46	6.89E-40
MZT2B	1.4E-213	0.7015219	2.01682	0.996	0.972	4.51E-209
RPL29	0.00E+00	0.7014695	2.016714	1	1	0.00E+00
NQO2	4.79E-67	0.7009836	2.015734	0.8	0.53	1.51E-62
RNF150	4.08E-45	0.6998843	2.01352	0.548	0.279	1.29E-40
LPIN1	9.41E-51	0.6998588	2.013468	0.705	0.471	2.97E-46
NUDT1	1.33E-74	0.6997705	2.013291	0.778	0.564	4.21E-70
ZDHHC17	1.65E-60	0.6992235	2.01219	0.791	0.546	5.21E-56
SNX6	7.24E-236	0.6990959	2.011933	1	1	2.28E-231
TNS2	6.11E-56	0.6990552	2.011851	0.661	0.373	1.93E-51
C8orf58	1.95E-50	0.6990296	2.0118	0.503	0.225	6.16E-46
UBL4A	3.13E-60	0.6989532	2.011646	0.644	0.337	9.89E-56

PGAP4	1.30E-55	0.6988781	2.011495	0.503	0.205	4.09E-51
RND3	1.37E-139	0.6986581	2.011052	1	0.999	4.32E-135
MGST3	2.26E-157	0.697567	2.008859	0.998	0.998	7.11E-153
NEIL3	4.33E-41	0.6973071	2.008337	0.313	0.098	1.37E-36
CMC1	3.06E-79	0.6967183	2.007155	0.745	0.447	9.65E-75
TP53TG1	2.18E-54	0.6965778	2.006873	0.579	0.336	6.88E-50
CHEK1	1.35E-69	0.6954527	2.004616	0.748	0.44	4.25E-65
CTDSPL	5.47E-61	0.6954397	2.00459	0.75	0.482	1.72E-56
NDUFS4	1.21E-114	0.6953798	2.00447	0.953	0.818	3.83E-110
USP53	1.95E-30	0.6947855	2.003279	0.603	0.429	6.14E-26
NQO1	1.95E-189	0.6947565	2.003221	1	1	6.14E-185
NDUFB11	8.70E-154	0.6947118	2.003132	0.984	0.91	2.74E-149
AL365205.1	1.11E-145	0.6944949	2.002697	0.979	0.893	3.50E-141
SNX18	3.53E-63	0.6941431	2.001993	0.835	0.618	1.11E-58
NUP62	1.09E-70	0.693981	2.001668	0.91	0.747	3.45E-66
TRMT1L	2.71E-54	0.6932211	2.000148	0.569	0.27	8.54E-50
PSMB10	1.42E-58	0.6930774	1.99986	0.741	0.558	4.48E-54
SETDB2	2.41E-49	0.6930238	1.999753	0.476	0.217	7.61E-45
HSPB11	1.10E-74	0.691947	1.997601	0.852	0.676	3.46E-70
TXNRD1	3.37E-136	0.6918976	1.997502	1	1	1.06E-131
NDUFB3	4.41E-138	0.6914252	1.996559	0.964	0.827	1.39E-133
NUDT12	9.28E-63	0.6907359	1.995183	0.493	0.18	2.93E-58
TCEAL4	1.55E-93	0.6905758	1.994864	0.975	0.881	4.89E-89
SMIM10	5.89E-59	0.6902884	1.994291	0.553	0.267	1.86E-54
SCRN1	1.25E-103	0.6897841	1.993285	0.953	0.785	3.95E-99
TFAP2C	1.54E-52	0.6896314	1.992981	0.26	0.042	4.87E-48
ATP5PO	2.25E-219	0.6893697	1.992459	1	1	7.09E-215
IL13RA2	7.17E-16	0.6887872	1.991299	0.184	0.076	2.26E-11
PARP9	9.22E-52	0.6885744	1.990875	0.505	0.218	2.91E-47
ZNF480	6.51E-56	0.6877649	1.989264	0.529	0.24	2.05E-51
KRT10	2.25E-186	0.6876542	1.989044	0.998	0.98	7.11E-182
ZDHHC2	3.46E-80	0.6873098	1.988359	0.904	0.707	1.09E-75
JUP	5.49E-29	0.6870659	1.987874	0.292	0.12	1.73E-24
LSM7	4.84E-130	0.6870614	1.987865	0.948	0.773	1.53E-125
MAPK8	4.36E-69	0.686571	1.986891	0.904	0.753	1.38E-64
SKP2	5.14E-64	0.686142	1.986039	0.625	0.308	1.62E-59
RWDD1	4.00E-138	0.6857659	1.985292	0.983	0.922	1.26E-133
ZMYM1	2.26E-45	0.6857442	1.985249	0.438	0.185	7.13E-41
CENPK	2.02E-40	0.6853967	1.984559	0.609	0.397	6.38E-36
BARD1	6.34E-39	0.6850784	1.983927	0.448	0.215	2.00E-34

RUNX1T1	7.18E-52	0.6850644	1.9839	0.487	0.205	2.27E-47
KBTBD6	6.05E-45	0.6841668	1.98212	0.54	0.288	1.91E-40
UQCC2	2.07E-107	0.6839911	1.981772	0.881	0.607	6.53E-103
GSTO1	8.06E-202	0.6838638	1.981519	1	0.998	2.54E-197
DENND3	3.46E-50	0.6837952	1.981383	0.376	0.124	1.09E-45
GINS1	5.89E-42	0.6837581	1.98131	0.388	0.162	1.86E-37
STAT2	9.54E-48	0.6828018	1.979416	0.723	0.502	3.01E-43
PARP2	5.26E-39	0.6826881	1.979191	0.544	0.308	1.66E-34
CNTLN	3.16E-60	0.6825202	1.978858	0.661	0.375	9.96E-56
SOX5	2.50E-56	0.6824708	1.978761	0.24	0.026	7.87E-52
EHD3	1.55E-25	0.6823719	1.978565	0.72	0.58	4.88E-21
ACYP1	1.06E-65	0.682273	1.97837	0.504	0.193	3.33E-61
TAF9B	3.36E-62	0.6815696	1.976978	0.655	0.339	1.06E-57
DIAPH3	3.06E-48	0.6812096	1.976267	0.847	0.725	9.67E-44
RNASEK	1.34E-181	0.6809941	1.975841	0.993	0.952	4.23E-177
PAFAH1B3	2.75E-67	0.6808921	1.97564	0.802	0.547	8.69E-63
BCAR1	1.71E-121	0.6808655	1.975587	0.971	0.802	5.40E-117
ITGB3BP	4.26E-39	0.6800993	1.974074	0.511	0.295	1.34E-34
COMMD8	5.59E-98	0.6797421	1.973369	0.913	0.665	1.76E-93
SDC1	8.29E-60	0.6797382	1.973361	0.697	0.418	2.62E-55
TRABD2A	6.48E-41	0.6796109	1.97311	0.382	0.162	2.04E-36
EAPP	4.45E-65	0.6795025	1.972896	0.814	0.564	1.40E-60
SEPTIN8	3.74E-73	0.679068	1.972039	0.827	0.559	1.18E-68
LARP7	6.99E-83	0.6783137	1.970552	0.881	0.668	2.20E-78
MEA1	2.16E-106	0.6780107	1.969955	0.974	0.923	6.82E-102
LRRC1	2.90E-48	0.6772755	1.968507	0.426	0.165	9.16E-44
MICOS10	1.62E-199	0.6771915	1.968342	0.997	0.968	5.12E-195
LRR1	2.22E-48	0.6770829	1.968128	0.646	0.402	6.99E-44
PSME1	1.21E-145	0.6761392	1.966272	0.995	0.977	3.82E-141
EVA1B	4.88E-125	0.6754988	1.965013	0.98	0.876	1.54E-120
NOL4L	3.58E-50	0.6753628	1.964746	0.485	0.213	1.13E-45
LSM6	9.62E-60	0.6751968	1.96442	0.725	0.504	3.03E-55
TIMELESS	5.57E-43	0.6751237	1.964276	0.539	0.278	1.76E-38
CENPH	1.72E-45	0.6743704	1.962797	0.606	0.345	5.43E-41
MRPL27	3.51E-98	0.6729006	1.959914	0.932	0.839	1.11E-93
COX6A1	4.60E-256	0.6727685	1.959655	1	0.99	1.45E-251
TADA2A	4.73E-52	0.6725076	1.959144	0.517	0.239	1.49E-47
ASB8	8.57E-72	0.6717362	1.957633	0.806	0.52	2.70E-67
RPAIN	3.01E-66	0.670417	1.955052	0.825	0.61	9.48E-62
ATP5MC1	6.23E-112	0.6700541	1.954343	0.954	0.831	1.96E-107

STARD7	3.74E-119	0.669087	1.952454	0.978	0.821	1.18E-114
ZWILCH	6.41E-43	0.6688854	1.95206	0.728	0.52	2.02E-38
LINC00888	2.43E-47	0.6688807	1.952051	0.429	0.17	7.67E-43
MRPS14	8.32E-73	0.6686793	1.951658	0.825	0.669	2.62E-68
FKBP3	1.40E-144	0.6685859	1.951476	0.99	0.938	4.41E-140
GTF2IP1	1.31E-151	0.6685619	1.951429	0.977	0.86	4.13E-147
CCDC28B	5.63E-47	0.6684438	1.951199	0.584	0.35	1.77E-42
TMEM52B	2.50E-44	0.6679371	1.95021	0.176	0.016	7.88E-40
SNAPIN	7.27E-61	0.6678063	1.949955	0.852	0.644	2.29E-56
STXBP3	6.04E-77	0.6677234	1.949793	0.874	0.626	1.90E-72
MRPL22	2.95E-73	0.6671497	1.948675	0.883	0.745	9.30E-69
SOCS1	9.20E-57	0.6670696	1.948519	0.407	0.124	2.90E-52
OSGIN2	5.16E-46	0.6666268	1.947656	0.575	0.312	1.63E-41
FIGN	5.84E-44	0.6665104	1.94743	0.263	0.056	1.84E-39
KCNJ8	3.00E-28	0.6659986	1.946433	0.32	0.143	9.46E-24
STIL	4.06E-31	0.6659532	1.946345	0.426	0.239	1.28E-26
UQCRH	1.20E-221	0.6657952	1.946037	1	0.994	3.79E-217
KISS1	4.40E-37	0.6649654	1.944423	0.151	0.013	1.39E-32
PLK4	7.41E-30	0.6642096	1.942954	0.35	0.163	2.34E-25
CEP57L1	2.78E-47	0.6635337	1.941641	0.594	0.341	8.76E-43
AARS1	6.61E-91	0.6634434	1.941466	0.965	0.844	2.09E-86
SCLT1	7.39E-41	0.6631402	1.940878	0.574	0.337	2.33E-36
DEK	2.00E-97	0.6630582	1.940718	0.992	0.97	6.31E-93
KLHL5	6.35E-65	0.6630199	1.940644	0.901	0.782	2.00E-60
SUPT20H	1.55E-84	0.6629546	1.940517	0.926	0.763	4.88E-80
H1-10	1.08E-66	0.6629154	1.940441	0.941	0.831	3.40E-62
TLE1	2.07E-52	0.6627502	1.940121	0.718	0.453	6.52E-48
TOMM6	1.38E-126	0.6626769	1.939978	0.947	0.781	4.35E-122
ARHGAP19	6.48E-38	0.6619938	1.938654	0.361	0.14	2.04E-33
HEATR6	2.99E-49	0.6617542	1.938189	0.601	0.323	9.42E-45
LURAP1L	3.30E-33	0.6610656	1.936855	0.379	0.173	1.04E-28
PLCB3	1.37E-61	0.6608313	1.936401	0.726	0.447	4.33E-57
SDF2L1	3.92E-56	0.6602141	1.935207	0.828	0.737	1.24E-51
LAMTOR2	3.02E-82	0.6602035	1.935186	0.88	0.711	9.52E-78
PSMA7	1.99E-299	0.6600188	1.934829	1	1	6.28E-295
SH3PXD2A	2.32E-95	0.6599577	1.934711	0.966	0.854	7.31E-91
SMDT1	3.62E-83	0.6596283	1.934073	0.839	0.635	1.14E-78
COX6B1	6.6E-175	0.6583331	1.93157	0.986	0.916	2.07E-170
AP2S1	0.00E+00	0.6579427	1.930816	1	1	0.00E+00
FOXM1	4.09E-44	0.6576887	1.930326	0.74	0.536	1.29E-39

ANP32B	2.21E-129	0.6574178	1.929803	0.998	0.987	6.97E-125
ELOB	2.57E-203	0.6572622	1.929503	0.998	0.963	8.10E-199
TSPAN13	1.78E-13	0.6561142	1.927289	0.364	0.248	5.62E-09
EXOSC8	1.77E-51	0.6560742	1.927212	0.838	0.663	5.59E-47
PDLIM1	8.7E-165	0.6560393	1.927144	1	0.976	2.75E-160
RPS20	0.00E+00	0.6549647	1.925075	1	1	0.00E+00
RPS4Y1	2.03E-23	0.6549232	1.924995	0.296	0.363	6.39E-19
TMEM204	1.32E-67	0.6543441	1.92388	0.707	0.373	4.18E-63
GPN3	5.52E-63	0.6537928	1.92282	0.785	0.538	1.74E-58
LAMA2	1.82E-45	0.6535793	1.922409	0.392	0.141	5.73E-41
DUBR	6.65E-45	0.6532259	1.92173	0.471	0.212	2.10E-40
OSGEPL1	2.67E-38	0.6532109	1.921701	0.351	0.133	8.42E-34
METTL5	3.35E-79	0.6530293	1.921352	0.911	0.73	1.06E-74
ABHD10	3.14E-55	0.6528156	1.920942	0.646	0.362	9.89E-51
ZC2HC1A	1.83E-52	0.6525714	1.920473	0.705	0.441	5.77E-48
SP110	6.35E-49	0.6525075	1.92035	0.607	0.341	2.00E-44
JPT1	8.84E-130	0.6522361	1.919829	0.999	0.984	2.79E-125
NIBAN1	1.80E-71	0.6518147	1.91902	0.894	0.675	5.69E-67
MSH2	1.09E-45	0.6517759	1.918946	0.633	0.392	3.43E-41
SARNP	3.97E-124	0.65136	1.918148	0.983	0.888	1.25E-119
B3GALT2	8.81E-40	0.6506199	1.916729	0.155	0.011	2.78E-35
PTPMT1	1.90E-75	0.6500882	1.91571	0.868	0.713	5.99E-71
NTAN1	1.93E-72	0.6494931	1.91457	0.859	0.635	6.10E-68
PSMB3	4.37E-180	0.6490419	1.913706	1	1	1.38E-175
ATP5MC2	5.30E-212	0.6489228	1.913479	1	0.996	1.67E-207
MSI2	5.05E-43	0.648856	1.913351	0.73	0.535	1.59E-38
HES1	8.10E-56	0.6486869	1.913027	0.527	0.228	2.56E-51
POP7	1.19E-51	0.6486343	1.912927	0.768	0.592	3.74E-47
DTYMK	1.87E-57	0.6484687	1.91261	0.918	0.801	5.90E-53
RNF157	2.84E-42	0.6482068	1.912109	0.354	0.145	8.97E-38
DSEL	4.83E-67	0.6478694	1.911464	0.984	0.97	1.52E-62
HLTF	5.82E-59	0.6475573	1.910867	0.806	0.573	1.84E-54
VRK1	1.04E-34	0.6475471	1.910848	0.522	0.314	3.27E-30
FLRT2	9.14E-39	0.6474869	1.910733	0.638	0.441	2.88E-34
MZT1	2.49E-42	0.6472368	1.910255	0.844	0.735	7.86E-38
GNG2	2.58E-35	0.6467676	1.909359	0.415	0.199	8.14E-31
TRAPPC2L	3.95E-71	0.646513	1.908873	0.789	0.584	1.24E-66
MCM5	2.92E-58	0.6464303	1.908715	0.706	0.406	9.22E-54
SNAPC5	1.44E-47	0.6459716	1.90784	0.581	0.312	4.53E-43
CD81	1.98E-295	0.6458753	1.907656	1	1	6.26E-291

NSMCE4A	1.23E-48	0.6457619	1.90744	0.657	0.391	3.87E-44
POLR3GL	4.97E-60	0.6455854	1.907103	0.703	0.446	1.57E-55
IDH1	2.48E-65	0.6454972	1.906935	0.913	0.737	7.81E-61
C9orf40	6.08E-40	0.6454943	1.906929	0.544	0.312	1.92E-35
NDUFS6	6.02E-144	0.6449069	1.90581	0.972	0.872	1.90E-139
PHC2	1.29E-87	0.6446743	1.905366	0.951	0.809	4.07E-83
PMAIP1	5.76E-85	0.6443131	1.904678	0.886	0.612	1.82E-80
TIA1	1.02E-91	0.6439449	1.903977	0.967	0.854	3.21E-87
VPS29	1.02E-143	0.6437262	1.903561	0.999	0.981	3.23E-139
NCAPG2	5.36E-38	0.6430871	1.902345	0.592	0.369	1.69E-33
MIEN1	6.97E-83	0.6427538	1.901711	0.911	0.826	2.20E-78
DHTKD1	7.69E-52	0.6425178	1.901262	0.654	0.381	2.43E-47
BTN3A2	4.76E-55	0.6424295	1.901094	0.741	0.483	1.50E-50
MRPL1	3.14E-65	0.6419019	1.900091	0.763	0.505	9.91E-61
LSP1P5	1.04E-84	0.6417695	1.89984	0.855	0.615	3.28E-80
MCM7	3.31E-58	0.6414318	1.899198	0.828	0.65	1.04E-53
TAP1	8.27E-60	0.6409506	1.898284	0.846	0.646	2.61E-55
SSB	8.12E-167	0.6409096	1.898207	1	0.996	2.56E-162
CETN3	2.13E-57	0.640271	1.896995	0.613	0.323	6.71E-53
KNTC1	4.99E-29	0.640228	1.896913	0.416	0.229	1.57E-24
LASP1	2.92E-169	0.6394168	1.895375	1	0.996	9.20E-165
PPP1R14B	3.97E-147	0.6388827	1.894363	0.993	0.969	1.25E-142
DUSP10	9.82E-58	0.6384425	1.893529	0.759	0.47	3.10E-53
ADCY3	7.60E-58	0.6377532	1.892225	0.598	0.285	2.40E-53
MIS18A	7.53E-34	0.6373722	1.891504	0.549	0.406	2.37E-29
XPO1	8.17E-133	0.6370706	1.890933	0.998	0.968	2.58E-128
MAGOHB	4.94E-64	0.6369901	1.890781	0.816	0.579	1.56E-59
RNF26	3.02E-49	0.6369139	1.890637	0.725	0.478	9.54E-45
FBXO5	1.96E-27	0.6367019	1.890236	0.554	0.454	6.19E-23
WAC-AS1	1.60E-73	0.6357366	1.888413	0.862	0.643	5.05E-69
DPY30	3.15E-103	0.6356424	1.888235	0.96	0.867	9.94E-99
RSBN1L	5.54E-49	0.635217	1.887432	0.669	0.426	1.75E-44
NR2F2-AS1	1.82E-55	0.6350621	1.887139	0.283	0.049	5.75E-51
LCORL	6.47E-39	0.6338162	1.88479	0.507	0.275	2.04E-34
SERTAD4-AS	7.93E-74	0.6336742	1.884522	0.366	0.07	2.50E-69
CLIC3	7.64E-21	0.6332411	1.883706	0.405	0.252	2.41E-16
CDC25B	6.91E-30	0.6332316	1.883688	0.672	0.52	2.18E-25
SSBP1	2.07E-128	0.6328909	1.883046	0.986	0.962	6.54E-124
DNAJC9	1.58E-50	0.6328784	1.883023	0.781	0.543	4.98E-46
NTMT1	4.05E-71	0.6328179	1.882909	0.921	0.784	1.28E-66

POGZ	6.07E-49	0.6319393	1.881255	0.758	0.538	1.92E-44
NCAPD3	4.65E-32	0.6314198	1.880278	0.555	0.365	1.47E-27
GLCCI1	2.84E-39	0.6307926	1.879099	0.334	0.113	8.96E-35
NAV3	3.22E-53	0.6306032	1.878744	0.816	0.602	1.02E-48
AFAP1L1	6.48E-43	0.6304888	1.878529	0.392	0.155	2.05E-38
HINT2	4.12E-57	0.6302289	1.87804	0.673	0.415	1.30E-52
ETV4	4.40E-45	0.6300579	1.877719	0.379	0.133	1.39E-40
SVBP	6.87E-62	0.6297669	1.877173	0.511	0.215	2.17E-57
BRI3	1.17E-89	0.6293026	1.876302	0.946	0.825	3.68E-85
SELENOH	2.39E-112	0.6292078	1.876124	0.966	0.908	7.53E-108
TRAPPC1	2.14E-129	0.6290398	1.875809	0.994	0.977	6.74E-125
NDUFB9	1.09E-137	0.6287515	1.875268	0.994	0.98	3.44E-133
POLR3K	2.61E-42	0.6285303	1.874853	0.671	0.501	8.23E-38
NDUFS8	2.22E-153	0.6284298	1.874665	0.998	0.995	7.01E-149
STIM2	2.56E-42	0.6284176	1.874642	0.642	0.409	8.08E-38
SENP7	1.04E-41	0.6281584	1.874156	0.462	0.219	3.28E-37
MRPL52	4.53E-99	0.6274123	1.872758	0.954	0.8	1.43E-94
PCID2	4.91E-76	0.6270453	1.872071	0.928	0.748	1.55E-71
EP400P1	8.58E-45	0.6269288	1.871853	0.447	0.191	2.71E-40
RNF146	3.86E-55	0.6267736	1.871562	0.594	0.288	1.22E-50
ATP6V1F	3.33E-97	0.6266844	1.871395	0.951	0.842	1.05E-92
ANP32A	2.07E-101	0.6266766	1.871381	0.972	0.842	6.54E-97
NDUFA11	8.34E-186	0.6266459	1.871323	0.997	0.981	2.63E-181
GNPDA1	1.85E-49	0.6264846	1.871022	0.739	0.494	5.84E-45
COMMD4	1.36E-84	0.6264234	1.870907	0.961	0.924	4.29E-80
SPA17	1.68E-52	0.6259063	1.86994	0.509	0.225	5.31E-48
PDE6D	3.12E-44	0.6252768	1.868763	0.694	0.454	9.84E-40
LAMTOR5	8.36E-142	0.6252098	1.868638	0.994	0.974	2.64E-137
OTUD6B-AS1	9.33E-64	0.6251716	1.868567	0.808	0.583	2.94E-59
RFC1	1.12E-65	0.6240793	1.866527	0.893	0.706	3.54E-61
PIM3	2.35E-42	0.6238133	1.86603	0.632	0.384	7.41E-38
USP13	1.98E-45	0.6237704	1.86595	0.698	0.45	6.25E-41
CYB5B	1.44E-123	0.623727	1.865869	0.998	0.981	4.56E-119
CAV2	1.41E-168	0.6236203	1.86567	1	1	4.45E-164
TRMT112	1.72E-170	0.6232447	1.864969	0.998	0.988	5.44E-166
ARHGAP20	9.13E-51	0.6231586	1.864809	0.197	0.014	2.88E-46
LY6E	1.48E-121	0.6227631	1.864072	0.999	0.996	4.67E-117
COX7A2	2.62E-187	0.6223636	1.863327	0.995	0.952	8.26E-183
PRAG1	5.55E-37	0.6222359	1.863089	0.452	0.225	1.75E-32
SLC20A2	4.77E-43	0.621974	1.862601	0.808	0.641	1.50E-38

CCAR1	1.12E-99	0.6217216	1.862131	0.981	0.941	3.54E-95
ACTN4	7.53E-233	0.6216169	1.861936	1	1	2.38E-228
POC5	2.20E-38	0.6206704	1.860175	0.438	0.205	6.93E-34
PLAG1	3.38E-39	0.620616	1.860073	0.325	0.108	1.07E-34
PSMB6	6.92E-186	0.6193529	1.857725	0.999	1	2.18E-181
BRCA2	6.21E-24	0.6192815	1.857593	0.407	0.313	1.96E-19
KIF18B	3.44E-24	0.619068	1.857196	0.348	0.199	1.09E-19
TOB1	5.34E-68	0.6185149	1.856169	0.888	0.685	1.68E-63
C16orf74	5.00E-53	0.6181788	1.855546	0.29	0.061	1.58E-48
KIF26B	3.02E-23	0.6181583	1.855508	0.291	0.128	9.53E-19
ZNHIT1	6.37E-100	0.6168833	1.853143	0.966	0.902	2.01E-95
VPS33A	4.34E-50	0.6167553	1.852906	0.657	0.39	1.37E-45
DSN1	2.99E-34	0.6164749	1.852387	0.482	0.27	9.43E-30
ATP5MC3	2.53E-237	0.6162995	1.852062	1	1	7.99E-233
PSG1	5.38E-42	0.6162809	1.852027	0.152	0.007	1.70E-37
COX4I1	2.66E-300	0.6159383	1.851393	1	1	8.39E-296
LAMTOR4	1.28E-86	0.6153675	1.850336	0.91	0.759	4.03E-82
ATP23	4.90E-30	0.6149006	1.849473	0.383	0.205	1.55E-25
RANGRF	3.86E-44	0.6148741	1.849424	0.765	0.591	1.22E-39
RIPOR2	3.72E-28	0.6148387	1.849358	0.281	0.129	1.17E-23
TTC8	1.12E-52	0.6146862	1.849076	0.674	0.394	3.55E-48
BCL7C	4.97E-78	0.6143434	1.848443	0.916	0.781	1.57E-73
ATOH8	3.09E-58	0.6142336	1.848239	0.477	0.174	9.74E-54
BLOC1S1	2.89E-91	0.6138809	1.847588	0.878	0.653	9.11E-87
ATP5IF1	2.67E-119	0.6137104	1.847273	0.978	0.848	8.43E-115
AOX1	9.28E-44	0.6135846	1.84704	0.593	0.33	2.93E-39
MAP3K20	1.82E-128	0.6134346	1.846763	0.999	0.993	5.75E-124
DRAP1	4.83E-168	0.6131696	1.846274	1	0.999	1.52E-163
FAM110A	1.98E-40	0.6130744	1.846098	0.318	0.106	6.23E-36
HP1BP3	5.80E-173	0.6120023	1.84412	1	0.996	1.83E-168
C11orf68	8.65E-64	0.6115311	1.843251	0.844	0.6	2.73E-59
VRK2	7.95E-44	0.6114127	1.843033	0.646	0.404	2.51E-39
SNRPG	1.47E-118	0.6113122	1.842848	0.99	0.954	4.65E-114
ARL6IP1	1.56E-13	0.610672	1.841669	0.993	0.993	4.92E-09
KIAA0586	2.62E-41	0.6104132	1.841192	0.647	0.422	8.26E-37
AMPD3	4.27E-48	0.6103056	1.840994	0.283	0.062	1.35E-43
TIMM17B	3.08E-56	0.6102199	1.840836	0.84	0.703	9.70E-52
YEATS4	5.61E-39	0.6100429	1.84051	0.561	0.339	1.77E-34
COMMD7	3.26E-73	0.6100162	1.840461	0.945	0.799	1.03E-68
CRIM1-DT	4.40E-56	0.6096974	1.839875	0.433	0.152	1.39E-51

WDR83OS	4.01E-152	0.6095726	1.839645	0.994	0.958	1.27E-147
DEPDC1B	8.02E-27	0.6095052	1.839521	0.26	0.103	2.53E-22
GABBR2	6.00E-15	0.6091005	1.838777	0.168	0.091	1.89E-10
RPS27L	1.27E-150	0.6090969	1.83877	1	1	4.02E-146
POLR2J	1.16E-93	0.6087982	1.838221	0.926	0.765	3.67E-89
CU633906.1	3.28E-113	0.6087562	1.838144	0.51	0.1	1.03E-108
NUPR1	2.94E-45	0.6087013	1.838043	0.873	0.935	9.26E-41
TMEM106B	1.40E-59	0.6085094	1.83769	0.971	0.93	4.42E-55
LBHD1	1.59E-68	0.6071954	1.835277	0.733	0.463	5.02E-64
MYEF2	1.29E-38	0.6066622	1.834299	0.457	0.218	4.08E-34
RPL36AL	1.52E-181	0.6055644	1.832286	0.999	0.995	4.80E-177
MRPL57	8.65E-74	0.6053993	1.831984	0.877	0.689	2.73E-69
EXOSC9	3.51E-55	0.6049156	1.831098	0.866	0.67	1.11E-50
DYNLL1	3.14E-194	0.604546	1.830421	1	1	9.90E-190
FANCD2	3.33E-36	0.6044376	1.830223	0.539	0.306	1.05E-31
CNIH4	4.02E-80	0.6044358	1.830219	0.961	0.902	1.27E-75
C2orf74	3.66E-53	0.6035765	1.828647	0.436	0.159	1.15E-48
SP3	7.47E-75	0.6030998	1.827776	0.897	0.667	2.36E-70
JUN	3.61E-81	0.6028663	1.827349	0.985	0.964	1.14E-76
PGPEP1	3.96E-39	0.6028166	1.827258	0.53	0.303	1.25E-34
CENPN	6.51E-40	0.6011283	1.824176	0.731	0.559	2.05E-35
MCTP1	6.41E-40	0.600282	1.822633	0.19	0.025	2.02E-35
UBTF	3.03E-78	0.6001412	1.822376	0.944	0.805	9.55E-74
AHNAK2	5.14E-43	0.59995	1.822028	0.551	0.285	1.62E-38
ETS2	2.79E-51	0.599839	1.821825	0.825	0.613	8.80E-47
HUNK	2.54E-36	0.5998015	1.821757	0.355	0.167	8.00E-32
MT1L	3.97E-76	0.5989792	1.82026	0.59	0.248	1.25E-71
AP000356.5	5.64E-92	0.5989289	1.820168	0.878	0.704	1.78E-87
SLF2	1.17E-45	0.5988731	1.820067	0.648	0.393	3.68E-41
ASF1B	2.91E-28	0.598469	1.819331	0.442	0.241	9.17E-24
METTL4	1.28E-37	0.5984402	1.819279	0.497	0.261	4.03E-33
NFU1	1.15E-65	0.5977673	1.818055	0.919	0.788	3.64E-61
HPS3	3.19E-47	0.5977654	1.818052	0.679	0.418	1.01E-42
S100A3	9.54E-37	0.5977194	1.817968	0.431	0.216	3.01E-32
NAP1L4	3.74E-101	0.597698	1.817929	0.982	0.881	1.18E-96
BRD8	9.84E-47	0.5968096	1.816315	0.654	0.388	3.10E-42
ANP32E	2.94E-58	0.5967309	1.816172	0.966	0.928	9.27E-54
EFHD1	1.12E-24	0.5964867	1.815728	0.242	0.088	3.54E-20
PFN1	0.00E+00	0.596246	1.815291	1	1	0.00E+00
MB21D2	3.61E-45	0.5948053	1.812678	0.58	0.308	1.14E-40

TULP3	9.46E-56	0.5943274	1.811812	0.861	0.698	2.98E-51
ARMC1	1.02E-77	0.5942325	1.81164	0.929	0.751	3.23E-73
SWAP70	1.83E-70	0.5941338	1.811461	0.926	0.752	5.77E-66
VEZF1	1.44E-88	0.5941258	1.811447	0.978	0.908	4.53E-84
EXOC1	3.26E-65	0.5939996	1.811218	0.883	0.682	1.03E-60
TMEM158	2.49E-10	0.5939612	1.811148	0.536	0.548	7.85E-06
HNRNPR	8.26E-128	0.5925172	1.808535	1	1	2.60E-123
POLR2F	1.15E-99	0.5919577	1.807524	0.953	0.845	3.64E-95
CU633967.1	5.34E-88	0.5918972	1.807414	0.623	0.237	1.69E-83
UQCR10	6.21E-108	0.591364	1.806451	0.977	0.887	1.96E-103
TERF1	7.48E-63	0.5912514	1.806247	0.924	0.809	2.36E-58
GNB1L	4.24E-45	0.5908814	1.805579	0.415	0.161	1.34E-40
FBLN2	6.56E-52	0.5908586	1.805538	0.703	0.415	2.07E-47
TIPARP	3.50E-46	0.5906826	1.80522	0.833	0.658	1.10E-41
PARD3B	1.72E-33	0.5899107	1.803827	0.4	0.182	5.43E-29
MRPL14	8.30E-77	0.5891622	1.802478	0.961	0.897	2.62E-72
FCGRT	2.25E-40	0.5888815	1.801972	0.768	0.595	7.10E-36
RASGRF2	1.45E-43	0.5883966	1.801098	0.182	0.019	4.59E-39
GCLC	2.36E-42	0.588241	1.800818	0.656	0.41	7.44E-38
EEA1	6.23E-84	0.5882194	1.800779	0.983	0.946	1.97E-79
MRPS36	5.49E-78	0.5880898	1.800546	0.818	0.543	1.73E-73
MT-RNR2	0.00E+00	0.5880477	1.80047	1	1	0.00E+00
WDHD1	2.85E-28	0.5876093	1.799681	0.436	0.233	8.98E-24
C12orf57	7.13E-92	0.5873888	1.799284	0.961	0.881	2.25E-87
THBS1	3.89E-42	0.5873033	1.79913	1	1	1.23E-37
RPS27A	0.00E+00	0.5870357	1.798649	1	1	0.00E+00
IFI27L2	2.81E-79	0.5866577	1.797969	0.794	0.496	8.86E-75
CDK19	1.96E-38	0.5861365	1.797032	0.672	0.444	6.18E-34
E2F7	1.17E-26	0.5858368	1.796494	0.515	0.348	3.71E-22
AKNA	2.33E-39	0.5857344	1.79631	0.343	0.143	7.35E-35
WDR41	7.77E-58	0.5857016	1.796251	0.901	0.761	2.45E-53
LPAR6	3.87E-38	0.5853564	1.795631	0.289	0.088	1.22E-33
IFFO1	1.87E-48	0.5853526	1.795624	0.619	0.343	5.89E-44
C15orf61	1.96E-48	0.5852574	1.795453	0.528	0.273	6.19E-44
NXPE3	1.77E-47	0.5851962	1.795343	0.757	0.532	5.58E-43
H3P6	7.38E-127	0.5851508	1.795262	0.892	0.595	2.33E-122
CASK	3.99E-49	0.5851485	1.795258	0.818	0.615	1.26E-44
SYT16	2.42E-36	0.5848417	1.794707	0.232	0.056	7.65E-32
PDE4B	2.69E-40	0.5843527	1.793829	0.444	0.195	8.48E-36
ZNF521	6.03E-32	0.5838181	1.792871	0.272	0.091	1.90E-27

SCAND1	1.76E-74	0.5833267	1.79199	0.945	0.872	5.54E-70
IQGAP3	2.67E-33	0.5832033	1.791769	0.414	0.209	8.42E-29
TPM2	1.15E-146	0.5831681	1.791706	1	1	3.62E-142
NDUFB8	2.47E-126	0.5829968	1.791399	0.995	0.983	7.79E-122
ZNRD2	5.70E-65	0.5829714	1.791353	0.855	0.709	1.80E-60
ANKRD39	9.75E-33	0.5826338	1.790749	0.527	0.343	3.08E-28
LRRC61	2.09E-57	0.5826213	1.790726	0.243	0.025	6.58E-53
LYPD6	1.10E-42	0.5826039	1.790695	0.233	0.042	3.47E-38
DOCK11	9.21E-40	0.582504	1.790516	0.575	0.321	2.91E-35
DDI2	5.33E-42	0.5819667	1.789555	0.648	0.398	1.68E-37
NIF3L1	1.66E-35	0.581833	1.789315	0.581	0.35	5.22E-31
GDF15	2.46E-11	0.5817305	1.789132	0.381	0.26	7.75E-07
SYNRG	4.29E-51	0.5817064	1.789089	0.697	0.43	1.35E-46
PMS1	2.87E-41	0.5817045	1.789085	0.565	0.308	9.04E-37
EXTL1	2.21E-52	0.5811472	1.788089	0.205	0.017	6.98E-48
MIPEP	1.87E-35	0.5811281	1.788054	0.553	0.32	5.91E-31
SMS	3.02E-111	0.5809289	1.787698	0.996	0.976	9.53E-107
GPC1	6.65E-113	0.5807899	1.78745	0.994	0.97	2.10E-108
NDUFA8	1.53E-77	0.5807568	1.787391	0.97	0.935	4.82E-73
HIGD2A	1.19E-85	0.5805471	1.787016	0.939	0.866	3.76E-81
ETNK2	2.62E-43	0.5804533	1.786848	0.282	0.068	8.26E-39
PRR14	5.14E-41	0.5804455	1.786834	0.581	0.337	1.62E-36
EDA2R	1.88E-42	0.5802926	1.786561	0.501	0.24	5.92E-38
KRTAP1-5	2.03E-13	0.5800442	1.786117	0.358	0.224	6.42E-09
NDUFAF8	6.50E-92	0.5798982	1.785857	0.924	0.755	2.05E-87
SHMT1	8.33E-31	0.5798205	1.785718	0.453	0.242	2.63E-26
STRA6	3.86E-36	0.5797167	1.785532	0.512	0.275	1.22E-31
RUSC1	3.94E-50	0.5794726	1.785097	0.741	0.484	1.24E-45
SLC15A3	7.52E-37	0.5789359	1.784139	0.311	0.104	2.37E-32
MBOAT7	7.62E-98	0.5783957	1.783175	0.988	0.964	2.40E-93
PHF11	7.61E-61	0.5782423	1.782902	0.764	0.476	2.40E-56
RNF7	1.28E-103	0.5782129	1.782849	0.998	0.987	4.03E-99
LYRM2	4.79E-57	0.5778428	1.78219	0.887	0.752	1.51E-52
TOR3A	2.64E-41	0.5777473	1.78202	0.631	0.391	8.32E-37
PBX2	4.91E-44	0.5766328	1.780035	0.728	0.51	1.55E-39
SIVA1	7.28E-69	0.5766196	1.780011	0.945	0.9	2.30E-64
LINC00643	9.15E-48	0.5760486	1.778995	0.138	0	2.89E-43
RP9	1.27E-51	0.5759266	1.778778	0.62	0.359	4.00E-47
SPATS2	6.74E-53	0.5756067	1.778209	0.858	0.695	2.13E-48
AURKAIP1	3.43E-107	0.575582	1.778165	0.995	0.986	1.08E-102

COX5A	9.11E-106	0.5755444	1.778098	0.992	0.976	2.87E-101
CU634019.1	9.69E-109	0.575094	1.777298	0.496	0.095	3.06E-104
RAD17	5.52E-47	0.5748842	1.776925	0.648	0.375	1.74E-42
NT5E	3.95E-144	0.5747005	1.776598	1	0.994	1.24E-139
TM2D3	1.26E-48	0.574662	1.77653	0.719	0.456	3.98E-44
PKN2	7.88E-63	0.5745041	1.776249	0.896	0.716	2.49E-58
WWC3	3.73E-46	0.5740961	1.775525	0.832	0.637	1.18E-41
OCIAD2	5.64E-38	0.5738398	1.77507	0.745	0.589	1.78E-33
TCTEX1D2	2.20E-46	0.5733453	1.774192	0.513	0.249	6.94E-42
DMAC1	8.96E-58	0.5726027	1.772875	0.815	0.604	2.83E-53
ZNF75A	1.02E-31	0.5724241	1.772559	0.488	0.273	3.22E-27
CU633904.1	1.26E-101	0.5723387	1.772407	0.505	0.114	3.97E-97
MRE11	1.81E-33	0.5721083	1.771999	0.609	0.405	5.72E-29
SAP30	1.74E-36	0.5719908	1.771791	0.597	0.415	5.50E-32
ACTB	1.17E-298	0.5715607	1.771029	1	1	3.68E-294
CLIP4	4.28E-49	0.5715602	1.771028	0.68	0.408	1.35E-44
CEP44	7.32E-29	0.5711598	1.770319	0.48	0.284	2.31E-24
EXOSC1	1.61E-42	0.5709996	1.770036	0.814	0.67	5.09E-38
GNAI1	1.40E-50	0.5709257	1.769905	0.901	0.796	4.40E-46
TRIM6	2.69E-39	0.5697744	1.767868	0.454	0.215	8.50E-35
MRPL55	5.96E-52	0.5696627	1.767671	0.785	0.596	1.88E-47
MRPS28	5.61E-57	0.5695692	1.767506	0.828	0.665	1.77E-52
MYO18A	2.07E-42	0.5694977	1.767379	0.577	0.315	6.53E-38
FANCB	7.64E-39	0.5693336	1.767089	0.304	0.1	2.41E-34
REV3L	1.33E-49	0.5691742	1.766807	0.847	0.669	4.21E-45
KLHL22	5.91E-40	0.5690504	1.766589	0.387	0.15	1.86E-35
C18orf54	1.48E-31	0.5690414	1.766573	0.336	0.135	4.68E-27
CEBPD	2.66E-36	0.5689963	1.766493	0.574	0.354	8.38E-32
DAD1	5.93E-216	0.5684005	1.765441	1	1	1.87E-211
NUP107	2.88E-41	0.568248	1.765172	0.749	0.538	9.09E-37
RRM1	2.89E-60	0.5682443	1.765165	0.911	0.771	9.10E-56
TMEM223	2.58E-34	0.5681278	1.76496	0.563	0.357	8.15E-30
DGUOK	6.98E-76	0.5679215	1.764595	0.951	0.845	2.20E-71
PRSS12	8.26E-31	0.5677943	1.764371	0.619	0.412	2.61E-26
SMC3	1.24E-67	0.5674834	1.763823	0.975	0.888	3.92E-63
C2CD5	6.57E-43	0.5674247	1.763719	0.547	0.278	2.07E-38
FDFT1	1.02E-63	0.5671921	1.763309	0.969	0.899	3.23E-59
TIMM13	9.33E-80	0.5670784	1.763108	0.976	0.948	2.94E-75
AC023055.1	6.32E-89	0.5665148	1.762115	0.872	0.613	1.99E-84
APPL2	2.88E-81	0.5665124	1.762111	0.966	0.859	9.08E-77

MRPS18C	1.40E-67	0.5664585	1.762016	0.876	0.673	4.42E-63
MCM4	1.35E-37	0.5663643	1.76185	0.778	0.603	4.26E-33
SERPINB1	2.70E-49	0.5661217	1.761423	0.834	0.669	8.51E-45
CTCF	1.52E-54	0.5652742	1.75993	0.862	0.683	4.81E-50
COX5B	9.98E-144	0.5649638	1.759384	0.992	0.962	3.15E-139
UBE2L6	3.25E-37	0.5648964	1.759265	0.855	0.718	1.03E-32
PSMA5	1.87E-151	0.5648272	1.759144	1	1	5.90E-147
ASXL1	1.82E-46	0.564474	1.758523	0.733	0.492	5.74E-42
TMSB4XP1	1.19E-132	0.5642533	1.758135	0.674	0.213	3.74E-128
MRPL13	9.44E-77	0.5642159	1.758069	0.977	0.946	2.98E-72
POLA2	8.94E-28	0.5640463	1.757771	0.448	0.259	2.82E-23
PRIM1	1.15E-21	0.5639895	1.757671	0.337	0.195	3.64E-17
COA3	2.43E-59	0.563178	1.756245	0.918	0.863	7.68E-55
H2AZ2	1.38E-88	0.5621533	1.754446	0.998	0.996	4.35E-84
RPL18	0.00E+00	0.5621371	1.754418	1	1	0.00E+00
ZNF280D	4.16E-40	0.5620837	1.754324	0.732	0.505	1.31E-35
SCNM1	3.15E-42	0.5620365	1.754241	0.813	0.657	9.94E-38
SMAD2	7.16E-70	0.5611298	1.752651	0.96	0.878	2.26E-65
ANAPC10	1.38E-32	0.5607293	1.75195	0.573	0.367	4.35E-28
AC093849.2	1.06E-42	0.5601923	1.751009	0.278	0.073	3.33E-38
USP4	5.87E-54	0.5600581	1.750774	0.779	0.526	1.85E-49
NISCH	1.30E-31	0.5594465	1.749704	0.662	0.492	4.10E-27
UHRF1	2.07E-36	0.5590528	1.749015	0.647	0.429	6.52E-32
SYDE2	2.59E-29	0.5587824	1.748542	0.374	0.185	8.16E-25
DHRS4L2	1.97E-38	0.5585798	1.748188	0.802	0.668	6.22E-34
CBX5	2.39E-69	0.5584807	1.748015	0.979	0.926	7.54E-65
ATP5PD	8.71E-109	0.5584282	1.747923	0.994	0.963	2.75E-104
BORA	6.67E-22	0.5582033	1.74753	0.268	0.124	2.10E-17
APOBEC3A	1.59E-47	0.5580079	1.747189	0.515	0.241	5.02E-43
PGD	2.57E-96	0.5579621	1.747108	0.996	0.987	8.09E-92
TMEM187	1.27E-38	0.5575706	1.746425	0.279	0.077	3.99E-34
SYNPO2L	2E-38	0.5568115	1.745099	0.267	0.08	6.32E-34
RPS14	0.00E+00	0.5564259	1.744427	1	1	0.00E+00
PEX11B	2.12E-39	0.5562144	1.744058	0.587	0.331	6.69E-35
NME4	1.86E-80	0.5558709	1.743459	0.988	0.972	5.86E-76
FANCG	3.36E-28	0.5557799	1.7433	0.385	0.187	1.06E-23
PSENEN	8.79E-75	0.5556527	1.743078	0.909	0.773	2.77E-70
THSD8	3.03E-50	0.5556058	1.742997	0.612	0.339	9.57E-46
VPS72	5.64E-58	0.5549643	1.741879	0.789	0.514	1.78E-53
INCENP	1.88E-23	0.5549089	1.741782	0.286	0.141	5.93E-19

MYL12A	5.66E-254	0.5547306	1.741472	1	1	1.79E-249
CWC15	7.41E-71	0.5546536	1.741338	0.877	0.646	2.34E-66
PELO	1.14E-52	0.5546487	1.741329	0.964	0.92	3.61E-48
RANBP6	1.80E-41	0.5545966	1.741238	0.645	0.393	5.69E-37
PRKDC	4.92E-80	0.5545736	1.741198	0.984	0.945	1.55E-75
ATP8B3	8.25E-32	0.5541668	1.74049	0.288	0.108	2.60E-27
RANBP1	1.52E-94	0.5541239	1.740416	0.998	0.993	4.81E-90
RPS16	0.00E+00	0.5541148	1.7404	1	1	0.00E+00
AGAP3	1.16E-46	0.5537913	1.739837	0.719	0.466	3.65E-42
ALYREF	8.66E-48	0.5527363	1.738002	0.923	0.847	2.73E-43
TMEM14C	1.64E-137	0.5527285	1.737989	1	0.993	5.16E-133
SALL1	2.37E-39	0.5525378	1.737657	0.469	0.217	7.48E-35
DCTN3	7.20E-83	0.5523041	1.737251	0.99	0.971	2.27E-78
NARS2	1.01E-44	0.5523019	1.737247	0.524	0.251	3.18E-40
CUL4B	3.47E-46	0.5522305	1.737123	0.964	0.933	1.09E-41
RAMP1	3.99E-40	0.5521698	1.737018	0.179	0.02	1.26E-35
PDS5B	2.57E-47	0.5520599	1.736827	0.726	0.469	8.09E-43
CAV1	7.60E-164	0.5518709	1.736499	1	1	2.40E-159
DBN1	1.99E-96	0.5512789	1.735471	0.992	0.971	6.28E-92
GTF2IRD1	7.00E-36	0.5511524	1.735252	0.566	0.335	2.21E-31
DAP	2.07E-126	0.5510544	1.735081	1	0.99	6.52E-122
SLC25A24	8.15E-59	0.551019	1.73502	0.923	0.788	2.57E-54
RPL27	0.00E+00	0.5510186	1.735019	1	1	0.00E+00
ATP5PF	8.58E-124	0.5509958	1.73498	0.992	0.968	2.71E-119
LSM4	2.57E-97	0.5504116	1.733966	0.995	0.97	8.11E-93
NUDCD1	8.59E-54	0.5503882	1.733926	0.882	0.698	2.71E-49
TNFRSF11B	1.45E-19	0.5501587	1.733528	0.712	0.556	4.57E-15
RNASEH2C	8.58E-53	0.5496446	1.732637	0.801	0.635	2.71E-48
FRMD4A	1.56E-51	0.549615	1.732586	0.893	0.724	4.92E-47
EVA1C	9.41E-33	0.5487434	1.731076	0.326	0.129	2.97E-28
EHMT2	1.49E-40	0.5487105	1.731019	0.633	0.391	4.69E-36
CUL2	5.06E-61	0.5482198	1.73017	0.901	0.721	1.60E-56
NDUFB7	1.92E-96	0.5481926	1.730123	0.961	0.859	6.04E-92
DEF8	4.49E-51	0.5478126	1.729466	0.884	0.769	1.42E-46
GARRE1	2.28E-26	0.5476842	1.729244	0.728	0.592	7.19E-22
SEC11C	6.69E-43	0.5474811	1.728893	0.76	0.564	2.11E-38
SMARCE1	5.78E-130	0.5474144	1.728777	0.999	0.993	1.82E-125
NUP37	1.25E-43	0.5469994	1.72806	0.804	0.583	3.96E-39
RHNO1	1.13E-38	0.5464266	1.72707	0.61	0.359	3.57E-34
NR2F1-AS1	3.97E-28	0.5459274	1.726208	0.685	0.556	1.25E-23

KCTD20	5.60E-86	0.5458877	1.72614	0.975	0.878	1.77E-81
HYLS1	1.70E-22	0.5458653	1.726101	0.418	0.255	5.36E-18
MCTS1	3.22E-73	0.5455345	1.72553	0.971	0.932	1.01E-68
CISD3	1.04E-48	0.5454276	1.725346	0.807	0.668	3.27E-44
NDUFA7	9.99E-69	0.5453465	1.725206	0.82	0.571	3.15E-64
COA6	2.25E-47	0.5452578	1.725053	0.739	0.537	7.08E-43
GPSM2	2.47E-21	0.545194	1.724943	0.675	0.582	7.78E-17
MYH10	8.90E-35	0.5450991	1.724779	0.914	0.814	2.81E-30
TMEM106C	2.67E-48	0.5450204	1.724644	0.847	0.652	8.42E-44
COPS6	6.05E-147	0.5448465	1.724344	1	0.998	1.91E-142
HS3ST3A1	4.85E-20	0.5446311	1.723972	0.541	0.43	1.53E-15
RHBDD1	8.11E-32	0.5445909	1.723903	0.61	0.406	2.56E-27
ADAM19	1.20E-41	0.5444827	1.723716	0.886	0.805	3.80E-37
AC087392.1	2.02E-55	0.5442649	1.723341	0.23	0.024	6.36E-51
TTC1	4.07E-70	0.5438385	1.722606	0.951	0.839	1.28E-65
C9orf16	1.17E-51	0.543507	1.722035	0.757	0.553	3.70E-47
TMCO4	1.59E-32	0.5434932	1.722012	0.429	0.211	5.02E-28
NSD2	1.09E-34	0.5433918	1.721837	0.821	0.709	3.42E-30
RPLP1	0.00E+00	0.5431808	1.721474	1	1	0.00E+00
NEMP1	1.51E-24	0.5430981	1.721331	0.374	0.199	4.78E-20
SASS6	1.07E-29	0.5429014	1.720993	0.295	0.115	3.38E-25
NDUFA13	5.71E-117	0.5426673	1.72059	0.973	0.902	1.80E-112
PLPBP	3.08E-44	0.5422214	1.719823	0.807	0.602	9.71E-40
CABYR	8.77E-44	0.5410735	1.71785	0.248	0.048	2.77E-39
PFDN4	6.40E-64	0.5408882	1.717532	0.941	0.849	2.02E-59
MPHOSPH6	8.17E-43	0.5408818	1.717521	0.745	0.534	2.58E-38
LONRF1	1.71E-30	0.5407761	1.717339	0.38	0.176	5.39E-26
ACIN1	3.71E-80	0.5407372	1.717272	0.976	0.939	1.17E-75
LYSMD2	1.87E-27	0.540468	1.71681	0.487	0.291	5.91E-23
PHIP	7.15E-39	0.5404516	1.716782	0.8	0.639	2.26E-34
PAK1	1.74E-45	0.540387	1.716671	0.693	0.43	5.48E-41
CDC42BPA	4.07E-44	0.5400721	1.716131	0.928	0.874	1.28E-39
HMGN3	1.11E-53	0.5399387	1.715902	0.901	0.764	3.49E-49
DNAJC21	7.61E-71	0.5390773	1.714424	0.954	0.856	2.40E-66
VPS45	1.03E-40	0.5385556	1.71353	0.716	0.487	3.24E-36
CCDC58	9.82E-39	0.538528	1.713483	0.623	0.421	3.10E-34
RANBP17	4.52E-35	0.5384782	1.713397	0.22	0.05	1.43E-30
TYMS	1.13E-52	0.538131	1.712803	0.781	0.549	3.56E-48
PFDN2	9.33E-82	0.5380586	1.712679	0.983	0.952	2.94E-77
NDUFV2	3.46E-97	0.5378382	1.712301	0.994	0.974	1.09E-92

METTL14	4.10E-39	0.5376233	1.711933	0.646	0.409	1.29E-34
ARMC4	9.51E-43	0.5375473	1.711803	0.168	0.012	3.00E-38
LAMA1	6.80E-27	0.5372245	1.711251	0.272	0.104	2.14E-22
PHLDA1	1.63E-61	0.5364966	1.710006	0.995	0.984	5.13E-57
ZSWIM7	8.82E-32	0.5364682	1.709957	0.607	0.417	2.78E-27
NSA2	3.74E-101	0.5356695	1.708592	0.991	0.98	1.18E-96
ERI1	4.34E-32	0.5356562	1.708569	0.536	0.315	1.37E-27
TBC1D14	3.93E-33	0.5355903	1.708456	0.611	0.384	1.24E-28
SAPCD2	1.34E-31	0.5353147	1.707986	0.27	0.09	4.23E-27
ESYT1	2.86E-70	0.5348603	1.70721	0.978	0.941	9.01E-66
EMC9	2.90E-24	0.5347846	1.707081	0.588	0.456	9.13E-20
LSM8	4.42E-55	0.5346647	1.706876	0.872	0.718	1.39E-50
CRYZ	8.46E-40	0.5346617	1.706871	0.754	0.534	2.67E-35
GTF2E1	4.79E-34	0.5346243	1.706807	0.498	0.263	1.51E-29
MRPS10	9.15E-88	0.534611	1.706784	0.991	0.954	2.88E-83
GEMIN6	1.66E-39	0.5345479	1.706677	0.64	0.417	5.25E-35
ELAC1	7.63E-31	0.5336346	1.705118	0.402	0.205	2.41E-26
ATP5MF	4.81E-111	0.5335499	1.704974	0.981	0.881	1.52E-106
EMILIN2	1.03E-29	0.5334646	1.704829	0.521	0.308	3.24E-25
DIPK1A	1.85E-46	0.5334233	1.704758	0.853	0.667	5.83E-42
TIGD2	1.22E-35	0.5333883	1.704699	0.267	0.077	3.86E-31
ORMDL2	2.05E-55	0.5333066	1.704559	0.856	0.686	6.46E-51
GYG1	5.28E-56	0.5332757	1.704507	0.944	0.812	1.67E-51
ADK	5.36E-63	0.5326931	1.703514	0.981	0.934	1.69E-58
S1PR3	2.18E-29	0.5325484	1.703267	0.859	0.741	6.86E-25
AC024592.3	3.69E-97	0.5324507	1.703101	0.794	0.457	1.16E-92
CALM1	3.69E-206	0.5321863	1.702651	1	1	1.16E-201
MAP3K12	1.27E-37	0.5321622	1.70261	0.504	0.289	3.99E-33
STMN3	2.65E-38	0.5319325	1.702219	0.442	0.198	8.37E-34
HMGA2	1.66E-55	0.531866	1.702105	0.878	0.712	5.23E-51
MLLT10	6.44E-36	0.53185	1.702078	0.611	0.378	2.03E-31
EPS8L2	1.62E-28	0.5318354	1.702053	0.464	0.263	5.10E-24
WNT2	9.33E-18	0.5316193	1.701686	0.16	0.053	2.94E-13
ANAPC7	6.82E-44	0.5315436	1.701557	0.778	0.568	2.15E-39
LIG1	6.30E-34	0.5314667	1.701426	0.504	0.267	1.99E-29
MRPS34	1.84E-73	0.5314337	1.70137	0.976	0.9	5.79E-69
H4C3	4.73E-42	0.5311056	1.700812	0.36	0.134	1.49E-37
OXLD1	7.67E-35	0.5308264	1.700337	0.477	0.252	2.42E-30
TMEM51	2.03E-30	0.5306408	1.700021	0.325	0.131	6.40E-26
BDH2	2.28E-26	0.530479	1.699746	0.742	0.596	7.19E-22

TST	2.30E-28	0.5302581	1.699371	0.672	0.487	7.25E-24
LIN9	6.65E-24	0.5301742	1.699228	0.304	0.145	2.10E-19
TUBB4B	4.71E-31	0.530045	1.699009	1	1	1.49E-26
GIT2	6.40E-42	0.5298017	1.698595	0.754	0.524	2.02E-37
LARP6	1.10E-67	0.5296587	1.698352	0.937	0.797	3.48E-63
EPOR	2.80E-34	0.5294002	1.697914	0.253	0.071	8.83E-30
MYL6B	2.89E-39	0.52928	1.69771	0.753	0.613	9.13E-35
ENDOG	3.13E-27	0.5292789	1.697708	0.47	0.299	9.88E-23
PIP4K2B	1.10E-37	0.5288914	1.69705	0.722	0.506	3.46E-33
EPS8	4.94E-55	0.5286488	1.696638	0.994	0.988	1.56E-50
ABHD12	1.00E-53	0.528535	1.696445	0.884	0.705	3.15E-49
COX15	1.50E-34	0.5285257	1.696429	0.674	0.452	4.74E-30
THOC7	1.16E-68	0.5278544	1.695291	0.979	0.926	3.66E-64
NSD1	8.97E-37	0.5277801	1.695165	0.689	0.47	2.83E-32
RP9P	4.09E-34	0.5274914	1.694676	0.541	0.332	1.29E-29
EIF2AK4	8.79E-67	0.5274665	1.694634	0.957	0.867	2.77E-62
SCARB1	1.26E-35	0.5273422	1.694423	0.463	0.229	3.98E-31
GGA3	2.63E-32	0.5263323	1.692712	0.497	0.273	8.28E-28
RRM2	2.63E-37	0.5262031	1.692494	0.763	0.658	8.31E-33
TK1	4.36E-62	0.5258236	1.691852	0.933	0.855	1.38E-57
CDCA5	1.67E-21	0.5253779	1.691098	0.406	0.227	5.28E-17
ARMCX6	1.52E-48	0.5247197	1.689985	0.868	0.725	4.78E-44
ABCC3	1.89E-29	0.5245272	1.68966	0.712	0.531	5.97E-25
TMSB15B	1.19E-31	0.5243012	1.689278	0.499	0.305	3.74E-27
CKLF	1.32E-46	0.5240736	1.688894	0.694	0.459	4.15E-42
PLEKHH3	5.11E-45	0.5237215	1.688299	0.444	0.198	1.61E-40
TGFBRAP1	7.18E-29	0.5235653	1.688035	0.638	0.456	2.27E-24
S100A6	3.90E-256	0.5233428	1.68766	1	1	1.23E-251
EXOC6	5.12E-29	0.5228457	1.686821	0.271	0.097	1.61E-24
RIPK2	1.24E-46	0.5228254	1.686787	0.803	0.577	3.90E-42
TKT	9.37E-108	0.5224797	1.686204	1	1	2.96E-103
KAT2B	4.34E-28	0.5217465	1.684968	0.416	0.216	1.37E-23
GRK5	1.79E-22	0.5216694	1.684838	0.407	0.236	5.64E-18
GPSM3	5.22E-37	0.521589	1.684703	0.298	0.095	1.65E-32
DDX39A	7.80E-40	0.5215379	1.684616	0.892	0.814	2.46E-35
MYL12B	5.66E-273	0.5214432	1.684457	1	1	1.79E-268
BCL2L1	1.04E-53	0.5211735	1.684003	0.773	0.508	3.29E-49
CIAO2A	3.08E-47	0.5211576	1.683976	0.906	0.783	9.70E-43
ZFAND6	5.06E-70	0.5210699	1.683828	0.975	0.879	1.59E-65
FUT8	2.29E-42	0.5209157	1.683569	0.869	0.755	7.24E-38

GADD45GIP1	2.67E-85	0.5209148	1.683567	0.995	0.98	8.42E-81
RAI1	1.87E-34	0.5204054	1.68271	0.592	0.368	5.90E-30
KRTCAP2	3E-90	0.5198221	1.681728	0.961	0.882	9.45E-86
RPS7	0.00E+00	0.5198217	1.681728	1	1	0.00E+00
RGL1	1.51E-31	0.5197497	1.681607	0.532	0.305	4.75E-27
RNF181	1.53E-77	0.5196066	1.681366	0.957	0.876	4.82E-73
VAMP5	2.51E-46	0.5192291	1.680731	0.849	0.752	7.92E-42
AL133352.1	3.80E-85	0.5191201	1.680548	0.87	0.665	1.20E-80
BAIAP2L2	5.07E-27	0.5190276	1.680393	0.195	0.054	1.60E-22
PI4KB	1.19E-40	0.5188696	1.680127	0.839	0.674	3.76E-36
SUMO2	6.97E-196	0.5181789	1.678967	1	1	2.20E-191
ZNF692	1.81E-21	0.518156	1.678929	0.322	0.161	5.70E-17
HTATSF1	8.37E-54	0.5180271	1.678713	0.919	0.815	2.64E-49
SOGA1	9.42E-31	0.5180081	1.678681	0.635	0.44	2.97E-26
LCOR	3.38E-39	0.517997	1.678662	0.66	0.412	1.07E-34
PDCD1LG2	1.97E-27	0.5179451	1.678575	0.626	0.44	6.21E-23
FKBP2	1.47E-61	0.5178044	1.678339	0.847	0.659	4.64E-57
H2AJ	6.69E-57	0.5176956	1.678156	0.9	0.813	2.11E-52
MCM8	4.62E-27	0.5174999	1.677828	0.509	0.326	1.46E-22
SSBP4	7.26E-53	0.5174518	1.677747	0.865	0.664	2.29E-48
MRPS16	6.14E-66	0.5174037	1.677666	0.966	0.923	1.94E-61
DZIP3	5.00E-28	0.5170832	1.677129	0.53	0.323	1.58E-23
SMARCAL1	4.29E-31	0.5169547	1.676913	0.519	0.295	1.35E-26
ARRB1	8.76E-29	0.5165141	1.676174	0.366	0.167	2.76E-24
MZT2A	3.67E-61	0.5163617	1.675919	0.883	0.742	1.16E-56
BTN2A2	2.09E-28	0.5163527	1.675904	0.361	0.167	6.59E-24
MRPL53	1.23E-73	0.5160926	1.675468	0.801	0.506	3.87E-69
CWC27	6.39E-51	0.516059	1.675412	0.836	0.632	2.02E-46
RPS19	0.00E+00	0.5160328	1.675368	1	1	0.00E+00
C2CD3	2.97E-26	0.5159956	1.675306	0.605	0.464	9.38E-22
CSE1L	1.40E-42	0.5159072	1.675158	0.913	0.835	4.41E-38
DDX60	2.07E-30	0.5156956	1.674803	0.333	0.135	6.52E-26
APOL2	6.68E-29	0.5156706	1.674761	0.501	0.291	2.11E-24
CCDC28A	1.60E-32	0.5155513	1.674561	0.483	0.255	5.05E-28
CTBP1	4.30E-65	0.515457	1.674404	0.935	0.801	1.36E-60
HBD	2.97E-44	0.5149727	1.673593	0.128	0	9.37E-40
KLHL17	3.67E-33	0.5146815	1.673105	0.357	0.15	1.16E-28
MRPS9	4.84E-39	0.5145982	1.672966	0.716	0.493	1.53E-34
ARMT1	1.96E-49	0.5145779	1.672932	0.882	0.718	6.17E-45
SIGIRR	3.69E-32	0.5145711	1.672921	0.498	0.301	1.17E-27

TAP2	2.15E-40	0.5145492	1.672884	0.76	0.538	6.79E-36
SMARCA1	2.21E-60	0.5143981	1.672631	0.975	0.953	6.98E-56
RCCD1	8.22E-28	0.5142003	1.672301	0.486	0.285	2.59E-23
BTN3A3	2.40E-30	0.513967	1.67191	0.305	0.115	7.57E-26
FAH	8.37E-39	0.5138373	1.671694	0.84	0.656	2.64E-34
H1-2	2.04E-24	0.5136651	1.671406	0.454	0.278	6.44E-20
RNGTT	4.95E-33	0.513553	1.671218	0.692	0.511	1.56E-28
SAE1	9.98E-79	0.5134323	1.671017	0.988	0.926	3.15E-74
WSB2	3.17E-82	0.5133699	1.670912	0.986	0.954	9.99E-78
HNRNPA3	8.64E-140	0.513365	1.670904	1	1	2.72E-135
SLC25A30	1.02E-32	0.5131348	1.67052	0.533	0.306	3.21E-28
BBC3	5.66E-33	0.5129491	1.67021	0.412	0.194	1.78E-28
PPIA	0.00E+00	0.512724	1.669834	1	1	0.00E+00
RHOBTB1	3.74E-23	0.5122099	1.668975	0.547	0.362	1.18E-18
SF3B6	1.31E-107	0.5120668	1.668737	0.997	0.994	4.14E-103
EPG5	1.92E-34	0.5119641	1.668565	0.669	0.459	6.05E-30
UXT	4.09E-64	0.5119163	1.668485	0.909	0.803	1.29E-59
PKIG	1.01E-49	0.5118542	1.668382	0.955	0.904	3.20E-45
RBM34	1.04E-46	0.5118127	1.668313	0.787	0.596	3.27E-42
SEPTIN7	6.69E-220	0.5117856	1.668267	1	1	2.11E-215
CEP152	2.82E-24	0.5115103	1.667808	0.411	0.235	8.91E-20
APOBEC3C	3.31E-59	0.5113386	1.667522	0.937	0.779	1.04E-54
CAP2	7.35E-37	0.5110548	1.667049	0.881	0.753	2.32E-32
TMEM203	5.11E-42	0.5110052	1.666966	0.72	0.477	1.61E-37
NDUFA2	2.44E-65	0.5109493	1.666873	0.893	0.719	7.69E-61
PDE10A	5.76E-29	0.5107699	1.666574	0.255	0.084	1.82E-24
RASSF3	7.27E-29	0.5106416	1.66636	0.583	0.368	2.29E-24
TSPAN5	6.50E-55	0.5104488	1.666039	0.96	0.86	2.05E-50
ARSK	3.72E-25	0.5102689	1.665739	0.666	0.499	1.17E-20
NDUFB2	9.42E-113	0.510256	1.665718	0.989	0.94	2.97E-108
IFT74	9.79E-37	0.5100184	1.665322	0.638	0.412	3.09E-32
ARPC5	2.09E-244	0.5099378	1.665188	1	1	6.59E-240
EDF1	6.12E-133	0.5099011	1.665126	1	0.999	1.93E-128
FCMR	1.85E-32	0.5096587	1.664723	0.156	0.022	5.84E-28
LSP1P4	3.28E-59	0.5095333	1.664514	0.762	0.493	1.03E-54
ORAI1	2.73E-36	0.5093134	1.664148	0.632	0.402	8.61E-32
PRIM2	1.36E-34	0.5086816	1.663097	0.546	0.311	4.29E-30
NUDT3	4.44E-51	0.5085237	1.662835	0.954	0.886	1.40E-46
EIF3K	1.30E-91	0.5084687	1.662743	1	0.998	4.10E-87
GNA12	8.49E-60	0.5082697	1.662412	0.957	0.868	2.68E-55

KLHL9	2.12E-63	0.5078422	1.661702	0.927	0.781	6.70E-59
C11orf98	5.96E-52	0.5078085	1.661646	0.682	0.446	1.88E-47
SPG11	8.39E-30	0.5074771	1.661095	0.625	0.426	2.65E-25
MRPL20	2.92E-67	0.5074112	1.660986	0.974	0.929	9.21E-63
PAAF1	2.86E-34	0.5072828	1.660772	0.677	0.472	9.02E-30
CHML	2.13E-26	0.5071114	1.660488	0.649	0.492	6.73E-22
RABIF	3.19E-28	0.5070543	1.660393	0.57	0.373	1.01E-23
TMEM19	2.02E-37	0.5069912	1.660288	0.74	0.541	6.37E-33
BBS10	2.48E-37	0.5069306	1.660188	0.423	0.186	7.81E-33
ARID4A	1.36E-25	0.5068004	1.659971	0.499	0.315	4.28E-21
TBK1	8.58E-36	0.5062968	1.659136	0.717	0.5	2.71E-31
MICOS13	3.04E-67	0.5062241	1.659015	0.864	0.676	9.58E-63
DCXR	7.54E-34	0.5060378	1.658706	0.855	0.783	2.38E-29
RBM43	8.18E-27	0.5059146	1.658502	0.434	0.241	2.58E-22
ALDH1A2	2.07E-47	0.5057111	1.658164	0.151	0.002	6.52E-43
RPS10-NUDT	2.24E-270	0.505637	1.658041	1	1	7.06E-266
COQ3	3.85E-27	0.5055613	1.657916	0.501	0.29	1.21E-22
OSGIN1	5.18E-22	0.5047112	1.656507	0.334	0.167	1.64E-17
TCF7L2	6.68E-34	0.5046168	1.656351	0.7	0.487	2.11E-29
NUMA1	1.83E-48	0.5044297	1.656041	0.926	0.8	5.77E-44
USP1	8.11E-30	0.5042575	1.655756	0.814	0.667	2.56E-25
PIN4	2.30E-54	0.5039108	1.655182	0.666	0.38	7.27E-50
TCAF1	1.46E-49	0.503828	1.655045	0.943	0.857	4.61E-45
GTF2IP4	8.07E-79	0.5038116	1.655018	0.893	0.685	2.54E-74
CPSF3	1.08E-47	0.5037389	1.654897	0.783	0.54	3.41E-43
DEPDC7	2.08E-27	0.5036843	1.654807	0.331	0.143	6.57E-23
CCDC122	2.72E-26	0.5036553	1.654759	0.402	0.221	8.59E-22
AP005263.1	1.72E-84	0.5035704	1.654618	0.952	0.829	5.42E-80
NOX4	4.37E-32	0.5035621	1.654605	0.221	0.055	1.38E-27
MARCHF4	6.05E-24	0.5035595	1.6546	0.587	0.402	1.91E-19
IRF2	4.73E-41	0.5035346	1.654559	0.487	0.233	1.49E-36
C4orf46	1.92E-26	0.5034481	1.654416	0.492	0.289	6.06E-22
PPIH	5.81E-28	0.5034045	1.654344	0.657	0.508	1.83E-23
PRPF40B	2.88E-35	0.5033904	1.654321	0.376	0.156	9.10E-31
TNFAIP2	7.56E-42	0.5032793	1.654137	0.627	0.363	2.39E-37
TMEM220	5.05E-36	0.5030713	1.653793	0.341	0.128	1.59E-31
BOLA3	4.99E-55	0.5030672	1.653786	0.769	0.544	1.57E-50
OXR1	3.26E-30	0.5029523	1.653596	0.771	0.616	1.03E-25
HTATIP2	2.74E-37	0.5029115	1.653529	0.725	0.506	8.64E-33
XPO7	3.90E-45	0.502781	1.653313	0.829	0.622	1.23E-40

POMGNT2	3.94E-23	0.5025825	1.652985	0.319	0.158	1.24E-18
HSDL1	4.71E-30	0.5024515	1.652768	0.476	0.26	1.49E-25
PPP1R7	1.68E-68	0.5023143	1.652541	0.979	0.916	5.29E-64
CPSF2	5.25E-64	0.5022119	1.652372	0.969	0.878	1.66E-59
PARP1	9.99E-53	0.5021818	1.652322	0.957	0.873	3.15E-48
SOD3	2.03E-30	0.5021093	1.652203	0.327	0.133	6.41E-26
LRRCC1	7.30E-28	0.5020301	1.652072	0.356	0.165	2.30E-23
NYNRIN	1.12E-25	0.5019043	1.651864	0.197	0.055	3.55E-21
PGP	5.24E-30	0.5015497	1.651278	0.666	0.481	1.65E-25
RPS11	0.00E+00	0.5014823	1.651167	1	1	0.00E+00
ARHGEF25	1.81E-26	0.5013861	1.651008	0.479	0.284	5.71E-22
TTF2	4.34E-25	0.5011708	1.650653	0.553	0.384	1.37E-20
ZCCHC17	3.08E-45	0.5009652	1.650313	0.905	0.803	9.72E-41
ACBD6	5.02E-46	0.5008724	1.65016	0.867	0.716	1.58E-41
PDCL3	1.83E-38	0.5006179	1.64974	0.872	0.754	5.77E-34
ARL6IP5	3.18E-135	0.5006122	1.649731	1	0.998	1.00E-130
SREBF1	2.27E-35	0.5005858	1.649687	0.708	0.495	7.15E-31
PLGRKT	1.48E-40	0.5004836	1.649519	0.801	0.622	4.68E-36
EXTL3	2.75E-31	0.5004487	1.649461	0.683	0.499	8.69E-27
FEZ2	4.02E-59	0.5000951	1.648878	0.979	0.946	1.27E-54
UPF3A	1.17E-48	0.5000291	1.648769	0.878	0.709	3.67E-44
AL109918.1	8.50E-33	0.4999476	1.648635	0.252	0.072	2.68E-28
RNF207	5.96E-25	0.4998067	1.648403	0.242	0.097	1.88E-20
PTGER3	1.83E-20	0.4996537	1.64815	0.159	0.044	5.79E-16
MRPL36	5.72E-50	0.4996457	1.648137	0.866	0.74	1.80E-45
ARL3	1.69E-49	0.4995572	1.647991	0.949	0.882	5.33E-45
SPSB2	3.12E-37	0.4992768	1.647529	0.284	0.083	9.86E-33
NT5C2	1.17E-41	0.4990409	1.647141	0.865	0.709	3.68E-37
NUP205	2.67E-29	0.4985005	1.646251	0.601	0.396	8.42E-25
GUK1	3.07E-122	0.4983696	1.646035	1	0.999	9.70E-118
POLR2I	1.08E-48	0.4983168	1.645949	0.734	0.494	3.42E-44
GNG5	1.65E-140	0.4981842	1.64573	0.999	0.992	5.19E-136
POP5	1.20E-30	0.4981155	1.645617	0.759	0.663	3.79E-26
RPS15	3.67E-260	0.4980947	1.645583	1	1	1.16E-255
NEO1	1.54E-23	0.4976671	1.644879	0.642	0.501	4.84E-19
HTR7P1	4.06E-25	0.4976566	1.644862	0.285	0.116	1.28E-20
POLR3B	5.69E-39	0.4975713	1.644722	0.465	0.215	1.79E-34
HACD2	1.88E-41	0.4975065	1.644615	0.872	0.719	5.92E-37
TCEAL8	1.83E-48	0.4973934	1.644429	0.952	0.862	5.76E-44
N4BP2L2	3.14E-53	0.4972443	1.644184	0.94	0.874	9.89E-49

RPL6P27	4.69E-231	0.4971513	1.644031	1	1	1.48E-226
FAR2	4.45E-25	0.4969509	1.643702	0.448	0.255	1.40E-20
DCK	3.54E-31	0.4968874	1.643597	0.732	0.579	1.12E-26
SMARCA4	2.27E-64	0.4968776	1.643581	0.974	0.914	7.16E-60
AASDH	6.31E-31	0.4965535	1.643049	0.289	0.104	1.99E-26
RPL9P7	2.30E-144	0.4963986	1.642794	0.986	0.929	7.25E-140
IL32	2.97E-30	0.4963279	1.642678	0.242	0.079	9.38E-26
WDR6	6.46E-48	0.496312	1.642652	0.781	0.544	2.04E-43
MAVS	9.89E-49	0.4962774	1.642595	0.916	0.794	3.12E-44
NUP85	3.60E-25	0.496199	1.642466	0.616	0.454	1.14E-20
UBE2E3	4.40E-58	0.4960498	1.642221	0.955	0.867	1.39E-53
MTMR6	3.32E-41	0.4959937	1.642129	0.95	0.882	1.05E-36
ZDHHC7	1.91E-52	0.4958641	1.641916	0.935	0.815	6.03E-48
XPA	5.59E-43	0.4957321	1.6417	0.52	0.261	1.76E-38
TMTC1	1.65E-08	0.4955981	1.64148	0.496	0.427	5.22E-04
NNT-AS1	8.45E-29	0.4955004	1.641319	0.72	0.597	2.67E-24
TIMM10	1.03E-39	0.4953897	1.641138	0.7	0.542	3.26E-35
MRPL35	1.40E-51	0.4952342	1.640882	0.829	0.594	4.40E-47
NKTR	5.21E-43	0.4951181	1.640692	0.895	0.795	1.64E-38
AC011511.1	1.96E-59	0.4949541	1.640423	0.522	0.225	6.18E-55
MVK	3.17E-27	0.4946731	1.639962	0.327	0.141	1.00E-22
HLA-DPB1	3.39E-24	0.4945955	1.639835	0.469	0.282	1.07E-19
MPHOSPH9	2.61E-23	0.4945341	1.639734	0.574	0.415	8.23E-19
SCUBE3	6.62E-51	0.4945009	1.63968	0.886	0.695	2.09E-46
NOL7	1.10E-79	0.4944387	1.639578	0.988	0.938	3.45E-75
DGLUCY	1.66E-26	0.4944175	1.639543	0.438	0.242	5.23E-22
FANCM	4.14E-19	0.4943927	1.639502	0.316	0.165	1.31E-14
PLCXD3	1.27E-14	0.4943903	1.639498	0.109	0.035	4.00E-10
KDM4A	2.51E-36	0.494374	1.639472	0.614	0.375	7.91E-32
UBR1	1.37E-31	0.4941473	1.6391	0.675	0.462	4.33E-27
HIRIP3	2.95E-23	0.4939748	1.638817	0.463	0.283	9.30E-19
NFYB	1.14E-35	0.4936762	1.638328	0.762	0.57	3.59E-31
MAPK6	1.46E-63	0.4935781	1.638167	0.969	0.854	4.62E-59
CISD1	1.54E-44	0.4933871	1.637854	0.862	0.759	4.85E-40
TNRC6B	1.50E-32	0.4932043	1.637555	0.777	0.609	4.72E-28
CPXM2	1.26E-16	0.4929959	1.637214	0.153	0.053	3.99E-12
MRPL40	3.00E-49	0.4929425	1.637126	0.915	0.83	9.45E-45
HDAC5	9.89E-34	0.4925642	1.636507	0.783	0.616	3.12E-29
LMNB2	4.17E-32	0.4918849	1.635396	0.859	0.753	1.31E-27
NKAP	6.34E-35	0.4918703	1.635372	0.798	0.671	2.00E-30

ABHD17C	8.04E-26	0.4917445	1.635166	0.396	0.206	2.54E-21
ESCO2	1.57E-31	0.4913892	1.634585	0.575	0.351	4.95E-27
NME1	1.16E-97	0.4912773	1.634403	1	1	3.64E-93
CWF19L1	2.22E-33	0.491154	1.634201	0.678	0.468	7.01E-29
LAMTOR1	5.09E-92	0.4906093	1.633311	0.998	0.988	1.61E-87
ASH2L	8.15E-34	0.4906065	1.633307	0.763	0.565	2.57E-29
WDR70	1.62E-26	0.4903841	1.632943	0.609	0.416	5.10E-22
NDUFV3	7.25E-48	0.4902879	1.632786	0.889	0.755	2.29E-43
POLR2G	3.59E-64	0.4898923	1.63214	0.986	0.963	1.13E-59
ZSCAN31	4.85E-20	0.4896927	1.631815	0.121	0.024	1.53E-15
RPL35	3.29E-250	0.4896324	1.631716	1	1	1.04E-245
RMI1	6.44E-23	0.4894494	1.631418	0.489	0.306	2.03E-18
CALM2	6.11E-269	0.4892951	1.631166	1	1	1.93E-264
HSD17B14	1.51E-47	0.4892148	1.631035	0.178	0.012	4.78E-43
AEBP1	5.89E-51	0.4891401	1.630913	0.908	0.716	1.86E-46
LINC00517	1.16E-30	0.4891125	1.630868	0.156	0.023	3.67E-26
PIAS3	2.77E-42	0.489099	1.630846	0.754	0.518	8.74E-38
TCF19	5.96E-25	0.4888645	1.630464	0.475	0.278	1.88E-20
TMSB15A	7.24E-34	0.4886716	1.630149	0.233	0.062	2.28E-29
RPL19	0.00E+00	0.4885989	1.630031	1	1	0.00E+00
CCDC90B	2.76E-51	0.4885929	1.630021	0.941	0.837	8.72E-47
FAM216A	1.60E-30	0.4883198	1.629576	0.7	0.571	5.04E-26
TEAD2	1.47E-29	0.4882971	1.629539	0.581	0.372	4.64E-25
ETV5	1.12E-34	0.4882369	1.629441	0.843	0.711	3.53E-30
PDCD5	6.00E-69	0.4881104	1.629235	0.957	0.897	1.89E-64
EZH2	1.34E-21	0.4881096	1.629233	0.568	0.409	4.22E-17
CLSPN	1.21E-18	0.4878707	1.628844	0.522	0.415	3.83E-14
DARS2	2.90E-32	0.487835	1.628786	0.541	0.315	9.14E-28
CREM	1.59E-37	0.4875615	1.628341	0.715	0.496	5.02E-33
CCDC9B	4.38E-28	0.4875345	1.628297	0.357	0.168	1.38E-23
ERH	9.39E-114	0.4874728	1.628196	1	0.998	2.96E-109
SUOX	3.29E-24	0.4874549	1.628167	0.371	0.201	1.04E-19
PRKCZ	3.82E-38	0.4873158	1.627941	0.168	0.018	1.20E-33
BOLA3-AS1	5.84E-28	0.4872258	1.627794	0.342	0.155	1.84E-23
PCMTD2	1.86E-33	0.4870011	1.627428	0.49	0.257	5.87E-29
POLB	6.67E-28	0.486973	1.627383	0.511	0.302	2.10E-23
ZFAND2B	1.59E-28	0.4865181	1.626642	0.582	0.382	5.02E-24
WLS	1.71E-33	0.4864988	1.626611	0.877	0.769	5.40E-29
RBM8A	1.05E-65	0.4863585	1.626383	0.992	0.981	3.32E-61
RPS9	0.00E+00	0.4863514	1.626371	1	1	0.00E+00

HLA-B	8.91E-34	0.4862529	1.626211	1	1	2.81E-29
KIRREL3	4.45E-26	0.4859984	1.625797	0.419	0.235	1.40E-21
PRSS3	1.65E-18	0.4857861	1.625452	0.382	0.247	5.21E-14
ACTR1B	8.31E-49	0.4857764	1.625436	0.922	0.789	2.62E-44
DNAJC8	1.27E-88	0.4852999	1.624662	0.997	0.987	4.01E-84
CENPQ	7.80E-22	0.4851526	1.624423	0.341	0.171	2.46E-17
SSR4	7.41E-100	0.484996	1.624168	1	0.998	2.34E-95
LDLR	1.81E-37	0.4847925	1.623838	0.908	0.807	5.72E-33
NLN	3.02E-39	0.4847803	1.623818	0.88	0.765	9.53E-35
AP001931.1	2.92E-69	0.4846145	1.623549	0.752	0.475	9.22E-65
HDAC1	2.95E-67	0.4845702	1.623477	0.981	0.95	9.29E-63
MICU2	7.84E-50	0.4845216	1.623398	0.907	0.741	2.47E-45
HIBADH	1.06E-39	0.4843766	1.623163	0.857	0.695	3.35E-35
CENPI	3.57E-23	0.4840479	1.622629	0.265	0.109	1.13E-18
RFC5	1.77E-28	0.4839601	1.622487	0.452	0.241	5.59E-24
CNTRL	2.59E-24	0.4839067	1.6224	0.354	0.175	8.16E-20
ROBO1	2.43E-41	0.4838877	1.62237	0.712	0.463	7.65E-37
PRR13	1.89E-73	0.4837832	1.6222	0.988	0.966	5.97E-69
RNF34	5.37E-29	0.4835753	1.621863	0.681	0.502	1.69E-24
HOXD3	5.54E-40	0.4833916	1.621565	0.187	0.025	1.75E-35
ATOX1	7.68E-64	0.4833829	1.621551	0.886	0.709	2.42E-59
SLITRK4	5.98E-29	0.4829558	1.620858	0.113	0.008	1.89E-24
PCNA	1.77E-51	0.4827644	1.620548	0.946	0.856	5.59E-47
MTFR2	1.82E-21	0.4825444	1.620192	0.263	0.118	5.74E-17
HNRNPL	1.09E-83	0.4823601	1.619893	0.995	0.966	3.43E-79
CAMTA1	2.11E-65	0.4821553	1.619561	0.954	0.891	6.65E-61
SMIM26	9.18E-52	0.4820681	1.61942	0.846	0.657	2.90E-47
NOP10	5.78E-97	0.4818581	1.61908	0.996	0.992	1.82E-92
NUDCD2	1.90E-35	0.4818328	1.619039	0.835	0.691	6.01E-31
NDUFAF3	1.34E-68	0.481768	1.618934	0.974	0.927	4.23E-64
GINS4	1.07E-24	0.4815584	1.618595	0.546	0.373	3.38E-20
CBX3	1.14E-129	0.4814461	1.618413	1	1	3.61E-125
BRCA1	1.91E-23	0.4811292	1.6179	0.578	0.414	6.02E-19
ACAD11	3.96E-29	0.4809387	1.617592	0.4	0.203	1.25E-24
CSTF3	1.20E-36	0.4808187	1.617398	0.756	0.558	3.78E-32
RNF115	1.29E-46	0.4806038	1.61705	0.943	0.839	4.08E-42
MALSU1	3.84E-47	0.4805237	1.616921	0.881	0.796	1.21E-42
FIS1	4.36E-65	0.4805055	1.616892	0.983	0.976	1.38E-60
TRIM21	1.01E-24	0.4802358	1.616456	0.401	0.216	3.19E-20
INPP5A	5.78E-30	0.4801433	1.616306	0.625	0.441	1.82E-25

EIF2B3	6.41E-40	0.4799909	1.61606	0.817	0.639	2.02E-35
PPIG	9.24E-47	0.4798613	1.61585	0.946	0.862	2.91E-42
PSMB8	1.09E-37	0.4797304	1.615639	0.883	0.733	3.44E-33
OSR1	1.85E-28	0.4796456	1.615502	0.278	0.101	5.84E-24
PRRT1	3.44E-35	0.4796211	1.615462	0.188	0.036	1.09E-30
COX6C	8.34E-99	0.4793475	1.61502	0.992	0.947	2.63E-94
RAD50	2.90E-42	0.4793171	1.614971	0.868	0.724	9.14E-38
LINC00909	6.80E-24	0.4793031	1.614948	0.459	0.276	2.14E-19
MRPS24	3.97E-92	0.4792735	1.614901	0.986	0.951	1.25E-87
ANKRD26	7.07E-29	0.4789105	1.614315	0.658	0.496	2.23E-24
AMACR	1.70E-32	0.4786699	1.613926	0.422	0.2	5.35E-28
UQCRQ	3.15E-120	0.4784618	1.61359	0.995	0.969	9.95E-116
ABCC9	4.75E-21	0.4781633	1.613109	0.496	0.321	1.50E-16
YY1	3.40E-100	0.4777317	1.612413	0.996	0.982	1.07E-95
CACNB3	5.35E-26	0.4773473	1.611793	0.544	0.348	1.69E-21
MYD88	5.12E-28	0.4772804	1.611685	0.64	0.435	1.62E-23
MINDY2	3.23E-32	0.4769613	1.611171	0.901	0.795	1.02E-27
CHD6	3.70E-25	0.476937	1.611132	0.571	0.385	1.17E-20
MRPL16	9.55E-43	0.476908	1.611085	0.877	0.712	3.01E-38
COTL1	7.48E-171	0.4767992	1.61091	1	1	2.36E-166
MT-ND5	2.17E-191	0.4766694	1.610701	1	1	6.85E-187
CIR1	2.27E-33	0.4763893	1.61025	0.747	0.614	7.17E-29
HMG20B	6.39E-54	0.4759128	1.609483	0.94	0.796	2.01E-49
AP000311.1	8.07E-80	0.475747	1.609216	0.78	0.487	2.54E-75
LYRM7	2.92E-28	0.4756321	1.609031	0.712	0.571	9.21E-24
MAT2B	5.51E-52	0.4754682	1.608767	0.957	0.868	1.74E-47
EBAG9	3.25E-26	0.4754524	1.608742	0.545	0.356	1.03E-21
LYPLA1	1.07E-77	0.4752377	1.608397	0.989	0.946	3.37E-73
GSR	1.65E-33	0.475016	1.60804	0.711	0.508	5.20E-29
RPL6	6.43E-296	0.4749085	1.607867	1	1	2.03E-291
CAPN5	1.89E-28	0.4748027	1.607697	0.481	0.273	5.96E-24
LDB1	3.67E-38	0.4746366	1.60743	0.914	0.83	1.16E-33
COG8	5.00E-33	0.4744926	1.607198	0.77	0.595	1.58E-28
RBBP7	3.37E-88	0.4744711	1.607164	0.996	0.97	1.06E-83
PDLIM7	4.26E-106	0.4743625	1.606989	0.999	0.998	1.34E-101
MRPL11	2.95E-41	0.4743445	1.60696	0.901	0.817	9.30E-37
NIPSNAP1	1.44E-35	0.4740248	1.606447	0.905	0.814	4.54E-31
ACTR3	9.30E-205	0.4739751	1.606367	1	1	2.93E-200
LIG3	1.12E-24	0.4739365	1.606305	0.564	0.385	3.53E-20
TCHP	2.64E-28	0.47352	1.605636	0.603	0.411	8.32E-24

NDUFC1	2.81E-56	0.4734695	1.605555	0.889	0.743	8.86E-52
DYRK4	6.11E-32	0.4731994	1.605121	0.665	0.46	1.93E-27
MITD1	7.41E-24	0.4730957	1.604955	0.59	0.435	2.34E-19
C7orf25	1.32E-28	0.4729818	1.604772	0.414	0.213	4.16E-24
DDX59	5.19E-25	0.4727449	1.604392	0.54	0.374	1.64E-20
HDAC7	4.83E-49	0.4721905	1.603503	0.922	0.779	1.52E-44
DNAJC14	6.09E-26	0.4719004	1.603038	0.574	0.385	1.92E-21
APEX2	3.83E-26	0.4718734	1.602994	0.487	0.289	1.21E-21
FOSL2	2.14E-54	0.4714867	1.602375	0.893	0.679	6.76E-50
NBDY	4.98E-37	0.4712602	1.602012	0.88	0.805	1.57E-32
METTL15	1.51E-31	0.4711745	1.601875	0.539	0.315	4.76E-27
LIN52	4.01E-26	0.4711727	1.601872	0.459	0.271	1.27E-21
TRIM8	3.72E-49	0.4710421	1.601662	0.932	0.82	1.17E-44
GPAT3	8.33E-32	0.4709809	1.601564	0.349	0.145	2.63E-27
TUT4	2.98E-25	0.4708574	1.601367	0.632	0.452	9.41E-21
FADS2	2.30E-31	0.4708331	1.601328	0.843	0.683	7.25E-27
HAT1	9.39E-43	0.4708287	1.601321	0.925	0.813	2.96E-38
CBX1	2.43E-60	0.4707242	1.601153	0.985	0.946	7.66E-56
PRKG1	1.76E-28	0.4703798	1.600602	0.342	0.15	5.54E-24
NNT	1.49E-42	0.4702406	1.600379	0.893	0.763	4.70E-38
NCOR1	3.60E-54	0.4698802	1.599802	0.977	0.944	1.14E-49
PTMAP5	1.70E-84	0.4698523	1.599758	0.869	0.602	5.36E-80
SWI5	2.86E-32	0.4698125	1.599694	0.732	0.649	9.03E-28
ALDH9A1	2.08E-57	0.4697035	1.59952	0.974	0.896	6.55E-53
METTL17	1.38E-27	0.4695019	1.599197	0.721	0.549	4.36E-23
TAF12	4.60E-39	0.4690113	1.598413	0.753	0.528	1.45E-34
POLE4	2.40E-49	0.4689832	1.598368	0.876	0.818	7.56E-45
PAGR1	3.77E-39	0.4689338	1.598289	0.612	0.369	1.19E-34
MRPL17	3.92E-49	0.4689069	1.598246	0.968	0.936	1.24E-44
TNRC6C	1.79E-29	0.4687698	1.598027	0.452	0.24	5.64E-25
SNAP29	6.42E-38	0.4685396	1.597659	0.856	0.713	2.02E-33
TMBIM4	1.03E-66	0.4682318	1.597168	0.994	0.981	3.26E-62
TPP2	9.28E-48	0.4682178	1.597145	0.935	0.817	2.93E-43
MT-ND2	4.79E-293	0.4681034	1.596963	1	1	1.51E-288
ORC3	1.28E-31	0.467884	1.596612	0.611	0.39	4.05E-27
MRPS27	5.68E-29	0.4678226	1.596514	0.746	0.578	1.79E-24
SLC35E2B	2.98E-31	0.4677864	1.596456	0.71	0.529	9.41E-27
CHMP4A	6.78E-37	0.4677207	1.596351	0.848	0.717	2.14E-32
COX6A1P2	4.37E-77	0.4675483	1.596076	0.74	0.435	1.38E-72
MMP1	3.87E-14	0.4675205	1.596032	0.356	0.403	1.22E-09

ENY2	7.62E-75	0.4671936	1.59551	0.986	0.966	2.40E-70
PPP1R35	1.41E-40	0.4669116	1.59506	0.546	0.288	4.44E-36
FAM217B	4.35E-31	0.466812	1.594902	0.639	0.426	1.37E-26
RIT1	1.51E-30	0.4668075	1.594894	0.755	0.576	4.78E-26
TSPAN15	5.57E-38	0.4666778	1.594688	0.237	0.053	1.76E-33
ATP5MG	2.07E-104	0.4664939	1.594394	0.996	0.959	6.54E-100
DDB2	1.73E-27	0.4663151	1.594109	0.632	0.442	5.46E-23
PHF21A	1.49E-33	0.4662274	1.593969	0.574	0.337	4.70E-29
PDZRN3	3.22E-16	0.46592	1.593479	0.451	0.3	1.01E-11
LINC00339	9.33E-22	0.4657414	1.593195	0.378	0.205	2.94E-17
SP100	2.63E-71	0.465564	1.592912	0.996	0.992	8.29E-67
CCDC15	3.59E-31	0.4653884	1.592633	0.228	0.061	1.13E-26
NRM	9.06E-27	0.4653706	1.592604	0.665	0.482	2.86E-22
CENPC	8.99E-22	0.4652667	1.592439	0.525	0.368	2.83E-17
RAP1GDS1	8.39E-45	0.4652138	1.592355	0.892	0.741	2.64E-40
IER2	2.82E-50	0.4649267	1.591897	0.956	0.848	8.89E-46
BCL2L12	1.39E-21	0.4649093	1.59187	0.554	0.394	4.39E-17
C9orf78	1.91E-53	0.4647947	1.591687	0.964	0.9	6.04E-49
DCLK2	1.08E-24	0.4646155	1.591402	0.403	0.211	3.40E-20
FANCL	5.47E-28	0.4641809	1.590711	0.357	0.162	1.73E-23
CAMK4	3.82E-24	0.4637606	1.590042	0.252	0.097	1.20E-19
GSTM5	4.09E-26	0.463729	1.589992	0.176	0.043	1.29E-21
DCAF7	1.85E-44	0.4636461	1.58986	0.974	0.929	5.82E-40
DUT	3.69E-56	0.4635399	1.589691	0.983	0.976	1.17E-51
JDP2	2.94E-32	0.4631835	1.589125	0.767	0.59	9.27E-28
THUMPD3	2.36E-36	0.4629237	1.588712	0.838	0.681	7.46E-32
TUBG1	1.95E-38	0.4628656	1.58862	0.876	0.736	6.16E-34
RPS10	7.91E-286	0.4626137	1.58822	1	1	2.49E-281
PCNT	3.92E-25	0.462598	1.588195	0.572	0.381	1.23E-20
CCDC12	5.65E-28	0.462575	1.588158	0.782	0.671	1.78E-23
RNASEK-C17	9.12E-99	0.4625103	1.588055	0.986	0.936	2.88E-94
TDP1	8.69E-21	0.4624573	1.587971	0.428	0.257	2.74E-16
ABHD11	1.21E-24	0.4623807	1.58785	0.53	0.351	3.81E-20
MLLT11	1.85E-25	0.4620785	1.58737	0.772	0.622	5.84E-21
SNTB2	1.93E-41	0.461616	1.586636	0.95	0.89	6.09E-37
WWC2	4.36E-33	0.4615633	1.586552	0.983	0.974	1.38E-28
CENPO	3.71E-20	0.4611787	1.585942	0.434	0.26	1.17E-15
RB1	9.22E-41	0.4609856	1.585636	0.904	0.769	2.91E-36
URGCP-MRP	1.26E-74	0.4609636	1.585601	0.936	0.796	3.97E-70
TRAF4	2.23E-27	0.4605385	1.584927	0.698	0.507	7.03E-23

TRUB1	7.20E-28	0.460536	1.584923	0.658	0.452	2.27E-23
NSMCE2	2.29E-31	0.4604873	1.584846	0.707	0.507	7.22E-27
FIP1L1	9.18E-51	0.460244	1.584461	0.878	0.669	2.89E-46
PSMC3IP	1.29E-17	0.4601235	1.58427	0.393	0.246	4.08E-13
NDUFS7	1.38E-54	0.4600837	1.584207	0.95	0.915	4.37E-50
TARS2	3.73E-21	0.4600567	1.584164	0.454	0.293	1.18E-16
STN1	2.05E-24	0.4599372	1.583975	0.52	0.329	6.47E-20
RPL36	7.66E-190	0.4598251	1.583797	1	1	2.42E-185
BTN3A1	9.90E-25	0.459726	1.58364	0.417	0.227	3.12E-20
PPIAP22	1.73E-95	0.4596908	1.583584	0.924	0.758	5.46E-91
RAD18	3.43E-23	0.4596108	1.583458	0.509	0.324	1.08E-18
VPS11	8.86E-23	0.4594141	1.583146	0.546	0.366	2.79E-18
DCHS1	2.94E-37	0.4593933	1.583113	0.198	0.035	9.29E-33
RALBP1	1.28E-64	0.4593104	1.582982	0.988	0.964	4.03E-60
TMEM126B	1.02E-38	0.4592525	1.58289	0.886	0.763	3.20E-34
AL590867.2	4.74E-137	0.4589723	1.582447	0.99	0.946	1.49E-132
DHX29	6.86E-47	0.4589576	1.582424	0.951	0.871	2.16E-42
CCND3	1.41E-36	0.4587773	1.582138	0.821	0.632	4.44E-32
PAF1	4.75E-41	0.4585441	1.581769	0.826	0.624	1.50E-36
FAM204A	1.15E-44	0.4585343	1.581754	0.88	0.733	3.62E-40
DPH5	2.42E-26	0.4583201	1.581415	0.678	0.496	7.63E-22
CCDC25	7.63E-40	0.4579637	1.580852	0.824	0.657	2.41E-35
MRPS22	1.49E-41	0.4574362	1.580018	0.887	0.747	4.71E-37
SURF2	1.13E-19	0.4573661	1.579907	0.46	0.319	3.56E-15
WRAP73	2.40E-23	0.4573404	1.579867	0.421	0.237	7.57E-19
TPBG	5.25E-42	0.4571708	1.579599	0.984	0.957	1.65E-37
MRPS17	1.96E-34	0.4571431	1.579555	0.68	0.487	6.20E-30
RETSAT	6.27E-33	0.4569635	1.579271	0.638	0.403	1.98E-28
RTP4	6.92E-32	0.456767	1.578961	0.135	0.012	2.18E-27
LRCH3	3.82E-31	0.4567617	1.578953	0.739	0.583	1.20E-26
HAUS8	1.66E-15	0.4567027	1.578859	0.37	0.228	5.24E-11
ZFPM2	4.72E-27	0.4562497	1.578144	0.161	0.036	1.49E-22
UBE2S	6.11E-10	0.4561047	1.577916	0.97	0.983	1.93E-05
ALKBH7	5.54E-34	0.4560826	1.577881	0.805	0.751	1.75E-29
SLC5A3	8.63E-22	0.4559933	1.57774	0.687	0.526	2.72E-17
POLRMT	1.23E-31	0.4559553	1.57768	0.489	0.265	3.88E-27
FMC1	2.47E-46	0.4558838	1.577567	0.596	0.33	7.79E-42
ABRACL	8.66E-27	0.4555033	1.576967	0.778	0.67	2.73E-22
RPL23AP82	7.88E-41	0.4554398	1.576867	0.88	0.749	2.49E-36
CMTR2	7.90E-24	0.4553318	1.576697	0.522	0.327	2.49E-19

ME1	1.29E-37	0.455241	1.576553	0.941	0.908	4.06E-33
PDHB	2.53E-67	0.454827	1.575901	0.992	0.97	7.99E-63
PLD1	1.21E-19	0.4548078	1.57587	0.282	0.135	3.81E-15
REEP4	3.25E-16	0.4547697	1.57581	0.566	0.44	1.03E-11
MMP17	6.50E-44	0.4544891	1.575368	0.285	0.07	2.05E-39
POLE2	5.19E-20	0.454216	1.574938	0.308	0.151	1.64E-15
FAM174C	3.39E-32	0.453991	1.574584	0.831	0.728	1.07E-27
LINC00672	1.07E-25	0.4536774	1.57409	0.249	0.101	3.37E-21
NEURL1B	2.08E-14	0.4536069	1.573979	0.197	0.101	6.57E-10
LRRC42	6.11E-36	0.4534734	1.573769	0.796	0.598	1.93E-31
BROX	4.45E-45	0.4533867	1.573633	0.938	0.841	1.40E-40
MTMR4	2.59E-26	0.4531954	1.573332	0.516	0.308	8.18E-22
BBS7	6.55E-34	0.4531688	1.57329	0.704	0.484	2.07E-29
COMMD6	5.22E-73	0.4530367	1.573082	0.981	0.934	1.65E-68
MCM2	2.89E-25	0.4528565	1.572798	0.487	0.283	9.11E-21
XRCC1	3.71E-30	0.4525167	1.572264	0.617	0.399	1.17E-25
UFL1	5.20E-41	0.452148	1.571685	0.938	0.861	1.64E-36
CEP63	6.02E-27	0.4519021	1.571298	0.703	0.542	1.90E-22
MRPS18B	3.83E-45	0.4518603	1.571232	0.937	0.831	1.21E-40
NLRC5	1.38E-20	0.4517757	1.571099	0.465	0.296	4.35E-16
SMC6	6.07E-29	0.451513	1.570687	0.83	0.693	1.91E-24
PLEKHO1	4.63E-39	0.4512356	1.570251	0.872	0.701	1.46E-34
ZSCAN30	5.84E-25	0.4511966	1.57019	0.5	0.307	1.84E-20
NUP93	6.76E-30	0.4511907	1.570181	0.753	0.579	2.13E-25
PRKACB	3.35E-34	0.4511836	1.57017	0.842	0.677	1.06E-29
RPP25L	2.76E-16	0.4511309	1.570087	0.462	0.325	8.70E-12
POLD3	4.28E-18	0.4511171	1.570065	0.443	0.289	1.35E-13
SAV1	9.60E-30	0.4509697	1.569834	0.906	0.824	3.03E-25
KEAP1	1.54E-44	0.4505955	1.569246	0.853	0.652	4.86E-40
HMGB1P10	3.81E-71	0.4504911	1.569083	0.624	0.296	1.20E-66
TMPO-AS1	2.87E-22	0.4503696	1.568892	0.18	0.054	9.07E-18
PHYHD1	8.08E-19	0.4502483	1.568702	0.198	0.074	2.55E-14
USP33	7.80E-45	0.4498798	1.568124	0.949	0.857	2.46E-40
UBXN4	4.80E-90	0.4498482	1.568074	0.998	1	1.51E-85
MTIF3	1.05E-47	0.4496302	1.567732	0.674	0.408	3.32E-43
SETD9	1.12E-26	0.4496132	1.567706	0.329	0.143	3.55E-22
GTF2F2	2.85E-38	0.4496018	1.567688	0.959	0.932	8.98E-34
TMEM256	1.14E-49	0.4495897	1.567669	0.666	0.388	3.61E-45
FOXRED2	1.04E-26	0.4493073	1.567226	0.395	0.207	3.30E-22
AAMDC	5.19E-33	0.4493016	1.567217	0.68	0.502	1.64E-28

DUS2	5.11E-22	0.4492721	1.567171	0.371	0.2	1.61E-17
AC009133.6	3.77E-35	0.4491141	1.566923	0.553	0.323	1.19E-30
SH3BGRL3	5.97E-151	0.4489899	1.566729	1	1	1.88E-146
TMEM87B	6.25E-32	0.448561	1.566057	0.87	0.743	1.97E-27
HSDL2	1.07E-35	0.4480849	1.565312	0.883	0.779	3.36E-31
C18orf32	7.78E-39	0.4478946	1.565014	0.912	0.805	2.46E-34
DYNLT1	1.93E-82	0.4476417	1.564618	1	0.998	6.08E-78
GLOD4	4.02E-33	0.447595	1.564545	0.839	0.681	1.27E-28
RHOT1	3.01E-33	0.4475861	1.564531	0.687	0.463	9.48E-29
ISCU	1.74E-52	0.447474	1.564356	0.981	0.954	5.49E-48
AC079848.2	3.26E-36	0.4470714	1.563726	0.156	0.016	1.03E-31
NAA38	2.77E-52	0.4470001	1.563614	0.866	0.7	8.75E-48
RMDN1	2.03E-35	0.4465501	1.562911	0.874	0.759	6.40E-31
POLR3G	1.39E-16	0.4463769	1.56264	0.323	0.199	4.38E-12
NT5C3A	1.77E-30	0.4459367	1.561953	0.801	0.652	5.59E-26
SGCD	1.46E-07	0.4456293	1.561473	0.372	0.354	4.61E-03
AP001267.5	2.25E-74	0.4455335	1.561323	0.809	0.51	7.11E-70
RPL17	6.07E-243	0.4455323	1.561321	1	1	1.91E-238
ZFPM2-AS1	3.44E-35	0.4454473	1.561188	0.207	0.043	1.09E-30
CEP68	5.33E-20	0.445255	1.560888	0.509	0.343	1.68E-15
LCLAT1	2.12E-29	0.4449803	1.560459	0.747	0.573	6.69E-25
CBR1	5.33E-76	0.4448679	1.560284	0.999	0.992	1.68E-71
UEVLD	2.39E-33	0.4448528	1.560261	0.804	0.629	7.53E-29
ZYG11B	2.03E-34	0.4447635	1.560121	0.914	0.812	6.39E-30
TDP2	4.13E-42	0.444582	1.559838	0.824	0.616	1.30E-37
ANAPC13	2.25E-49	0.4445524	1.559792	0.969	0.917	7.09E-45
VPS35	9.96E-89	0.444521	1.559743	0.998	0.989	3.14E-84
COA4	1.47E-41	0.4444892	1.559693	0.909	0.855	4.62E-37
PCBD1	3.16E-18	0.4442869	1.559378	0.623	0.528	9.96E-14
AC016747.1	1.30E-25	0.444263	1.55934	0.467	0.279	4.09E-21
PPP1R26	1.10E-18	0.4442038	1.559248	0.3	0.165	3.48E-14
PRIMPOL	7.66E-22	0.4441303	1.559134	0.387	0.217	2.42E-17
AC089983.1	2.83E-40	0.4440784	1.559053	0.183	0.023	8.93E-36
SEPTIN10	3.87E-53	0.4439082	1.558787	0.984	0.939	1.22E-48
DNAJC19	1.07E-35	0.4433657	1.557942	0.768	0.579	3.39E-31
AC011448.1	3.09E-69	0.4430852	1.557505	0.829	0.582	9.76E-65
C11orf54	5.54E-31	0.4429259	1.557257	0.578	0.366	1.75E-26
USP21	7.60E-27	0.4429252	1.557256	0.393	0.194	2.40E-22
FDXR	2.95E-25	0.4429246	1.557255	0.465	0.264	9.31E-21
STX8	5.51E-27	0.4428401	1.557123	0.686	0.536	1.74E-22

ERCC5	3.70E-29	0.4424044	1.556445	0.701	0.52	1.17E-24
FAF1	5.90E-33	0.4423532	1.556365	0.746	0.559	1.86E-28
LZTS2	2.46E-32	0.441961	1.555755	0.81	0.633	7.75E-28
RPL26L1	2.57E-40	0.4419149	1.555683	0.932	0.886	8.12E-36
SCG5	4.23E-25	0.4419003	1.555661	0.255	0.094	1.33E-20
PDE9A	1.39E-51	0.4416978	1.555346	0.148	0	4.38E-47
PRR5L	3.26E-20	0.4415716	1.555149	0.729	0.639	1.03E-15
BANF1	6.52E-79	0.4414582	1.554973	1	1	2.06E-74
MRPL42	1.10E-57	0.4414559	1.554969	0.984	0.944	3.46E-53
NTHL1	3.42E-20	0.4413345	1.554781	0.564	0.405	1.08E-15
CINP	1.42E-30	0.4412504	1.55465	0.818	0.681	4.47E-26
C12orf43	4.18E-26	0.4409574	1.554194	0.636	0.46	1.32E-21
PSMA4	4.40E-103	0.4408828	1.554079	1	1	1.39E-98
FOCAD	7.81E-24	0.4408428	1.554016	0.569	0.371	2.46E-19
ZBTB8OS	3.43E-33	0.4407745	1.55391	0.705	0.522	1.08E-28
C1orf112	1.77E-19	0.4407518	1.553875	0.278	0.132	5.59E-15
DOCK7	1.07E-29	0.4406797	1.553763	0.86	0.755	3.39E-25
ZMAT5	1.92E-25	0.4406785	1.553761	0.674	0.555	6.06E-21
NUDT7	1.24E-38	0.4406301	1.553686	0.202	0.032	3.92E-34
POLR2A	1.85E-46	0.4405616	1.553579	0.934	0.853	5.85E-42
STK40	5.65E-28	0.4404021	1.553332	0.53	0.317	1.78E-23
TCIRG1	7.59E-39	0.4403447	1.553243	0.891	0.753	2.39E-34
FAM177A1	4.99E-58	0.4400256	1.552747	0.98	0.936	1.57E-53
ABCD3	7.64E-30	0.4399055	1.55256	0.762	0.591	2.41E-25
RWDD2B	1.02E-25	0.4398412	1.552461	0.753	0.619	3.23E-21
RPP30	1.64E-28	0.4396277	1.552129	0.828	0.675	5.18E-24
THAP7	1.11E-25	0.4395672	1.552035	0.544	0.344	3.50E-21
GLI4	1.00E-26	0.4395516	1.552011	0.358	0.168	3.16E-22
CBWD5	4.55E-61	0.439392	1.551763	0.942	0.854	1.44E-56
APOL3	4.01E-32	0.439392	1.551763	0.199	0.043	1.27E-27
RPL10	0.00E+00	0.4393456	1.551691	1	1	0.00E+00
HPS5	7.45E-27	0.4390968	1.551305	0.668	0.471	2.35E-22
NBN	3.97E-43	0.4390799	1.551279	0.942	0.827	1.25E-38
COQ5	6.62E-36	0.4389581	1.55109	0.725	0.496	2.09E-31
ATPAF1	4.41E-29	0.4387587	1.550781	0.804	0.643	1.39E-24
RPP21	7.52E-37	0.4385513	1.550459	0.684	0.474	2.37E-32
ADAMTS4	1.10E-20	0.438512	1.550399	0.365	0.209	3.47E-16
DENND10	8.74E-38	0.4384843	1.550356	0.9	0.802	2.76E-33
RPL13	0.00E+00	0.4384457	1.550296	1	1	0.00E+00
RNF214	1.82E-24	0.4383748	1.550186	0.449	0.259	5.74E-20

KBTBD7	2.67E-26	0.4381269	1.549802	0.299	0.122	8.44E-22
CCDC77	5.23E-18	0.4380779	1.549726	0.312	0.173	1.65E-13
DTX3L	7.16E-22	0.4380452	1.549675	0.552	0.386	2.26E-17
MRPS26	3.47E-25	0.4379881	1.549586	0.748	0.626	1.09E-20
TASOR	2.37E-30	0.4378739	1.549409	0.829	0.686	7.47E-26
SNRNP35	3.88E-23	0.4378087	1.549309	0.566	0.381	1.22E-18
PUDP	3.19E-23	0.4376673	1.54909	0.539	0.347	1.00E-18
NAA16	2.55E-19	0.4376598	1.549078	0.551	0.387	8.05E-15
POLH	1.14E-25	0.4375279	1.548874	0.725	0.568	3.59E-21
ALPK1	1.02E-23	0.4373717	1.548632	0.291	0.127	3.21E-19
BTBD10	2.20E-30	0.4373696	1.548628	0.81	0.65	6.93E-26
MMUT	1.71E-33	0.4373405	1.548583	0.748	0.543	5.39E-29
NT5C	2.91E-29	0.4366265	1.547478	0.806	0.677	9.17E-25
RPS19BP1	3.58E-40	0.4362019	1.546821	0.926	0.865	1.13E-35
MKKS	2.48E-40	0.4360434	1.546576	0.817	0.633	7.84E-36
SRSF6	3.55E-64	0.4359376	1.546412	0.992	0.969	1.12E-59
EIF5	1.24E-86	0.4358677	1.546304	1	0.996	3.93E-82
MAU2	4.39E-22	0.435695	1.546037	0.597	0.429	1.38E-17
ACTC1	1.45E-04	0.4355413	1.5458	0.122	0.097	1.00E+00
IK	7.19E-59	0.4355368	1.545793	0.987	0.964	2.27E-54
CEP295	1.56E-18	0.4355229	1.545771	0.366	0.217	4.91E-14
SSNA1	1.49E-40	0.4355182	1.545764	0.951	0.917	4.70E-36
MEN1	1.59E-25	0.4354446	1.54565	0.539	0.336	5.00E-21
TMEM80	2.28E-19	0.4354292	1.545626	0.399	0.236	7.19E-15
KIAA1586	2.22E-30	0.4353519	1.545507	0.532	0.311	7.01E-26
SLC44A2	2.08E-43	0.4353071	1.545438	0.947	0.885	6.55E-39
IL15	1.94E-22	0.4352713	1.545382	0.363	0.191	6.13E-18
CDH24	2.14E-28	0.4349953	1.544956	0.256	0.089	6.73E-24
ATXN2	3.35E-42	0.4349786	1.54493	0.932	0.825	1.06E-37
COX7C	2.47E-100	0.4349054	1.544817	0.995	0.975	7.79E-96
MRPS5	1.34E-33	0.4346805	1.54447	0.871	0.781	4.22E-29
CLDN7	6.04E-24	0.4346096	1.54436	0.134	0.025	1.91E-19
CDCA7	1.33E-16	0.4344574	1.544125	0.243	0.113	4.18E-12
DCTD	9.65E-52	0.4341815	1.543699	0.985	0.952	3.04E-47
FAM122B	7.76E-24	0.4341247	1.543611	0.536	0.342	2.45E-19
PPP4R1	7.70E-44	0.4339121	1.543283	0.934	0.818	2.43E-39
ASAP1	3.28E-62	0.4335789	1.542769	0.993	0.972	1.04E-57
ANKMY2	1.46E-22	0.43336	1.542431	0.594	0.409	4.60E-18
ZNF706	5.46E-38	0.4325813	1.541231	0.932	0.881	1.72E-33
UBA52	3.36E-208	0.4325214	1.541138	1	1	1.06E-203

CHAF1A	7.07E-22	0.4325102	1.541121	0.425	0.243	2.23E-17
NPM3	1.05E-23	0.4324756	1.541068	0.804	0.736	3.31E-19
NBEAL1	2.09E-60	0.4323546	1.540881	0.949	0.818	6.61E-56
GINS2	2.88E-19	0.4321548	1.540574	0.478	0.307	9.07E-15
HDHD2	2.62E-24	0.4318253	1.540066	0.707	0.537	8.27E-20
ZP3	6.62E-17	0.4317993	1.540026	0.419	0.284	2.09E-12
L3HYPDH	1.19E-29	0.4317479	1.539947	0.763	0.578	3.74E-25
REXO2	2.29E-99	0.4314112	1.539428	1	1	7.21E-95
ARNT2	2.09E-19	0.431178	1.53907	0.421	0.254	6.61E-15
SMARCB1	8.07E-47	0.431096	1.538943	0.944	0.843	2.55E-42
PKN1	7.47E-47	0.4307698	1.538441	0.933	0.818	2.36E-42
DNAJC13	1.07E-36	0.4304422	1.537937	0.875	0.736	3.38E-32
MAMDC2	8.43E-18	0.4300924	1.5374	0.136	0.038	2.66E-13
F2RL2	5.62E-19	0.4300049	1.537265	0.46	0.29	1.77E-14
COX7B	8.36E-72	0.4299977	1.537254	0.977	0.898	2.64E-67
EXOSC4	2.11E-23	0.4299662	1.537206	0.663	0.519	6.66E-19
CEP20	3.10E-31	0.4298956	1.537097	0.904	0.839	9.77E-27
ILK	1.73E-103	0.4298724	1.537061	1	0.995	5.46E-99
CSDC2	8.98E-38	0.429485	1.536466	0.204	0.036	2.83E-33
FBLIM1	1.16E-23	0.4292661	1.53613	0.979	0.974	3.66E-19
NMT2	2.21E-32	0.428994	1.535712	0.937	0.857	6.98E-28
BIRC6	6.90E-30	0.428759	1.535351	0.877	0.773	2.18E-25
APIP	2.69E-30	0.4287192	1.53529	0.736	0.552	8.48E-26
CEP192	4.90E-24	0.4286979	1.535257	0.382	0.195	1.55E-19
CHCHD2	3.27E-160	0.428615	1.53513	1	1	1.03E-155
SAP18	6.08E-103	0.4284838	1.534928	1	1	1.92E-98
TACO1	8.19E-28	0.4284384	1.534859	0.743	0.571	2.58E-23
TFDP1	4.01E-44	0.4281239	1.534376	0.967	0.904	1.26E-39
RPS23	1.92E-285	0.4280906	1.534325	1	1	6.06E-281
PIK3R2	7.05E-35	0.4279964	1.534181	0.76	0.577	2.22E-30
HVCN1	1.50E-27	0.4279634	1.53413	0.217	0.062	4.74E-23
LINC02802	4.76E-40	0.4278406	1.533942	0.547	0.306	1.50E-35
HSPB1	1.24E-86	0.4278266	1.53392	1	0.999	3.92E-82
ATF5	4.57E-23	0.4274853	1.533397	0.711	0.548	1.44E-18
DVL1	3.86E-28	0.4273789	1.533233	0.696	0.508	1.22E-23
RABGAP1	3.53E-24	0.4272381	1.533018	0.728	0.583	1.11E-19
S100PBP	3.00E-26	0.4272235	1.532995	0.554	0.348	9.48E-22
TMEM147	5.46E-45	0.4271877	1.53294	0.963	0.957	1.72E-40
ZHX3	5.08E-35	0.4270496	1.532729	0.767	0.556	1.60E-30
TRIM65	5.17E-25	0.4270227	1.532687	0.43	0.236	1.63E-20

NCOA2	2.29E-22	0.4269123	1.532518	0.431	0.247	7.21E-18
CEP41	9.08E-22	0.4268815	1.532471	0.629	0.464	2.86E-17
TP53INP1	2.76E-14	0.4268673	1.532449	0.345	0.216	8.69E-10
MTHFD1L	1.61E-46	0.4266261	1.53208	0.95	0.904	5.08E-42
TSPYL4	2.59E-17	0.426546	1.531957	0.581	0.439	8.16E-13
MARCKS	5.41E-52	0.4263923	1.531722	1	1	1.71E-47
PCED1B	1.75E-34	0.4263832	1.531708	0.224	0.052	5.52E-30
KIF16B	3.58E-18	0.426271	1.531536	0.348	0.194	1.13E-13
TWSG1	1.23E-52	0.4261267	1.531315	0.988	0.975	3.87E-48
CCDC34	6.78E-18	0.4260503	1.531198	0.543	0.423	2.14E-13
RPL23AP7	7.06E-39	0.4259565	1.531054	0.577	0.331	2.23E-34
HYAL2	1.51E-32	0.4258058	1.530823	0.743	0.534	4.77E-28
EFS	3.69E-22	0.4257997	1.530814	0.459	0.271	1.16E-17
CARD19	7.33E-30	0.4256035	1.530514	0.859	0.802	2.31E-25
NFATC2IP	1.76E-23	0.4255123	1.530374	0.685	0.534	5.55E-19
NAAA	2.07E-24	0.4254317	1.530251	0.693	0.535	6.53E-20
CASP4	1.02E-23	0.4254311	1.53025	0.873	0.795	3.22E-19
RFC4	7.09E-15	0.4252089	1.52991	0.465	0.315	2.24E-10
RPL27A	1.24E-252	0.4251968	1.529892	1	1	3.91E-248
UBA6	5.08E-35	0.4251518	1.529823	0.915	0.809	1.60E-30
MRPL43	1.09E-35	0.4251069	1.529754	0.937	0.876	3.44E-31
RPL17-C18or	1.15E-179	0.4251051	1.529751	1	1	3.62E-175
GTPBP10	4.89E-23	0.4250844	1.529719	0.671	0.51	1.54E-18
CEP70	4.10E-21	0.4250125	1.529609	0.495	0.32	1.29E-16
CFDP1	7.59E-33	0.4249045	1.529444	0.836	0.683	2.39E-28
PRXL2A	7.39E-19	0.4248923	1.529426	0.337	0.199	2.33E-14
TANK	2.31E-37	0.4248386	1.529344	0.928	0.821	7.30E-33
ADH5	6.50E-95	0.4248307	1.529332	1	0.984	2.05E-90
ZNF438	1.83E-19	0.4248289	1.529329	0.183	0.065	5.79E-15
GTPBP8	1.94E-23	0.4246304	1.529025	0.58	0.41	6.11E-19
FDPS	4.49E-56	0.4243089	1.528534	0.989	0.957	1.41E-51
HTR1B	6.93E-31	0.4241765	1.528331	0.114	0.006	2.18E-26
MTHFD1	2.04E-35	0.4240083	1.528074	0.891	0.772	6.44E-31
CPM	3.86E-14	0.4239715	1.528018	0.526	0.428	1.22E-09
AC010422.8	8.58E-87	0.4239479	1.527982	0.98	0.928	2.71E-82
CDK2AP1	1.61E-102	0.423917	1.527935	1	1	5.07E-98
FAM20B	3.24E-28	0.4239008	1.52791	0.856	0.758	1.02E-23
H2AW	2.11E-15	0.4237801	1.527726	0.261	0.146	6.66E-11
MAPRE2	1.05E-26	0.4234513	1.527223	0.805	0.669	3.30E-22
HACD1	7.95E-26	0.423435	1.527199	0.829	0.71	2.51E-21

HDDC2	2.99E-27	0.4232472	1.526912	0.9	0.832	9.43E-23
SUV39H1	3.81E-18	0.4232131	1.52686	0.307	0.162	1.20E-13
ZNF318	4.60E-17	0.4232014	1.526842	0.445	0.29	1.45E-12
BRCC3	3.61E-29	0.4231707	1.526795	0.731	0.546	1.14E-24
MTOR	1.76E-20	0.4230816	1.526659	0.56	0.386	5.55E-16
NECTIN2	1.50E-30	0.4228274	1.526271	0.927	0.856	4.73E-26
TYRO3	3.32E-22	0.4225314	1.525819	0.518	0.341	1.05E-17
NME2	2.05E-168	0.4225173	1.525798	1	1	6.46E-164
PAFAH2	7.06E-21	0.4224081	1.525631	0.399	0.229	2.23E-16
CHURC1	7.35E-44	0.4223686	1.525571	0.979	0.946	2.32E-39
DIS3L	1.49E-19	0.4223642	1.525564	0.495	0.33	4.69E-15
MOV10	2.44E-21	0.4223211	1.525498	0.642	0.489	7.69E-17
TNFRSF14	8.40E-17	0.4222372	1.52537	0.349	0.2	2.65E-12
CCNG1	8.95E-42	0.4221823	1.525287	0.98	0.957	2.82E-37
CYBRD1	1.12E-43	0.4221033	1.525166	0.981	0.946	3.53E-39
ZFYVE21	8.21E-30	0.4219989	1.525007	0.788	0.615	2.59E-25
RPAP1	2.90E-22	0.4218117	1.524721	0.448	0.273	9.16E-18
UBE2L3	1.69E-109	0.4217834	1.524678	1	1	5.32E-105
TBCK	2.80E-18	0.4217512	1.524629	0.566	0.421	8.84E-14
ZNF839	5.21E-21	0.421723	1.524586	0.264	0.118	1.64E-16
GTF3A	1.17E-47	0.4216793	1.524519	0.975	0.941	3.68E-43
FCRLB	1.49E-27	0.4215688	1.524351	0.26	0.09	4.70E-23
OST4	1.48E-101	0.4214711	1.524202	0.997	0.98	4.68E-97
CEP97	7.36E-13	0.4212531	1.52387	0.487	0.373	2.32E-08
RWDD3	3.59E-18	0.4212474	1.523861	0.497	0.335	1.13E-13
CD55	2.23E-41	0.4211536	1.523718	0.956	0.861	7.04E-37
NTNG1	1.40E-13	0.4205876	1.522856	0.177	0.078	4.43E-09
MYEOV	4.63E-45	0.4204061	1.52258	0.364	0.119	1.46E-40
CHCHD5	6.30E-31	0.4201429	1.522179	0.621	0.439	1.99E-26
TIPRL	4.69E-49	0.420125	1.522152	0.978	0.928	1.48E-44
USP28	6.28E-18	0.4193672	1.520999	0.465	0.305	1.98E-13
BMPR1A	8.44E-27	0.4193177	1.520923	0.693	0.505	2.66E-22
RPS7P1	9.23E-96	0.4193005	1.520897	0.98	0.905	2.91E-91
DDIAS	1.92E-15	0.4191628	1.520688	0.301	0.171	6.05E-11
NEDD8-MDP	3.56E-55	0.4191576	1.52068	0.623	0.333	1.12E-50
TGFB1I1	1.97E-56	0.4191496	1.520668	0.995	0.986	6.20E-52
GSN	9.01E-39	0.4190683	1.520544	0.99	0.978	2.84E-34
UBA3	5.01E-41	0.418876	1.520252	0.945	0.867	1.58E-36
CUTC	1.48E-20	0.4187953	1.520129	0.51	0.341	4.66E-16
NAP1L1	9.14E-183	0.418655	1.519916	1	1	2.88E-178

DENR	1.48E-68	0.4180268	1.518961	0.994	0.969	4.65E-64
TPM3	1.00E-149	0.4179848	1.518898	1	1	3.17E-145
CCDC66	6.72E-18	0.4179568	1.518855	0.501	0.348	2.12E-13
TTYH3	6.13E-35	0.4179207	1.5188	0.907	0.833	1.93E-30
PSMD7	1.91E-92	0.4177785	1.518584	0.999	0.995	6.03E-88
PAM16	2.66E-34	0.4176356	1.518367	0.652	0.448	8.38E-30
ZNF639	1.06E-30	0.4175616	1.518255	0.745	0.547	3.34E-26
RUFY1	1.00E-26	0.417488	1.518143	0.755	0.598	3.16E-22
BAZ2B	1.69E-18	0.4173331	1.517908	0.747	0.62	5.34E-14
SERF2	6.97E-161	0.417237	1.517762	1	1	2.20E-156
RPL30	3.17E-236	0.4169315	1.517299	1	1	9.99E-232
SPATC1L	1.24E-25	0.4168324	1.517148	0.238	0.08	3.91E-21
PSMD10	8.27E-40	0.4166914	1.516934	0.931	0.855	2.61E-35
TAPT1	8.36E-24	0.4166644	1.516893	0.53	0.336	2.64E-19
PIP4P2	8.75E-42	0.4165885	1.516778	0.813	0.584	2.76E-37
GRK6	5.30E-19	0.4163292	1.516385	0.564	0.402	1.67E-14
KCTD1	1.11E-18	0.4162534	1.51627	0.47	0.321	3.51E-14
RGS5	1.46E-09	0.4160747	1.515999	0.198	0.107	4.60E-05
HMGN4	1.35E-38	0.415977	1.515851	0.934	0.823	4.26E-34
CMTM3	2.58E-30	0.4158351	1.515636	0.857	0.706	8.14E-26
PRDX2	2.18E-55	0.4157012	1.515433	0.997	0.999	6.87E-51
SH3BP1	7.09E-32	0.4155815	1.515252	0.27	0.088	2.24E-27
MEX3A	1.14E-17	0.4155737	1.51524	0.197	0.078	3.58E-13
NAIF1	1.73E-14	0.4154771	1.515093	0.347	0.234	5.45E-10
MSL1	9.57E-39	0.4154036	1.514982	0.933	0.838	3.02E-34
GTF2A2	1.52E-44	0.4153577	1.514912	0.986	0.96	4.78E-40
CKB	3.91E-17	0.4153144	1.514847	0.685	0.549	1.23E-12
COG4	7.31E-33	0.4150812	1.514494	0.771	0.572	2.31E-28
LDOC1	3.60E-28	0.4150579	1.514458	0.782	0.624	1.13E-23
PRORP	5.85E-25	0.4148621	1.514162	0.618	0.432	1.85E-20
PHF3	1.88E-30	0.4148216	1.514101	0.852	0.707	5.92E-26
SNHG32	6.21E-58	0.4147147	1.513939	0.998	0.99	1.96E-53
NME1-NME2	3.26E-145	0.4146594	1.513855	1	1	1.03E-140
AAK1	1.39E-36	0.4146435	1.513831	0.926	0.837	4.37E-32
RIC1	7.96E-23	0.4143359	1.513365	0.58	0.396	2.51E-18
GLI1	8.24E-31	0.4141432	1.513074	0.15	0.02	2.60E-26
NUDCD3	1.99E-27	0.413995	1.51285	0.748	0.57	6.27E-23
SET	1.16E-129	0.4139865	1.512837	1	1	3.65E-125
TMEM25	3.21E-27	0.4139373	1.512762	0.397	0.195	1.01E-22
TXNDC17	7.49E-57	0.4138239	1.512591	0.969	0.914	2.36E-52

HOXA7	1.15E-18	0.4137997	1.512554	0.479	0.318	3.63E-14
PANK4	1.35E-25	0.4137981	1.512552	0.459	0.26	4.25E-21
GDE1	4.64E-45	0.4132053	1.511655	0.968	0.896	1.46E-40
ERI2	2.20E-17	0.4130557	1.511429	0.4	0.252	6.92E-13
BIVM	7.02E-26	0.4128923	1.511182	0.781	0.655	2.21E-21
SPECC1	5.85E-21	0.4125302	1.510635	0.881	0.826	1.85E-16
SAC3D1	8.22E-23	0.412487	1.51057	0.449	0.261	2.59E-18
C9orf64	2.93E-23	0.4124844	1.510566	0.573	0.397	9.25E-19
ACO1	6.19E-31	0.4124424	1.510503	0.943	0.885	1.95E-26
TRIO	1.65E-45	0.4121998	1.510136	0.988	0.963	5.22E-41
FRA10AC1	3.24E-30	0.4121056	1.509994	0.533	0.313	1.02E-25
MASTL	9.12E-15	0.4120027	1.509839	0.505	0.39	2.88E-10
XRCC4	3.71E-18	0.411817	1.509558	0.4	0.248	1.17E-13
THAP5	6.17E-24	0.4117903	1.509518	0.614	0.433	1.95E-19
TM2D1	8.30E-26	0.4117436	1.509447	0.851	0.754	2.62E-21
MADD	4.36E-23	0.4115439	1.509146	0.459	0.266	1.37E-18
PTCD3	5.74E-30	0.4115318	1.509128	0.831	0.683	1.81E-25
MYO1C	6.09E-93	0.4113901	1.508914	1	0.999	1.92E-88
EML4	7.38E-29	0.411232	1.508675	0.855	0.721	2.33E-24
DIAPH2	6.22E-18	0.4111587	1.508565	0.621	0.476	1.96E-13
ARRDC3	9.49E-26	0.4110966	1.508471	0.476	0.272	2.99E-21
NECAP2	9.66E-30	0.4107941	1.508015	0.903	0.813	3.05E-25
MAD2L2	2.14E-27	0.4107672	1.507974	0.765	0.613	6.76E-23
BCAS2	1.23E-34	0.4102017	1.507122	0.869	0.725	3.87E-30
PTPRJ	8.31E-18	0.4101522	1.507047	0.57	0.421	2.62E-13
CAD	8.54E-21	0.4099321	1.506715	0.63	0.464	2.70E-16
CUL4A	3.01E-46	0.4098355	1.50657	0.974	0.912	9.49E-42
RPL12	7.36E-180	0.4096158	1.506239	1	1	2.32E-175
AC116366.2	8.80E-40	0.4095068	1.506075	0.841	0.668	2.78E-35
TBC1D31	2.71E-21	0.4092132	1.505633	0.292	0.133	8.56E-17
COG1	4.34E-25	0.4091639	1.505559	0.672	0.48	1.37E-20
RPS7P11	1.29E-101	0.4089731	1.505271	0.992	0.951	4.07E-97
ATN1	1.29E-35	0.4087575	1.504947	0.953	0.897	4.06E-31
CLK2	2.98E-19	0.40875	1.504935	0.545	0.375	9.39E-15
MIEF2	2.66E-17	0.408494	1.50455	0.266	0.134	8.37E-13
ZNF274	6.25E-24	0.4083471	1.504329	0.376	0.194	1.97E-19
HSF2	1.51E-19	0.4082309	1.504154	0.396	0.227	4.77E-15
PRPSAP2	9.56E-28	0.4080186	1.503835	0.621	0.41	3.01E-23
RNF213	1.09E-25	0.4079523	1.503735	0.83	0.688	3.43E-21
DLG3	1.69E-17	0.4078748	1.503619	0.266	0.131	5.33E-13

CREB1	1.00E-34	0.4077239	1.503392	0.819	0.621	3.17E-30
BTBD6	7.41E-28	0.4076302	1.503251	0.82	0.669	2.34E-23
SERF1B	2.04E-35	0.4075655	1.503154	0.706	0.52	6.42E-31
LZIC	4.61E-33	0.4075386	1.503114	0.851	0.695	1.45E-28
MMS22L	3.55E-18	0.4075355	1.503109	0.658	0.523	1.12E-13
MRPS21	2.26E-52	0.4073741	1.502866	0.967	0.9	7.12E-48
INMT	2.49E-14	0.407272	1.502713	0.127	0.06	7.86E-10
PPP1R21	7.40E-20	0.4071054	1.502462	0.527	0.355	2.33E-15
AC087477.2	4.41E-32	0.406999	1.502303	0.158	0.023	1.39E-27
LTBP4	7.07E-27	0.4068025	1.502007	0.59	0.384	2.23E-22
MCM3	2.24E-17	0.4066278	1.501745	0.599	0.446	7.07E-13
AC010442.3	3.97E-22	0.4066254	1.501741	0.446	0.266	1.25E-17
FANCE	2.54E-21	0.4065857	1.501682	0.28	0.124	8.01E-17
CCDC84	2.54E-17	0.4064394	1.501462	0.402	0.258	8.02E-13
IKBKB	9.65E-25	0.4063833	1.501378	0.564	0.362	3.04E-20
DOLK	1.34E-27	0.4063654	1.501351	0.55	0.333	4.23E-23
TMEM200A	2.07E-17	0.4063256	1.501291	0.902	0.881	6.54E-13
AC118553.2	2.75E-32	0.4061658	1.501051	0.871	0.741	8.68E-28
TMEM160	1.50E-34	0.4061434	1.501018	0.546	0.314	4.72E-30
ORC5	3.17E-19	0.4059713	1.50076	0.538	0.363	1.00E-14
RTN4IP1	5.19E-20	0.4058712	1.500609	0.287	0.133	1.64E-15
CIBAR1	8.75E-24	0.4058711	1.500609	0.797	0.661	2.76E-19
ECI2	2.97E-48	0.4057149	1.500375	0.989	0.948	9.36E-44
NUP88	2.60E-28	0.4056039	1.500208	0.829	0.687	8.19E-24
STK4	8.66E-28	0.4055304	1.500098	0.827	0.682	2.73E-23
MBOAT1	1.78E-22	0.4054955	1.500046	0.236	0.103	5.62E-18

Downregulated genes in hAM-Muse cells than in hBM-Muse cells

GeneSymbol	p-value	avg_logFC	FC	pct.1	pct.2	p_val_adj
HSPA6	0	-5.154398	0.005774	0.018	0.669	0
EFEMP1	0	-5.014612	0.00664	0.055	0.975	0
IL6	0	-4.562931	0.010431	0.108	0.958	0
HSPA1A	0	-4.107433	0.01645	0.953	1	0
PTGS2	0	-3.941404	0.019421	0.027	0.855	0
ANGPTL4	0	-3.932262	0.019599	0.109	0.915	0
RRAD	0	-3.837944	0.021538	0.213	0.868	0
CYP1B1	0	-3.837333	0.021551	0.016	0.924	0
HAPLN1	0	-3.751253	0.023488	0.042	0.769	0
PTX3	0	-3.603653	0.027224	0.164	0.914	0
HSPA1B	0	-3.601969	0.02727	0.959	1	0
NFATC2	0	-3.435276	0.032217	0.021	0.865	0
FNDC1	0	-3.049893	0.047364	0.02	0.836	0
TM4SF1	0	-3.034601	0.048094	0.588	0.994	0
PPP1R14C	0	-3.008223	0.049379	0.011	0.841	0
PENK	0	-2.993358	0.050119	0.011	0.609	0
MEDAG	0	-2.9707	0.051267	0.261	0.923	0
JAG1	0	-2.91078	0.054433	0.194	0.934	0
RPL10P9	0	-2.66442	0.06964	0.1	0.971	0
INHBA	0	-2.652261	0.070492	0.964	1	0
VEGFA	0	-2.624388	0.072484	0.629	0.987	0
TNFAIP3	0	-2.619353	0.07285	0.142	0.832	0
IL1R1	0	-2.520424	0.080426	0.402	0.965	0
ST3GAL1	0	-2.39578	0.091102	0.682	0.963	0
AK4	0	-2.388569	0.091761	0.1	0.867	0
HAS1	0	-2.349401	0.095426	0.016	0.657	0
PMEPA1	0	-2.279472	0.102338	0.181	0.89	0
CYTL1	0	-2.264822	0.103848	0.001	0.643	0
PTHLH	0	-2.229727	0.107558	0.209	0.879	0
LMCD1	0	-2.227184	0.107832	0.403	0.902	0
HBEGF	0	-2.202848	0.110488	0.327	0.914	0
TIMP3	0	-2.162741	0.115009	0.931	1	0
TNFAIP6	0	-2.127843	0.119094	0.269	0.902	0
CEMIP	9.88E-233	-2.118848	0.12017	0.229	0.781	3.12E-228
LOXL3	0	-2.114131	0.120738	0.339	0.942	0
FGF1	1.86E-235	-2.103939	0.121975	0.484	0.82	5.87E-231
CHST11	0	-2.089515	0.123747	0.146	0.867	0

HSPH1	0	-2.081547	0.124737	0.997	0.999	0
DNAJB1	4.26E-301	-2.077097	0.125293	0.968	0.996	1.34E-296
KRT81	5.88E-89	-2.064309	0.126906	0.102	0.39	1.85E-84
KIAA1217	0	-2.064056	0.126938	0.075	0.751	0
GFPT2	0	-2.062319	0.127159	0.37	0.899	0
PGF	0	-2.057092	0.127825	0.123	0.821	0
BGN	0	-2.051704	0.128516	0.754	0.998	0
TGFBI	0	-2.044944	0.129387	0.972	1	0
ITGA11	0	-2.0375	0.130354	0.68	0.974	0
HK2	0	-2.00821	0.134229	0.333	0.905	0
HSPB8	0	-1.996494	0.135811	0.487	0.93	0
SEL1L3	0	-1.990204	0.136668	0.096	0.807	0
KCTD12	6.12E-265	-1.978443	0.138284	0.206	0.783	1.93E-260
IL11	9.60E-242	-1.976917	0.138496	0.324	0.793	3.03E-237
PTGES	4.61E-297	-1.969139	0.139577	0.218	0.811	1.46E-292
TET3	1.61831928	-1.968798	0.139625	0.264	0.829	0.00E+00
CCL2	1.77E-265	-1.965561	0.140077	0.202	0.815	5.59E-261
P3H2	6.20457519	-1.955657	0.141471	0.218	0.833	1.95698506181449e-3
PLPP3	0	-1.953717	0.141746	0.448	0.968	0
MAFB	8.20E-203	-1.946921	0.142713	0.065	0.528	2.59E-198
TGFB2	6.05008086	-1.945404	0.14293	0.264	0.86	1.90825600599205e-3
CFH	0	-1.922938	0.146177	0.195	0.849	0
SLC19A2	2.61898148	-1.922392	0.146257	0.144	0.782	8.26052950598169e-3
GALNT1	0	-1.918951	0.146761	0.945	0.996	0
ROR1	0	-1.894265	0.150429	0.022	0.628	0
AL139393.3	3.01E-225	-1.881781	0.152319	0.148	0.677	9.50E-221
HIPK2	0	-1.879357	0.152688	0.51	0.963	0
MTND1P23	0	-1.871149	0.153947	0.972	1	0
NR4A1	1.83E-189	-1.869605	0.154185	0.352	0.776	5.78E-185
CLDN14	1.20E-198	-1.865133	0.154876	0	0.349	3.78E-194
PNP	0	-1.865061	0.154887	0.697	0.998	0
DNAJA4	6.97E-168	-1.860771	0.155553	0.183	0.6	2.20E-163
LIF	1.53E-198	-1.817324	0.16246	0.019	0.435	4.81E-194
GDF6	3.28E-298	-1.799092	0.165449	0.008	0.532	1.04E-293
PLXNA4	1.02E-306	-1.79787	0.165651	0.019	0.591	3.22E-302
FOXC2	0.00E+00	-1.795511	0.166043	0.149	0.783	2.65E-304
MFAP5	6.41E-256	-1.789252	0.167085	0.113	0.689	2.02E-251
SMOX	2.08001636	-1.779444	0.168732	0.437	0.904	6.56057962943656e-3
STK38L	0	-1.765703	0.171066	0.452	0.91	0
NNMT	0	-1.75633	0.172677	0.825	1	0

BAG3	0	-1.753104	0.173235	0.951	0.993	0
FN1	0	-1.748213	0.174085	1	1	0
EMB	1.60E-281	-1.745794	0.174506	0.126	0.761	5.05E-277
DNAJB4	0	-1.742048	0.175161	0.771	0.981	0
LINC00511	6.36E-301	-1.734045	0.176569	0.209	0.761	2.01E-296
HEYL	8.20E-262	-1.733481	0.176668	0.011	0.495	2.58E-257
NFIB	2.62101570	-1.719077	0.179232	0.071	0.7	8.26694562507602e-3
TP53I11	0.00E+00	-1.70268	0.182195	0.328	0.887	3.28E-305
CRYAB	2.09E-237	-1.69394	0.183794	0.436	0.921	6.58E-233
DSP	2.60E-297	-1.689785	0.184559	0.851	0.98	8.21E-293
CXCL3	9.96E-101	-1.684706	0.185499	0.088	0.411	3.14E-96
MLF1	0	-1.675793	0.18716	0.326	0.884	0
SORBS2	1.75E-242	-1.661159	0.189919	0.077	0.604	5.51E-238
C3orf52	1.31E-258	-1.655843	0.190931	0.133	0.722	4.15E-254
EDIL3	0	-1.654839	0.191123	0.737	0.977	0
GPC6	5.06E-258	-1.651435	0.191775	0.224	0.819	1.60E-253
TENT5A	0	-1.648024	0.19243	0.688	0.975	0
NGF	5.83E-239	-1.630813	0.19577	0.19	0.781	1.84E-234
LRRC32	4.83E-189	-1.629552	0.196017	0.133	0.618	1.52E-184
RPS6KA2	1.33E-307	-1.628402	0.196243	0.308	0.902	4.20E-303
CREB5	1.19E-239	-1.628097	0.196303	0.063	0.604	3.77E-235
GRB10	1.27E-252	-1.62565	0.196784	0.428	0.857	4.00E-248
ENTPD7	3.55727265	-1.623053	0.197295	0.416	0.911	1.12199936655447e-3
EDNRA	5.54E-213	-1.620136	0.197872	0.156	0.686	1.75E-208
SHISAL1	1.18E-290	-1.610332	0.199821	0.068	0.682	3.74E-286
ITPRIP	0	-1.606758	0.200537	0.61	0.929	0
CRIP2	0	-1.599797	0.201937	0.471	0.983	0
TIAM1	6.70E-270	-1.5938	0.203152	0.073	0.613	2.11E-265
RORA	2.90E-262	-1.580446	0.205883	0.126	0.718	9.15E-258
PLOD2	0	-1.578423	0.2063	0.999	1	0
ELN	5.90E-158	-1.576783	0.206639	0.27	0.7	1.86E-153
NREP	0	-1.576362	0.206726	0.838	0.993	0
ATP10A	2.22E-265	-1.570958	0.207846	0.309	0.809	7.01E-261
C11orf87	3.00E-235	-1.566111	0.208856	0.016	0.482	9.45E-231
SLC41A2	1.22E-253	-1.558983	0.21035	0.124	0.698	3.84E-249
CRLF1	4.12E-162	-1.557218	0.210721	0.065	0.481	1.30E-157
NDRG1	0	-1.552186	0.211784	0.681	0.981	0
AL671762.1	0	-1.551165	0.212001	0.326	0.825	0
AC119674.2	2.75E-286	-1.549238	0.21241	0.234	0.845	8.68E-282
COL15A1	4.85E-130	-1.547955	0.212682	0.067	0.424	1.53E-125

ABL2	0	-1.547108	0.212863	0.879	0.99	0
RGS4	9.95E-179	-1.543169	0.213703	0.807	0.977	3.14E-174
UGDH	0	-1.537166	0.214989	0.968	1	0
ABI3BP	1.42E-246	-1.52554	0.217504	0.349	0.892	4.48E-242
CSGALNACT	6.01E-189	-1.506568	0.221669	0.044	0.486	1.90E-184
SPHK1	5.64E-308	-1.50234	0.222609	0.63	0.974	1.78E-303
PRUNE2	1.56E-232	-1.500591	0.222998	0.031	0.52	4.93E-228
CLCF1	5.75E-210	-1.496245	0.22397	0.421	0.815	1.81E-205
PTPRE	1.19E-195	-1.488269	0.225763	0.162	0.65	3.76E-191
TLE3	2.30E-262	-1.487473	0.225943	0.313	0.836	7.25E-258
FNIP2	1.00E-262	-1.483979	0.226734	0.506	0.888	3.16E-258
SUSD5	5.34E-240	-1.483561	0.226829	0.127	0.7	1.68E-235
CDH6	9.72E-189	-1.479362	0.227783	0.108	0.517	3.07E-184
SYNE1	4.49E-307	-1.478756	0.227921	0.686	0.969	1.42E-302
HAS2	6.35E-263	-1.478415	0.227999	0.485	0.948	2.00E-258
ARC	7.28E-164	-1.464303	0.231239	0.017	0.366	2.30E-159
MFSD2A	2.26E-188	-1.462708	0.231608	0.139	0.618	7.14E-184
ADAMTS2	0	-1.462375	0.231685	0.747	0.994	0
BHLHE40	6.49E-266	-1.461267	0.231942	0.604	0.944	2.05E-261
CCDC80	0	-1.449132	0.234774	0.996	1	0
HES4	2.55E-214	-1.444874	0.235776	0.217	0.686	8.06E-210
GASK1B	1.57E-197	-1.443759	0.236039	0.236	0.761	4.94E-193
UBASH3B	7.79E-272	-1.43862	0.237255	0.582	0.934	2.46E-267
ZFAND2A	1.16E-196	-1.438437	0.237298	0.482	0.861	3.66E-192
LAMA4	5.55E-283	-1.438329	0.237324	0.886	0.978	1.75E-278
KCNE4	4.84E-77	-1.436576	0.23774	0.398	0.653	1.53E-72
ADAMTSL1	4.06E-241	-1.427962	0.239797	0.166	0.765	1.28E-236
ADAMTS1	1.80E-203	-1.427732	0.239852	0.94	0.999	5.66E-199
SERPINE2	5.76E-294	-1.425858	0.240302	0.995	1	1.82E-289
RGS3	2.75E-291	-1.423229	0.240935	0.756	0.965	8.69E-287
CHST15	2.59E-191	-1.414446	0.24306	0.111	0.613	8.16E-187
ADGRL2	6.93E-240	-1.410917	0.24392	0.312	0.842	2.19E-235
ITGBL1	8.68928947	-1.404713	0.245437	0.694	0.993	2.74E-306
ACAN	5.08E-83	-1.401697	0.246179	0.093	0.314	1.60E-78
EMP1	0	-1.397778	0.247146	1	1	0
POSTN	2.61E-74	-1.396313	0.247508	0.856	0.926	8.25E-70
DOK1	3.71E-178	-1.395811	0.247632	0.359	0.755	1.17E-173
SNAI1	1.53E-110	-1.394792	0.247885	0.245	0.544	4.83E-106
C11orf96	7.19E-166	-1.390905	0.24885	0.163	0.637	2.27E-161
TES	1.19841874	-1.386162	0.250033	0.764	0.987	3.78E-306

SERPINE1	0	-1.384025	0.250568	0.998	1	0
MAFF	2.80E-303	-1.382231	0.251018	0.644	0.959	8.82E-299
KITLG	1.29E-264	-1.372248	0.253536	0.699	0.974	4.07E-260
XYLT1	6.68E-229	-1.369534	0.254225	0.414	0.881	2.11E-224
PIEZO2	3.70E-174	-1.361544	0.256265	0.019	0.388	1.17E-169
TSC22D3	3.05E-159	-1.355044	0.257936	0.479	0.843	9.61E-155
LRIG1	8.81E-163	-1.352525	0.258587	0.133	0.594	2.78E-158
PNMA2	1.55E-193	-1.348257	0.259693	0.119	0.528	4.87E-189
EGFLAM	2.21E-240	-1.342717	0.261135	0.011	0.464	6.98E-236
PDE3A	6.54E-173	-1.333771	0.263482	0.087	0.52	2.06E-168
TGFBR1	5.37E-230	-1.33342	0.263574	0.526	0.906	1.69E-225
ATP13A3	0	-1.329907	0.264502	0.862	0.994	0
KCNN4	5.44E-169	-1.322478	0.266474	0.058	0.482	1.71E-164
NFATC1	3.68E-174	-1.319131	0.267368	0.115	0.582	1.16E-169
TSC22D1	9.53E-198	-1.318194	0.267618	0.768	0.956	3.01E-193
B3GNT2	1.00E-200	-1.317178	0.26789	0.293	0.785	3.17E-196
PCOLCE2	6.19E-181	-1.316103	0.268178	0.102	0.594	1.95E-176
IGFBP3	7.82E-250	-1.31513	0.268439	0.969	0.996	2.47E-245
IRX3	1.34500551	-1.311987	0.269284	0.063	0.689	4.24E-307
COL5A3	4.80E-236	-1.303312	0.271631	0.127	0.692	1.52E-231
MEST	1.59E-157	-1.300612	0.272365	0.519	0.884	5.00E-153
KIF21A	4.16E-144	-1.300242	0.272466	0.207	0.618	1.31E-139
STK17B	6.91E-264	-1.298896	0.272833	0.542	0.952	2.18E-259
SFRP4	4.77E-114	-1.296924	0.273371	0.028	0.319	1.50E-109
AC245297.1	2.00E-265	-1.295046	0.273885	0.053	0.613	6.32E-261
CHSY1	2.75E-211	-1.291864	0.274758	0.841	0.953	8.69E-207
SMIM3	3.72E-210	-1.287093	0.276072	0.549	0.911	1.17E-205
DCLK1	1.11E-147	-1.280402	0.277926	0.082	0.49	3.50E-143
CHST3	2.30E-220	-1.279195	0.278261	0.597	0.906	7.26E-216
COL14A1	1.11E-137	-1.278797	0.278372	0.052	0.417	3.50E-133
SOX9	1.72E-156	-1.273996	0.279712	0.121	0.573	5.42E-152
ZNF503	8.89E-208	-1.273722	0.279788	0.404	0.829	2.80E-203
CPEB4	9.48E-178	-1.271832	0.280318	0.618	0.902	2.99E-173
GLIS3	4.27E-215	-1.263097	0.282777	0.663	0.908	1.35E-210
C1R	5.34E-228	-1.259235	0.283871	0.638	0.936	1.69E-223
VCAN	7.04E-287	-1.259121	0.283904	0.974	1	2.22E-282
LGR4	5.24E-177	-1.250508	0.286359	0.385	0.824	1.65E-172
AC134312.5	7.67E-114	-1.250209	0.286445	0.131	0.454	2.42E-109
LEPR	4.65E-139	-1.248539	0.286924	0.556	0.808	1.47E-134
JARID2	8.79E-155	-1.245931	0.287673	0.432	0.794	2.77E-150

ACKR3	2.51E-180	-1.240857	0.289136	0.005	0.35	7.93E-176
DOK5	2.37E-138	-1.238643	0.289777	0.021	0.345	7.46E-134
ANKH	1.78E-199	-1.238534	0.289809	0.555	0.911	5.62E-195
CRY1	8.99E-192	-1.237976	0.28997	0.464	0.854	2.84E-187
CCN4	5.04E-116	-1.234806	0.290891	0.173	0.568	1.59E-111
ARG2	2.06E-129	-1.23159	0.291828	0.121	0.505	6.49E-125
LGMN	2.69E-205	-1.229234	0.292516	0.534	0.927	8.48E-201
PKIB	1.70E-170	-1.225775	0.29353	0.045	0.426	5.35E-166
AL450405.1	0	-1.219397	0.295408	0.53	0.916	0
NDUFA4L2	7.24E-95	-1.217663	0.295921	0.063	0.342	2.28E-90
SERPING1	7.68E-138	-1.205872	0.299431	0.258	0.691	2.42E-133
PAPPA2	2.24E-158	-1.202544	0.300429	0.015	0.354	7.07E-154
HMOX1	2.06E-110	-1.201933	0.300612	0.58	0.851	6.48E-106
CCPG1	3.14E-267	-1.198266	0.301717	0.857	0.983	9.91E-263
DUXAP10	2.40E-178	-1.196997	0.3021	0.311	0.759	7.56E-174
NOTCH2	1.59E-300	-1.196634	0.30221	0.926	0.996	5.01E-296
LINC01119	4.16E-93	-1.195612	0.302519	0.141	0.46	1.31E-88
CXCL2	5.05E-25	-1.193663	0.303109	0.15	0.267	1.59E-20
GALNT5	5.76E-184	-1.1936	0.303128	0.383	0.856	1.82E-179
SULF2	3.76E-114	-1.192198	0.303553	0.059	0.381	1.19E-109
FOSB	1.40E-171	-1.188074	0.304808	0.708	0.959	4.41E-167
MOK	9.44E-172	-1.181793	0.306728	0.414	0.845	2.98E-167
ENG	0	-1.181703	0.306756	0.998	1	0
LOX	4.94065645	-1.180605	0.307093	1	1	1.55833245354788e-3
CHORDC1	4.64E-237	-1.178091	0.307866	0.912	0.978	1.46E-232
PDK1	2.38E-156	-1.177002	0.308201	0.308	0.746	7.51E-152
MAP2K3	5.95E-246	-1.176668	0.308304	0.937	0.989	1.88E-241
COL5A1	0	-1.173737	0.309209	0.999	1	0
CFI	1.76E-154	-1.171447	0.309918	0.069	0.476	5.56E-150
HOMER2	3.43E-146	-1.170595	0.310182	0.179	0.595	1.08E-141
PTGFRN	2.81E-126	-1.170336	0.310263	0.069	0.433	8.85E-122
RABGEF1	6.49E-241	-1.16762	0.311107	0.706	0.948	2.05E-236
PGM1	2.04E-179	-1.164813	0.311981	0.486	0.865	6.44E-175
NPR3	2.32E-111	-1.162958	0.31256	0.286	0.674	7.31E-107
AMOTL1	2.64E-180	-1.160135	0.313444	0.504	0.879	8.33E-176
LINC00968	3.25E-166	-1.159357	0.313688	0.007	0.338	1.02E-161
SLC7A5	8.47E-228	-1.158347	0.314005	0.792	0.972	2.67E-223
RASAL2	4.89E-185	-1.156594	0.314556	0.611	0.893	1.54E-180
DNAJA1	0	-1.152698	0.315784	0.998	1	0
SERINC5	4.42E-165	-1.152426	0.315869	0.448	0.806	1.39E-160

BAMBI	1.10E-122	-1.151686	0.316103	0.172	0.584	3.46E-118
ADORA2B	7.78E-173	-1.148057	0.317253	0.031	0.429	2.45E-168
IGFBP4	0	-1.145344	0.318114	1	1	0
SOWAHC	2.43E-121	-1.145084	0.318197	0.525	0.742	7.65E-117
FBXO32	8.97E-84	-1.134972	0.321431	0.24	0.574	2.83E-79
CXCL8	5.84E-34	-1.134526	0.321574	0.476	0.68	1.84E-29
AHSA1	1.28E-279	-1.13306	0.322046	0.963	0.999	4.05E-275
PGM2L1	3.77E-119	-1.129509	0.323192	0.349	0.709	1.19E-114
ENC1	9.27E-111	-1.126661	0.324114	0.516	0.824	2.92E-106
IER3	9.90E-126	-1.123454	0.325155	0.978	0.995	3.12E-121
LSP1	3.28E-108	-1.121376	0.325831	0.082	0.343	1.03E-103
OSBPL6	1.07E-133	-1.119777	0.326353	0.202	0.639	3.36E-129
PLAUR	2.48E-268	-1.115363	0.327796	0.973	1	7.82E-264
SGMS2	8.71E-185	-1.112395	0.32877	0.604	0.921	2.75E-180
NR4A3	1.97E-134	-1.111464	0.329077	0.031	0.365	6.23E-130
CRISPLD2	1.18E-149	-1.107729	0.330308	0.595	0.871	3.73E-145
AKR1C3	1.87E-118	-1.105733	0.330968	0.073	0.434	5.89E-114
SLC22A3	1.40E-175	-1.10459	0.331347	0	0.307	4.43E-171
IPMK	2.95E-113	-1.10399	0.331546	0.225	0.614	9.29E-109
MYC	1.90E-173	-1.102241	0.332126	0.759	0.945	5.98E-169
ZNF469	2.70E-120	-1.099962	0.332884	0.288	0.628	8.51E-116
COL6A2	0	-1.099681	0.332977	1	1	0
FAM20A	1.17E-141	-1.096957	0.333885	0.057	0.446	3.69E-137
CYTH3	2.07E-162	-1.094958	0.334554	0.637	0.894	6.53E-158
TPD52L1	5.70E-90	-1.093133	0.335165	0.101	0.387	1.80E-85
GAS6	7.56E-184	-1.09308	0.335182	0.931	0.995	2.39E-179
HSPA7	3.27E-120	-1.092489	0.335381	0.003	0.239	1.03E-115
DAAM2	8.60E-146	-1.089057	0.336534	0.134	0.529	2.71E-141
ZNFX1	1.65E-203	-1.086827	0.337285	0.8	0.97	5.21E-199
STK26	3.79E-136	-1.086109	0.337527	0.142	0.571	1.19E-131
PARD6G-AS1	6.02E-116	-1.085854	0.337613	0.017	0.289	1.90E-111
LAMC2	1.88E-100	-1.0846	0.338037	0.164	0.5	5.93E-96
AL355916.1	5.46E-145	-1.080325	0.339485	0.422	0.689	1.72E-140
SPTY2D1	1.58E-209	-1.077186	0.340552	0.784	0.964	4.99E-205
RLF	5.88E-146	-1.073779	0.341715	0.44	0.833	1.85E-141
IGFBP2	6.13E-70	-1.070383	0.342877	0.092	0.325	1.93E-65
IL1RL1	2.95E-60	-1.069133	0.343306	0.014	0.168	9.30E-56
VSTM4	4.98E-132	-1.06887	0.343396	0.34	0.705	1.57E-127
ANKRD1	1.34E-32	-1.067688	0.343802	0.135	0.276	4.23E-28
NTN4	1.36E-169	-1.066865	0.344085	0.578	0.921	4.29E-165

SFRP1	3.14E-100	-1.063534	0.345234	0.195	0.578	9.91E-96
SLIT3	8.34E-184	-1.062558	0.345571	0.236	0.782	2.63E-179
SLC1A3	1.65E-143	-1.060487	0.346287	0.02	0.349	5.21E-139
DYRK3	4.08E-157	-1.060116	0.346416	0.559	0.874	1.29E-152
TMEM100	1.41E-153	-1.057406	0.347356	0.006	0.309	4.46E-149
CCN2	5.89E-131	-1.057308	0.34739	0.998	1	1.86E-126
SH3BP5	9.29E-135	-1.057108	0.347459	0.422	0.803	2.93E-130
FZD1	3.83E-126	-1.056963	0.34751	0.391	0.767	1.21E-121
CLDN4	2.85E-50	-1.054663	0.34831	0.07	0.275	8.98E-46
DUXAP8	2.38E-129	-1.05352	0.348708	0.289	0.704	7.51E-125
ADGRL4	1.39E-147	-1.046752	0.351076	0.241	0.733	4.38E-143
GJA1	3.30E-239	-1.046524	0.351156	0.981	0.996	1.04E-234
EHD1	7.09E-190	-1.046242	0.351255	0.87	0.965	2.24E-185
MSX1	9.45E-113	-1.036712	0.354619	0.226	0.598	2.98E-108
ADAM12	2.06E-263	-1.035984	0.354877	0.987	0.995	6.50E-259
ASS1	1.04E-117	-1.03585	0.354924	0.262	0.688	3.29E-113
FBN1	0	-1.034993	0.355229	1	1	0
TGFB2-OT1	2.22E-187	-1.028054	0.357703	0.115	0.61	7.01E-183
JMJD6	1.49E-183	-1.026933	0.358104	0.781	0.948	4.69E-179
BAIAP2	3.29E-119	-1.026414	0.358289	0.467	0.739	1.04E-114
PDE1C	7.86E-127	-1.025845	0.358493	0.468	0.847	2.48E-122
ANK3	2.36E-111	-1.025286	0.358694	0.051	0.355	7.46E-107
AL354743.1	3.26E-170	-1.025178	0.358733	0	0.305	1.03E-165
KCTD11	4.65E-88	-1.022702	0.359622	0.339	0.632	1.47E-83
SLC35E4	3.96E-118	-1.022647	0.359642	0.3	0.68	1.25E-113
LOXL1	1.48E-260	-1.017171	0.361616	0.961	0.998	4.67E-256
DENND2C	1.18E-125	-1.016378	0.361903	0.147	0.496	3.73E-121
EBF1	3.24E-118	-1.016225	0.361959	0.563	0.83	1.02E-113
CGREF1	8.70E-164	-1.014973	0.362412	0.026	0.393	2.75E-159
IFI30	1.41E-169	-1.013508	0.362943	0.469	0.891	4.43E-165
BNIP3L	1.56E-258	-1.012949	0.363146	0.986	1	4.93E-254
TSKU	2.68E-124	-1.011482	0.36368	0.339	0.742	8.47E-120
SIX2	3.19E-218	-1.009178	0.364519	0.001	0.386	1.01E-213
RAPGEF2	5.54E-145	-1.008261	0.364853	0.614	0.894	1.75E-140
ZNF275	1.56E-110	-1.005628	0.365815	0.452	0.763	4.93E-106
HOMER1	3.65E-114	-1.003982	0.366417	0.291	0.685	1.15E-109
KDM6B	1.14E-126	-1.001871	0.367192	0.425	0.776	3.61E-122
RELT	1.24E-115	-1.000983	0.367518	0.182	0.573	3.92E-111
CLU	1.13E-65	-0.999454	0.36808	0.631	0.813	3.56E-61
ITGA5	3.88707109	-0.999253	0.368154	1	1	1.23E-307

GATA6	5.50E-122	-0.99899	0.368251	0.065	0.427	1.74E-117
CHMP1B	7.86E-198	-0.995119	0.36968	0.932	0.996	2.48E-193
CHRDL1	2.15E-131	-0.994526	0.369899	0.01	0.287	6.79E-127
SLC2A3	1.18E-137	-0.992719	0.370568	0.737	0.917	3.71E-133
DPYSL4	2.45E-154	-0.990609	0.371351	0.027	0.391	7.72E-150
CAMK1D	4.20E-107	-0.988405	0.37217	0.218	0.554	1.33E-102
APBB1IP	9.30E-122	-0.987578	0.372478	0.313	0.722	2.93E-117
SIX1	8.40E-105	-0.986779	0.372775	0.123	0.486	2.65E-100
SEC14L2	8.30E-105	-0.98665	0.372824	0.252	0.632	2.62E-100
NRG1	4.37E-97	-0.985112	0.373397	0.612	0.887	1.38E-92
EFNB2	1.06E-66	-0.97818	0.375995	0.406	0.653	3.34E-62
ULBP3	1.69E-122	-0.974289	0.377461	0.452	0.785	5.33E-118
PORCN	5.19E-86	-0.974099	0.377532	0.318	0.633	1.64E-81
AC027644.4	6.68E-198	-0.972534	0.378124	0.516	0.865	2.11E-193
ERRFI1	5.36E-150	-0.971875	0.378373	0.857	0.978	1.69E-145
MEGF6	4.12E-111	-0.971618	0.37847	0.099	0.463	1.30E-106
LHFPL2	7.47E-108	-0.97103	0.378693	0.398	0.741	2.36E-103
ETS1	5.28E-188	-0.965507	0.38079	0.925	0.986	1.67E-183
PTPRG	3.09E-149	-0.965227	0.380897	0.52	0.882	9.73E-145
EBF3	1.79E-121	-0.963877	0.381411	0.053	0.397	5.66E-117
PPP1R3B	1.51E-104	-0.963348	0.381613	0.325	0.661	4.75E-100
ZBTB43	5.16E-109	-0.96274	0.381845	0.519	0.826	1.63E-104
TENM2	3.86E-94	-0.961225	0.382424	0.22	0.606	1.22E-89
LINC00839	2.80E-109	-0.959635	0.383033	0.139	0.502	8.82E-105
RCL1	1.55E-97	-0.958651	0.38341	0.233	0.597	4.88E-93
DNAJC15	2.19E-234	-0.957312	0.383923	0.353	0.948	6.89E-230
SMOC1	4.28E-113	-0.956516	0.384229	0.003	0.225	1.35E-108
SDC2	1.35E-226	-0.955973	0.384438	0.964	0.999	4.25E-222
RNF19B	5.66E-91	-0.9555	0.38462	0.319	0.651	1.78E-86
ABHD2	1.11E-160	-0.953976	0.385206	0.856	0.969	3.50E-156
DACT1	8.22E-90	-0.953548	0.385371	0.238	0.591	2.59E-85
RFLNA	1.31E-86	-0.953293	0.385469	0.076	0.366	4.15E-82
CDC42SE1	1.38E-169	-0.953262	0.385482	0.726	0.922	4.34E-165
HSPD1	4.40E-308	-0.951297	0.38624	1	1	1.39E-303
ITPKC	8.50E-88	-0.950062	0.386717	0.298	0.608	2.68E-83
EFHD2	6.38E-187	-0.948917	0.38716	0.876	0.976	2.01E-182
ITGB8	1.35E-84	-0.947898	0.387555	0.162	0.417	4.26E-80
MAP2K1	6.42E-196	-0.946882	0.387949	0.827	0.976	2.03E-191
PID1	5.08E-94	-0.94392	0.389099	0.197	0.571	1.60E-89
LXN	1.78E-97	-0.94251	0.389649	0.718	0.882	5.62E-93

LINC01711	7.99E-126	-0.942172	0.38978	0.022	0.323	2.52E-121
ZNF654	1.18E-124	-0.939179	0.390949	0.49	0.823	3.71E-120
SYNM	3.89E-78	-0.937206	0.391721	0.111	0.41	1.23E-73
CYP27C1	6.88E-111	-0.936558	0.391975	0.134	0.409	2.17E-106
B4GALT1	1.50E-235	-0.935716	0.392305	0.977	1	4.74E-231
ESM1	6.59E-58	-0.933811	0.393053	0.073	0.303	2.08E-53
NEDD9	8.09E-96	-0.933176	0.393303	0.493	0.781	2.55E-91
ITGAV	1.98E-229	-0.932795	0.393453	0.985	0.999	6.25E-225
TGFB3	6.02E-117	-0.93261	0.393525	0.017	0.294	1.90E-112
EVA1A	4.09E-114	-0.932036	0.393751	0.753	0.915	1.29E-109
LYPD1	2.73E-83	-0.928886	0.394994	0.398	0.731	8.62E-79
NFASC	1.40E-95	-0.928786	0.395033	0.186	0.513	4.41E-91
CD200	6.72E-58	-0.927768	0.395435	0.1	0.343	2.12E-53
CDKN2B	2.60E-65	-0.927507	0.395538	0.245	0.522	8.19E-61
ATG101	1.62E-165	-0.926663	0.395873	0.83	0.969	5.11E-161
PDLIM3	1.20E-76	-0.926383	0.395983	0.092	0.314	3.78E-72
KLF4	5.58E-78	-0.925933	0.396162	0.377	0.704	1.76E-73
RASD1	1.94E-47	-0.925102	0.396491	0.092	0.285	6.13E-43
STAC	7.12E-95	-0.924677	0.396659	0.078	0.388	2.25E-90
NBPF14	2.56E-247	-0.922944	0.397348	0.963	0.998	8.08E-243
TNC	1.49E-167	-0.921771	0.397814	0.965	0.995	4.71E-163
HSPA4L	2.35E-93	-0.921705	0.39784	0.456	0.685	7.40E-89
DCN	1.20E-171	-0.921005	0.398119	0.955	0.998	3.79E-167
STC2	2.25E-132	-0.919159	0.398854	0.947	0.986	7.11E-128
AUTS2	3.98E-110	-0.915119	0.400469	0.198	0.594	1.26E-105
INSIG2	4.21E-103	-0.912085	0.401686	0.507	0.79	1.33E-98
TCF7	1.17E-88	-0.911991	0.401723	0.195	0.516	3.68E-84
BMP6	2.24E-87	-0.911125	0.402072	0.039	0.273	7.05E-83
KIAA0040	1.51E-119	-0.909243	0.402829	0.003	0.237	4.78E-115
SPAG9	1.27E-192	-0.908488	0.403133	0.904	0.989	4.00E-188
DEDD2	1.32E-64	-0.907288	0.403618	0.357	0.609	4.16E-60
LINC00882	1.40E-107	-0.903808	0.405024	0.142	0.448	4.43E-103
TINAGL1	6.37E-78	-0.903801	0.405027	0.125	0.396	2.01E-73
TSPAN2	5.06E-117	-0.903406	0.405187	0.043	0.368	1.60E-112
JCAD	1.44E-84	-0.902463	0.40557	0.22	0.561	4.56E-80
GLS	1.77E-214	-0.900705	0.406283	0.995	1	5.58E-210
QSOX1	3.79E-225	-0.899462	0.406788	0.988	1	1.20E-220
CHSY3	4.81E-71	-0.895369	0.408457	0.151	0.432	1.52E-66
MYLIP	8.31E-31	-0.894949	0.408628	0.343	0.417	2.62E-26
DEPTOR	1.30E-83	-0.894824	0.40868	0.037	0.284	4.11E-79

ELFN1	1.96E-102	-0.891647	0.40998	0.079	0.405	6.19E-98
BPGM	1.06E-120	-0.890367	0.410505	0.759	0.928	3.33E-116
PAPPA	1.00E-106	-0.887778	0.411569	0.692	0.945	3.17E-102
PLCE1	3.59E-89	-0.887415	0.411719	0.116	0.374	1.13E-84
MAP1B	3.05E-263	-0.887067	0.411862	0.998	1	9.61E-259
ALPL	3.04E-128	-0.885294	0.412593	0	0.235	9.60E-124
PLA2G4A	2.85E-103	-0.88429	0.413007	0.04	0.333	8.99E-99
UAP1	1.34E-187	-0.883507	0.413331	0.934	0.999	4.22E-183
FGFRL1	4.20E-118	-0.880762	0.414467	0.55	0.843	1.32E-113
IFITM10	1.72E-97	-0.87813	0.415559	0.143	0.495	5.42E-93
GXYLT2	2.31E-97	-0.878054	0.415591	0.228	0.603	7.28E-93
WWC1	3.33E-116	-0.877774	0.415707	0.056	0.381	1.05E-111
SERPINF1	1.44E-88	-0.875336	0.416722	0.639	0.863	4.55E-84
SYNPO	1.50E-79	-0.874824	0.416935	0.246	0.567	4.74E-75
ACAA2	1.10E-166	-0.874589	0.417034	0.722	0.972	3.47E-162
GPX3	7.86E-117	-0.873924	0.417311	0.045	0.369	2.48E-112
KDM3A	3.80E-95	-0.872352	0.417967	0.425	0.758	1.20E-90
KLHL21	5.25E-80	-0.871445	0.418346	0.549	0.758	1.66E-75
FAM180A	2.84E-97	-0.871315	0.418401	0.254	0.656	8.96E-93
ETV3	5.05E-106	-0.871284	0.418414	0.473	0.8	1.59E-101
NDEL1	6.82E-135	-0.86945	0.419182	0.774	0.921	2.15E-130
EPAS1	1.93E-156	-0.86853	0.419568	0.847	0.976	6.08E-152
PGK1	0	-0.867483	0.420007	1	1	0
ALDH1A3	4.23E-41	-0.866304	0.420503	0.284	0.535	1.34E-36
FYN	2.97E-95	-0.866029	0.420619	0.467	0.788	9.35E-91
ERMN	3.06E-124	-0.860902	0.422781	0.004	0.254	9.66E-120
PELI1	1.55E-73	-0.858993	0.423588	0.141	0.42	4.90E-69
P4HA3	2.25E-80	-0.858462	0.423814	0.18	0.507	7.10E-76
SLC12A8	2.28E-52	-0.85787	0.424064	0.285	0.49	7.19E-48
SNED1	2.30E-89	-0.853596	0.425881	0.057	0.336	7.26E-85
GEM	1.13E-44	-0.851664	0.426704	0.355	0.589	3.55E-40
DIRAS1	5.76E-90	-0.847242	0.428595	0.115	0.442	1.82E-85
GRPEL1	7.74E-156	-0.846856	0.428761	0.845	0.98	2.44E-151
SEMA7A	2.67E-107	-0.84615	0.429064	0.632	0.89	8.44E-103
KRT7	4.25E-17	-0.845498	0.429344	0.536	0.637	1.34E-12
DDX21	2.35E-188	-0.844894	0.429603	0.994	1	7.41E-184
DYRK2	2.83E-120	-0.844496	0.429774	0.787	0.916	8.91E-116
TUFT1	2.56E-49	-0.841441	0.431089	0.231	0.452	8.07E-45
SHISA2	2.03E-114	-0.841058	0.431254	0.018	0.275	6.41E-110
HAPLN3	7.96E-65	-0.839484	0.431933	0.153	0.432	2.51E-60

ROR1-AS1	4.46E-54	-0.837546	0.432771	0.175	0.325	1.41E-49
HSP90AB1	0	-0.837409	0.432831	1	1	0
LTBP2	5.41E-195	-0.83452	0.434083	0.994	1	1.71E-190
NBPF10	7.19E-223	-0.834479	0.434101	0.949	0.998	2.27E-218
NOTCH2NLC	8.82E-133	-0.831849	0.435244	0.528	0.848	2.78E-128
CPA4	2.13E-55	-0.831022	0.435604	0.153	0.403	6.71E-51
MIR222HG	1.93E-66	-0.830956	0.435633	0.146	0.412	6.10E-62
ODC1	9.72E-172	-0.828737	0.4366	0.932	0.999	3.07E-167
TCAF2	9.69E-102	-0.827736	0.437038	0.043	0.331	3.06E-97
CLMP	1.89E-198	-0.826061	0.43777	0.947	0.998	5.96E-194
VSIR	1.63E-73	-0.825708	0.437925	0.433	0.718	5.15E-69
RBP4	5.56E-81	-0.825109	0.438187	0.006	0.183	1.75E-76
KLF13	3.38E-80	-0.824749	0.438345	0.381	0.689	1.07E-75
SIPA1L1	7.58E-110	-0.823915	0.438711	0.576	0.886	2.39E-105
CABLES1	2.75E-88	-0.823139	0.439051	0.041	0.306	8.67E-84
EAF1	2.27E-87	-0.822674	0.439256	0.507	0.787	7.15E-83
EPSTI1	1.09E-90	-0.822428	0.439364	0.169	0.532	3.43E-86
SAMD8	5.10E-109	-0.821972	0.439564	0.589	0.866	1.61E-104
MIR210HG	1.17E-95	-0.821515	0.439765	0.068	0.371	3.70E-91
EYA2	1.05E-121	-0.819893	0.440479	0.003	0.241	3.31E-117
HIF1A	6.23E-268	-0.819529	0.440639	1	1	1.96E-263
IGF1	1.49E-29	-0.81671	0.441883	0.034	0.122	4.71E-25
PALM2AKAP	3.59E-197	-0.815583	0.442381	0.991	1	1.13E-192
DNAAF4-CCF	2.06E-161	-0.81115	0.444347	0.48	0.838	6.51E-157
FNBP1L	7.46E-70	-0.809721	0.444982	0.261	0.59	2.35E-65
ANK2	6.03E-68	-0.808085	0.445711	0.098	0.361	1.90E-63
PFKFB3	3.23E-72	-0.806247	0.446531	0.557	0.769	1.02E-67
TMEM59L	1.36E-90	-0.802572	0.448175	0.025	0.265	4.29E-86
PGM3	1.42E-171	-0.802278	0.448307	0.975	1	4.48E-167
COL8A1	3.56E-106	-0.801243	0.448771	0.976	0.996	1.12E-101
PEAK1	3.49E-116	-0.800783	0.448977	0.781	0.941	1.10E-111
MED13	4.58E-117	-0.800263	0.449211	0.855	0.963	1.44E-112
FAM83G	8.74E-68	-0.79837	0.450062	0.334	0.616	2.76E-63
NKX3-1	3.59E-102	-0.797741	0.450345	0.223	0.627	1.13E-97
MYO1E	1.93E-108	-0.797672	0.450376	0.679	0.911	6.09E-104
NXPH4	5.25E-108	-0.797175	0.4506	0.068	0.397	1.65E-103
CTSD	3.87E-174	-0.796458	0.450923	0.995	0.998	1.22E-169
FAM241A	2.76E-96	-0.795684	0.451273	0.536	0.803	8.69E-92
RRP12	1.93E-85	-0.794192	0.451946	0.546	0.795	6.08E-81
PODNL1	8.81E-81	-0.793581	0.452222	0.253	0.583	2.78E-76

DUXAP9	2.68E-100	-0.793231	0.452381	0.182	0.541	8.46E-96
CHI3L1	6.86E-33	-0.792181	0.452856	0.012	0.101	2.17E-28
SLC6A8	1.41E-73	-0.792035	0.452922	0.388	0.679	4.45E-69
EVL	1.07E-96	-0.791996	0.45294	0.26	0.643	3.36E-92
BNC1	6.05E-62	-0.789475	0.454083	0.277	0.572	1.91E-57
CCNYL1	2.60E-88	-0.789427	0.454105	0.482	0.772	8.19E-84
SEC24A	2.32E-116	-0.789286	0.454169	0.738	0.932	7.31E-112
RGS2	7.38E-33	-0.788857	0.454364	0.403	0.589	2.33E-28
CDH11	8.61E-189	-0.788313	0.454611	0.989	1	2.72E-184
NECTIN1	1.79E-71	-0.786578	0.455401	0.108	0.386	5.64E-67
BICC1	1.41E-107	-0.785054	0.456095	0.693	0.932	4.45E-103
MAP3K4	2.10E-69	-0.784766	0.456227	0.398	0.691	6.61E-65
KIF1B	5.96E-122	-0.784651	0.456279	0.913	0.989	1.88E-117
TPST2	6.30E-102	-0.783564	0.456775	0.788	0.942	1.99E-97
OSMR	1.35E-140	-0.781035	0.457932	0.85	0.972	4.27E-136
RCN3	1.67E-197	-0.78101	0.457943	0.995	1	5.26E-193
HSPA8	3.87E-286	-0.780089	0.458365	1	1	1.22E-281
CTH	1.04E-65	-0.778891	0.458915	0.079	0.324	3.28E-61
SVIL	1.06E-83	-0.77845	0.459117	0.16	0.488	3.34E-79
ADARB1	3.32E-62	-0.777866	0.459386	0.227	0.52	1.05E-57
COL12A1	2.09E-110	-0.777763	0.459432	0.998	0.998	6.59E-106
HTRA3	6.55E-59	-0.777689	0.459467	0.187	0.463	2.07E-54
DUSP1	3.73E-36	-0.777658	0.459481	0.978	0.969	1.18E-31
RASA2	9.85E-52	-0.776539	0.459996	0.532	0.692	3.11E-47
ARSI	3.38E-61	-0.775892	0.460293	0.318	0.62	1.07E-56
COL1A1	5.41E-252	-0.774837	0.460779	1	1	1.71E-247
SNHG7	1.22E-91	-0.772103	0.46204	0.469	0.797	3.84E-87
NET1	1.81E-64	-0.769814	0.463099	0.621	0.815	5.70E-60
HLX	3.47E-57	-0.769269	0.463352	0.332	0.589	1.09E-52
NOLC1	8.86E-133	-0.768113	0.463887	0.893	0.988	2.79E-128
TUBB2B	6.52E-139	-0.766976	0.464415	0.875	0.968	2.05E-134
FUCA1	2.06E-94	-0.766964	0.464421	0.04	0.318	6.50E-90
MICAL2	9.85E-142	-0.766028	0.464856	0.97	0.996	3.11E-137
PTGIS	9.63E-63	-0.764645	0.465499	0.089	0.261	3.04E-58
MSC	2.18E-60	-0.763245	0.466151	0.336	0.618	6.87E-56
GALNT2	5.93E-159	-0.763217	0.466164	0.934	0.995	1.87E-154
GNPNAT1	1.32E-134	-0.760786	0.467299	0.954	0.993	4.17E-130
FMO3	2.22E-38	-0.760723	0.467329	0.022	0.127	6.99E-34
ZNF672	4.08E-55	-0.760528	0.46742	0.371	0.62	1.29E-50
EPB41L4A-A	1.13E-99	-0.760505	0.46743	0.448	0.801	3.57E-95

PKNOX2	5.10E-104	-0.758574	0.468334	0.006	0.224	1.61E-99
HEG1	7.83E-104	-0.758215	0.468502	0.979	0.996	2.47E-99
GTPBP4	4.24E-147	-0.757335	0.468914	0.969	0.996	1.34E-142
TUBB2A	3.52E-128	-0.757012	0.469066	0.967	0.993	1.11E-123
AC007192.1	8.75E-119	-0.756853	0.46914	0.646	0.876	2.76E-114
SAMD4A	5.20E-74	-0.755007	0.470007	0.608	0.819	1.64E-69
PDGFRL	2.36E-68	-0.754724	0.47014	0.071	0.321	7.44E-64
FAM83H	4.02E-103	-0.754387	0.470299	0.036	0.309	1.27E-98
H19	2.94E-59	-0.750047	0.472345	0.002	0.125	9.28E-55
MYOCD	4.82E-38	-0.748933	0.472871	0.119	0.313	1.52E-33
DUSP6	7.92E-47	-0.748408	0.473119	0.777	0.791	2.50E-42
VLDLR	2.64E-66	-0.745896	0.474309	0.275	0.565	8.32E-62
DNAJB9	3.78E-57	-0.745485	0.474504	0.532	0.731	1.19E-52
LRRC8C	1.26E-74	-0.745441	0.474525	0.67	0.855	3.99E-70
EGLN1	4.33E-112	-0.745082	0.474695	0.739	0.918	1.37E-107
AMMECR1L	1.27E-85	-0.744172	0.475128	0.539	0.802	4.01E-81
NR4A2	1.39E-19	-0.74416	0.475133	0.201	0.332	4.39E-15
PTP4A1	1.25E-184	-0.743654	0.475374	0.984	1	3.93E-180
SH3BP5L	7.37E-80	-0.742754	0.475802	0.552	0.793	2.32E-75
WHRN	8.55E-74	-0.742186	0.476072	0.093	0.353	2.70E-69
AC022034.1	4.90E-60	-0.742116	0.476105	0.131	0.354	1.54E-55
KCNG1	2.13E-60	-0.741151	0.476565	0.43	0.657	6.72E-56
LIFR	4.31E-73	-0.740737	0.476763	0.255	0.595	1.36E-68
ENPP2	4.05E-170	-0.739606	0.477302	0.328	0.853	1.28E-165
BST1	1.01E-60	-0.739377	0.477411	0.527	0.755	3.18E-56
FAM219A	4.53E-63	-0.738629	0.477769	0.334	0.598	1.43E-58
DHRS9	3.12E-48	-0.738248	0.477951	0.011	0.132	9.84E-44
ATL1	1.45E-64	-0.738228	0.47796	0.138	0.404	4.57E-60
HIVEP3	2.50E-86	-0.736369	0.47885	0.068	0.341	7.87E-82
ATP1B1	8.91E-100	-0.736034	0.47901	0.941	0.978	2.81E-95
NPC1	1.48E-89	-0.734403	0.479792	0.518	0.818	4.68E-85
SRC	1.04E-73	-0.733338	0.480303	0.474	0.737	3.29E-69
NIPAL1	2.86E-62	-0.732153	0.480873	0.094	0.332	9.01E-58
ECM2	1.71E-60	-0.731674	0.481103	0.089	0.335	5.41E-56
NUMBL	1.66E-70	-0.730878	0.481486	0.543	0.742	5.23E-66
PLAGL1	8.33E-80	-0.730734	0.481555	0.544	0.802	2.63E-75
EDIL3-DT	1.37E-75	-0.727011	0.483352	0.203	0.488	4.33E-71
MIR31HG	1.62E-70	-0.725019	0.484315	0.21	0.538	5.10E-66
KCTD5	1.47E-79	-0.723543	0.485031	0.699	0.861	4.64E-75
TMEM217	1.18E-89	-0.722723	0.485428	0.032	0.285	3.72E-85

TUBB3	5.42E-121	-0.722698	0.485441	0.941	0.99	1.71E-116
FLI1	7.42E-62	-0.721485	0.48603	0.128	0.39	2.34E-57
FAM210A	5.20E-75	-0.721478	0.486033	0.531	0.789	1.64E-70
CRYBB2P1	2.93E-120	-0.721211	0.486163	0.549	0.88	9.24E-116
SIK3	1.20E-62	-0.720875	0.486326	0.353	0.635	3.77E-58
C1GALT1	1.72E-80	-0.720362	0.486576	0.62	0.862	5.41E-76
BMP1	8.76E-119	-0.720164	0.486672	0.845	0.976	2.76E-114
AL135905.2	2.64E-170	-0.719925	0.486789	0.966	0.998	8.34E-166
CD83	1.98E-48	-0.719662	0.486917	0.029	0.186	6.25E-44
GOLGA8A	3.94E-87	-0.719144	0.487169	0.53	0.803	1.24E-82
CBLB	1.13E-69	-0.719076	0.487202	0.614	0.841	3.56E-65
ANKRD37	2.40E-89	-0.718715	0.487378	0.153	0.508	7.58E-85
FAM162A	8.76E-92	-0.718339	0.487562	0.425	0.778	2.76E-87
EPDR1	8.21E-71	-0.714972	0.489206	0.604	0.836	2.59E-66
STAT3	3.80E-122	-0.714556	0.489409	0.917	0.99	1.20E-117
SLC41A1	3.73E-61	-0.714411	0.48948	0.394	0.671	1.18E-56
YWHAG	1.63E-224	-0.713802	0.489778	1	1	5.15E-220
SLC12A6	7.69E-66	-0.713588	0.489883	0.414	0.659	2.43E-61
NFIX	7.13E-116	-0.713248	0.49005	0.777	0.934	2.25E-111
SBSN	1.48E-68	-0.709616	0.491833	0.067	0.319	4.68E-64
SLC16A1	8.93E-111	-0.708618	0.492324	0.949	0.992	2.82E-106
ATF3	9.89E-24	-0.708148	0.492556	0.311	0.405	3.12E-19
ABHD5	7.90E-90	-0.708115	0.492572	0.665	0.903	2.49E-85
SOX11	9.01E-71	-0.707362	0.492943	0.169	0.366	2.84E-66
P2RY6	3.68E-86	-0.707332	0.492958	0.017	0.234	1.16E-81
SLC2A1	3.34E-83	-0.704861	0.494178	0.945	0.983	1.05E-78
LDHA	0	-0.704755	0.49423	1	1	0
EGR3	1.58E-58	-0.704271	0.494469	0.12	0.379	4.97E-54
SHB	3.51E-66	-0.703225	0.494986	0.613	0.797	1.11E-61
SPOCK1	5.24E-101	-0.702934	0.49513	0.972	0.998	1.65E-96
NKRF	4.91E-57	-0.702218	0.495485	0.306	0.594	1.55E-52
CYTOR	8.55E-138	-0.702164	0.495512	0.959	0.993	2.70E-133
FEM1B	9.08E-84	-0.701072	0.496053	0.839	0.927	2.86E-79
STC1	2.26E-37	-0.700618	0.496278	0.663	0.76	7.13E-33
EIF4A3	1.91E-113	-0.700497	0.496339	0.958	1	6.04E-109
FPR1	5.11E-72	-0.700428	0.496373	0.026	0.222	1.61E-67
ADD3	3.11E-111	-0.699965	0.496603	0.955	0.994	9.80E-107
LEMD3	2.66E-62	-0.698487	0.497337	0.513	0.73	8.40E-58
EPHB2	1.95E-58	-0.698486	0.497338	0.372	0.651	6.14E-54
GPR68	2.29E-83	-0.697919	0.49762	0.054	0.327	7.22E-79

OLFM2	1.08E-69	-0.696961	0.498097	0.037	0.251	3.41E-65
CYB561	3.21E-92	-0.695862	0.498645	0.432	0.796	1.01E-87
FEM1C	2.92E-64	-0.694848	0.49915	0.528	0.761	9.22E-60
PPFIA1	5.54E-82	-0.694793	0.499178	0.756	0.922	1.75E-77
AL445524.2	2.23E-98	-0.694312	0.499418	0.646	0.873	7.03E-94
GNL2	1.59E-99	-0.693852	0.499648	0.841	0.96	5.02E-95
SDE2	3.27E-69	-0.692668	0.50024	0.542	0.793	1.03E-64
HSP90AA1	4.10E-273	-0.69225	0.500449	1	1	1.29E-268
RHOD	1.38E-72	-0.691668	0.50074	0.19	0.512	4.34E-68
PRSS23	4.51E-155	-0.690441	0.501355	1	1	1.42E-150
THAP2	1.70E-69	-0.690244	0.501454	0.262	0.577	5.35E-65
MTSS1	6.12E-52	-0.690186	0.501483	0.055	0.242	1.93E-47
HIPK3	2.05E-67	-0.68978	0.501686	0.63	0.825	6.46E-63
INHBB	5.84E-81	-0.688712	0.502223	0.036	0.281	1.84E-76
BASP1	2.45E-186	-0.688447	0.502356	0.999	0.999	7.71E-182
PLXNA2	1.15E-46	-0.688285	0.502437	0.133	0.366	3.63E-42
ITGA10	3.39E-53	-0.6877	0.502731	0.109	0.299	1.07E-48
PRDM16	3.63E-94	-0.687624	0.502769	0.005	0.205	1.14E-89
CD2AP	7.88E-95	-0.687619	0.502772	0.814	0.941	2.49E-90
MCL1	1.73E-122	-0.686878	0.503144	0.992	0.996	5.45E-118
SLC25A36	4.55E-102	-0.686109	0.503532	0.808	0.954	1.43E-97
AFF4	3.62E-123	-0.686004	0.503584	0.958	0.998	1.14E-118
POLR1C	7.18E-63	-0.685853	0.50366	0.538	0.763	2.27E-58
IL1RAP	9.82E-52	-0.685412	0.503883	0.315	0.568	3.10E-47
MCC	6.49E-53	-0.684982	0.504099	0.23	0.504	2.05E-48
SLC39A14	5.39E-100	-0.68494	0.504121	0.927	0.992	1.70E-95
GGT5	4.40E-40	-0.684349	0.504418	0.159	0.342	1.39E-35
COL27A1	2.37E-50	-0.684146	0.504521	0.451	0.644	7.47E-46
ANXA10	2.28E-52	-0.68349	0.504852	0.022	0.182	7.18E-48
ARL4C	4.29E-58	-0.682972	0.505113	0.649	0.861	1.35E-53
CA12	2.58E-106	-0.681199	0.50601	0.825	0.975	8.13E-102
CHD7	4.50E-67	-0.679895	0.50667	0.072	0.271	1.42E-62
DLX5	4.07E-78	-0.679279	0.506982	0.002	0.156	1.28E-73
PYGL	1.07E-68	-0.679065	0.507091	0.556	0.809	3.36E-64
CARMN	4.07E-49	-0.678346	0.507456	0.433	0.638	1.28E-44
MELTF	2.44E-54	-0.677552	0.507858	0.071	0.277	7.69E-50
BNIP3	2.61E-88	-0.676978	0.508151	0.985	0.992	8.22E-84
GATAD2A	1.87E-93	-0.676908	0.508186	0.862	0.948	5.89E-89
HRH1	2.58E-63	-0.674979	0.509167	0.687	0.855	8.12E-59
NAP1L3	4.32E-72	-0.674274	0.509526	0.069	0.33	1.36E-67

CREG1	5.79E-66	-0.673517	0.509912	0.562	0.79	1.83E-61
GDNF	1.46E-38	-0.673501	0.50992	0.127	0.332	4.60E-34
SEMA3F	2.03E-133	-0.67341	0.509967	0.013	0.306	6.39E-129
USP38	1.33E-71	-0.672362	0.510502	0.639	0.853	4.20E-67
DNAJB6	4.17E-145	-0.672271	0.510548	0.998	0.999	1.32E-140
SNTB1	7.95E-50	-0.671691	0.510844	0.338	0.6	2.51E-45
AC009093.1	2.26E-35	-0.671147	0.511122	0.199	0.398	7.11E-31
RIPK4	1.77E-52	-0.669711	0.511857	0.01	0.145	5.59E-48
NALCN	3.13E-58	-0.669613	0.511907	0.137	0.381	9.87E-54
ADAMTS15	3.78E-91	-0.669068	0.512186	0.003	0.189	1.19E-86
MYCT1	2.18E-70	-0.668641	0.512404	0.002	0.144	6.86E-66
EIF1AD	1.76E-61	-0.667374	0.513054	0.479	0.742	5.54E-57
GOLM1	3.85E-100	-0.666041	0.513738	0.793	0.954	1.21E-95
TCP1	4.69E-201	-0.665822	0.513851	0.999	1	1.48E-196
ALKBH5	4.04E-95	-0.665484	0.514025	0.918	0.982	1.27E-90
LACC1	4.81E-35	-0.665449	0.514043	0.227	0.436	1.52E-30
VCAM1	8.97E-75	-0.664804	0.514374	0	0.141	2.83E-70
MT-TT	9.64E-189	-0.66475	0.514402	0.026	0.441	3.04E-184
AC092143.1	2.96E-107	-0.662879	0.515365	0.805	0.948	9.32E-103
UTP25	1.26E-58	-0.662564	0.515528	0.491	0.739	3.98E-54
TWNK	2.96E-50	-0.662232	0.515699	0.214	0.475	9.34E-46
AL139220.2	6.15E-63	-0.662192	0.515719	0.261	0.522	1.94E-58
PNMA1	4.11E-87	-0.66111	0.516278	0.925	0.978	1.30E-82
CNN3	4.82E-174	-0.660422	0.516633	1	1	1.52E-169
SBSPON	2.69E-58	-0.660211	0.516742	0.055	0.258	8.49E-54
TMEM233	1.95E-111	-0.659505	0.517107	0.018	0.281	6.15E-107
GOLT1B	3.06E-128	-0.658374	0.517692	0.976	0.995	9.66E-124
POLR3D	1.75E-81	-0.657666	0.518059	0.654	0.882	5.51E-77
NOTCH2NLA	2.30E-84	-0.657615	0.518085	0.471	0.76	7.25E-80
NOTCH2NLB	2.00E-104	-0.657518	0.518136	0.243	0.619	6.31E-100
UNC5B	1.19E-51	-0.657452	0.51817	0.589	0.779	3.76E-47
RRP7A	1.54E-85	-0.657294	0.518252	0.775	0.917	4.85E-81
PYURF	1.80E-79	-0.657266	0.518266	0.998	1	5.68E-75
INSIG1	1.42E-69	-0.656977	0.518416	0.845	0.964	4.49E-65
TNFRSF10B	7.59E-80	-0.656878	0.518467	0.817	0.926	2.39E-75
AKIRIN1	9.40E-123	-0.656668	0.518576	0.977	0.998	2.96E-118
FSTL3	1.07E-65	-0.656616	0.518603	0.689	0.866	3.39E-61
MED13L	3.29E-93	-0.655912	0.518969	0.834	0.968	1.04E-88
HSPB7	6.39E-33	-0.655841	0.519005	0.317	0.534	2.02E-28
MCUB	4.62E-70	-0.655005	0.51944	0.549	0.833	1.46E-65

SLC37A3	1.60E-58	-0.653652	0.520143	0.45	0.722	5.03E-54
TSLP	5.62E-62	-0.652093	0.520954	0.013	0.175	1.77E-57
RNF180	3.25E-63	-0.651988	0.521009	0.037	0.24	1.02E-58
COL6A1	6.84E-177	-0.651815	0.521099	1	1	2.16E-172
KREMEN1	1.27E-61	-0.651766	0.521125	0.3	0.525	4.00E-57
LAPTM5	2.47E-41	-0.649763	0.522169	0.053	0.224	7.81E-37
ANXA8	8.45E-69	-0.649727	0.522189	0.085	0.332	2.67E-64
AMD1	8.83E-117	-0.649093	0.52252	0.977	1	2.78E-112
DIPK2A	6.90E-51	-0.648804	0.52267	0.529	0.741	2.18E-46
TNFRSF1A	1.75E-99	-0.647535	0.523334	0.871	0.98	5.52E-95
FTSJ1	4.92E-105	-0.646126	0.524072	0.906	0.987	1.55E-100
TTPAL	4.82E-63	-0.64603	0.524122	0.641	0.823	1.52E-58
ALG2	3.43E-83	-0.644611	0.524867	0.773	0.917	1.08E-78
ADCY7	6.22E-67	-0.643864	0.525259	0.488	0.776	1.96E-62
NAV1	4.99E-117	-0.642655	0.525894	0.969	0.999	1.57E-112
AC010198.2	9.55E-54	-0.642002	0.526238	0.362	0.584	3.01E-49
ARL8B	1.79E-126	-0.640829	0.526856	0.982	0.999	5.63E-122
CXCL12	5.39E-81	-0.640159	0.527208	0.911	0.994	1.70E-76
NBPF20	2.03E-124	-0.63985	0.527372	0.945	0.993	6.39E-120
FHL1	6.85E-50	-0.639524	0.527544	0.714	0.836	2.16E-45
FNDC3B	5.61E-132	-0.638224	0.52823	0.993	0.999	1.77E-127
FAM47E-STE	1.31E-44	-0.636641	0.529067	0.294	0.507	4.12E-40
S1PR5	2.27E-42	-0.636409	0.529189	0.041	0.188	7.15E-38
EML1	2.49E-48	-0.636288	0.529253	0.516	0.722	7.87E-44
MGAM	4.74E-54	-0.636168	0.529317	0.045	0.24	1.49E-49
CSF1	2.50E-48	-0.6352	0.52983	0.554	0.752	7.87E-44
FEZ1	4.86E-56	-0.63415	0.530386	0.44	0.693	1.53E-51
VDR	5.04E-35	-0.633945	0.530495	0.304	0.499	1.59E-30
NBPF19	7.20E-134	-0.633256	0.53086	0.907	0.974	2.27E-129
NAF1	3.32E-48	-0.632933	0.531032	0.331	0.588	1.05E-43
JAM3	3.40E-68	-0.631217	0.531944	0.676	0.881	1.07E-63
PKP1	5.29E-85	-0.631148	0.531981	0.008	0.2	1.67E-80
AL139385.1	5.79E-39	-0.630038	0.532572	0.078	0.231	1.83E-34
PLXDC2	2.36E-39	-0.629852	0.532671	0.164	0.381	7.44E-35
DNAH10	2.29E-73	-0.629165	0.533037	0.016	0.201	7.23E-69
SLC30A1	1.04E-48	-0.627244	0.534062	0.61	0.777	3.28E-44
EOGT	6.83E-56	-0.625665	0.534905	0.522	0.761	2.15E-51
BTAF1	1.73E-55	-0.624865	0.535334	0.529	0.755	5.47E-51
VWA5A	6.57E-64	-0.623386	0.536126	0.025	0.212	2.07E-59
CERCAM	1.46E-99	-0.622142	0.536794	0.901	0.988	4.60E-95

GAA	9.28E-49	-0.621284	0.537254	0.267	0.531	2.93E-44
PMP22	1.56E-56	-0.620343	0.53776	0.724	0.869	4.92E-52
VGLL4	1.39E-68	-0.619655	0.53813	0.697	0.868	4.38E-64
HGF	2.16E-21	-0.619158	0.538398	0.07	0.182	6.82E-17
RIOK3	2.80E-92	-0.618683	0.538653	0.933	0.992	8.83E-88
FGFR1	7.36E-95	-0.617548	0.539265	0.963	0.996	2.32E-90
SEZ6L2	9.57E-43	-0.617393	0.539349	0.11	0.318	3.02E-38
ZNF423	8.28E-110	-0.616796	0.539671	0.011	0.255	2.61E-105
BEND7	8.21E-56	-0.616624	0.539764	0.148	0.355	2.59E-51
NEFM	1.35E-14	-0.616529	0.539815	0.224	0.354	4.27E-10
MAFK	3.95E-56	-0.616102	0.540045	0.554	0.73	1.24E-51
TAF13	4.24E-117	-0.615447	0.540399	0.934	0.994	1.34E-112
SARAF	3.14E-136	-0.615334	0.54046	0.994	1	9.89E-132
IL26	6.64E-99	-0.61525	0.540506	0	0.179	2.10E-94
PABPC4	8.03E-118	-0.615058	0.54061	0.979	0.996	2.53E-113
IGFBP7	1.73E-114	-0.613972	0.541197	1	0.998	5.46E-110
GAP43	3.72E-87	-0.613514	0.541445	0.009	0.21	1.17E-82
AC242842.3	2.44E-141	-0.613396	0.541509	0.857	0.953	7.70E-137
THBS2	2.14E-35	-0.613084	0.541678	0.953	0.968	6.74E-31
ZCCHC14	1.95E-42	-0.612773	0.541846	0.49	0.691	6.14E-38
SDC4	2.11E-88	-0.612362	0.542069	0.93	0.993	6.65E-84
MAN1A1	4.01E-54	-0.612279	0.542114	0.57	0.803	1.26E-49
COL1A2	1.99E-187	-0.612115	0.542203	1	1	6.29E-183
NAB1	9.18E-48	-0.612031	0.542249	0.416	0.656	2.89E-43
ABCA1	1.04E-27	-0.611974	0.542279	0.281	0.466	3.29E-23
CMKLR1	6.07E-50	-0.611579	0.542494	0.01	0.137	1.92E-45
KDM5B	1.76E-63	-0.610744	0.542947	0.865	0.941	5.55E-59
KCTD15	6.00E-64	-0.610361	0.543155	0.247	0.571	1.89E-59
C1S	6.90E-58	-0.609697	0.543515	0.629	0.843	2.18E-53
KCTD9	5.95E-97	-0.609592	0.543573	0.952	0.999	1.88E-92
TNFSF9	3.87E-88	-0.608899	0.54395	0.067	0.362	1.22E-83
GPRC5C	7.80E-59	-0.608842	0.54398	0.015	0.162	2.46E-54
PLAAT3	1.95E-58	-0.608371	0.544237	0.208	0.507	6.16E-54
FOXP1	4.86E-103	-0.608151	0.544356	0.954	0.99	1.53E-98
SESN3	7.96E-58	-0.607904	0.544491	0.028	0.209	2.51E-53
IL6ST	8.46E-141	-0.607845	0.544523	0.999	1	2.67E-136
PDE7B	9.58E-43	-0.607734	0.544584	0.13	0.347	3.02E-38
SPRY2	8.62E-41	-0.605901	0.545583	0.621	0.764	2.72E-36
GABRE	1.32E-38	-0.605788	0.545644	0.2	0.423	4.17E-34
RDH5	5.84E-54	-0.605161	0.545986	0.077	0.275	1.84E-49

C18orf25	2.47E-54	-0.604442	0.546379	0.561	0.775	7.80E-50
SCRG1	2.22E-46	-0.604118	0.546556	0.124	0.296	7.01E-42
MAPKAPK2	8.15E-81	-0.603866	0.546694	0.849	0.946	2.57E-76
ZNF12	5.05E-58	-0.603846	0.546705	0.435	0.727	1.59E-53
PPTC7	3.38E-61	-0.603799	0.546731	0.708	0.876	1.07E-56
COL16A1	1.33E-68	-0.602768	0.547295	0.693	0.893	4.18E-64
NFKB1	2.34E-48	-0.602461	0.547463	0.646	0.79	7.39E-44
MRPL18	1.21E-91	-0.601532	0.547971	0.968	0.995	3.82E-87
ARHGEF3	1.56E-50	-0.601488	0.547996	0.074	0.284	4.92E-46
BMT2	6.85E-45	-0.599763	0.548942	0.326	0.574	2.16E-40
ARL5B	3.92E-48	-0.599634	0.549012	0.64	0.808	1.24E-43
ANPEP	8.24E-146	-0.599446	0.549116	0.905	0.998	2.60E-141
BTG1	3.96E-99	-0.59942	0.54913	0.99	0.996	1.25E-94
BACE2	9.46E-70	-0.597708	0.550071	0.471	0.787	2.98E-65
AC092807.4	1.19E-41	-0.597196	0.550353	0.372	0.594	3.76E-37
SDSL	2.45E-38	-0.594481	0.551849	0.164	0.379	7.72E-34
ZNF503-AS2	4.21E-74	-0.594461	0.55186	0.027	0.241	1.33E-69
ARFGAP1	9.44E-62	-0.592396	0.553001	0.751	0.839	2.98E-57
GFOD1	1.17E-36	-0.592132	0.553147	0.182	0.362	3.69E-32
TSPYL2	3.35E-45	-0.590854	0.553854	0.49	0.719	1.06E-40
ZNF175	7.44E-41	-0.590016	0.554319	0.23	0.464	2.35E-36
ARHGAP26	2.66E-41	-0.589515	0.554596	0.213	0.386	8.38E-37
TGFBR3	1.34E-46	-0.589398	0.554661	0.246	0.517	4.22E-42
UBC	1.21E-121	-0.588957	0.554906	1	1	3.83E-117
COL5A2	2.02E-138	-0.588045	0.555412	1	1	6.37E-134
DCLK3	4.40E-80	-0.587449	0.555743	0.005	0.177	1.39E-75
AC254813.1	1.77E-87	-0.587198	0.555883	0.02	0.248	5.59E-83
LRP1	1.76E-96	-0.586141	0.55647	0.979	0.993	5.54E-92
BMP2	2.01E-15	-0.585375	0.556897	0.232	0.327	6.33E-11
DENND5A	7.69E-75	-0.585097	0.557052	0.825	0.952	2.43E-70
SMCO4	1.71E-51	-0.58475	0.557245	0.178	0.442	5.38E-47
ATP1A1	2.66E-116	-0.584683	0.557282	0.989	1	8.39E-112
ADAMTS7	3.89E-50	-0.584245	0.557526	0.238	0.504	1.23E-45
ULBP2	2.96E-39	-0.58169	0.558953	0.444	0.669	9.33E-35
SIM2	6.06E-56	-0.581479	0.559071	0.05	0.224	1.91E-51
AGPAT4	1.42E-37	-0.58089	0.5594	0.32	0.541	4.47E-33
ZNF121	1.04E-49	-0.579934	0.559935	0.581	0.743	3.28E-45
LAMC1	1.21E-134	-0.579495	0.560181	1	1	3.83E-130
BCL10	3.15E-73	-0.578993	0.560462	0.844	0.951	9.93E-69
SCN3A	8.48E-35	-0.577909	0.56107	0.021	0.107	2.67E-30

SLC4A7	6.24E-55	-0.577697	0.56119	0.723	0.879	1.97E-50
ABL1	5.63E-61	-0.576852	0.561664	0.68	0.861	1.78E-56
FLNC	1.79E-65	-0.576495	0.561864	0.997	0.996	5.63E-61
MIR22HG	6.42E-53	-0.575914	0.562191	0.657	0.859	2.03E-48
SPARC	7.42E-215	-0.574594	0.562934	1	1	2.34E-210
EGF	3.75E-30	-0.574488	0.562993	0.098	0.258	1.18E-25
HSPA9	2.38E-158	-0.572943	0.563864	0.998	1	7.51E-154
LUM	5.70E-49	-0.57287	0.563905	0.974	0.992	1.80E-44
SMIM14	5.19E-84	-0.572713	0.563993	0.934	0.99	1.64E-79
SFPQ	2.05E-150	-0.572163	0.564304	1	1	6.46E-146
RCAN1	6.61E-20	-0.571892	0.564456	0.825	0.885	2.09E-15
TBC1D10A	1.45E-33	-0.57188	0.564463	0.331	0.534	4.57E-29
MANBA	4.73E-39	-0.570953	0.564987	0.292	0.514	1.49E-34
DUSP5	4.20E-37	-0.569831	0.565621	0.533	0.735	1.33E-32
MAP1LC3B	4.12E-108	-0.567937	0.566693	0.986	1	1.30E-103
STARD4	2.40E-38	-0.567643	0.56686	0.681	0.82	7.57E-34
PAWR	1.68E-85	-0.566919	0.56727	0.958	0.994	5.29E-81
EYA4	7.65E-50	-0.565931	0.567831	0.023	0.179	2.41E-45
GADD45A	5.62E-61	-0.565691	0.567967	0.96	0.992	1.77E-56
HEPH	3.07E-34	-0.565652	0.56799	0.076	0.236	9.68E-30
ENO2	8.53E-42	-0.565512	0.568069	0.405	0.645	2.69E-37
CA13	2.14E-50	-0.56498	0.568372	0.063	0.257	6.74E-46
RBAK	8.76E-35	-0.564452	0.568672	0.327	0.538	2.76E-30
FUT11	3.37E-56	-0.564418	0.568691	0.567	0.788	1.06E-51
SLC35G2	4.01E-36	-0.564407	0.568697	0.491	0.659	1.27E-31
ZC3H12A	2.57E-32	-0.563747	0.569073	0.239	0.439	8.11E-28
ITPR1	2.00E-46	-0.563744	0.569074	0.121	0.288	6.31E-42
BCL7A	1.08E-28	-0.562914	0.569547	0.233	0.399	3.40E-24
IRAK2	6.90E-38	-0.562725	0.569654	0.092	0.278	2.18E-33
LINC02381	4.29E-56	-0.562129	0.569994	0.105	0.354	1.35E-51
PLAU	3.93E-19	-0.561869	0.570142	0.658	0.736	1.24E-14
TBPL1	7.32E-40	-0.560911	0.570689	0.584	0.747	2.31E-35
SPRED2	6.30E-34	-0.56036	0.571003	0.506	0.686	1.99E-29
FAR1	3.46E-63	-0.560184	0.571104	0.745	0.894	1.09E-58
DCBLD2	9.50E-88	-0.559906	0.571263	0.998	1	3.00E-83
SENP5	4.23E-60	-0.559905	0.571263	0.808	0.899	1.33E-55
TPI1	5.67E-280	-0.559282	0.571619	1	1	1.79E-275
FURIN	1.36E-68	-0.558288	0.572188	0.763	0.934	4.30E-64
PHLDB1	5.84E-54	-0.558018	0.572342	0.885	0.9	1.84E-49
RCOR1	2.68E-50	-0.556514	0.573204	0.635	0.811	8.44E-46

LMO2	3.87E-71	-0.55636	0.573292	0.003	0.15	1.22E-66
GAS1	3.66E-14	-0.556248	0.573356	0.165	0.279	1.15E-09
FNDC4	1.99E-38	-0.555675	0.573685	0.252	0.484	6.27E-34
ZNF212	6.80E-33	-0.555602	0.573727	0.186	0.391	2.14E-28
STX3	2.53E-24	-0.555491	0.573791	0.32	0.477	7.99E-20
FAM3C	3.81E-74	-0.555479	0.573797	0.873	0.971	1.20E-69
NBPF26	1.51E-112	-0.555031	0.574054	0.866	0.956	4.76E-108
PTGER2	2.88E-33	-0.554649	0.574274	0.285	0.505	9.10E-29
VPS37B	4.02E-53	-0.554175	0.574546	0.604	0.791	1.27E-48
GPI	1.78E-104	-0.553982	0.574657	0.996	1	5.61E-100
RRN3	2.35E-56	-0.553801	0.574761	0.664	0.86	7.40E-52
AC016588.2	2.15E-36	-0.553363	0.575013	0.196	0.4	6.78E-32
GAN	2.14E-28	-0.552729	0.575378	0.47	0.601	6.73E-24
EBLN3P	1.13E-57	-0.552327	0.575609	0.798	0.915	3.58E-53
SMILR	6.78E-101	-0.552309	0.575619	0.01	0.24	2.14E-96
LMNA	3.53E-244	-0.552167	0.575701	1	1	1.11E-239
GALNT18	1.71E-40	-0.552159	0.575706	0.045	0.205	5.38E-36
HSPG2	3.48E-74	-0.551993	0.575801	0.909	0.975	1.10E-69
BMPER	1.40E-31	-0.551529	0.576068	0.223	0.428	4.41E-27
ALDOA	0	-0.551165	0.576278	1	1	0
ABTB2	4.05E-33	-0.550986	0.576381	0.136	0.321	1.28E-28
PIM1	1.23E-22	-0.550566	0.576623	0.411	0.553	3.87E-18
ZNF395	9.99E-39	-0.550424	0.576705	0.411	0.634	3.15E-34
MAMLD1	1.51E-29	-0.550405	0.576716	0.106	0.266	4.75E-25
RHOB	2.91E-51	-0.550361	0.576742	0.99	0.996	9.17E-47
LINC01133	3.55E-105	-0.549275	0.577368	0.044	0.344	1.12E-100
DISP2	6.94E-53	-0.549105	0.577467	0.284	0.446	2.19E-48
DLX2	4.01E-24	-0.547982	0.578116	0.125	0.266	1.26E-19
PDCD4	1.14E-49	-0.54698	0.578695	0.611	0.808	3.61E-45
SMURF1	3.90E-36	-0.546957	0.578708	0.422	0.635	1.23E-31
MAP3K8	1.98E-26	-0.545806	0.579375	0.249	0.436	6.23E-22
TAGLN2	1.01E-168	-0.545485	0.579561	1	1	3.18E-164
PPAN	2.09E-48	-0.545329	0.579651	0.373	0.64	6.58E-44
ADAMTS12	3.25E-27	-0.544743	0.579991	0.245	0.427	1.02E-22
GTF3C4	3.38E-50	-0.544231	0.580288	0.7	0.853	1.07E-45
CTSZ	9.76E-91	-0.544188	0.580313	0.953	0.996	3.08E-86
SYNDIG1	2.75E-81	-0.544001	0.580421	0.005	0.18	8.67E-77
CDK17	4.64E-41	-0.543901	0.58048	0.505	0.712	1.46E-36
PKDCC	1.57E-40	-0.543533	0.580693	0.068	0.247	4.96E-36
BPNT2	1.87E-92	-0.543457	0.580737	0.982	0.998	5.90E-88

KAZALD1	7.50E-30	-0.542542	0.581269	0.164	0.325	2.37E-25
WSB1	7.13E-68	-0.542353	0.581379	0.974	0.993	2.25E-63
CD276	1.05E-77	-0.542229	0.581451	0.943	0.987	3.32E-73
SESTD1	2.87E-46	-0.542053	0.581553	0.429	0.694	9.06E-42
PLCXD1	4.11E-30	-0.541736	0.581737	0.431	0.567	1.30E-25
AC093512.2	0	-0.54173	0.581741	1	1	0
CCL26	9.83E-30	-0.540718	0.58233	0.122	0.267	3.10E-25
PAX9	1.41E-61	-0.538928	0.583374	0.007	0.152	4.45E-57
ARL4D	1.13E-22	-0.538199	0.583798	0.383	0.514	3.55E-18
NRXN2	2.96E-81	-0.538069	0.583875	0.023	0.245	9.34E-77
RAB11FIP1	5.10E-27	-0.538065	0.583877	0.289	0.453	1.61E-22
BYSL	3.50E-43	-0.537065	0.584461	0.592	0.781	1.10E-38
STK32B	1.03E-53	-0.537064	0.584462	0.034	0.216	3.25E-49
ARSB	1.04E-45	-0.536572	0.584749	0.549	0.752	3.29E-41
YRDC	1.87E-51	-0.535598	0.585319	0.536	0.79	5.91E-47
FSTL1	1.99E-241	-0.535294	0.585497	1	1	6.28E-237
PIGT	5.35E-69	-0.53513	0.585593	0.935	0.977	1.69E-64
LMLN	2.71E-33	-0.535041	0.585645	0.378	0.571	8.54E-29
PRKAB2	3.03E-44	-0.534781	0.585798	0.643	0.826	9.56E-40
CLSTN1	1.21E-84	-0.534389	0.586027	0.93	0.989	3.81E-80
C2orf27A	1.12E-36	-0.533486	0.586557	0.376	0.601	3.52E-32
PSAP	7.90E-138	-0.533382	0.586617	1	1	2.49E-133
MUC1	3.23E-40	-0.532717	0.587008	0.072	0.249	1.02E-35
TSPAN18	2.52E-40	-0.532196	0.587314	0.02	0.147	7.95E-36
CSRNP1	4.69E-30	-0.532136	0.587349	0.536	0.669	1.48E-25
COQ10B	6.26E-63	-0.531707	0.587601	0.798	0.923	1.97E-58
STIP1	1.83E-96	-0.531052	0.587986	0.994	0.999	5.78E-92
PTBP2	7.29E-36	-0.530503	0.588309	0.278	0.494	2.30E-31
ITGA7	2.89E-42	-0.530305	0.588425	0.026	0.163	9.12E-38
FHL3	7.24E-40	-0.530296	0.588431	0.502	0.698	2.28E-35
SERPINH1	1.10E-175	-0.52983	0.588705	1	1	3.47E-171
CDO1	6.17E-42	-0.529699	0.588782	0.022	0.157	1.95E-37
ELOA	1.23E-61	-0.528737	0.589349	0.868	0.94	3.87E-57
ZNF317	7.76E-32	-0.52867	0.589388	0.356	0.536	2.45E-27
CSNK1G3	1.09E-52	-0.527643	0.589994	0.757	0.89	3.45E-48
TRABD2B	5.32E-90	-0.527237	0.590234	0	0.163	1.68E-85
EXOC8	1.30E-28	-0.527138	0.590292	0.325	0.484	4.11E-24
LINC01128	2.41E-29	-0.527136	0.590293	0.237	0.428	7.61E-25
TNS1	3.32E-59	-0.527044	0.590348	0.907	0.964	1.05E-54
CXCL16	1.56E-47	-0.526689	0.590557	0.039	0.192	4.92E-43

GDF5	1.30E-26	-0.52668	0.590563	0.065	0.191	4.11E-22
AL118506.1	1.35E-43	-0.524473	0.591867	0.105	0.311	4.24E-39
ARRDC4	3.47E-43	-0.521777	0.593465	0.101	0.311	1.09E-38
CHD1	2.14E-41	-0.521763	0.593474	0.722	0.869	6.77E-37
LENG8	7.29E-48	-0.521367	0.593708	0.604	0.789	2.30E-43
GBP3	1.07E-29	-0.521289	0.593754	0.373	0.582	3.39E-25
ZNF267	2.36E-39	-0.521118	0.593856	0.229	0.471	7.43E-35
BAALC	2.48E-75	-0.520283	0.594352	0.216	0.56	7.81E-71
PPP1R15A	1.14E-41	-0.52025	0.594372	0.973	0.992	3.59E-37
ALDH1B1	2.81E-30	-0.520245	0.594375	0.606	0.753	8.86E-26
USP12	4.35E-42	-0.519788	0.594647	0.613	0.797	1.37E-37
WDR45B	5.69E-58	-0.519328	0.59492	0.821	0.893	1.79E-53
EFNB1	1.19E-32	-0.519306	0.594933	0.173	0.365	3.76E-28
EVI5	9.40E-59	-0.518907	0.595171	0.822	0.947	2.96E-54
NBPF9	1.34E-94	-0.517887	0.595778	0.912	0.98	4.22E-90
SLC16A3	3.26E-86	-0.517758	0.595855	0.996	0.999	1.03E-81
SNHG15	1.59E-52	-0.517079	0.59626	0.493	0.761	5.03E-48
EOLA2	2.28E-40	-0.515853	0.596991	0.557	0.735	7.18E-36
RABGGTB	1.68E-63	-0.515829	0.597005	0.899	0.968	5.31E-59
CDC42EP2	3.18E-36	-0.515829	0.597006	0.43	0.616	1.00E-31
CSGALNACT	2.62E-40	-0.515664	0.597104	0.594	0.796	8.25E-36
CEP85L	1.36E-37	-0.515598	0.597143	0.382	0.615	4.29E-33
SLC25A32	1.53E-58	-0.515264	0.597343	0.79	0.918	4.81E-54
RFX3	1.51E-30	-0.515103	0.597439	0.104	0.275	4.75E-26
CREB3L1	2.89E-81	-0.515014	0.597492	0.994	0.998	9.12E-77
LZTFL1	1.01E-30	-0.514025	0.598083	0.409	0.598	3.19E-26
ARHGAP5	2.23E-64	-0.514004	0.598096	0.934	0.984	7.04E-60
ENO1	3.31E-224	-0.513579	0.59835	1	1	1.04E-219
OGA	9.62E-61	-0.513385	0.598466	0.917	0.978	3.03E-56
RNF122	1.45E-49	-0.513095	0.59864	0.068	0.246	4.58E-45
TRAF3IP2	3.91E-30	-0.512491	0.599002	0.589	0.722	1.23E-25
KCNMA1	1.45E-137	-0.512362	0.599079	0.359	0.841	4.58E-133
FOXF2	8.12E-27	-0.512144	0.599209	0.097	0.251	2.56E-22
PRR7	4.79E-40	-0.511779	0.599428	0.214	0.445	1.51E-35
SIDT2	2.53E-38	-0.510547	0.600167	0.575	0.758	7.97E-34
CREB3L2	2.18E-63	-0.50981	0.60061	0.955	0.989	6.88E-59
SOBP	5.76E-54	-0.509204	0.600974	0.037	0.223	1.82E-49
TGFB1	5.68E-85	-0.508826	0.601201	0.939	0.993	1.79E-80
CD59	1.13E-174	-0.508523	0.601383	1	1	3.55E-170
CD74	8.52E-34	-0.508352	0.601486	0.042	0.181	2.69E-29

EREG	1.20E-22	-0.507959	0.601723	0.08	0.151	3.78E-18
THY1	1.39E-130	-0.507158	0.602204	0.713	0.986	4.39E-126
EIF3B	9.75E-88	-0.506375	0.602676	0.988	0.999	3.07E-83
CTDP1	2.40E-34	-0.506104	0.60284	0.376	0.585	7.58E-30
RUNDC1	4.95E-32	-0.505397	0.603266	0.516	0.651	1.56E-27
SPRY4	3.26E-37	-0.505273	0.603341	0.526	0.739	1.03E-32
ETF1	7.95E-111	-0.505215	0.603376	0.997	1	2.51E-106
AC112220.2	9.46E-28	-0.504645	0.60372	0.267	0.451	2.98E-23
BMPR2	2.64E-63	-0.504465	0.603828	0.983	0.998	8.32E-59
ENDOD1	5.70E-36	-0.503965	0.60413	0.62	0.802	1.80E-31
RELB	3.08E-31	-0.503787	0.604238	0.174	0.337	9.72E-27
CNN1	2.60E-23	-0.503246	0.604565	0.702	0.701	8.20E-19
KIAA0895	4.76E-64	-0.503012	0.604707	0.017	0.193	1.50E-59
FAM20C	2.75E-37	-0.502968	0.604733	0.69	0.86	8.67E-33
GOLGB1	2.42E-65	-0.502643	0.60493	0.917	0.983	7.64E-61
DPH2	7.47E-29	-0.5026	0.604956	0.321	0.514	2.36E-24
TOM1	5.21E-43	-0.502539	0.604993	0.615	0.787	1.64E-38
LRRFIP1	2.70E-68	-0.502508	0.605012	1	0.999	8.50E-64
RDH8	1.88E-63	-0.501367	0.605702	0.006	0.149	5.92E-59
PTK2B	1.86E-35	-0.501195	0.605806	0.075	0.222	5.86E-31
C15orf39	2.02E-27	-0.50108	0.605876	0.329	0.522	6.37E-23
UBE2O	1.07E-31	-0.500991	0.60593	0.45	0.6	3.37E-27
PFKL	1.31E-55	-0.500099	0.606471	0.843	0.944	4.12E-51
LTBP3	2.36E-56	-0.50007	0.606488	0.741	0.904	7.46E-52
PGAM1	1.48E-210	-0.500038	0.606507	1	1	4.67E-206
PTPN3	8.24E-47	-0.500006	0.606527	0.085	0.237	2.60E-42
EMP2	6.49E-46	-0.499945	0.606564	0.77	0.923	2.05E-41
ARHGAP31	2.48E-31	-0.499403	0.606893	0.55	0.694	7.83E-27
GAREM1	3.61E-41	-0.499314	0.606947	0.029	0.174	1.14E-36
SEPTIN6	6.21E-35	-0.49858	0.607393	0.278	0.512	1.96E-30
MBNL2	7.17E-55	-0.497415	0.6081	0.951	0.984	2.26E-50
NGEF	3.24E-45	-0.497384	0.60812	0.048	0.224	1.02E-40
JMJD1C	1.60E-21	-0.49725	0.608201	0.773	0.86	5.06E-17
FAM126A	8.75E-64	-0.497093	0.608296	0.934	0.988	2.76E-59
SYNJ2	4.92E-60	-0.495277	0.609402	0.858	0.965	1.55E-55
NDN	3.73E-46	-0.494725	0.609738	0.387	0.67	1.18E-41
FADS3	8.68E-46	-0.494472	0.609893	0.778	0.899	2.74E-41
NOCT	4.49E-34	-0.493841	0.610278	0.2	0.365	1.42E-29
FGFR2	7.11E-42	-0.49327	0.610626	0.165	0.324	2.24E-37
MBOAT2	5.11E-47	-0.492821	0.610901	0.9	0.963	1.61E-42

PROCR	9.32E-31	-0.492379	0.61117	0.543	0.718	2.94E-26
IKZF5	9.56E-28	-0.492196	0.611283	0.344	0.544	3.01E-23
SEMA3C	4.84E-38	-0.491884	0.611474	0.889	0.956	1.53E-33
MORC4	1.05E-33	-0.491734	0.611565	0.678	0.773	3.31E-29
JUND	5.55E-49	-0.491284	0.61184	0.96	0.966	1.75E-44
PER2	7.64E-26	-0.491213	0.611884	0.284	0.464	2.41E-21
ZNF587B	1.01E-34	-0.490926	0.612059	0.123	0.303	3.18E-30
SLC35E1	1.96E-50	-0.490915	0.612066	0.872	0.959	6.19E-46
PDZD8	9.65E-52	-0.490772	0.612154	0.847	0.936	3.04E-47
TNK2	6.85E-35	-0.490721	0.612185	0.287	0.434	2.16E-30
MYO10	6.13E-45	-0.4901	0.612565	0.987	0.994	1.93E-40
NOP53	1.98E-74	-0.489638	0.612848	0.943	0.987	6.23E-70
UGCG	6.46E-57	-0.48959	0.612878	0.943	0.99	2.04E-52
ZEB2	8.57E-37	-0.48943	0.612976	0.742	0.865	2.70E-32
RTL6	5.08E-29	-0.489332	0.613036	0.464	0.659	1.60E-24
NRP2	1.07E-48	-0.488931	0.613282	0.884	0.962	3.37E-44
FLRT3	3.35E-40	-0.488915	0.613292	0.023	0.155	1.06E-35
MN1	8.53E-50	-0.488794	0.613366	0.033	0.201	2.69E-45
LMO4	8.52E-38	-0.488746	0.613395	0.922	0.974	2.69E-33
AC099066.2	2.63E-57	-0.488577	0.613499	0.035	0.227	8.30E-53
BIN3	5.99E-28	-0.488322	0.613655	0.583	0.687	1.89E-23
SLC25A4	1.06E-30	-0.488289	0.613675	0.535	0.698	3.33E-26
COBLL1	5.06E-26	-0.488216	0.61372	0.217	0.391	1.60E-21
DGKI	1.30E-43	-0.487629	0.614081	0.353	0.625	4.09E-39
AP000944.5	1.46E-33	-0.487516	0.61415	0.449	0.622	4.60E-29
CDK6	7.83E-47	-0.487463	0.614182	0.943	0.982	2.47E-42
ZNF668	1.24E-30	-0.487338	0.61426	0.346	0.537	3.90E-26
PLBD2	5.73E-38	-0.487166	0.614365	0.619	0.777	1.81E-33
ESYT2	2.13E-58	-0.486941	0.614503	0.889	0.958	6.73E-54
EXT2	5.72E-70	-0.486878	0.614542	0.968	0.996	1.81E-65
TXNL4B	1.34E-29	-0.486758	0.614616	0.369	0.556	4.23E-25
SIRPA	2.02E-37	-0.486638	0.614689	0.484	0.697	6.36E-33
BEND5	1.26E-81	-0.486025	0.615066	0.001	0.159	3.98E-77
ECH1	1.16E-42	-0.485856	0.61517	0.624	0.831	3.66E-38
ULK4	3.63E-32	-0.485379	0.615464	0.091	0.258	1.14E-27
TRO	1.20E-27	-0.484914	0.61575	0.073	0.216	3.79E-23
ASAP2	2.96E-39	-0.484726	0.615866	0.764	0.833	9.35E-35
UBE2H	8.59E-64	-0.484542	0.615979	0.954	0.993	2.71E-59
POFUT2	5.55E-44	-0.484152	0.616219	0.686	0.85	1.75E-39
PLAC9	1.26E-84	-0.483794	0.61644	0.096	0.41	3.96E-80

SLC30A7	5.38E-57	-0.483697	0.6165	0.895	0.974	1.70E-52
KDM2A	4.84E-46	-0.483449	0.616653	0.872	0.944	1.53E-41
RBM27	1.23E-43	-0.482461	0.617263	0.809	0.918	3.89E-39
SCAND2P	1.89E-27	-0.482041	0.617522	0.222	0.379	5.95E-23
MAK16	1.89E-32	-0.481545	0.617828	0.635	0.788	5.95E-28
PPP2CB	1.26E-83	-0.481144	0.618076	0.996	1	3.97E-79
AC015922.3	3.11E-36	-0.481046	0.618137	0.121	0.317	9.80E-32
	3.65E-98	-0.480871	0.618244	1	1	1.15E-93
DUSP4	3.22E-22	-0.480733	0.61833	0.067	0.183	1.02E-17
RBM12	3.69E-48	-0.480263	0.618621	0.839	0.927	1.16E-43
GALNT10	3.63E-60	-0.479749	0.618939	0.958	0.995	1.14E-55
RLIM	4.00E-42	-0.479143	0.619314	0.745	0.875	1.26E-37
B4GALT5	2.05E-38	-0.47803	0.620003	0.6	0.783	6.46E-34
ACLY	3.93E-95	-0.477522	0.620319	0.999	0.999	1.24E-90
ABHD17B	1.81E-23	-0.47703	0.620624	0.364	0.528	5.71E-19
PDE4DIP	3.49E-54	-0.476853	0.620734	0.861	0.959	1.10E-49
FKBP10	3.44E-89	-0.476348	0.621047	0.998	1	1.09E-84
ERO1A	3.70E-51	-0.475883	0.621336	0.913	0.97	1.17E-46
C2orf88	2.13E-36	-0.475624	0.621497	0.029	0.156	6.73E-32
NCF2	1.49E-50	-0.475037	0.621862	0.011	0.147	4.69E-46
BRPF1	4.28E-26	-0.474331	0.622301	0.319	0.502	1.35E-21
IVNS1ABP	3.78E-33	-0.473016	0.62312	0.607	0.783	1.19E-28
TESK1	1.40E-28	-0.473007	0.623126	0.449	0.6	4.42E-24
MFHAS1	2.53E-24	-0.472848	0.623225	0.33	0.511	7.98E-20
HLA-L	1.62E-48	-0.472844	0.623227	0.039	0.205	5.12E-44
HOXC10	8.11E-82	-0.472367	0.623525	0.172	0.507	2.56E-77
GLIPR1	9.02E-82	-0.471404	0.624126	0.999	1	2.85E-77
TMEM185B	1.60E-33	-0.470487	0.624698	0.626	0.8	5.04E-29
EPHA3	1.53E-32	-0.4704	0.624752	0.022	0.134	4.81E-28
KLHL28	1.88E-23	-0.469757	0.625154	0.393	0.558	5.92E-19
KHDC4	1.31E-26	-0.469368	0.625397	0.484	0.635	4.12E-22
MTCO1P12	0	-0.469113	0.625557	1	1	0
CTSA	3.13E-71	-0.469057	0.625592	0.972	0.998	9.87E-67
LINC01018	2.92E-51	-0.467971	0.626272	0.025	0.169	9.21E-47
P4HA1	2.82E-66	-0.467242	0.626728	0.985	0.996	8.90E-62
FBXW7	1.10E-32	-0.466851	0.626974	0.522	0.722	3.46E-28
DNMBP	6.59E-27	-0.46645	0.627225	0.618	0.739	2.08E-22
ZPR1	2.02E-48	-0.465323	0.627932	0.788	0.914	6.39E-44
ZNF597	1.13E-27	-0.463648	0.628985	0.11	0.271	3.56E-23
DSG2	5.74E-31	-0.463219	0.629255	0.05	0.173	1.81E-26

HSPD1P1	2.25E-88	-0.462603	0.629643	0.291	0.608	7.11E-84
MFAP3L	6.70E-34	-0.462126	0.629943	0.052	0.197	2.11E-29
PABPC1	6.08E-259	-0.461258	0.63049	1	1	1.92E-254
RAP2A	2.51E-38	-0.461181	0.630538	0.723	0.861	7.91E-34
NOP58	1.96E-54	-0.460939	0.630691	0.916	0.978	6.18E-50
GPR161	3.03E-23	-0.460837	0.630755	0.534	0.675	9.56E-19
IFFO2	8.56E-19	-0.46014	0.631195	0.269	0.406	2.70E-14
DBNDD2	1.22E-33	-0.459302	0.631725	0.543	0.727	3.86E-29
STEAP1	1.40E-36	-0.458513	0.632223	0.349	0.601	4.43E-32
RAB35	1.93E-38	-0.458077	0.632499	0.746	0.856	6.09E-34
TMEM87A	5.27E-58	-0.457948	0.63258	0.944	0.981	1.66E-53
AHI1	5.84E-26	-0.457048	0.63315	0.53	0.693	1.84E-21
PURB	1.79E-54	-0.456826	0.633291	0.951	0.986	5.65E-50
AL021155.4	3.78E-31	-0.456588	0.633441	0.093	0.249	1.19E-26
RBM14	5.63E-36	-0.456537	0.633474	0.681	0.825	1.78E-31
SLC2A5	9.36E-35	-0.456347	0.633594	0.121	0.228	2.95E-30
AGFG1	1.81E-54	-0.456286	0.633632	0.853	0.969	5.72E-50
RAB1A	2.47E-124	-0.456239	0.633663	1	1	7.80E-120
AGPAT5	6.27E-28	-0.456101	0.63375	0.52	0.71	1.98E-23
TRIM61	5.47E-31	-0.455895	0.63388	0.091	0.253	1.73E-26
IL17RA	1.54E-25	-0.455066	0.634406	0.501	0.658	4.86E-21
RUNX3	1.32E-22	-0.454395	0.634832	0.121	0.27	4.16E-18
SLC4A4	4.40E-25	-0.454394	0.634833	0.525	0.7	1.39E-20
GPT2	1.20E-23	-0.454303	0.63489	0.426	0.583	3.79E-19
GSTT2	1.60E-36	-0.453787	0.635218	0.223	0.451	5.04E-32
METRNL	2.22E-53	-0.453085	0.635664	0.757	0.916	7.01E-49
TMF1	2.67E-60	-0.452893	0.635786	0.961	0.998	8.41E-56
SIK1	6.03E-37	-0.452423	0.636085	0.119	0.314	1.90E-32
EPHA4	3.08E-28	-0.452243	0.636199	0.226	0.43	9.71E-24
SMCR8	1.75E-26	-0.451768	0.636502	0.507	0.646	5.51E-22
ZNF622	5.19E-37	-0.45128	0.636812	0.743	0.865	1.64E-32
SPTLC3	6.66E-30	-0.450441	0.637347	0.145	0.285	2.10E-25
TMC7	4.72E-30	-0.450019	0.637616	0.122	0.237	1.49E-25
OTULIN	2.18E-22	-0.449993	0.637633	0.388	0.544	6.88E-18
SMAP2	2.33E-28	-0.449565	0.637905	0.611	0.766	7.35E-24
KCNC4	4.90E-22	-0.449385	0.63802	0.187	0.337	1.55E-17
MTF1	8.82E-29	-0.448677	0.638472	0.567	0.71	2.78E-24
CFB	1.74E-32	-0.448498	0.638587	0.037	0.164	5.48E-28
FZD8	2.86E-32	-0.448279	0.638727	0.23	0.451	9.03E-28
HSPA4	9.04E-82	-0.447961	0.638929	1	1	2.85E-77

ABCA8	1.13E-35	-0.447525	0.639208	0.059	0.21	3.56E-31
TUB	4.04E-23	-0.446947	0.639578	0.102	0.242	1.28E-18
SLC22A23	4.65E-33	-0.446146	0.64009	0.05	0.192	1.47E-28
PDXDC1	3.06E-39	-0.446046	0.640155	0.81	0.898	9.67E-35
C1orf52	1.15E-40	-0.445865	0.64027	0.776	0.893	3.63E-36
PPP1R10	4.60E-20	-0.445644	0.640412	0.673	0.73	1.45E-15
TMEM189	9.17E-36	-0.445474	0.640521	0.781	0.884	2.89E-31
LINC02693	5.31E-24	-0.445442	0.640541	0.389	0.566	1.68E-19
PPAT	4.11E-32	-0.444809	0.640947	0.539	0.752	1.30E-27
MOCS1	5.17E-22	-0.444119	0.641389	0.144	0.297	1.63E-17
CALB2	3.06E-33	-0.443923	0.641515	0.044	0.186	9.66E-29
SVEP1	1.39E-21	-0.443871	0.641548	0.13	0.278	4.38E-17
MMP24OS	6.66E-59	-0.443825	0.641578	0.909	0.972	2.10E-54
MACROH2A2	1.04E-40	-0.443598	0.641723	0.148	0.375	3.28E-36
FRS2	3.52E-30	-0.442819	0.642223	0.635	0.769	1.11E-25
KPNA4	4.38E-65	-0.442209	0.642615	0.986	0.996	1.38E-60
SERTAD1	4.70E-22	-0.44069	0.643592	0.786	0.835	1.48E-17
DSE	1.17E-62	-0.439726	0.644213	0.956	0.992	3.69E-58
COL11A1	8.83E-10	-0.439256	0.644516	0.075	0.151	2.78E-05
PITPNB	4.64E-52	-0.438964	0.644704	0.967	0.989	1.46E-47
FAM102A	1.71E-21	-0.438475	0.645019	0.462	0.619	5.39E-17
ZNF791	6.74E-31	-0.437238	0.645817	0.633	0.755	2.13E-26
MSX2	1.45E-72	-0.437128	0.645889	0.001	0.144	4.57E-68
CSTA	4.31E-114	-0.437071	0.645925	0.003	0.228	1.36E-109
PMM2	1.65E-43	-0.437071	0.645926	0.808	0.909	5.22E-39
PCSK5	2.39E-30	-0.436643	0.646202	0.079	0.225	7.53E-26
IFNGR1	6.06E-31	-0.436562	0.646254	0.628	0.781	1.91E-26
BZW2	1.75E-52	-0.43649	0.646301	0.932	0.987	5.53E-48
GRWD1	2.31E-25	-0.436446	0.646329	0.611	0.717	7.28E-21
CERT1	5.46E-29	-0.436085	0.646563	0.723	0.842	1.72E-24
AC022167.2	2.00E-44	-0.435976	0.646633	0.604	0.733	6.30E-40
FAM168B	1.34E-42	-0.43558	0.646889	0.842	0.917	4.24E-38
KALRN	1.11E-25	-0.435339	0.647045	0.113	0.263	3.49E-21
MEX3C	4.53E-33	-0.435297	0.647072	0.679	0.829	1.43E-28
ANKRD29	2.04E-34	-0.435222	0.647121	0.052	0.2	6.42E-30
KCNQ5	6.92E-63	-0.43502	0.647252	0.108	0.376	2.18E-58
COL3A1	2.32E-42	-0.434201	0.647782	1	1	7.32E-38
MARCHF3	1.56E-20	-0.434029	0.647893	0.256	0.417	4.91E-16
ZDHHC9	5.62E-24	-0.432683	0.648766	0.556	0.716	1.77E-19
AC004980.5	1.48E-62	-0.43218	0.649092	0.048	0.258	4.68E-58

SRPX	1.34E-27	-0.432171	0.649098	0.598	0.769	4.23E-23
AMMECR1	7.30E-28	-0.431913	0.649266	0.713	0.824	2.30E-23
AC004980.1	1.14E-71	-0.43155	0.649502	0.04	0.27	3.60E-67
DIP2B	3.46E-21	-0.431106	0.64979	0.437	0.576	1.09E-16
PPIC	3.51E-55	-0.430314	0.650305	0.924	0.982	1.11E-50
AC005261.1	2.73E-27	-0.428444	0.651522	0.518	0.691	8.61E-23
LINC02436	9.42E-44	-0.428275	0.651632	0.513	0.718	2.97E-39
FO681492.1	1.32E-30	-0.427813	0.651933	0.168	0.325	4.16E-26
UBE2E2	2.01E-25	-0.427548	0.652106	0.407	0.596	6.34E-21
RAP1B	2.72E-105	-0.426806	0.65259	1	1	8.58E-101
TNFAIP8	2.91E-20	-0.426046	0.653086	0.305	0.466	9.19E-16
FAT1	4.76E-22	-0.425298	0.653575	0.889	0.924	1.50E-17
SYT15	7.78E-26	-0.425053	0.653735	0.237	0.387	2.45E-21
MOCS3	6.62E-22	-0.42453	0.654077	0.354	0.514	2.09E-17
AC012213.5	1.24E-52	-0.424258	0.654255	0.615	0.794	3.90E-48
EIF2AK3	4.27E-22	-0.424148	0.654327	0.221	0.393	1.35E-17
MED27	5.92E-28	-0.424119	0.654346	0.705	0.806	1.87E-23
TMEM165	7.65E-63	-0.424013	0.654415	0.99	0.996	2.41E-58
TRIM39	2.56E-23	-0.423638	0.654661	0.194	0.357	8.06E-19
HK1	1.80E-45	-0.423277	0.654897	0.945	0.978	5.69E-41
KDM6A	5.25E-22	-0.423274	0.654899	0.488	0.64	1.66E-17
LRRC8B	1.93E-27	-0.423261	0.654908	0.081	0.206	6.10E-23
NUTM2B-AS	3.48E-37	-0.423215	0.654938	0.629	0.805	1.10E-32
SMG1	1.01E-40	-0.422345	0.655508	0.918	0.951	3.18E-36
SLCO3A1	3.61E-27	-0.422332	0.655516	0.229	0.428	1.14E-22
PALM	7.90E-51	-0.421682	0.655943	0.056	0.222	2.49E-46
ICE1	2.76E-26	-0.421365	0.656151	0.612	0.755	8.71E-22
SNIP1	1.01E-30	-0.42098	0.656403	0.536	0.725	3.18E-26
ANKRD42	9.96E-21	-0.420613	0.656644	0.329	0.496	3.14E-16
VGLL3	4.16E-29	-0.420531	0.656698	0.979	0.984	1.31E-24
ISG20L2	1.64E-30	-0.42045	0.656751	0.66	0.761	5.16E-26
RAB7B	3.99E-21	-0.420399	0.656785	0.172	0.333	1.26E-16
LARP4	9.90E-41	-0.42013	0.656962	0.839	0.929	3.12E-36
SNTA1	2.42E-19	-0.419755	0.657208	0.248	0.387	7.64E-15
LIMA1	1.62E-76	-0.419668	0.657265	0.998	1	5.10E-72
RRBP1	2.56E-78	-0.418887	0.657779	1	1	8.07E-74
NMD3	9.32E-44	-0.418804	0.657833	0.912	0.96	2.94E-39
APCDD1L	1.97E-21	-0.41768	0.658573	0.82	0.789	6.21E-17
THBS3	9.31E-20	-0.417561	0.658651	0.368	0.512	2.94E-15
IDS	9.10E-49	-0.416705	0.659215	0.927	0.971	2.87E-44

PYCR1	8.72E-53	-0.416613	0.659276	0.947	0.989	2.75E-48
PLD3	1.54E-37	-0.416288	0.65949	0.906	0.956	4.86E-33
TRIM16	8.32E-19	-0.415639	0.659919	0.496	0.615	2.63E-14
MED26	4.75E-23	-0.415396	0.660079	0.198	0.369	1.50E-18
SAR1A	5.96E-75	-0.415142	0.660246	0.997	1	1.88E-70
FLVCR1	4.85E-22	-0.415108	0.660269	0.291	0.458	1.53E-17
FARP2	1.82E-21	-0.414987	0.660349	0.562	0.7	5.74E-17
GSTT2B	7.64E-37	-0.414954	0.660371	0.211	0.44	2.41E-32
SLC7A6	2.72E-26	-0.414806	0.660468	0.527	0.689	8.57E-22
INSR	1.01E-23	-0.414737	0.660514	0.205	0.384	3.17E-19
PNPLA2	1.78E-35	-0.414152	0.6609	0.841	0.905	5.61E-31
TSPYL5	5.85E-19	-0.413538	0.661307	0.406	0.548	1.84E-14
ACVR1	2.45E-25	-0.41331	0.661457	0.73	0.832	7.72E-21
ASPHD1	1.50E-37	-0.413161	0.661556	0.103	0.294	4.74E-33
GNL3	1.07E-48	-0.412853	0.66176	0.973	0.995	3.36E-44
ZNF644	1.32E-34	-0.412744	0.661832	0.828	0.909	4.16E-30
WDR36	7.49E-26	-0.412595	0.66193	0.696	0.813	2.36E-21
TIMP1	3.65E-58	-0.412594	0.661931	1	1	1.15E-53
MAPKAPK3	6.08E-24	-0.4119	0.662391	0.447	0.627	1.92E-19
ANXA8L1	3.91E-50	-0.411602	0.662588	0.054	0.248	1.23E-45
ERN1	4.82E-21	-0.410852	0.663085	0.203	0.354	1.52E-16
AC244197.3	2.04E-47	-0.410635	0.663229	0.917	0.96	6.42E-43
ATP6V0D1	7.32E-33	-0.410571	0.663271	0.802	0.872	2.31E-28
LINC00632	8.59E-28	-0.410248	0.663486	0.663	0.794	2.71E-23
PLAA	1.18E-28	-0.409827	0.663765	0.812	0.868	3.71E-24
OLFML1	4.39E-28	-0.40935	0.664082	0.032	0.144	1.38E-23
ZNF385A	9.47E-34	-0.409113	0.664239	0.15	0.341	2.99E-29
TMEM64	8.38E-23	-0.407835	0.665089	0.391	0.573	2.64E-18
SLC1A1	8.13E-28	-0.407694	0.665182	0.361	0.574	2.57E-23
CPEB2	2.49E-19	-0.407629	0.665226	0.51	0.649	7.86E-15
RABGAP1L	7.68E-16	-0.407563	0.665269	0.447	0.506	2.42E-11
AC099552.5	9.30E-35	-0.407278	0.665459	0.026	0.152	2.93E-30
RC3H1	3.25E-23	-0.406708	0.665838	0.592	0.724	1.03E-18
ODAPH	1.62E-20	-0.406303	0.666108	0.039	0.107	5.10E-16
AC099548.2	1.49E-56	-0.406069	0.666264	0.027	0.203	4.70E-52
AC068580.4	3.45E-52	-0.40606	0.66627	0.154	0.398	1.09E-47
TIAM2	1.00E-15	-0.405968	0.666332	0.322	0.447	3.16E-11
SIX4	8.40E-17	-0.405909	0.666371	0.21	0.344	2.65E-12
PDE1A	5.76E-19	-0.405836	0.66642	0.122	0.257	1.82E-14
SCAMP5	5.44E-55	-0.405643	0.666548	0.013	0.163	1.71E-50

TCTA	1.79E-32	-0.405615	0.666567	0.724	0.867	5.65E-28

pct.1 Percent of cells expressing in hAM-Muse cells

pct.2 Percent of cells expressing in hBM-Muse cells

Supplementary Table. 2

Upregulated in the large subpopulation than in the small subpopulation

GeneSymbol	p-value	avg_logFC	FC	pct.1	pct.2	p_val_adj
KRT18	0	3.0779793	21.71448	0.995	0.622	0
IGF2	0	2.9256935	18.64715	0.986	0.327	0
RARRES2	1E-296	2.8296411	16.93938	0.955	0.316	3.29E-292
ALDH1A1	1.9E-161	2.7369317	15.43954	0.845	0.283	6.32E-157
HTR2B	9.7E-264	2.7064242	14.97563	0.928	0.241	3.2E-259
CHRM2	9.5E-293	2.6863678	14.67826	0.953	0.265	3.13E-288
SRGN	0	2.6279734	13.84568	0.989	0.645	0
GULP1	0	2.6057482	13.54135	0.986	0.336	0
XIST	4.6E-301	2.5620692	12.96261	0.954	0.258	1.5E-296
TGM2	0	2.534813	12.61407	1	0.782	0
NR2F2	0	2.5174796	12.39731	1	0.852	0
FOS	1.22987761	2.4279003	11.33506	0.984	0.701	4.04642033187492e-3
NR2F1	1E-225	2.3900868	10.91444	0.935	0.355	3.37E-221
GLRX	0	2.3667574	10.66276	1	0.852	0
IRAG1	7.5E-204	2.2935616	9.910171	0.868	0.193	2.48E-199
ADAMTS5	5.2E-136	2.293552	9.910076	0.908	0.538	1.72E-131
PCDH10	4.9E-127	2.293508	9.90964	0.711	0.158	1.61E-122
EDIL3	1E-162	2.2378317	9.372986	0.828	0.267	3.43E-158
AC132217.2	4.5E-301	2.2211834	9.218233	0.943	0.2	1.49E-296
ACTA2	1.9E-234	2.176089	8.811776	1	0.981	6.24E-230
COL4A1	0	2.1493644	8.579403	1	0.926	0
RHOBTB3	0	2.1156949	8.295348	1	0.923	0
SCN9A	1.5E-190	2.0972283	8.143567	0.857	0.202	4.85E-186
LRRC17	3.3E-134	2.0910081	8.093069	0.769	0.22	1.07E-129
PDE5A	4.8E-215	2.0877355	8.066627	0.892	0.232	1.56E-210
OLR1	7.4E-150	2.0590952	7.838874	0.774	0.172	2.42E-145
RGCC	9.69E-76	2.0523513	7.786187	0.569	0.162	3.188E-71
GABARAPL1	3.2E-221	2.0506944	7.773297	0.967	0.508	1.04E-216
MMP11	3E-37	2.0481765	7.753749	0.392	0.137	9.882E-33
GPRC5A	1.1E-208	2.0269766	7.591101	0.958	0.499	3.51E-204
HOXD11	1.6E-231	2.025756	7.58184	0.854	0.09	5.24E-227
SYTL5	1.1E-117	2.0187535	7.528934	0.635	0.1	3.58E-113
TRHDE	4.6E-259	1.9940501	7.345223	0.961	0.362	1.53E-254
ACTG2	3.05E-28	1.9898156	7.314185	0.316	0.128	1.003E-23
MITF	1.1E-171	1.944524	6.990304	0.847	0.232	3.63E-167
SYNPO2	1.4E-149	1.9035158	6.709442	0.925	0.494	4.45E-145
NUAK1	5.2E-209	1.8983999	6.675205	0.949	0.434	1.72E-204

FOXL2	4.3E-241	1.897255	6.667567	0.962	0.422	1.42E-236
PRICKLE1	5.5E-157	1.8938488	6.644894	0.751	0.128	1.82E-152
ESAM	9.8E-116	1.8673924	6.471399	0.636	0.116	3.22E-111
HOXD10	1.3E-264	1.8662685	6.46413	0.904	0.116	4.35E-260
SOX4	1.4E-101	1.8587511	6.415719	0.87	0.536	4.608E-97
REN	1.26E-32	1.8473256	6.342834	0.26	0.046	4.137E-28
PPME1	5.8E-111	1.8383348	6.286062	0.971	0.835	1.9E-106
MGST1	0	1.834441	6.261633	1	0.624	0
FILIP1L	2.2E-245	1.8291307	6.22847	0.993	0.735	7.14E-241
MEST	6.73E-87	1.8253882	6.205203	0.597	0.158	2.215E-82
FOSB	1.4E-114	1.8172998	6.155216	0.793	0.346	4.45E-110
ERAP2	4.2E-150	1.7920946	6.002011	0.804	0.255	1.39E-145
COL4A2	0	1.7813745	5.938013	1	0.958	0
ARMC9	1.9E-205	1.7808437	5.934862	0.967	0.534	6.28E-201
GBP4	1.5E-119	1.758202	5.801996	0.787	0.316	4.83E-115
LMOD1	5E-119	1.7440359	5.720384	0.735	0.22	1.64E-114
CALD1	0	1.7301148	5.641302	1	1	0
DIO2	1.75E-30	1.6963455	5.453979	0.294	0.086	5.771E-26
OTULINL	4.3E-116	1.6864131	5.400076	0.696	0.19	1.41E-111
TMTC1	8.78E-76	1.6725153	5.325547	0.565	0.132	2.887E-71
HLA-A	0	1.6666232	5.29426	1	1	0
FOXL2NB	3.4E-108	1.6599527	5.259062	0.693	0.19	1.12E-103
TRBC2	3.6E-101	1.6536509	5.226025	0.536	0.044	1.198E-96
AMIGO2	2.4E-152	1.6501495	5.207758	0.949	0.626	7.77E-148
AR	1.6E-130	1.6475305	5.194137	0.721	0.148	5.21E-126
RBP1	1.35E-83	1.6352732	5.130859	0.56	0.109	4.439E-79
HAND2	9.4E-138	1.6009301	4.957641	0.828	0.255	3.08E-133
CDH2	1.2E-307	1.5972449	4.939405	0.998	0.715	3.86E-303
PALLD	0	1.584072	4.874766	1	0.998	0
ARID5B	1.2E-263	1.5816228	4.862841	0.998	0.905	4.07E-259
IGFBP5	2.06E-08	1.5816212	4.862833	0.484	0.473	0.0006764
NCKAP5	3.83E-81	1.5527592	4.724488	0.531	0.093	1.259E-76
LBH	2.4E-134	1.5392997	4.661325	0.987	0.9	7.98E-130
FAM43A	1.93E-80	1.5349978	4.641315	0.648	0.211	6.359E-76
AC132217.1	1.7E-212	1.5179349	4.562793	0.825	0.079	5.56E-208
SORT1	1.6E-114	1.5096129	4.524979	0.81	0.353	5.34E-110
RNF150	2.62E-93	1.4901394	4.437714	0.63	0.142	8.605E-89
IFIT1	5.53E-88	1.4856487	4.41783	0.703	0.246	1.821E-83
LXN	2E-113	1.4749405	4.370776	0.797	0.318	6.62E-109
RBM24	2E-122	1.4694504	4.346845	0.907	0.568	6.6E-118

ITGA1	3.06E-95	1.4641118	4.323701	0.978	0.896	1.007E-90
TNFSF4	3.38E-61	1.4623435	4.316062	0.626	0.258	1.113E-56
MGARP	2.41E-83	1.4550626	4.284752	0.613	0.16	7.924E-79
HNMT	4.4E-104	1.4488784	4.258336	0.827	0.434	1.44E-99
GSDME	3.8E-167	1.435563	4.20201	0.976	0.654	1.26E-162
FBLIM1	1.5E-187	1.4233917	4.151176	0.996	0.914	5.02E-183
PCDH7	6.57E-37	1.4212421	4.142262	0.346	0.093	2.162E-32
A2M	1.17E-37	1.4190309	4.133113	0.273	0.039	3.844E-33
PTN	1.8E-135	1.4068062	4.082895	0.941	0.624	5.83E-131
LINC02844	1.95E-72	1.3997909	4.054352	0.426	0.037	6.423E-68
PLAT	1.1E-104	1.3891174	4.011308	0.976	0.828	3.6E-100
WNT5A	6.6E-148	1.3808005	3.978085	0.996	0.835	2.17E-143
FAM155A	8.38E-92	1.3786746	3.969637	0.69	0.213	2.759E-87
IRS1	3.9E-170	1.3715173	3.941326	0.981	0.682	1.29E-165
THSD4	6.54E-78	1.3705621	3.937564	0.665	0.239	2.152E-73
KLF9	3E-105	1.3624505	3.905753	0.878	0.503	9.97E-101
SLC7A11	2.1E-226	1.3563923	3.882162	0.999	0.824	6.75E-222
NEXN	2.1E-260	1.3407496	3.821908	0.999	0.875	6.96E-256
OGFRL1	2.2E-128	1.3358235	3.803127	0.987	0.817	7.2E-124
HOXA11	4.3E-101	1.3311316	3.785324	0.92	0.666	1.419E-96
AGRN	7.4E-142	1.3298571	3.780503	0.921	0.527	2.44E-137
GBP2	4.83E-88	1.3284626	3.775235	0.79	0.392	1.589E-83
CNN1	3.71E-79	1.3260841	3.766266	0.781	0.397	1.22E-74
THBS1	2.7E-267	1.3229429	3.754454	1	1	8.91E-263
NEO1	1.21E-83	1.3088655	3.701971	0.713	0.306	3.993E-79
SLC16A4	3.25E-60	1.3071512	3.695631	0.66	0.348	1.071E-55
LYPD1	3.58E-56	1.3018249	3.675999	0.45	0.095	1.177E-51
KRT8	6.8E-85	1.296074	3.654919	0.651	0.206	2.238E-80
RARB	1.23E-59	1.2905149	3.634658	0.409	0.06	4.031E-55
MEIS1	2.96E-93	1.2891118	3.629561	0.829	0.436	9.754E-89
IFIT3	5.01E-88	1.2844543	3.612696	0.901	0.596	1.647E-83
HOXA5	8.09E-85	1.2813387	3.601458	0.61	0.142	2.66E-80
TCIM	1.22E-44	1.2796274	3.5953	0.405	0.118	4.002E-40
HOXD9	9.54E-85	1.2792311	3.593876	0.559	0.102	3.139E-80
GDF15	8.37E-30	1.2765769	3.584349	0.418	0.172	2.753E-25
HAND2-AS1	7.16E-77	1.267157	3.550744	0.635	0.274	2.357E-72
ATP10D	5.34E-73	1.2618695	3.532018	0.731	0.362	1.757E-68
ADGRG6	1.09E-86	1.2613823	3.530298	0.799	0.374	3.587E-82
ZNF804A	7.15E-53	1.2562028	3.51206	0.411	0.084	2.352E-48
C1orf198	5.63E-73	1.2540998	3.504682	0.913	0.738	1.852E-68

ABLIM1	6.5E-71	1.2488641	3.48638	0.667	0.26	2.14E-66
NEDD4L	3.43E-69	1.248557	3.48531	0.646	0.262	1.13E-64
LGALS3BP	3.4E-168	1.2477596	3.482532	0.987	0.735	1.1E-163
GJA1	1.1E-144	1.2472473	3.480748	0.997	0.919	3.68E-140
CPED1	4.9E-120	1.23928	3.453126	0.962	0.677	1.61E-115
CITED2	7E-239	1.2386298	3.450882	1	0.991	2.29E-234
GBP1	1.5E-123	1.2375104	3.447021	0.977	0.791	4.93E-119
MYLK	7.7E-176	1.2341014	3.43529	1	0.993	2.54E-171
AC243919.1	0	1.2315893	3.426671	1	0.995	0
AC011295.1	0	1.2310512	3.424828	1	1	0
AASS	2.3E-95	1.2303422	3.422401	0.854	0.469	7.575E-91
AFF3	1.13E-53	1.2193948	3.385138	0.517	0.183	3.709E-49
BST2	1.55E-37	1.2170872	3.377336	0.331	0.074	5.093E-33
PEAR1	3.42E-68	1.2138028	3.366262	0.546	0.137	1.127E-63
VAT1L	4.21E-76	1.2128034	3.362899	0.791	0.399	1.386E-71
JAZF1	5.9E-115	1.2127388	3.362682	0.945	0.689	1.95E-110
FRY	1.57E-44	1.2117622	3.3594	0.46	0.172	5.179E-40
LIMCH1	4.35E-44	1.2085938	3.348772	0.493	0.188	1.433E-39
CARD16	3.66E-77	1.206565	3.341985	0.604	0.167	1.204E-72
CAMK1G	5.6E-63	1.2031311	3.330529	0.4	0.039	1.842E-58
MASP1	1.89E-92	1.2027922	3.3294	0.933	0.668	6.212E-88
HSBP1L1	5.58E-95	1.2022114	3.327467	0.805	0.422	1.835E-90
NDFIP2	4.1E-167	1.1990714	3.317035	0.994	0.847	1.35E-162
CDKN1B	5.18E-81	1.1954833	3.305155	0.871	0.573	1.703E-76
PRR5L	1.03E-78	1.1901139	3.287456	0.802	0.436	3.4E-74
FENDRR	1.42E-54	1.1897259	3.28618	0.379	0.058	4.675E-50
HOXA11-AS	8.72E-64	1.1847579	3.269895	0.612	0.234	2.87E-59
ME1	3.7E-130	1.1829425	3.263964	0.979	0.798	1.22E-125
HLA-B	1.1E-207	1.1778586	3.247413	1	1	3.66E-203
CLIC3	7.42E-37	1.1774938	3.246228	0.441	0.176	2.443E-32
TRHDE-AS1	9.07E-54	1.1754826	3.239706	0.477	0.125	2.985E-49
ST6GALNAC5	6.48E-50	1.1713017	3.226189	0.672	0.394	2.133E-45
TRIB1	1.19E-54	1.1698106	3.221382	0.618	0.29	3.91E-50
PDLIM5	9.7E-190	1.1680285	3.215647	1	0.991	3.18E-185
PLBD1	5.24E-53	1.1620704	3.196545	0.415	0.077	1.723E-48
NCAM2	1.24E-57	1.1610402	3.193253	0.468	0.107	4.094E-53
GSTM1	3.2E-126	1.1587073	3.185812	0.932	0.617	1.05E-121
IFIT2	5.19E-39	1.154154	3.171339	0.409	0.116	1.708E-34
ADAM19	6.7E-101	1.1528888	3.16733	0.94	0.701	2.209E-96
ITPRID2	8.7E-190	1.1504537	3.159626	1	0.963	2.86E-185

PLPP2	2.3E-56	1.1482649	3.152718	0.518	0.158	7.551E-52
MARCKSL1	8.93E-59	1.1469527	3.148584	0.766	0.42	2.94E-54
STRA6	6.94E-62	1.1376245	3.11935	0.565	0.179	2.285E-57
MLLT11	2.44E-70	1.1366881	3.11643	0.843	0.545	8.028E-66
BEX1	1.08E-15	1.1320082	3.10188	0.216	0.102	3.553E-11
ITGA6	1.3E-209	1.1257043	3.082387	1	0.991	4.29E-205
KCNE4	1.33E-32	1.1225265	3.072607	0.435	0.19	4.381E-28
CCDC68	7.74E-61	1.1183803	3.059894	0.427	0.06	2.546E-56
LITAF	9.6E-137	1.1138264	3.045991	0.988	0.78	3.15E-132
UACA	3.1E-226	1.1120982	3.040732	1	0.991	1.03E-221
CSF2RB	3.45E-43	1.1099943	3.034341	0.28	0.021	1.137E-38
KCNJ8	6.4E-48	1.1063602	3.023334	0.363	0.056	2.104E-43
GNG4	5.18E-60	1.1048571	3.018793	0.6	0.223	1.703E-55
TNS3	3.4E-105	1.1010783	3.007407	0.96	0.694	1.11E-100
SYPL2	2.15E-56	1.1001831	3.004716	0.409	0.058	7.081E-52
AC010735.2	2.97E-54	1.0983104	2.999094	0.433	0.086	9.777E-50
L3MBTL3	9.93E-52	1.0959023	2.991881	0.544	0.202	3.268E-47
MICU3	4.88E-56	1.0902176	2.974921	0.664	0.306	1.606E-51
DMD	3.49E-41	1.0865899	2.964149	0.459	0.19	1.147E-36
SAMD12	3.15E-48	1.0819977	2.950568	0.529	0.246	1.036E-43
HOXA10	5.96E-79	1.0802903	2.945534	0.962	0.826	1.961E-74
ISYNA1	5.41E-56	1.079612	2.943537	0.642	0.297	1.781E-51
HSPB6	2.1E-135	1.075144	2.930415	0.993	0.926	6.9E-131
TXNRD1	1.1E-283	1.0727671	2.923458	1	1	3.52E-279
PCDH9	4.62E-15	1.072189	2.921768	0.692	0.652	1.52E-10
NTN4	6.7E-39	1.0702003	2.915963	0.633	0.378	2.203E-34
DSP	3.47E-64	1.0653704	2.901914	0.896	0.601	1.141E-59
NRGN	1.89E-66	1.0621927	2.892707	0.51	0.102	6.221E-62
PCDH18	1.4E-102	1.0604622	2.887705	0.971	0.752	4.754E-98
PABPC4L	2.1E-45	1.0574558	2.879037	0.315	0.032	6.896E-41
SERTAD4	1.38E-38	1.055903	2.87457	0.382	0.097	4.526E-34
SQSTM1	1.4E-198	1.053716	2.86829	1	0.984	4.5E-194
SPECC1	4.36E-76	1.0532372	2.866917	0.928	0.733	1.434E-71
TEAD3	1.05E-79	1.0524598	2.864689	0.865	0.585	3.445E-75
ARHGDIB	5.19E-41	1.0513951	2.861641	0.52	0.193	1.707E-36
DSEL	5.6E-127	1.0504316	2.858885	0.996	0.935	1.83E-122
CSRP2	7.4E-86	1.0501371	2.858043	0.963	0.782	2.436E-81
LPP	6.9E-150	1.0482249	2.852583	1	0.991	2.27E-145
OXTR	1.1E-105	1.0450518	2.843546	0.993	0.907	3.61E-101
JUP	1.07E-31	1.0443616	2.841584	0.322	0.095	3.532E-27

ANO4	6.65E-47	1.0423298	2.835816	0.421	0.107	2.189E-42
PAPSS2	1.9E-225	1.0403658	2.830252	1	0.998	6.33E-221
RGS4	2.98E-42	1.0366433	2.819736	0.841	0.624	9.796E-38
FAT4	1.51E-50	1.0346101	2.814009	0.617	0.276	4.952E-46
DENND2B	4.68E-83	1.0287967	2.797697	0.879	0.566	1.538E-78
RAB27B	9.01E-36	1.0287609	2.797597	0.414	0.125	2.964E-31
SSTR1	3.47E-93	1.0264371	2.791104	0.989	0.865	1.143E-88
PGR	3.22E-45	1.0253423	2.78805	0.349	0.053	1.06E-40
ZP3	1.63E-52	1.0245692	2.785895	0.466	0.123	5.364E-48
KLHL5	4E-92	1.0238852	2.78399	0.948	0.719	1.316E-87
INPP4B	6.03E-50	1.0212762	2.776736	0.731	0.422	1.984E-45
EPS8	1.3E-144	1.0193421	2.771371	0.999	0.97	4.18E-140
KIF26B	1.96E-28	1.0184135	2.768798	0.319	0.107	6.459E-24
LINC02762	1.68E-51	1.0168686	2.764524	0.609	0.281	5.527E-47
XAF1	1.79E-68	1.0094044	2.743966	0.849	0.619	5.887E-64
IFITM1	1.2E-121	1.0078155	2.73961	0.988	0.87	3.8E-117
KCNMB4	6.86E-36	1.0073007	2.7382	0.293	0.051	2.256E-31
SAMD9L	2.84E-49	1.0061351	2.73501	0.718	0.443	9.333E-45
PRDM1	3.28E-50	1.004962	2.731803	0.785	0.515	1.079E-45
ADRA1D	1.88E-45	1.0037246	2.728425	0.382	0.065	6.174E-41
GYPC	1.8E-83	0.9987751	2.714954	0.958	0.782	5.923E-79
ARHGEF17	4.95E-83	0.9977909	2.712283	0.927	0.671	1.63E-78
LACTB	7.07E-88	0.9956141	2.706386	0.968	0.817	2.326E-83
ZFHX4	2.61E-40	0.9948707	2.704375	0.591	0.325	8.585E-36
RAI14	4.4E-220	0.9929982	2.699316	1	0.991	1.45E-215
EFHD1	7.69E-36	0.992855	2.698929	0.276	0.039	2.53E-31
MSI2	4.57E-53	0.9885767	2.687407	0.793	0.529	1.503E-48
DHRS3	5.64E-25	0.9880422	2.685971	0.404	0.186	1.856E-20
SEMA6D	1.82E-26	0.9877561	2.685202	0.314	0.116	5.976E-22
SLC7A2	2.82E-39	0.9869263	2.682975	0.259	0.021	9.291E-35
FZD4	2.93E-37	0.9859285	2.680299	0.415	0.146	9.639E-33
CEBPD	8.3E-57	0.984269	2.675855	0.628	0.276	2.732E-52
GATA2	4.99E-80	0.9826254	2.671461	0.957	0.775	1.641E-75
LRRC1	7.51E-45	0.9806429	2.66617	0.468	0.16	2.47E-40
NECTIN2	7.6E-93	0.9800677	2.664636	0.964	0.791	2.501E-88
TMEM200A	4.09E-60	0.9759173	2.6536	0.933	0.794	1.344E-55
CDH4	7.12E-43	0.9758168	2.653333	0.553	0.23	2.344E-38
FOXF1	1.5E-45	0.9710477	2.64071	0.605	0.288	4.927E-41
TENT5C	4.17E-39	0.9670123	2.630075	0.266	0.023	1.373E-34
AP1M2	3.56E-57	0.9665317	2.628811	0.361	0.028	1.171E-52

TANC1	4.99E-51	0.9654537	2.625979	0.79	0.515	1.643E-46
PLEKHA2	1.2E-109	0.9652583	2.625466	0.99	0.905	4.03E-105
ABCC9	8.32E-40	0.9612093	2.614857	0.528	0.271	2.739E-35
UCHL1	1.9E-113	0.9608178	2.613833	0.993	0.868	6.1E-109
MYH10	1.41E-59	0.9605231	2.613063	0.948	0.803	4.632E-55
MPDZ	8.37E-50	0.9581863	2.606964	0.754	0.492	2.754E-45
RGS2	5.14E-22	0.9573289	2.60473	0.428	0.253	1.69E-17
TLR4	2.67E-40	0.9569393	2.603715	0.508	0.213	8.788E-36
MYH9	7.2E-302	0.9537914	2.595532	1	1	2.36E-297
TEK	9.4E-43	0.9519414	2.590734	0.332	0.049	3.092E-38
FRMPD4	2.64E-36	0.9515604	2.589748	0.363	0.093	8.701E-32
CEMIP2	2.91E-48	0.9502232	2.586287	0.767	0.513	9.568E-44
NCOA7	2.28E-41	0.9493283	2.583973	0.662	0.404	7.51E-37
TLE4	1.45E-52	0.9482064	2.581076	0.838	0.592	4.768E-48
GSTT1	7.54E-53	0.948172	2.580987	0.803	0.559	2.48E-48
TNS2	2.23E-47	0.9477377	2.579867	0.718	0.418	7.334E-43
GSTM2	1.03E-91	0.9458382	2.574971	0.915	0.636	3.38E-87
WWC2	8.94E-88	0.9452426	2.573438	0.994	0.949	2.941E-83
ANXA3	1.28E-29	0.9446482	2.571909	0.423	0.193	4.211E-25
SPTAN1	1.3E-124	0.9422588	2.56577	0.996	0.937	4.41E-120
NPC2	8.6E-142	0.9419643	2.565015	0.998	0.986	2.82E-137
ANGPT1	1.79E-85	0.9323472	2.540465	0.981	0.856	5.879E-81
HYI	4.45E-63	0.930418	2.535569	0.906	0.687	1.465E-58
SLC2A1	5.52E-71	0.9286012	2.530966	0.974	0.821	1.816E-66
AFAP1	5.9E-83	0.9275031	2.528189	0.955	0.749	1.94E-78
HSPB1	0	0.923282	2.517539	1	1	0
GSTM4	1.92E-42	0.9221041	2.514576	0.658	0.383	6.302E-38
SCUBE3	1.31E-64	0.9195407	2.508138	0.935	0.705	4.324E-60
LINC01391	1.59E-45	0.918713	2.506063	0.339	0.049	5.231E-41
HHIP	2.7E-36	0.918113	2.50456	0.292	0.044	8.875E-32
SNHG18	4.2E-48	0.9162614	2.499927	0.738	0.445	1.383E-43
USP53	8.62E-29	0.9158655	2.498937	0.641	0.445	2.835E-24
CUL4B	3.76E-79	0.9128045	2.4913	0.984	0.91	1.238E-74
MKX	1.05E-24	0.9109514	2.486687	0.485	0.253	3.456E-20
TSPAN14	7.59E-74	0.9080026	2.479365	0.95	0.814	2.498E-69
TPM2	4.5E-298	0.903957	2.469355	1	1	1.49E-293
FZD2	1.52E-89	0.90376	2.468869	0.975	0.833	4.994E-85
FRMD4A	7.06E-60	0.8985128	2.455948	0.934	0.775	2.323E-55
MAPK8	1.79E-70	0.894239	2.445474	0.947	0.768	5.882E-66
GCLC	2.45E-35	0.8941653	2.445294	0.694	0.48	8.045E-31

GRIK2	2.67E-35	0.8906638	2.436747	0.642	0.459	8.77E-31
IGFBP7	1.5E-145	0.8898345	2.434727	1	0.998	5.01E-141
NOL4L	3.81E-38	0.8874775	2.428995	0.52	0.248	1.253E-33
WLS	2.09E-58	0.8845655	2.421932	0.926	0.712	6.886E-54
PARD3B	3.56E-32	0.8819847	2.415689	0.429	0.19	1.171E-27
SMAD3	1.11E-45	0.8811908	2.413772	0.803	0.564	3.658E-41
GNAI1	5.88E-75	0.880181	2.411336	0.941	0.759	1.934E-70
FLNB	1.52E-88	0.8787432	2.407872	0.992	0.961	4.995E-84
NINJ1	5.8E-46	0.8786102	2.407551	0.85	0.629	1.908E-41
RHOBTB1	2.46E-29	0.8777694	2.405528	0.574	0.383	8.097E-25
ETNK2	7.09E-43	0.8777641	2.405515	0.322	0.042	2.332E-38
MARCKS	6E-187	0.8729161	2.393882	1	1	1.98E-182
INA	2.02E-31	0.8726336	2.393205	0.289	0.053	6.633E-27
SPOCK1	2.8E-75	0.8725046	2.392897	0.99	0.896	9.201E-71
ZNF462	2.67E-41	0.8717079	2.390991	0.497	0.19	8.783E-37
WFS1	2.16E-47	0.870047	2.387023	0.814	0.631	7.107E-43
KLHL24	3.61E-34	0.8683974	2.383089	0.577	0.378	1.188E-29
F2RL1	7.87E-32	0.8646782	2.374242	0.437	0.172	2.591E-27
CLIP3	5.95E-42	0.8623974	2.368833	0.73	0.506	1.958E-37
SLC38A1	3.72E-59	0.8614874	2.366678	0.932	0.684	1.224E-54
USP9X	2.35E-89	0.8612608	2.366142	0.987	0.891	7.73E-85
CCDC50	3E-166	0.857646	2.357604	1	0.993	9.79E-162
ACO1	1.67E-70	0.8564583	2.354806	0.97	0.847	5.485E-66
CADM1	7.04E-25	0.8564548	2.354798	0.323	0.118	2.317E-20
CCDC71L	2.08E-39	0.8557071	2.353038	0.955	0.907	6.84E-35
ARHGEF28	1.15E-34	0.8554484	2.352429	0.456	0.167	3.798E-30
TMEM106B	5.21E-77	0.8554119	2.352343	0.989	0.907	1.715E-72
PLD1	2.54E-33	0.8545593	2.350338	0.314	0.077	8.343E-29
FOXO1	6.76E-25	0.8510563	2.34212	0.25	0.058	2.224E-20
KDM5B	8.78E-53	0.8505086	2.340837	0.904	0.712	2.889E-48
ZNF516	1.11E-35	0.8495482	2.33859	0.706	0.497	3.646E-31
AJUBA	4.23E-58	0.8495122	2.338506	0.892	0.664	1.393E-53
COL8A1	2.85E-90	0.8461785	2.330723	0.993	0.903	9.382E-86
CENPV	8.29E-35	0.8453303	2.328747	0.544	0.262	2.727E-30
HERC2P2	1.46E-36	0.8423437	2.321802	0.602	0.387	4.8E-32
TP53INP2	1.57E-34	0.8421616	2.32138	0.573	0.29	5.162E-30
PDGFB	3.67E-28	0.8419096	2.320794	0.196	0.019	1.206E-23
LIMS2	2.44E-33	0.8396723	2.315608	0.554	0.29	8.033E-29
TLE1	7.61E-41	0.8388004	2.31359	0.766	0.524	2.503E-36
CCDC81	2.64E-37	0.8379986	2.311736	0.308	0.06	8.693E-33

ST3GAL5	2.03E-31	0.8372651	2.310041	0.629	0.408	6.693E-27
ATRX	1.98E-96	0.8330895	2.300415	0.994	0.923	6.503E-92
PARP14	5.78E-34	0.8330257	2.300268	0.699	0.49	1.903E-29
MT-RNR1	3.3E-128	0.8329569	2.30011	1	1	1.1E-123
KRT19	3.59E-73	0.832309	2.29862	0.894	0.55	1.18E-68
REPIN1	2.21E-48	0.8305775	2.294643	0.86	0.705	7.257E-44
LIMS1	2.7E-138	0.8294123	2.291971	1	0.998	8.98E-134
CASP1	9.06E-24	0.8281649	2.289114	0.436	0.223	2.98E-19
NFAT5	1.37E-60	0.827876	2.288453	0.943	0.807	4.498E-56
SOX5	1.85E-37	0.8265887	2.285509	0.275	0.032	6.081E-33
EPHB2	2.3E-26	0.8264698	2.285237	0.399	0.19	7.552E-22
TP53I3	3.51E-25	0.8256033	2.283258	0.89	0.831	1.153E-20
RRAD	1.21E-20	0.8245796	2.280922	0.241	0.067	3.973E-16
COMMD7	8.98E-78	0.8243056	2.280297	0.977	0.826	2.955E-73
APOL6	5.89E-41	0.822438	2.276042	0.872	0.715	1.936E-36
DRAM1	1.65E-73	0.8218699	2.274749	0.985	0.893	5.413E-69
NEFM	3.81E-11	0.8217313	2.274434	0.24	0.121	1.255E-06
BDKRB2	6.81E-23	0.8216994	2.274362	0.38	0.165	2.24E-18
VCL	6.7E-215	0.8206313	2.271934	1	1	2.21E-210
TAGLN	1E-143	0.8185085	2.267116	1	1	3.34E-139
HES1	2.62E-32	0.8152395	2.259717	0.564	0.297	8.631E-28
CBLB	1.51E-31	0.8139451	2.256794	0.651	0.448	4.954E-27
CPT1C	1.09E-37	0.8127493	2.254097	0.61	0.374	3.592E-33
ATXN1	3.21E-41	0.8125141	2.253567	0.868	0.719	1.056E-36
DYNLT3	5E-72	0.8104698	2.248964	0.998	0.986	1.643E-67
HSPA1B	1.78E-39	0.8104177	2.248847	0.965	0.912	5.856E-35
DDX60L	6.57E-36	0.8099156	2.247718	0.649	0.422	2.162E-31
AKAP9	1.13E-49	0.8088396	2.245301	0.93	0.821	3.715E-45
PTPRD	6.48E-28	0.8064157	2.239865	0.35	0.116	2.131E-23
INPPL1	3.41E-62	0.8060962	2.23915	0.949	0.798	1.121E-57
PPM1K	1.91E-33	0.8060818	2.239117	0.665	0.469	6.3E-29
TGIF2	7.79E-27	0.8044532	2.235474	0.347	0.13	2.564E-22
ZDHHC2	3E-58	0.8029391	2.232092	0.942	0.803	9.88E-54
SBF2-AS1	5.72E-17	0.8002939	2.226195	0.514	0.434	1.883E-12
TMEM25	9.68E-35	0.7995682	2.22458	0.435	0.142	3.185E-30
TOR4A	1.25E-46	0.7991091	2.223559	0.744	0.441	4.112E-42
FOXC1	8.62E-32	0.7986077	2.222445	0.532	0.288	2.836E-27
DDIT4	1.94E-17	0.7974437	2.219859	0.564	0.404	6.386E-13
NFIL3	2.27E-25	0.7955496	2.215658	0.495	0.276	7.455E-21
PDE10A	3.42E-28	0.7950806	2.214619	0.282	0.079	1.127E-23

C16orf87	4.81E-45	0.7919508	2.207699	0.851	0.687	1.582E-40
GOLGB1	1.59E-51	0.7887622	2.200671	0.939	0.833	5.216E-47
GLI2	9.63E-32	0.7878547	2.198675	0.673	0.457	3.167E-27
CDC42BPA	1.36E-56	0.7862542	2.195158	0.956	0.858	4.476E-52
TP53	5.93E-45	0.7862483	2.195146	0.872	0.677	1.952E-40
TRIM22	5.65E-43	0.7847942	2.191956	0.837	0.615	1.86E-38
RHOJ	1.42E-32	0.7845454	2.191411	0.3	0.063	4.658E-28
SHROOM3	1.58E-25	0.7811764	2.18404	0.668	0.513	5.19E-21
FBN2	9.37E-27	0.7766984	2.174282	0.363	0.118	3.083E-22
GPR37	1.8E-26	0.7764898	2.173828	0.333	0.1	5.91E-22
MAGI2-AS3	1.12E-52	0.776163	2.173118	0.953	0.828	3.689E-48
NLK	3.36E-30	0.77558	2.171851	0.544	0.332	1.106E-25
FCGRT	2.24E-42	0.7750498	2.1707	0.817	0.619	7.382E-38
ARRB1	2.91E-24	0.7745541	2.169625	0.394	0.179	9.58E-20
LURAP1L	9.66E-24	0.7728342	2.165896	0.404	0.204	3.177E-19
EPHA4	1.69E-13	0.7714681	2.162939	0.242	0.132	5.565E-09
CDK15	5.69E-28	0.7699067	2.159565	0.66	0.457	1.874E-23
MYL9	1.3E-182	0.7690857	2.157793	1	1	4.27E-178
BTBD3	5.99E-36	0.7673258	2.153998	0.924	0.821	1.971E-31
RABGAP1	3.12E-38	0.7673247	2.153996	0.763	0.603	1.025E-33
G6PD	1.13E-86	0.764614	2.148165	0.997	0.968	3.723E-82
KRTAP1-5	4.64E-13	0.764487	2.147892	0.373	0.234	1.525E-08
MCTP1	2.48E-30	0.7639176	2.14667	0.218	0.021	8.154E-26
MLLT3	3.31E-37	0.7632699	2.14528	0.808	0.633	1.089E-32
ZDHHC17	3.76E-39	0.7628797	2.144443	0.838	0.664	1.237E-34
NRIP1	1.7E-46	0.7627586	2.144183	0.908	0.74	5.585E-42
GCNT4	6.16E-25	0.7617538	2.14203	0.315	0.111	2.026E-20
TMEM52B	6.43E-24	0.7614841	2.141452	0.201	0.035	2.116E-19
B3GALT2	9.24E-24	0.760116	2.138524	0.177	0.021	3.04E-19
TFAP2A	8.54E-20	0.7574704	2.132874	0.356	0.376	2.811E-15
MACF1	1.79E-98	0.7571829	2.132261	0.998	0.972	5.896E-94
NID1	1.21E-79	0.7556139	2.128918	1	0.991	3.987E-75
RAI1	5.73E-30	0.7498117	2.116601	0.623	0.397	1.884E-25
KISS1	8.62E-22	0.7484629	2.113748	0.171	0.021	2.837E-17
REST	4.48E-38	0.748225	2.113246	0.889	0.715	1.473E-33
TACC1	6.6E-105	0.7479963	2.112762	1	0.991	2.18E-100
PRKAR2B	3.32E-25	0.7476707	2.112075	0.596	0.415	1.092E-20
HHIP-AS1	3.32E-29	0.7465161	2.109637	0.211	0.019	1.092E-24
DUXAP8	1.91E-32	0.7429838	2.102199	0.325	0.081	6.276E-28
STK38L	2.03E-22	0.7427706	2.101751	0.475	0.292	6.679E-18

ARHGAP20	1.1E-29	0.7423905	2.100952	0.227	0.026	3.612E-25
PIR	3.53E-22	0.7414167	2.098907	0.428	0.218	1.161E-17
ZMAT3	8.68E-30	0.7388232	2.09347	0.957	0.893	2.857E-25
PNRC1	7.2E-34	0.7383274	2.092433	0.727	0.524	2.368E-29
SYT11	5.43E-31	0.7366097	2.088842	0.745	0.548	1.786E-26
SYT16	3.95E-27	0.7353526	2.086217	0.259	0.06	1.3E-22
SLC5A3	1.4E-27	0.7345966	2.084641	0.734	0.531	4.612E-23
LPCAT2	8.93E-53	0.7331716	2.081672	0.95	0.821	2.938E-48
RAB8B	9.44E-49	0.7330119	2.08134	0.912	0.745	3.105E-44
JUND	2.16E-73	0.7303597	2.075827	0.974	0.907	7.107E-69
GLCCI1	7.57E-20	0.7296959	2.07445	0.359	0.181	2.49E-15
ZFAND5	1.48E-86	0.7290585	2.073128	0.999	0.991	4.881E-82
TJP1	1.59E-53	0.7283175	2.071592	0.959	0.849	5.226E-49
NRXN3	6.56E-20	0.7281068	2.071156	0.226	0.072	2.159E-15
ZFP36	3.76E-28	0.7277734	2.070465	0.82	0.694	1.238E-23
UBE2L6	7.2E-36	0.7277029	2.070319	0.897	0.766	2.369E-31
NF1	2.34E-41	0.7266273	2.068094	0.9	0.701	7.683E-37
LRRC61	9.21E-41	0.7250853	2.064907	0.278	0.023	3.029E-36
LUZP1	3.6E-39	0.7241441	2.062965	0.887	0.733	1.184E-34
ARHGEF35-A	1.7E-26	0.7237266	2.062104	0.463	0.248	5.59E-22
PHTF2	9.05E-63	0.7234338	2.0615	0.987	0.944	2.977E-58
NAPRT	5.04E-27	0.7228035	2.060201	0.54	0.302	1.658E-22
LAMA5	1.46E-29	0.7225836	2.059748	0.379	0.128	4.8E-25
GARRE1	1.22E-30	0.7224113	2.059393	0.76	0.643	4.02E-26
FAM160B1	2.38E-37	0.7210445	2.05658	0.844	0.65	7.82E-33
KHDRBS3	3.71E-29	0.7175265	2.049358	0.716	0.527	1.22E-24
TAP1	3.75E-39	0.7164334	2.047119	0.898	0.71	1.233E-34
RASSF3	2.02E-26	0.7148482	2.043876	0.617	0.397	6.636E-22
HOXA7	3.6E-30	0.7107992	2.035618	0.512	0.239	1.185E-25
BAIAP2L1	1.01E-17	0.7106649	2.035344	0.347	0.172	3.33E-13
TBX2	2E-37	0.7089443	2.031845	0.751	0.487	6.587E-33
MKNK2	3.13E-37	0.7085829	2.031111	0.855	0.689	1.029E-32
SLC6A6	1.06E-35	0.7077916	2.029504	0.765	0.594	3.494E-31
SSC5D	8.68E-30	0.7075547	2.029024	0.589	0.346	2.857E-25
DOCK11	7.92E-23	0.7072319	2.028369	0.598	0.418	2.607E-18
SPHKAP	1.11E-18	0.7063064	2.026492	0.184	0.039	3.665E-14
BDH2	9.74E-31	0.7045349	2.022906	0.786	0.617	3.204E-26
SLC35F5	1.11E-40	0.7036941	2.021205	0.967	0.912	3.643E-36
BCAT1	4.2E-116	0.7024043	2.0186	1	0.961	1.37E-111
IFIT5	7.37E-38	0.7020305	2.017846	0.877	0.738	2.424E-33

AMOT	1.8E-18	0.6993323	2.012409	0.355	0.195	5.921E-14
TMEM159	1.68E-25	0.6965654	2.006848	0.837	0.742	5.529E-21
IGF2R	4.83E-45	0.6964939	2.006705	0.967	0.879	1.59E-40
PRAG1	6.99E-22	0.6952752	2.00426	0.481	0.265	2.299E-17
DDAH2	5.41E-38	0.6942524	2.002212	0.903	0.752	1.781E-33
S1PR3	4.7E-22	0.6936494	2.001005	0.89	0.819	1.545E-17
SYDE2	2.52E-22	0.6927619	1.99923	0.405	0.183	8.278E-18
AGA	3.92E-29	0.6927459	1.999198	0.751	0.585	1.289E-24
SNTB2	1.3E-43	0.6913607	1.99643	0.968	0.889	4.269E-39
SLC1A1	1.68E-19	0.6888052	1.991335	0.386	0.186	5.52E-15
FLT1	1.16E-21	0.6872402	1.988221	0.661	0.497	3.818E-17
ATF3	1.35E-18	0.6854805	1.984725	0.333	0.158	4.438E-14
NAMPT	2.09E-37	0.6835795	1.980956	0.932	0.84	6.866E-33
AKAP13	2.71E-40	0.683452	1.980703	0.947	0.845	8.928E-36
SGIP1	3.75E-27	0.6825893	1.978995	0.722	0.564	1.233E-22
BAZ2B	6.91E-23	0.681898	1.977628	0.775	0.684	2.273E-18
INHBA	4.72E-22	0.6804963	1.974858	0.98	0.921	1.551E-17
LAMB1	8.88E-58	0.6794648	1.972822	0.998	1	2.921E-53
COL5A2	3.6E-172	0.6793442	1.972584	1	1	1.17E-167
NIPSNAP1	2.36E-37	0.6791679	1.972236	0.938	0.814	7.777E-33
PBXIP1	2.12E-17	0.6788921	1.971692	0.52	0.406	6.968E-13
CHD6	7.3E-23	0.6760404	1.966077	0.601	0.385	2.402E-18
TKT	3.4E-133	0.676007	1.966012	1	1	1.11E-128
PDLIM7	1.9E-143	0.6741569	1.962378	1	0.995	6.11E-139
IDH1	8.36E-39	0.6729701	1.96005	0.941	0.856	2.749E-34
MXRA5	1.98E-52	0.6728507	1.959816	0.982	0.903	6.53E-48
WWC3	1.39E-30	0.67276	1.959638	0.864	0.761	4.572E-26
CASK	1.33E-29	0.672651	1.959425	0.854	0.777	4.36E-25
DUXAP10	3.09E-35	0.6719378	1.958028	0.346	0.102	1.016E-30
MAP3K7CL	2.99E-18	0.6691123	1.952503	0.538	0.425	9.825E-14
PLXDC2	1.42E-12	0.6688074	1.951908	0.179	0.067	4.669E-08
LAMA1	3.05E-18	0.6686323	1.951566	0.294	0.125	1.003E-13
NR2F2-AS1	5E-31	0.6679348	1.950206	0.318	0.072	1.644E-26
MBOAT7	1.72E-70	0.6626153	1.939859	0.995	0.965	5.659E-66
EML4	6.81E-33	0.6622141	1.939081	0.893	0.761	2.24E-28
PSMB9	1.2E-22	0.6621754	1.939006	0.757	0.615	3.96E-18
OSR2	3.73E-20	0.6603839	1.935535	0.482	0.278	1.227E-15
KDM5C	8.19E-37	0.6591973	1.93324	0.774	0.596	2.695E-32
COL12A1	4.13E-71	0.6591905	1.933227	1	0.988	1.359E-66
FOXN3	3.52E-28	0.658122	1.931162	0.858	0.735	1.159E-23

DBN1	1.32E-79	0.656858	1.928723	1	0.958	4.336E-75
SUSD3	7.79E-16	0.6564644	1.927964	0.213	0.07	2.563E-11
PLCB1	1.22E-23	0.6560794	1.927222	0.762	0.61	4.024E-19
ACTB	0	0.6555318	1.926167	1	1	0
FGF7	1.04E-39	0.6552765	1.925675	0.973	0.868	3.413E-35
HOXD8	1.4E-27	0.6545866	1.924347	0.668	0.464	4.611E-23
NAT14	1.02E-30	0.6544708	1.924124	0.766	0.592	3.36E-26
EVI5	9.87E-26	0.6538966	1.92302	0.847	0.729	3.247E-21
C2CD3	2.18E-26	0.65305	1.921392	0.613	0.545	7.174E-22
ACTN4	2.3E-209	0.6528021	1.920916	1	1	7.65E-205
PDP2	5.85E-26	0.6520587	1.919488	0.592	0.483	1.926E-21
PGD	4.05E-84	0.6517004	1.918801	0.999	0.988	1.333E-79
FOXP1	1.83E-38	0.6507126	1.916906	0.965	0.912	6.005E-34
SSPN	1.26E-28	0.6502965	1.916109	0.901	0.775	4.152E-24
TRIB2	1.32E-18	0.6494694	1.914525	0.502	0.306	4.358E-14
NMT2	7.77E-36	0.6484391	1.912553	0.956	0.903	2.557E-31
OPN3	1.52E-19	0.6482397	1.912172	0.522	0.378	4.997E-15
SLC44A2	1.83E-52	0.6480015	1.911717	0.973	0.872	6.033E-48
PDGFRB	1.33E-80	0.6477293	1.911196	0.991	0.87	4.38E-76
AFF1	1.21E-20	0.6476861	1.911114	0.584	0.432	3.989E-16
LASP1	7.4E-113	0.6466839	1.909199	1	1	2.44E-108
MOSMO	2.85E-25	0.6464404	1.908734	0.724	0.568	9.383E-21
FLNA	4.7E-178	0.6459252	1.907751	1	1	1.55E-173
NQO1	4.6E-135	0.6452721	1.906506	1	1	1.5E-130
MAML2	4.18E-25	0.6449236	1.905841	0.707	0.531	1.375E-20
PLXNB2	1.72E-48	0.6442639	1.904585	0.963	0.877	5.651E-44
MTMR3	1.51E-19	0.6433468	1.902839	0.551	0.408	4.982E-15
RNF157	2.06E-20	0.642622	1.90146	0.372	0.211	6.793E-16
PVR	1.25E-27	0.6424686	1.901168	0.897	0.814	4.096E-23
MDK	1.62E-25	0.6421952	1.900649	0.868	0.766	5.321E-21
GPR183	8.75E-18	0.6421021	1.900472	0.173	0.035	2.877E-13
LIMS3	6.41E-51	0.6416868	1.899683	0.561	0.181	2.109E-46
IFT81	7.5E-23	0.6412678	1.898887	0.693	0.552	2.468E-18
PELO	4.83E-36	0.640059	1.896593	0.975	0.942	1.588E-31
DDX58	1.7E-16	0.6399039	1.896299	0.406	0.248	5.584E-12
LIMS4	2.76E-50	0.6384869	1.893614	0.554	0.176	9.08E-46
BMP2	9.86E-21	0.6384638	1.89357	0.257	0.072	3.243E-16
EXTL1	1.58E-30	0.6381216	1.892922	0.233	0.026	5.2E-26
MEDAG	1.18E-14	0.6379982	1.892688	0.286	0.135	3.867E-10
RAMP1	5.23E-23	0.6378858	1.892476	0.202	0.032	1.72E-18

LINC00643	5.86E-20	0.6378096	1.892331	0.156	0.019	1.929E-15
ATP6V1A	6.96E-49	0.6372181	1.891212	0.979	0.916	2.29E-44
TP53BP1	9.19E-25	0.6369921	1.890785	0.783	0.629	3.025E-20
CHD3	4.05E-40	0.6368693	1.890553	0.909	0.747	1.331E-35
SERPINB9	5.86E-19	0.6366905	1.890215	0.335	0.169	1.93E-14
HDAC9	1.24E-16	0.6364859	1.889828	0.284	0.123	4.089E-12
DENND3	5.91E-17	0.6364735	1.889805	0.393	0.239	1.946E-12
NR2F1-AS1	3.24E-22	0.6360111	1.888931	0.708	0.615	1.066E-17
PLEKHG2	2.59E-24	0.6358501	1.888627	0.588	0.385	8.535E-20
PRNP	1.88E-71	0.6353537	1.88769	1	1	6.198E-67
RASGRF2	1.95E-26	0.6339568	1.885055	0.207	0.023	6.4E-22
RPS27L	2.2E-110	0.6339307	1.885005	1	1	7.28E-106
TGFB1I1	7.93E-71	0.6338762	1.884903	0.998	0.988	2.608E-66
GALNT7	1.58E-24	0.6335706	1.884327	0.764	0.622	5.211E-20
CRIM1	8.4E-123	0.633517	1.884226	1	1	2.76E-118
AGPAT3	1.21E-34	0.6333396	1.883892	0.931	0.814	3.978E-30
SINHCAF	2.57E-22	0.6330982	1.883437	0.661	0.478	8.446E-18
SLC15A3	1.74E-17	0.6325261	1.88236	0.332	0.158	5.74E-13
NOX4	3.04E-19	0.6321026	1.881563	0.243	0.079	1.002E-14
NFE2L3	4.8E-21	0.6317071	1.880819	0.185	0.037	1.579E-16
SYNPO2L	1.09E-21	0.6307574	1.879033	0.29	0.114	3.57E-17
RNF207	4.71E-18	0.6293037	1.876304	0.261	0.114	1.551E-13
TNFAIP2	3.24E-21	0.6291507	1.876017	0.658	0.483	1.066E-16
OBSL1	1.85E-23	0.6290616	1.87585	0.731	0.568	6.079E-19
ZNF703	1.78E-34	0.6288515	1.875455	0.862	0.71	5.857E-30
H2AC6	7.15E-14	0.6288245	1.875405	0.292	0.128	2.352E-09
COL13A1	2.35E-27	0.628782	1.875325	0.712	0.575	7.743E-23
RIPOR2	3.76E-16	0.6277602	1.87341	0.301	0.146	1.238E-11
SLC14A1	9.77E-07	0.6274174	1.872768	0.165	0.111	0.0321518
ABHD4	1.36E-16	0.6257995	1.86974	0.584	0.432	4.461E-12
PLCXD3	1.18E-10	0.6249877	1.868223	0.12	0.042	3.878E-06
ARL5B	1.41E-22	0.62488	1.868022	0.668	0.513	4.631E-18
RBPMS	9.38E-40	0.6243232	1.866982	0.965	0.884	3.086E-35
PKIG	1.58E-45	0.6228771	1.864284	0.975	0.891	5.183E-41
STON1	7.92E-21	0.6209775	1.860746	0.485	0.274	2.605E-16
SHC4	6.07E-15	0.620206	1.859311	0.252	0.111	1.997E-10
NUDT16L1	6.29E-25	0.619872	1.85869	0.796	0.668	2.068E-20
DZIP3	3.09E-16	0.6195515	1.858095	0.553	0.387	1.017E-11
CA12	7.64E-15	0.6193768	1.85777	0.83	0.78	2.515E-10
NABP1	5.34E-25	0.6185564	1.856247	0.942	0.896	1.757E-20

EVA1C	2.08E-16	0.6179652	1.855149	0.345	0.19	6.846E-12
SPART	4.22E-41	0.6173236	1.853959	0.965	0.863	1.387E-36
SERTAD4-AS	9.93E-30	0.6172756	1.85387	0.403	0.148	3.269E-25
PGPEP1	3.81E-22	0.6172325	1.853791	0.544	0.394	1.252E-17
INKA2	1.74E-18	0.6166186	1.852653	0.31	0.142	5.717E-14
ITGA2	3.46E-46	0.6163194	1.852099	0.983	0.907	1.138E-41
ASAP3	1.03E-17	0.6161227	1.851734	0.397	0.2	3.392E-13
NLGN1	5.28E-19	0.615422	1.850437	0.498	0.292	1.736E-14
CILK1	5.16E-23	0.6152374	1.850096	0.704	0.571	1.698E-18
ZBTB38	1.84E-72	0.6141554	1.848095	0.999	0.977	6.048E-68
TEAD2	5.36E-23	0.613603	1.847074	0.6	0.476	1.763E-18
SMCHD1	1.74E-25	0.613108	1.84616	0.894	0.768	5.722E-21
CTTNBP2NL	2.1E-28	0.6127877	1.845569	0.872	0.768	6.901E-24
APOL2	1.58E-20	0.6123701	1.844799	0.521	0.348	5.195E-16
CTNND1	1.35E-44	0.6087996	1.838224	0.979	0.914	4.443E-40
DIPK2A	7.5E-15	0.6087659	1.838162	0.546	0.422	2.466E-10
ZNF281	3.93E-34	0.608369	1.837432	0.971	0.91	1.293E-29
PSMB8	1.66E-30	0.6078739	1.836523	0.915	0.81	5.459E-26
RTKN2	1.11E-13	0.6078442	1.836468	0.322	0.223	3.646E-09
ICAM1	5.1E-12	0.6077748	1.836341	0.149	0.044	1.679E-07
LPAR6	1.53E-17	0.6076577	1.836126	0.313	0.135	5.049E-13
FERMT2	2.49E-75	0.6073168	1.8355	1	0.995	8.206E-71
SLC30A7	9.15E-30	0.6062527	1.833548	0.918	0.826	3.01E-25
PBX1	2.34E-31	0.6053558	1.831904	0.904	0.796	7.702E-27
KLF5	7.48E-12	0.6047391	1.830775	0.472	0.397	2.462E-07
LRP5	4.06E-22	0.6047153	1.830731	0.428	0.234	1.335E-17
KANK2	6.43E-83	0.6047033	1.830709	1	0.991	2.116E-78
CBSL	3.45E-29	0.6045449	1.830419	0.774	0.578	1.135E-24
DCLK2	2.15E-18	0.6029623	1.827524	0.431	0.225	7.077E-14
MYOCD	2.75E-17	0.6029071	1.827424	0.136	0.016	9.048E-13
STAMBPL1	1.2E-21	0.6025809	1.826828	0.782	0.677	3.945E-17
TGFB2	2.2E-12	0.6023974	1.826492	0.277	0.165	7.224E-08
PTGER2	1.67E-11	0.6020918	1.825934	0.3	0.188	5.481E-07
ECHDC2	4.02E-18	0.6015934	1.825024	0.522	0.392	1.322E-13
SMARCA1	2.47E-45	0.6011486	1.824213	0.989	0.94	8.134E-41
TTC8	2.83E-21	0.6006019	1.823216	0.7	0.568	9.318E-17
ISG15	6.19E-32	0.5993083	1.820859	0.869	0.791	2.038E-27
ZBTB20	9.08E-20	0.5990369	1.820365	0.849	0.77	2.987E-15
ZSCAN31	3.29E-12	0.5984172	1.819237	0.134	0.037	1.083E-07
CNN2	3.5E-123	0.5979965	1.818472	1	1	1.14E-118

SYNGR1	1.66E-21	0.59731	1.817224	0.698	0.601	5.475E-17
ZFPM2	3.62E-23	0.5966966	1.81611	0.183	0.026	1.191E-18
NAMPTP1	2.54E-31	0.5964809	1.815718	0.724	0.575	8.369E-27
PLS3	4E-116	0.5964511	1.815664	1	1	1.32E-111
XRN1	7.46E-23	0.5952915	1.81356	0.785	0.65	2.456E-18
ITPR2	1.07E-16	0.5938926	1.811024	0.595	0.492	3.523E-12
ADAMTS1	2.39E-22	0.5935642	1.81043	0.96	0.877	7.87E-18
FAH	2.96E-23	0.5932679	1.809893	0.88	0.789	9.738E-19
BPGM	2.51E-18	0.5930209	1.809446	0.785	0.68	8.253E-14
TCEAL4	4.88E-38	0.5925464	1.808588	0.984	0.97	1.607E-33
HIBADH	2.3E-28	0.5923965	1.808317	0.895	0.768	7.57E-24
BIRC6	3.3E-29	0.5919928	1.807587	0.909	0.789	1.084E-24
CRYZ	6.58E-23	0.5916082	1.806892	0.788	0.696	2.166E-18
IRS2	2.19E-21	0.5914352	1.806579	0.824	0.708	7.216E-17
TCF7L1	2.59E-21	0.5912486	1.806242	0.569	0.378	8.512E-17
LRCH2	6.03E-13	0.5910336	1.805854	0.429	0.285	1.985E-08
F2R	4.67E-51	0.5909545	1.805711	0.993	0.921	1.537E-46
PANX1	2.22E-15	0.5905293	1.804943	0.557	0.406	7.306E-11
DDR1	2.24E-16	0.5895102	1.803105	0.425	0.234	7.383E-12
DDX3X	8.21E-92	0.5886946	1.801635	1	0.998	2.701E-87
PTMS	5.9E-156	0.5885846	1.801437	1	1	1.95E-151
GNG11	4.52E-86	0.5885501	1.801375	1	0.993	1.488E-81
SAV1	8.3E-30	0.5883808	1.80107	0.925	0.865	2.729E-25
MEX3A	2.41E-19	0.5883573	1.801028	0.219	0.053	7.942E-15
CAMK2N1	1.34E-30	0.5881012	1.800566	0.817	0.603	4.397E-26
CAVIN2	3.06E-13	0.5880745	1.800518	0.467	0.304	1.007E-08
SF3B1	2.69E-97	0.5879686	1.800328	0.999	0.993	8.844E-93
DHRS7	1.52E-36	0.5878143	1.80005	0.982	0.919	4.991E-32
ATXN7L3B	2.38E-26	0.5874391	1.799375	0.89	0.807	7.837E-22
CBS	9.79E-29	0.5874315	1.799361	0.789	0.626	3.221E-24
RICTOR	1.17E-20	0.5868969	1.798399	0.675	0.541	3.839E-16
CTSC	2.9E-143	0.5865324	1.797744	1	0.988	9.38E-139
TUBA4A	2.8E-17	0.5860916	1.796951	0.534	0.411	9.228E-13
TRAM1	1.2E-104	0.586083	1.796936	1	1	3.8E-100
VPS13B	4.17E-16	0.5859565	1.796709	0.491	0.341	1.373E-11
SKIL	1.47E-29	0.5848721	1.794761	0.959	0.891	4.839E-25
PPARG	8.77E-17	0.5843821	1.793882	0.191	0.049	2.887E-12
BHMT2	1.76E-17	0.5838171	1.792869	0.57	0.459	5.79E-13
ZMYM2	5.64E-17	0.5837622	1.792771	0.664	0.55	1.857E-12
LAMC1	3.46E-65	0.5836222	1.79252	1	0.998	1.138E-60

ZFPM2-AS1	1.29E-29	0.5835938	1.792469	0.236	0.032	4.259E-25
PALMD	9.39E-13	0.5831043	1.791591	0.158	0.049	3.088E-08
HBD	8.52E-18	0.582569	1.790633	0.146	0.021	2.803E-13
ADAM9	5.7E-101	0.5803637	1.786688	1	0.998	1.889E-96
TRAF5	6.97E-16	0.5802144	1.786421	0.589	0.427	2.292E-11
ANKRD35	1.29E-18	0.5801266	1.786265	0.402	0.211	4.242E-14
SPATS2	2.22E-28	0.5794935	1.785134	0.894	0.761	7.301E-24
TBCK	1.04E-17	0.5793486	1.784875	0.586	0.425	3.429E-13
NFKBIZ	1.15E-24	0.5788101	1.783914	0.914	0.8	3.78E-20
WNK4	7.04E-16	0.5787443	1.783797	0.454	0.276	2.317E-11
ZFX	2.95E-20	0.5785573	1.783464	0.729	0.626	9.706E-16
MYD88	4.07E-21	0.5784397	1.783254	0.658	0.575	1.337E-16
PAWR	1.99E-36	0.5776105	1.781776	0.976	0.893	6.551E-32
RRM2B	1.6E-29	0.5771111	1.780886	0.93	0.826	5.279E-25
NPTX1	1.44E-07	0.5770388	1.780758	0.102	0.028	0.0047504
TET2	1.04E-14	0.5765502	1.779888	0.446	0.297	3.41E-10
PYROXD2	1.3E-15	0.5763008	1.779444	0.266	0.121	4.261E-11
LTA4H	5.95E-21	0.5762244	1.779308	0.792	0.694	1.957E-16
ASCC3	1.79E-35	0.5754132	1.777865	0.979	0.923	5.877E-31
SULF1	2.65E-56	0.5753368	1.777729	1	0.988	8.728E-52
TP53TG1	6.79E-21	0.5752465	1.777569	0.593	0.459	2.234E-16
NEDD9	2.21E-11	0.5747153	1.776625	0.508	0.392	7.263E-07
CARD6	3.65E-19	0.5733396	1.774182	0.282	0.104	1.201E-14
DYSF	3.32E-19	0.5732401	1.774006	0.168	0.026	1.092E-14
NBPF3	2.18E-18	0.5729204	1.773439	0.454	0.302	7.167E-14
ENAH	1.39E-67	0.5727798	1.773189	1	0.993	4.565E-63
DUSP6	1.36E-15	0.5720991	1.771983	0.798	0.745	4.461E-11
BCAM	5.94E-24	0.5718588	1.771557	0.393	0.176	1.955E-19
ANKRD34A	1.84E-36	0.5709618	1.769969	0.768	0.619	6.041E-32
FADS2	1.29E-20	0.5708569	1.769783	0.878	0.782	4.252E-16
DLC1	5.17E-56	0.5703574	1.768899	0.998	0.979	1.702E-51
ITSN1	2E-25	0.570256	1.76872	0.905	0.824	6.567E-21
SPP1	5.02E-10	0.5699554	1.768188	0.35	0.225	1.651E-05
RHBDD2	7.57E-22	0.5684343	1.765501	0.829	0.729	2.491E-17
ZNF771	3.72E-18	0.567991	1.764718	0.538	0.376	1.224E-13
SEMA5A	1.25E-18	0.5672194	1.763357	0.931	0.847	4.115E-14
WNT2	1.82E-07	0.5667414	1.762514	0.17	0.086	0.0060018
IRF1	5.51E-20	0.5661541	1.761479	0.791	0.742	1.813E-15
HMCES	7.67E-19	0.565005	1.759457	0.781	0.631	2.525E-14
CPA4	3.54E-14	0.564536	1.758632	0.167	0.065	1.166E-09

CU633967.1	3.61E-33	0.5639646	1.757627	0.648	0.434	1.188E-28
MYO6	8.19E-16	0.563185	1.756257	0.83	0.745	2.693E-11
NEGR1	1.14E-29	0.5629692	1.755878	0.951	0.852	3.763E-25
CDH13	2.78E-30	0.5627329	1.755463	0.973	0.896	9.137E-26
PLAG1	1.24E-13	0.5626352	1.755292	0.339	0.202	4.089E-09
VSIR	5.2E-15	0.5625032	1.75506	0.452	0.306	1.71E-10
ARL4C	4.54E-17	0.5624839	1.755026	0.679	0.541	1.493E-12
LEPR	1.39E-16	0.5624332	1.754937	0.58	0.432	4.584E-12
GCC2	5.73E-29	0.5623636	1.754815	0.913	0.768	1.885E-24
AC005261.1	5.53E-14	0.5622306	1.754582	0.534	0.397	1.818E-09
CHD2	2.13E-23	0.5616226	1.753515	0.894	0.789	7.021E-19
ARHGEF12	3.98E-42	0.5600347	1.750733	0.989	0.961	1.308E-37
CCDC28B	8.54E-19	0.5599775	1.750633	0.611	0.429	2.81E-14
C1orf54	1.95E-24	0.559832	1.750378	0.316	0.114	6.4E-20
LACTB2	8.35E-21	0.5597377	1.750213	0.851	0.752	2.746E-16
ETNK1	1.32E-22	0.5594671	1.74974	0.816	0.71	4.337E-18
CEP70	4.15E-14	0.5587041	1.748405	0.508	0.397	1.366E-09
KIF3A	3.34E-15	0.5587005	1.748399	0.631	0.55	1.101E-10
TOB1	4.52E-23	0.5584612	1.747981	0.915	0.84	1.486E-18
ARMC4	5.47E-16	0.5578631	1.746935	0.186	0.053	1.799E-11
CREBRF	1.05E-15	0.556952	1.745345	0.575	0.497	3.441E-11
CDKN1A	9.73E-07	0.5569219	1.745292	0.969	0.977	0.0320082
YEATS2	4.55E-17	0.5560071	1.743696	0.646	0.536	1.497E-12
C12orf57	5.97E-52	0.555819	1.743368	0.973	0.956	1.963E-47
DDIT3	7.21E-16	0.554717	1.741448	0.605	0.531	2.373E-11
PTGER4	1.27E-11	0.5546986	1.741416	0.223	0.097	4.191E-07
TSHZ1	7.3E-18	0.5546251	1.741288	0.77	0.666	2.403E-13
PSME4	1.11E-31	0.5533779	1.739118	0.966	0.884	3.659E-27
RANBP17	5.82E-14	0.5533709	1.739105	0.236	0.111	1.916E-09
ABI2	3.11E-30	0.5533274	1.73903	0.955	0.9	1.024E-25
UBR1	1.3E-20	0.5532858	1.738957	0.699	0.587	4.289E-16
KCNH1	1.76E-18	0.5532138	1.738832	0.163	0.03	5.783E-14
PIANP	3.11E-20	0.5530623	1.738569	0.206	0.049	1.022E-15
FYTTD1	4.04E-49	0.5530465	1.738541	0.995	0.972	1.33E-44
SNAI2	3.1E-33	0.5529042	1.738294	0.976	0.926	1.019E-28
AC007920.2	1.54E-14	0.5526311	1.737819	0.287	0.125	5.06E-10
MKLN1	1.33E-25	0.5518083	1.73639	0.86	0.775	4.389E-21
IFITM2	1.98E-93	0.5515537	1.735948	1	1	6.501E-89
PRKAR1B	8.29E-21	0.5513956	1.735674	0.346	0.139	2.728E-16
BAZ2A	2.82E-22	0.5507443	1.734544	0.793	0.71	9.293E-18

RUNX2	2.24E-17	0.5502426	1.733674	0.633	0.515	7.368E-13
HDGFL3	1.26E-36	0.5499355	1.733141	0.961	0.863	4.131E-32
MB21D2	2.38E-16	0.5494501	1.7323	0.603	0.459	7.834E-12
METTL7A	7.51E-13	0.549274	1.731995	0.497	0.362	2.472E-08
NMNAT2	1.41E-14	0.5489685	1.731466	0.253	0.114	4.639E-10
TOP2B	4.85E-32	0.5485	1.730655	0.965	0.893	1.595E-27
RSF1	4.7E-33	0.5480789	1.729926	0.959	0.835	1.547E-28
RBMS2	5.03E-24	0.5477734	1.729398	0.847	0.717	1.656E-19
NES	1.82E-28	0.5471998	1.728406	0.967	0.889	5.989E-24
PLXNB1	3.21E-13	0.5471401	1.728303	0.267	0.125	1.056E-08
CCDC122	1.2E-16	0.5464502	1.727111	0.412	0.297	3.933E-12
XRRA1	2.96E-14	0.5460613	1.72644	0.509	0.353	9.732E-10
TSPAN9	1.59E-20	0.5460153	1.72636	0.645	0.543	5.246E-16
ZMYM3	1.88E-14	0.5459268	1.726207	0.562	0.455	6.183E-10
ZYX	7.73E-93	0.5458955	1.726154	1	1	2.544E-88
ZNF618	1.44E-18	0.5456037	1.72565	0.707	0.601	4.722E-14
TOX	2.81E-12	0.5455925	1.72563	0.464	0.313	9.232E-08
COTL1	3.3E-162	0.5455438	1.725546	1	1	1.08E-157
FAM171B	1.02E-13	0.5449435	1.724511	0.557	0.497	3.357E-09
PCGF2	1.18E-23	0.5448692	1.724383	0.791	0.731	3.887E-19
RARA	3.52E-16	0.5444307	1.723627	0.468	0.29	1.159E-11
KLHL42	2.13E-20	0.5433618	1.721785	0.845	0.752	7.012E-16
SLITRK4	1.77E-15	0.5432108	1.721526	0.129	0.016	5.813E-11
CD200	1.44E-14	0.5431768	1.721467	0.114	0.016	4.747E-10
ITGA4	6.36E-40	0.543049	1.721247	0.997	0.968	2.094E-35
PARP10	1.05E-19	0.542567	1.720418	0.37	0.176	3.465E-15
HDHD2	5.29E-19	0.5414637	1.71852	0.737	0.636	1.739E-14
MBNL1	3.7E-99	0.5414065	1.718422	1	1	1.215E-94
TNIP1	1.36E-23	0.5410423	1.717796	0.863	0.805	4.46E-19
TSPAN31	3.45E-19	0.5404934	1.716854	0.731	0.582	1.136E-14
STIM2	1.2E-16	0.5398003	1.715664	0.659	0.58	3.961E-12
PRDX2	4.57E-67	0.5397355	1.715553	1	0.986	1.505E-62
MT-ND5	1.1E-191	0.5387657	1.71389	1	1	3.54E-187
GATA2-AS1	2.99E-21	0.5387445	1.713854	0.375	0.16	9.844E-17
FABP4	2.92E-18	0.5386887	1.713758	0.128	0.009	9.619E-14
MDM2	1.81E-21	0.5383638	1.713201	0.911	0.865	5.946E-17
FTL	8.4E-130	0.5382992	1.713091	1	1	2.77E-125
GOPC	2.07E-32	0.5375002	1.711723	0.959	0.91	6.802E-28
PFN2	1.15E-54	0.5367085	1.710368	0.997	0.993	3.777E-50
SCD	1.86E-14	0.5366683	1.710299	0.965	0.937	6.126E-10

AEBP2	1.91E-19	0.5361738	1.709454	0.733	0.65	6.285E-15
MOV10	1.92E-17	0.5360357	1.709218	0.668	0.536	6.317E-13
NCOR1	2.1E-40	0.5355643	1.708412	0.987	0.949	6.924E-36
RUNX1	7.02E-17	0.5348465	1.707186	0.883	0.838	2.308E-12
JARID2	2.66E-12	0.5347972	1.707102	0.448	0.332	8.736E-08
AC089983.1	7.16E-26	0.534638	1.70683	0.209	0.028	2.355E-21
MTND5P11	7.35E-59	0.5344106	1.706442	0.9	0.796	2.417E-54
CTNNB1	4.71E-73	0.5340131	1.705764	0.999	0.993	1.549E-68
DZIP1	4.31E-26	0.5339664	1.705684	0.928	0.863	1.418E-21
ANTXR2	2.93E-31	0.5335132	1.704911	0.977	0.916	9.641E-27
OFD1	8.76E-16	0.5333654	1.70466	0.556	0.429	2.883E-11
TOP1	1.54E-42	0.533356	1.704644	0.983	0.898	5.074E-38
ALDH1A2	2.34E-20	0.5330445	1.704113	0.172	0.026	7.705E-16
ABLIM3	2.64E-18	0.533018	1.704067	0.793	0.71	8.677E-14
ZRSR2	2.57E-15	0.5329803	1.704003	0.398	0.23	8.452E-11
RAPGEF2	1.96E-15	0.5319617	1.702268	0.631	0.545	6.434E-11
DDX60	6.97E-13	0.5316579	1.701751	0.353	0.193	2.295E-08
YTHDC2	9.24E-15	0.531265	1.701083	0.547	0.434	3.04E-10
LPIN2	2.75E-15	0.5310542	1.700724	0.608	0.48	9.045E-11
SEMA3D	2.5E-11	0.5303402	1.69951	0.375	0.251	8.233E-07
ACTN1	3E-155	0.5295355	1.698143	1	1	9.88E-151
HOXD3	1.98E-20	0.5295355	1.698143	0.209	0.044	6.508E-16
FAM214A	2.37E-12	0.5292064	1.697585	0.41	0.262	7.802E-08
RAB43	1.99E-19	0.5289358	1.697125	0.478	0.271	6.542E-15
AKAP8L	4.91E-17	0.5283034	1.696052	0.688	0.566	1.614E-12
ZSWIM6	4.91E-16	0.528217	1.695906	0.636	0.503	1.616E-11
TET3	6.61E-10	0.528142	1.695779	0.276	0.183	2.174E-05
ARFGEF2	3.71E-18	0.5278424	1.695271	0.785	0.691	1.221E-13
KCTD16	5.01E-16	0.5274284	1.694569	0.126	0.014	1.649E-11
TCF7L2	7.13E-18	0.5271946	1.694173	0.739	0.585	2.344E-13
HECW2	1.62E-12	0.5266028	1.69317	0.397	0.288	5.327E-08
TNIK	5.56E-10	0.5264211	1.692863	0.239	0.137	1.828E-05
SNCAIP	4.85E-11	0.5258829	1.691952	0.314	0.186	1.595E-06
HEXIM1	2.07E-23	0.5245834	1.689755	0.927	0.87	6.798E-19
HIVEP1	3.89E-12	0.5238447	1.688507	0.365	0.237	1.281E-07
IGFBP4	9.22E-81	0.5234494	1.68784	1	1	3.032E-76
MAP1A	5.56E-30	0.5230574	1.687178	0.985	0.956	1.828E-25
VPS13C	2.48E-15	0.5230358	1.687142	0.64	0.571	8.161E-11
ZKSCAN1	1.76E-24	0.5229255	1.686956	0.959	0.926	5.775E-20
PYCARD	6.72E-18	0.5225314	1.686291	0.78	0.68	2.21E-13

APPBP2	3.2E-22	0.5224776	1.6862	0.879	0.798	1.053E-17
LINC01139	1.78E-13	0.5222336	1.685789	0.201	0.081	5.861E-09
FYCO1	2.11E-14	0.5218481	1.685139	0.446	0.288	6.956E-10
DUBR	9.16E-11	0.5218049	1.685066	0.479	0.362	3.014E-06
NAP1L5	4.48E-14	0.521612	1.684741	0.452	0.323	1.474E-09
TAX1BP1	3.27E-69	0.5214238	1.684424	1	1	1.076E-64
RNF217	3.07E-13	0.5213903	1.684368	0.635	0.548	1.009E-08
PRKCI	5.2E-29	0.5213773	1.684346	0.94	0.886	1.711E-24
TTYH3	3.41E-31	0.5212915	1.684201	0.936	0.854	1.123E-26
SMARCA5	4.81E-47	0.5207304	1.683257	0.995	0.961	1.582E-42
NUPR1	5.72E-18	0.5195388	1.681252	0.893	0.865	1.881E-13
DEPP1	1.81E-09	0.5194064	1.68103	0.166	0.067	5.942E-05
PIK3C2A	4.34E-21	0.5185633	1.679613	0.85	0.733	1.429E-16
USO1	1.03E-50	0.5185067	1.679518	1	0.979	3.386E-46
APOBEC3F	4.36E-18	0.5183576	1.679267	0.275	0.111	1.435E-13
POMK	3.04E-17	0.5171632	1.677263	0.693	0.606	9.998E-13
SLC8A1	1.99E-18	0.516177	1.67561	0.884	0.8	6.548E-14
CCNG1	6.53E-34	0.5157239	1.674851	0.987	0.963	2.148E-29
APBB1IP	3.86E-20	0.5156338	1.6747	0.342	0.128	1.27E-15
DUSP10	2.01E-17	0.5152027	1.673978	0.801	0.657	6.603E-13
LINC01085	1.74E-13	0.5151802	1.67394	0.355	0.197	5.712E-09
RBMS3	3.82E-17	0.513716	1.671491	0.863	0.78	1.257E-12
H2BC8	1.06E-15	0.5135926	1.671285	0.151	0.035	3.481E-11
TNFRSF14	1.98E-11	0.5126139	1.66965	0.363	0.251	6.505E-07
PSTPIP2	2.26E-11	0.512553	1.669548	0.411	0.271	7.439E-07
RANBP2	1.57E-28	0.5120704	1.668743	0.952	0.865	5.182E-24
NAV3	1.06E-15	0.5120249	1.668667	0.844	0.784	3.474E-11
IP6K2	2.88E-22	0.5117474	1.668204	0.873	0.787	9.492E-18
BBX	9.93E-28	0.5115495	1.667873	0.987	0.968	3.269E-23
USP11	1.22E-24	0.5103103	1.665808	0.888	0.773	3.998E-20
TUG1	3.8E-33	0.5101956	1.665617	0.993	0.968	1.249E-28
USP44	4.92E-18	0.5097826	1.664929	0.133	0.014	1.62E-13
MAP3K20	4.42E-55	0.509753	1.66488	1	1	1.455E-50
EPS8L2	6.38E-13	0.5094167	1.66432	0.472	0.35	2.098E-08
INMT	9.74E-12	0.5090466	1.663704	0.14	0.056	3.205E-07
INS-IGF2	4.39E-65	0.508985	1.663602	0.367	0.016	1.443E-60
TCEAL1	1.15E-16	0.5087828	1.663266	0.785	0.715	3.789E-12
CHD4	2.17E-54	0.5084822	1.662765	1	0.979	7.134E-50
NFIA	3.82E-14	0.5069515	1.660222	0.558	0.49	1.258E-09
AP003068.2	1.92E-17	0.5067479	1.659884	0.189	0.049	6.313E-13

PRRG1	3.51E-12	0.5066278	1.659685	0.435	0.353	1.154E-07
ZNF292	9.72E-17	0.5062826	1.659112	0.752	0.629	3.197E-12
WDFY3	2.34E-14	0.5062606	1.659076	0.54	0.462	7.688E-10
MAP4K3	2.95E-14	0.5061968	1.65897	0.577	0.485	9.703E-10
TNFRSF10B	1.13E-18	0.5059571	1.658572	0.841	0.733	3.73E-14
PKD2	1.41E-20	0.505832	1.658365	0.925	0.838	4.635E-16
ARID4A	1.76E-11	0.5056357	1.658039	0.514	0.383	5.78E-07
VGLL3	4.13E-30	0.5051087	1.657166	0.987	0.954	1.359E-25
ITGA11	9.64E-12	0.5049955	1.656978	0.689	0.64	3.172E-07
MBNL2	1.59E-28	0.5049698	1.656936	0.967	0.896	5.232E-24
CAND1	5.86E-28	0.504841	1.656722	0.976	0.937	1.927E-23
TPST1	1.06E-17	0.504385	1.655967	0.869	0.77	3.475E-13
BPTF	2.87E-27	0.5043762	1.655952	0.971	0.916	9.429E-23
PSMA5	3.05E-84	0.5039613	1.655265	1	1	1.002E-79
PI4KB	1.57E-21	0.5038906	1.655148	0.865	0.826	5.153E-17
COL4A5	1.31E-11	0.5035722	1.654621	0.268	0.158	4.305E-07
FZD6	1.04E-21	0.5030004	1.653675	0.939	0.872	3.428E-17
GRAMD2B	1.57E-15	0.5028468	1.653422	0.805	0.694	5.181E-11
MIPEP	7.57E-12	0.5024935	1.652837	0.558	0.497	2.491E-07
RTN2	2.32E-12	0.5020309	1.652073	0.399	0.271	7.625E-08
ZNF880	2.09E-13	0.5016673	1.651472	0.441	0.288	6.868E-09
MBNL1-AS1	7.48E-11	0.5010923	1.650523	0.517	0.443	2.463E-06
LYST	1.42E-15	0.5004151	1.649406	0.664	0.596	4.675E-11
KDM5A	1.34E-21	0.5001457	1.648962	0.91	0.798	4.405E-17
POGZ	1.29E-17	0.4996295	1.64811	0.793	0.684	4.253E-13
ADAR	1.25E-25	0.4992007	1.647404	0.961	0.928	4.119E-21
PSMB10	2.25E-20	0.4991677	1.64735	0.765	0.701	7.409E-16
PHF3	6.24E-18	0.498395	1.646077	0.885	0.775	2.054E-13
TP53INP1	4.3E-09	0.4981458	1.645667	0.355	0.248	0.0001416
STAT2	3.37E-16	0.4979984	1.645424	0.751	0.659	1.109E-11
TMEM140	9.59E-14	0.4979293	1.645311	0.16	0.051	3.154E-09
CMTM3	1.52E-19	0.4978734	1.645219	0.893	0.787	5.003E-15
DTX3	1.08E-13	0.4976806	1.644902	0.436	0.306	3.538E-09
AGO1	2.77E-13	0.4971379	1.644009	0.64	0.566	9.126E-09
MCAM	2.86E-12	0.4969638	1.643723	0.628	0.527	9.401E-08
STX6	1.18E-15	0.4969279	1.643664	0.629	0.557	3.888E-11
MFGE8	3.76E-16	0.496913	1.643639	0.981	0.958	1.238E-11
KMT5B	8.64E-16	0.4966786	1.643254	0.748	0.682	2.842E-11
GTF2E1	1.55E-10	0.4965384	1.643024	0.509	0.401	5.09E-06
MOSPD1	3.97E-14	0.4959822	1.64211	0.785	0.696	1.306E-09

SSBP2	5.96E-14	0.4947404	1.640072	0.717	0.643	1.96E-09
PDE9A	1E-21	0.4947089	1.640021	0.167	0.023	3.294E-17
CRY1	3.27E-12	0.4946634	1.639946	0.482	0.336	1.075E-07
TEAD1	1.47E-54	0.4946219	1.639878	1	1	4.842E-50
SNHG8	3.76E-21	0.4945532	1.639765	0.778	0.675	1.235E-16
EIF2S3	2.33E-61	0.4941871	1.639165	1	0.998	7.674E-57
KMT2C	2.76E-16	0.4939114	1.638713	0.753	0.708	9.091E-12
MYCBP2	5.11E-16	0.4938743	1.638653	0.839	0.722	1.682E-11
HUWE1	9.65E-29	0.4929675	1.637167	0.975	0.91	3.175E-24
SARM1	5.86E-10	0.4922122	1.635931	0.311	0.183	1.927E-05
UBR3	1.42E-14	0.4921561	1.635839	0.663	0.548	4.67E-10
TRIP12	7.52E-31	0.4919495	1.635501	0.986	0.947	2.473E-26
PRRC2C	1.18E-40	0.4918192	1.635288	0.997	0.984	3.876E-36
ZDHHC7	1.25E-24	0.4917974	1.635253	0.958	0.886	4.097E-20
SLC35E2B	1.44E-19	0.4915207	1.6348	0.729	0.631	4.724E-15
CRIM1-DT	8.38E-16	0.4915049	1.634775	0.449	0.309	2.757E-11
RGS5	1.84E-05	0.4913465	1.634516	0.202	0.155	0.6043785
NNT	3.6E-20	0.4913148	1.634464	0.917	0.877	1.184E-15
CACNA1H	4.93E-24	0.4907958	1.633616	0.168	0.016	1.622E-19
HACD4	1.16E-15	0.4906443	1.633368	0.812	0.733	3.827E-11
RB1CC1	6.89E-26	0.4905602	1.633231	0.966	0.9	2.268E-21
SSB	3.08E-58	0.4900973	1.632475	1	1	1.013E-53
FAR2	1.05E-10	0.4894797	1.631467	0.456	0.371	3.465E-06
CSRP1	1.71E-68	0.489428	1.631383	1	1	5.615E-64
PRKCA	3.44E-20	0.4892858	1.631151	0.955	0.9	1.133E-15
FDXR	1.21E-11	0.4891301	1.630897	0.483	0.346	3.995E-07
NPEPPS	1.55E-34	0.4889043	1.630529	0.988	0.951	5.11E-30
NLRP1	4.35E-11	0.4888733	1.630478	0.691	0.58	1.432E-06
ZNF638	4.72E-24	0.4885313	1.629921	0.933	0.84	1.552E-19
NEMF	1.7E-27	0.4880017	1.629058	0.972	0.905	5.595E-23
SLC27A3	1.11E-12	0.4879249	1.628933	0.254	0.107	3.667E-08
SPTBN1	2.82E-23	0.4873976	1.628074	0.98	0.944	9.285E-19
MYADM	1.32E-45	0.486859	1.627197	0.999	0.998	4.346E-41
MOSPD2	6.09E-14	0.4867378	1.627	0.867	0.803	2.005E-09
NCOA1	2.02E-13	0.486385	1.626426	0.561	0.392	6.64E-09
CBX6	9.39E-18	0.4863773	1.626413	0.9	0.807	3.091E-13
ARHGEF2	9.58E-18	0.4861174	1.625991	0.906	0.856	3.151E-13
PRSS12	5.76E-13	0.4858186	1.625505	0.632	0.585	1.895E-08
CPM	1.37E-10	0.4855822	1.625121	0.527	0.473	4.516E-06
AC087477.2	1.06E-18	0.4853345	1.624718	0.178	0.035	3.482E-14

GOLGA8B	7.09E-18	0.485104	1.624344	0.599	0.527	2.334E-13
MARCHF9	1.61E-13	0.4848778	1.623977	0.228	0.107	5.282E-09
SECISBP2L	1.91E-20	0.4847059	1.623697	0.896	0.798	6.298E-16
TPBG	3.54E-23	0.4841925	1.622864	0.99	0.97	1.165E-18
CUL5	8.37E-24	0.4841015	1.622716	0.971	0.916	2.754E-19
DGKH	2.74E-09	0.483712	1.622084	0.581	0.506	9.031E-05
BBC3	1.34E-14	0.483073	1.621048	0.434	0.258	4.392E-10
DTX3L	6.61E-14	0.4829301	1.620817	0.57	0.434	2.174E-09
ROGDI	2.61E-11	0.4828776	1.620732	0.459	0.339	8.574E-07
TBL1XR1	9.77E-23	0.482579	1.620248	0.952	0.912	3.215E-18
RIC1	2.4E-13	0.4824453	1.620031	0.592	0.499	7.9E-09
HTR7P1	3.18E-09	0.4823759	1.619919	0.296	0.186	0.0001047
HACD1	4.06E-14	0.4822681	1.619744	0.871	0.807	1.334E-09
BTN3A1	1.07E-09	0.482264	1.619737	0.43	0.313	3.531E-05
DHX36	1.58E-25	0.4818164	1.619012	0.98	0.928	5.196E-21
SOCS5	8.48E-30	0.4812687	1.618126	0.971	0.896	2.791E-25
SIDT2	1.13E-14	0.4804083	1.616734	0.588	0.506	3.723E-10
PITX1	1.95E-22	0.4803649	1.616664	0.382	0.515	6.422E-18
TAPBP	1.68E-20	0.4802857	1.616536	0.951	0.921	5.519E-16
TNRC6B	2.59E-13	0.4802724	1.616515	0.803	0.74	8.523E-09
MSMO1	5.19E-11	0.4799657	1.616019	0.863	0.856	1.708E-06
TRIM13	3.81E-12	0.4797243	1.615629	0.605	0.492	1.254E-07
GAS5	5.19E-37	0.4793503	1.615025	0.999	0.991	1.707E-32
MAP3K12	1.24E-15	0.4787806	1.614105	0.514	0.406	4.09E-11
RERE	2.52E-16	0.4786443	1.613885	0.841	0.749	8.304E-12
STX17	5.16E-14	0.4785921	1.613801	0.667	0.589	1.699E-09
LINC00672	8.21E-13	0.4784279	1.613536	0.266	0.139	2.703E-08
CLTC	7.8E-99	0.4784115	1.613509	1	1	2.569E-94
AK3	9.34E-31	0.4781044	1.613014	0.998	0.988	3.073E-26
HELZ	2.04E-11	0.4775969	1.612195	0.717	0.636	6.721E-07
EXOC6B	3.27E-12	0.4769338	1.611127	0.571	0.443	1.076E-07
PCM1	5.16E-21	0.476872	1.611027	0.942	0.861	1.699E-16
ADCY9	2.63E-13	0.4767182	1.610779	0.599	0.487	8.665E-09
LZTS2	4.05E-19	0.4765635	1.61053	0.844	0.738	1.333E-14
HEATR6	2.24E-12	0.4765251	1.610468	0.62	0.52	7.363E-08
CTIF	5.37E-19	0.4764935	1.610418	0.746	0.645	1.767E-14
AIG1	1.28E-19	0.4752534	1.608422	0.895	0.817	4.204E-15
RNF213	1.38E-15	0.4750404	1.608079	0.864	0.766	4.526E-11
THOC2	8.83E-23	0.4747269	1.607575	0.971	0.926	2.906E-18
LIMK2	4.86E-12	0.4741932	1.606717	0.481	0.406	1.6E-07

TRIM58	1.89E-08	0.4740674	1.606515	0.229	0.158	0.0006203
TMEM216	1.94E-10	0.4735811	1.605734	0.352	0.302	6.388E-06
MAP3K5	2.22E-13	0.4720762	1.603319	0.608	0.515	7.294E-09
PPP1R10	3.55E-15	0.4720024	1.603201	0.692	0.647	1.168E-10
MTMR2	1.53E-25	0.4718054	1.602885	0.973	0.928	5.029E-21
PGRMC2	2.75E-24	0.4717943	1.602868	0.999	0.998	9.032E-20
GSTA4	4.61E-12	0.4715375	1.602456	0.57	0.524	1.518E-07
CU633906.1	7.66E-29	0.4715084	1.602409	0.536	0.332	2.522E-24
RNF123	4.83E-10	0.4711922	1.601903	0.402	0.288	1.591E-05
NUDT3	1.26E-22	0.4710402	1.601659	0.964	0.951	4.144E-18
AZIN1	4.68E-32	0.4709811	1.601565	0.998	0.991	1.54E-27
LONP2	3.82E-24	0.4709493	1.601514	0.93	0.842	1.256E-19
MYOF	1.59E-63	0.4704985	1.600792	1	0.995	5.232E-59
MCC	8.21E-09	0.4703032	1.600479	0.242	0.142	0.00027
AC055839.2	1.29E-14	0.4702978	1.600471	0.513	0.339	4.249E-10
TRIM6	2.37E-12	0.4702662	1.60042	0.462	0.357	7.801E-08
CCDC6	5.23E-15	0.4701869	1.600293	0.915	0.872	1.721E-10
SMG1P5	1.69E-32	0.470033	1.600047	0.751	0.601	5.563E-28
EPHA2	1.64E-14	0.4697571	1.599606	0.817	0.729	5.398E-10
RC3H2	5.83E-16	0.469089	1.598537	0.803	0.687	1.919E-11
PPP1CB	3.46E-66	0.4689504	1.598316	1	1	1.137E-61
WDR11	6.1E-16	0.4688673	1.598183	0.708	0.661	2.006E-11
HEY1	6.46E-08	0.4684861	1.597574	0.22	0.125	0.0021265
ZNF827	7.77E-14	0.4679705	1.59675	0.727	0.652	2.558E-09
JPX	2.44E-24	0.4674205	1.595872	0.868	0.856	8.036E-20
SUPT6H	9.3E-19	0.4673509	1.595761	0.839	0.766	3.059E-14
IREB2	1.92E-18	0.4672545	1.595607	0.908	0.847	6.325E-14
ARHGEF25	1.59E-11	0.4671614	1.595459	0.49	0.385	5.228E-07
PIP4K2C	1.19E-09	0.4671062	1.595371	0.391	0.267	3.914E-05
LOXL4	1.89E-09	0.466866	1.594988	0.307	0.195	6.225E-05
H6PD	7.51E-15	0.4668011	1.594884	0.779	0.717	2.471E-10
PTGR1	5.63E-17	0.4667613	1.594821	0.978	0.947	1.852E-12
FNDC3A	2.41E-15	0.4667048	1.594731	0.911	0.858	7.919E-11
SLFN5	1.6E-19	0.4659551	1.593535	0.949	0.884	5.259E-15
ADGRA2	3.28E-20	0.4657632	1.59323	0.945	0.919	1.08E-15
LRRC8D	4.49E-16	0.4655445	1.592881	0.718	0.615	1.478E-11
FAM13A	1.05E-09	0.4654338	1.592705	0.563	0.487	3.439E-05
PSPH	5.74E-16	0.4653555	1.59258	0.813	0.731	1.887E-11
RARA-AS1	4.92E-16	0.4652039	1.592339	0.157	0.035	1.618E-11
AC090409.1	7.28E-11	0.4650752	1.592134	0.459	0.369	2.396E-06

RASL11A	1.04E-09	0.4650752	1.592134	0.194	0.107	3.43E-05
SCARB1	2.05E-11	0.4648904	1.59184	0.474	0.357	6.745E-07
HOXA3	7.46E-21	0.4646803	1.591505	0.179	0.03	2.454E-16
C5orf15	1.05E-21	0.4636866	1.589925	0.975	0.937	3.459E-17
CNTNAP1	8E-14	0.4636035	1.589792	0.507	0.397	2.631E-09
RNF41	2.6E-17	0.4629295	1.588721	0.817	0.798	8.55E-13
SERPINH1	4.9E-125	0.4627235	1.588394	1	1	1.62E-120
F2RL2	1.15E-08	0.4626374	1.588257	0.469	0.364	0.0003791
PLCG1	2.15E-11	0.4623871	1.58786	0.577	0.499	7.067E-07
SPG11	1.94E-13	0.4621856	1.58754	0.642	0.564	6.387E-09
PTK7	2.17E-15	0.4616874	1.586749	0.944	0.916	7.141E-11
GBE1	7.28E-28	0.4608791	1.585467	0.986	0.968	2.396E-23
TULP4	5.94E-12	0.4604534	1.584792	0.599	0.508	1.956E-07
DSE	2.21E-20	0.4603291	1.584595	0.969	0.914	7.276E-16
EID1	2E-134	0.460206	1.5844	1	1	6.43E-130
UBXN7	1.3E-15	0.4601783	1.584356	0.8	0.698	4.292E-11
TAF7	5.22E-30	0.4601611	1.584329	0.991	0.979	1.719E-25
PTPN13	6.61E-17	0.4595585	1.583375	0.756	0.636	2.175E-12
GTF3C1	1.83E-15	0.4595267	1.583324	0.781	0.668	6.013E-11
SLC12A2	1.96E-16	0.4593778	1.583089	0.76	0.698	6.461E-12
SH3PXD2A	5.2E-19	0.4582932	1.581373	0.978	0.944	1.712E-14
KIAA1109	9.24E-15	0.4575008	1.58012	0.757	0.689	3.039E-10
DCAF5	1.12E-14	0.4574681	1.580068	0.776	0.703	3.696E-10
BST1	7.92E-11	0.4572841	1.579778	0.543	0.441	2.606E-06
AFAP1L2	5.84E-14	0.4566363	1.578755	0.146	0.032	1.922E-09
AASDHPPT	1.49E-20	0.4565314	1.578589	0.96	0.914	4.899E-16
SH3BGRL	5.75E-25	0.4562544	1.578152	0.993	0.981	1.892E-20
DYNC1H1	2.81E-36	0.4562397	1.578129	0.998	0.993	9.239E-32
AC013271.1	2.08E-53	0.4561065	1.577918	0.351	0.03	6.844E-49
SOCS2	1.76E-12	0.4556365	1.577177	0.668	0.599	5.776E-08
TSPYL1	9.75E-19	0.4556175	1.577147	0.937	0.886	3.206E-14
MAMDC2	1.52E-09	0.4554904	1.576947	0.148	0.051	5.001E-05
PM20D2	6.63E-11	0.4554747	1.576922	0.464	0.355	2.182E-06
MED12	6.22E-11	0.4554696	1.576914	0.471	0.397	2.047E-06
BOLA2-SMG1	1.78E-30	0.455354	1.576731	0.916	0.865	5.859E-26
NOTCH3	1.82E-08	0.4550943	1.576322	0.74	0.708	0.0005973
SNTB1	2.84E-09	0.455036	1.57623	0.355	0.218	9.328E-05
ZC3H13	7.07E-19	0.4549821	1.576145	0.951	0.896	2.325E-14
POLR3GL	1.62E-15	0.4549814	1.576144	0.718	0.65	5.333E-11
BCL3	5.48E-12	0.4548366	1.575916	0.328	0.204	1.803E-07

TNPO1	2.76E-39	0.454687	1.57568	1	0.993	9.096E-35
TCAF1	3.26E-19	0.4546153	1.575567	0.958	0.916	1.071E-14
SMARCD3	4.9E-13	0.4545269	1.575428	0.652	0.589	1.611E-08
IGF2BP1	1.94E-07	0.4538282	1.574327	0.447	0.357	0.0063723
TFPI	1.48E-23	0.4537798	1.574251	0.985	0.94	4.869E-19
CHML	1.42E-14	0.4531999	1.573339	0.663	0.613	4.688E-10
H2BC21	1.05E-14	0.452979	1.572991	0.141	0.026	3.444E-10
OR2A20P	3.83E-23	0.4529182	1.572895	0.181	0.023	1.261E-18
ZCRB1	3.15E-35	0.4524524	1.572163	0.975	0.942	1.037E-30
GDF11	1.26E-11	0.4520362	1.571509	0.507	0.452	4.142E-07
HECTD1	4.61E-24	0.4520031	1.571457	0.969	0.898	1.516E-19
PDLIM1	5.38E-49	0.451764	1.571081	1	1	1.769E-44
USP47	1.79E-22	0.4513651	1.570454	0.972	0.921	5.874E-18
TCF12	4.04E-24	0.450703	1.569415	0.981	0.958	1.33E-19
DECR2	1.17E-09	0.450701	1.569412	0.532	0.464	3.836E-05
TET1	3.53E-12	0.4503097	1.568798	0.251	0.114	1.163E-07
BMP4	4.19E-15	0.4495994	1.567684	0.126	0.016	1.379E-10
HEATR5A	1.5E-13	0.4492108	1.567075	0.798	0.761	4.923E-09
LINC00475	2.78E-14	0.4491674	1.567007	0.19	0.058	9.15E-10
MIA2	2.45E-11	0.4490719	1.566857	0.555	0.499	8.068E-07
NBR1	5.05E-20	0.4485165	1.565987	0.96	0.921	1.661E-15
SNRK	5.58E-09	0.4481705	1.565446	0.431	0.318	0.0001834
TMSB4X	3.6E-132	0.4478785	1.564988	1	1	1.2E-127
BIN1	4.48E-16	0.4477064	1.564719	0.601	0.538	1.475E-11
HLA-C	1.7E-53	0.447703	1.564714	1	1	5.599E-49
FOSL2	2.05E-18	0.4475043	1.564403	0.92	0.849	6.734E-14
OSBPL10	3.42E-10	0.4466243	1.563027	0.297	0.176	1.127E-05
KDM6B	3.89E-13	0.4464868	1.562812	0.445	0.297	1.28E-08
SLC38A2	5.67E-52	0.446466	1.562779	1	1	1.866E-47
CCL2	0.00096	0.4458687	1.561846	0.206	0.165	1
AC079848.2	6.61E-13	0.4456831	1.561557	0.172	0.051	2.176E-08
DOCK6	3.03E-09	0.4446431	1.559933	0.412	0.329	9.961E-05
TPD52	3.74E-12	0.4444614	1.55965	0.112	0.023	1.23E-07
SCN2A	2.01E-12	0.4443477	1.559473	0.181	0.072	6.62E-08
CC2D2A	3.19E-12	0.4441813	1.559213	0.638	0.555	1.051E-07
PPHLN1	4.03E-22	0.4440073	1.558942	0.977	0.944	1.327E-17
SNHG5	7.78E-64	0.4439922	1.558918	1	1	2.561E-59
MFAP4	3.19E-09	0.443812	1.558637	0.588	0.548	0.0001049
ADA2	5.28E-12	0.4437244	1.558501	0.235	0.153	1.738E-07
RGS10	1.44E-18	0.4436578	1.558397	0.931	0.896	4.73E-14

RTP4	1.85E-11	0.4436518	1.558388	0.149	0.042	6.077E-07
ELF1	1.38E-13	0.4430912	1.557514	0.784	0.777	4.539E-09
SHFL	6.61E-11	0.4429817	1.557344	0.602	0.555	2.175E-06
PPP1R15B	5.07E-17	0.4429616	1.557313	0.854	0.773	1.668E-12
DGKI	2.12E-05	0.4429271	1.557259	0.354	0.336	0.6975308
LTN1	5.94E-12	0.4428506	1.55714	0.834	0.784	1.955E-07
YPEL2	2.18E-08	0.4428179	1.557089	0.218	0.104	0.0007178
DACT1	3.26E-07	0.4426809	1.556876	0.252	0.146	0.0107287
TNS1	8.63E-13	0.4421011	1.555973	0.919	0.877	2.838E-08
FARP1	8.97E-40	0.4419807	1.555786	1	0.993	2.952E-35
DDHD1	3.12E-14	0.4413121	1.554746	0.64	0.613	1.026E-09
ORAI3	5.26E-10	0.4409548	1.55419	0.395	0.267	1.732E-05
TJP2	1.12E-12	0.4405514	1.553564	0.668	0.636	3.698E-08
ZFC3H1	1.31E-09	0.4405343	1.553537	0.664	0.573	4.32E-05
EIF2AK4	1.68E-24	0.4401698	1.552971	0.969	0.935	5.539E-20
KMT2E	1.95E-16	0.4400503	1.552785	0.955	0.926	6.406E-12
CDKN1C	5.23E-16	0.4397813	1.552368	0.249	0.09	1.72E-11
NLGN2	8.19E-13	0.4397693	1.552349	0.603	0.497	2.695E-08
AC005670.3	1.32E-16	0.4397662	1.552344	0.721	0.659	4.351E-12
CU633904.1	7.95E-25	0.439755	1.552327	0.529	0.332	2.615E-20
SHC3	1.71E-11	0.439715	1.552265	0.148	0.039	5.635E-07
LRP6	2.77E-09	0.4396741	1.552201	0.56	0.469	9.122E-05
TENM3	3.43E-16	0.4392545	1.55155	0.644	0.45	1.128E-11
ARID4B	6.8E-18	0.4392302	1.551512	0.956	0.914	2.238E-13
PLEKHA6	4.54E-14	0.4391612	1.551405	0.143	0.035	1.494E-09
BCRP2	2.82E-14	0.4390669	1.551259	0.184	0.067	9.285E-10
AFF4	8.04E-20	0.4388785	1.550967	0.968	0.916	2.646E-15
AIFM2	4.18E-10	0.4387978	1.550842	0.638	0.575	1.374E-05
DIPK1A	1.04E-16	0.438771	1.5508	0.889	0.784	3.411E-12
TIMP2	1.05E-89	0.4386967	1.550685	1	1	3.46E-85
AC009093.1	7.95E-11	0.4386438	1.550603	0.215	0.1	2.615E-06
GRK3	1.41E-09	0.4383009	1.550071	0.223	0.155	4.652E-05
CUL7	1.89E-11	0.438206	1.549924	0.575	0.515	6.216E-07
PRKAR1A	1.2E-46	0.4380005	1.549606	0.999	0.998	3.944E-42
YTHDC1	6.97E-14	0.437864	1.549394	0.898	0.831	2.294E-09
NXPE3	4.51E-14	0.4378345	1.549349	0.782	0.71	1.484E-09
C8orf31	3.7E-11	0.4376207	1.549017	0.209	0.084	1.217E-06
COL25A1	8.65E-17	0.4367571	1.54768	0.106	0.005	2.847E-12
EEA1	2.04E-24	0.436754	1.547675	0.992	0.974	6.704E-20
FGD4	6.01E-10	0.4367189	1.547621	0.203	0.097	1.978E-05

CD4	2.4E-13	0.4365152	1.547306	0.115	0.019	7.912E-09
DNAJB14	4.98E-21	0.4363477	1.547047	0.949	0.858	1.64E-16
CD27-AS1	5.15E-14	0.4354058	1.54559	0.629	0.587	1.693E-09
TBC1D19	1.06E-08	0.4350916	1.545105	0.396	0.32	0.0003491
DOCK4	4.74E-08	0.435048	1.545037	0.262	0.19	0.001559
ATN1	1.61E-22	0.4344597	1.544129	0.966	0.942	5.287E-18
SNX27	1.09E-20	0.4342563	1.543815	0.93	0.93	3.576E-16
PDE1A	6.4E-16	0.4331555	1.542116	0.138	0.019	2.105E-11
CEACAM19	5.1E-14	0.432851	1.541647	0.223	0.09	1.678E-09
RAB29	1.16E-12	0.432803	1.541572	0.643	0.529	3.809E-08
PRKACB	3.53E-16	0.4326279	1.541303	0.871	0.796	1.163E-11
AGL	1.81E-10	0.4323433	1.540864	0.526	0.462	5.971E-06
CLK1	3.68E-12	0.4318495	1.540103	0.691	0.626	1.211E-07
ARID2	8.45E-14	0.4312923	1.539245	0.524	0.485	2.781E-09
SMARCC2	1.73E-15	0.4304279	1.537916	0.931	0.886	5.693E-11
DOK4	1.31E-09	0.4303796	1.537841	0.554	0.469	4.314E-05
TSTD3	3.11E-14	0.4302195	1.537595	0.491	0.413	1.025E-09
CNNM2	8.99E-12	0.4300704	1.537366	0.362	0.276	2.959E-07
LRRFIP1	1.16E-39	0.4299906	1.537243	1	1	3.831E-35
CHP1	6.54E-18	0.4299411	1.537167	0.96	0.94	2.151E-13
GPC4	1.51E-05	0.4298288	1.536994	0.141	0.07	0.4984494
KIAA0319L	6.92E-15	0.4291432	1.535941	0.648	0.629	2.277E-10
THYN1	2.93E-10	0.4288891	1.535551	0.735	0.647	9.645E-06
ANKRD33B	3.9E-08	0.4288498	1.53549	0.205	0.102	0.0012844
DCP1A	5.23E-13	0.4288063	1.535424	0.719	0.684	1.72E-08
ZNF469	6.56E-14	0.4286083	1.53512	0.311	0.146	2.16E-09
SH3BP1	3.28E-13	0.4285748	1.535068	0.284	0.153	1.081E-08
RBM23	1.42E-17	0.4284319	1.534849	0.819	0.777	4.683E-13
LTBP3	4.41E-16	0.4281643	1.534438	0.767	0.645	1.452E-11
TERF1	9.19E-16	0.4279759	1.534149	0.943	0.898	3.024E-11
BRWD3	4.88E-07	0.4276275	1.533615	0.347	0.253	0.0160617
SLC35F6	1.88E-12	0.4272251	1.532998	0.795	0.705	6.17E-08
FAM124A	1.48E-14	0.4271394	1.532866	0.161	0.037	4.874E-10
G3BP2	5.71E-25	0.4269336	1.532551	0.994	0.984	1.878E-20
AC118553.2	8.75E-17	0.4268162	1.532371	0.889	0.858	2.878E-12
PAPPA	0.000369	0.4267037	1.532199	0.697	0.675	1
WBP2	3.3E-17	0.4263999	1.531733	0.794	0.708	1.086E-12
SLC16A14	5.08E-17	0.4258492	1.53089	0.132	0.014	1.673E-12
KIAA0513	1.15E-10	0.4255375	1.530413	0.343	0.227	3.772E-06
POLB	3.35E-09	0.4253334	1.5301	0.514	0.432	0.0001101

PHKB	5.86E-14	0.4250319	1.529639	0.863	0.796	1.928E-09
AKNA	2.03E-10	0.4248924	1.529426	0.349	0.258	6.677E-06
ADD3	1.73E-17	0.4247696	1.529238	0.967	0.93	5.692E-13
ZBTB46	3.8E-18	0.4247444	1.529199	0.127	0.009	1.25E-13
PARP9	1.13E-09	0.4247188	1.52916	0.513	0.42	3.703E-05
GPRC5B	4.98E-09	0.4245624	1.528921	0.172	0.07	0.0001639
PRRT1	2.25E-14	0.4242906	1.528506	0.208	0.063	7.399E-10
TIA1	1.19E-17	0.4241206	1.528246	0.978	0.956	3.92E-13
TXLNG	1.52E-09	0.4238552	1.52784	0.571	0.522	5.007E-05
RO60	6.74E-21	0.423508	1.52731	0.97	0.928	2.216E-16
SMAD2	1.45E-19	0.4233465	1.527063	0.97	0.947	4.769E-15
FBXL17	1.8E-11	0.4232499	1.526916	0.642	0.541	5.923E-07
USP34	1.25E-12	0.4231385	1.526746	0.871	0.794	4.11E-08
RYBP	1.46E-14	0.4231317	1.526735	0.937	0.9	4.805E-10
GABPB1-AS1	4.71E-12	0.4227755	1.526192	0.662	0.592	1.55E-07
FNBP1	4.8E-14	0.4226857	1.526055	0.743	0.68	1.579E-09
UBR5	3.55E-11	0.4226577	1.526012	0.835	0.752	1.169E-06
RPS4X	8.5E-170	0.4223848	1.525595	1	1	2.78E-165
PJA1	4.47E-10	0.4223557	1.525551	0.53	0.476	1.471E-05
CU634019.1	8.32E-24	0.4221774	1.525279	0.521	0.306	2.738E-19
SEC23IP	1.56E-12	0.4221605	1.525253	0.906	0.852	5.137E-08
PTPRK	3.04E-15	0.4219351	1.52491	0.847	0.782	9.991E-11
PEX1	8.2E-09	0.421175	1.523751	0.4	0.341	0.00027
CREBBP	1.51E-12	0.4209369	1.523388	0.789	0.733	4.958E-08
AKR1B1	3.76E-28	0.4209254	1.523371	1	0.995	1.237E-23
CCDC170	3.16E-16	0.4207202	1.523058	0.13	0.016	1.038E-11
BOK	1.97E-14	0.4205179	1.52275	0.844	0.759	6.466E-10
IGF1R	9.71E-13	0.41919	1.520729	0.684	0.661	3.194E-08
LINC00847	1.73E-07	0.4190982	1.52059	0.228	0.139	0.0056798
PRDX6	1.85E-52	0.4189485	1.520362	1	1	6.09E-48
CLDN11	1.35E-07	0.4186688	1.519937	0.904	0.854	0.0044504
AMMECR1	4.92E-11	0.4184481	1.519601	0.736	0.668	1.619E-06
TCEAL7	3.24E-09	0.418292	1.519364	0.478	0.334	0.0001067
CAP2	3.62E-14	0.418035	1.518974	0.915	0.847	1.192E-09
SERPINF1	5.27E-10	0.4180066	1.518931	0.654	0.613	1.734E-05
PHF21A	1.88E-12	0.4170965	1.517549	0.584	0.501	6.172E-08
TMEM164	8.66E-08	0.4169918	1.51739	0.352	0.271	0.0028491
ADCY6	7.17E-10	0.4167551	1.517031	0.486	0.429	2.359E-05
TSPYL4	3.98E-11	0.4165966	1.516791	0.59	0.527	1.309E-06
UBP1	8.58E-13	0.4160274	1.515927	0.897	0.854	2.823E-08

RILPL2	1.06E-11	0.4157581	1.515519	0.675	0.622	3.475E-07
ANKRD17	3E-18	0.4157236	1.515467	0.94	0.858	9.886E-14
ABCC1	1.46E-11	0.415708	1.515443	0.663	0.578	4.818E-07
SYT1	1.73E-13	0.4153084	1.514838	0.13	0.023	5.696E-09
KDM4B	6.63E-11	0.4151951	1.514666	0.645	0.566	2.182E-06
SLC2A3	1.12E-12	0.4150612	1.514463	0.745	0.726	3.701E-08
NID2	1.21E-11	0.4146351	1.513818	0.878	0.828	3.974E-07
VPS13A	1.8E-08	0.4144403	1.513523	0.617	0.52	0.0005909
UBE2H	1.34E-15	0.4139921	1.512845	0.963	0.928	4.413E-11
RBMXL1	4.3E-12	0.4139316	1.512754	0.603	0.589	1.415E-07
AOX1	4.05E-09	0.4138417	1.512618	0.611	0.508	0.0001332
POLR2B	3.74E-20	0.4136565	1.512338	0.977	0.937	1.232E-15
YPEL5	1.08E-12	0.4135867	1.512232	0.949	0.947	3.548E-08
CYFIP2	5.29E-07	0.4133562	1.511883	0.166	0.084	0.017414
SPIN1	3.8E-36	0.4132197	1.511677	0.999	0.981	1.25E-31
PAIP2	2.81E-19	0.4131596	1.511586	0.978	0.956	9.251E-15
PLPPR4	1.54E-07	0.4126918	1.510879	0.192	0.1	0.0050639
PRRC2B	2.24E-13	0.4124814	1.510562	0.897	0.87	7.354E-09
ZNF605	4.34E-08	0.4122983	1.510285	0.543	0.473	0.0014283
ZNF480	5.9E-09	0.4118968	1.509679	0.538	0.457	0.0001942
TPM1	7.5E-106	0.4115088	1.509093	1	1	2.47E-101
WDR41	1.89E-13	0.4110222	1.508359	0.923	0.893	6.222E-09
ASB7	1.88E-08	0.4108547	1.508106	0.46	0.404	0.0006179
SYNRG	6.2E-11	0.4108326	1.508073	0.713	0.629	2.04E-06
NOTCH1	1.27E-07	0.4107864	1.508003	0.388	0.292	0.0041813
IRGQ	5.69E-11	0.4105173	1.507597	0.502	0.378	1.874E-06
LDB1	7.76E-19	0.4104869	1.507552	0.924	0.94	2.554E-14
NUMA1	4.42E-17	0.4104495	1.507495	0.945	0.898	1.454E-12
MEF2C	1.14E-06	0.4103908	1.507407	0.423	0.313	0.0374276
CUTA	1.31E-69	0.4103487	1.507343	1	1	4.319E-65
TBL1X	3.82E-11	0.4102255	1.507158	0.893	0.826	1.257E-06
OSBPL9	1.51E-13	0.4101026	1.506972	0.905	0.889	4.966E-09
WARS1	3.25E-14	0.4100944	1.50696	0.979	0.979	1.068E-09
HRH1	4.21E-10	0.4098466	1.506587	0.71	0.608	1.385E-05
B2M	4.1E-111	0.4097048	1.506373	1	1	1.34E-106
IARS1	3.24E-31	0.4096739	1.506326	0.999	0.995	1.065E-26
ACVR2A	2.47E-08	0.4096704	1.506321	0.253	0.158	0.0008121
RAB33A	3.1E-07	0.4092559	1.505697	0.143	0.067	0.0101991
ZNF436	1.67E-07	0.4091712	1.50557	0.251	0.151	0.0054933
CTF1	1.55E-08	0.4091159	1.505486	0.321	0.23	0.0005105

CROT	5.57E-10	0.408897	1.505157	0.523	0.476	1.834E-05
XPC	7.85E-11	0.408173	1.504067	0.734	0.687	2.584E-06
NCKAP5L	8.17E-12	0.4076369	1.503261	0.415	0.295	2.689E-07
RNF146	5.29E-11	0.4076286	1.503249	0.597	0.575	1.74E-06
BCL9	1.47E-07	0.407201	1.502606	0.222	0.125	0.0048396
TRIM38	1.77E-10	0.4070747	1.502416	0.683	0.647	5.838E-06
FP236241.1	1.61E-22	0.4067989	1.502002	0.518	0.32	5.293E-18
CLASP1	3.57E-11	0.406605	1.501711	0.608	0.506	1.174E-06
PAPSS1	1.05E-14	0.4060236	1.500838	0.896	0.877	3.438E-10
AC040162.1	6.19E-17	0.4058132	1.500522	0.579	0.455	2.038E-12
LEPROT	3.07E-30	0.4055528	1.500132	0.999	0.995	1.01E-25
CCDC102A	2.07E-09	0.4055057	1.500061	0.252	0.135	6.806E-05

Upregulated in the small subpopulation than in the large subpopulation

GeneSymbol	p-value	avg_logFC	FC	pct.1	pct.2	p_val_adj
RPS4Y1	0	-3.698737	0.024755	0.165	0.998	0
MMP3	0	-3.534051	0.029186	0.953	1	0
ENPP2	0	-3.208406	0.040421	0.208	0.977	0
CXCL1	2.1E-202	-3.182567	0.041479	0.098	0.682	6.91E-198
DIRAS3	9.1E-286	-3.166845	0.042136	0.143	0.833	2.98E-281
CXCL8	8E-142	-2.951316	0.052271	0.426	0.807	2.62E-137
HAS2	9.4E-288	-2.939355	0.0529	0.402	0.91	3.1E-283
PODXL	0	-2.931753	0.053303	0.596	0.97	0
MMP1	3.57E-170	-2.93006	0.053394	0.276	0.773	1.17E-165
FAM180A	0.00E+00	-2.871774	0.056598	0.123	0.951	0.00E+00
CRABP2	0	-2.780109	0.062032	0.379	0.981	0
LRRC15	0	-2.704811	0.066883	0.075	0.919	0
NRN1	0	-2.667487	0.069426	0.044	0.963	0
TGFBI	0	-2.64084	0.071301	0.973	1	0
TIMP3	0	-2.478183	0.083896	0.929	1	0
HMOX1	8.4E-274	-2.475155	0.08415	0.51	0.974	2.77E-269
SGCD	0	-2.366852	0.093775	0.256	0.97	0
KCNMA1	1.5E-306	-2.364017	0.094042	0.246	0.951	4.79E-302
TENM2	0	-2.357932	0.094616	0.088	0.912	0
TSPAN13	1.5E-299	-2.342902	0.096049	0.249	0.951	4.9E-295
LY6K	5E-238	-2.321334	0.098143	0.177	0.868	1.63E-233
BAALC	0	-2.316332	0.098635	0.079	0.928	0
ANPEP	0	-2.31109	0.099153	0.893	1	0
CCBE1	0	-2.284939	0.10178	0.5	0.998	0
IGFBP3	2E-273	-2.260004	0.10435	0.97	1	6.54E-269
SLIT3	7.37496730	-2.23975	0.106485	0.108	0.903	2.42643799204315e-3
CCDC80	0	-2.222975	0.108287	0.997	1	0
IL13RA2	2.5E-216	-2.144693	0.117104	0.075	0.747	8.3E-212
THY1	2.9914969[illegible]	-2.12503	0.119429	0.68	0.998	9.84232402673477e-3
AKR1C1	1.8E-201	-2.119352	0.120109	0.24	0.861	5.85E-197
TMEM158	3.3E-243	-2.07427	0.125648	0.469	0.942	1.1E-238
ADGRL4	4.8E-234	-2.033506	0.130876	0.126	0.838	1.57E-229
CRIP2	5.2888760[illegible]	-2.014426	0.133397	0.375	0.981	1.74E-306
SERPINB2	1.96E-39	-2.008592	0.134178	0.26	0.455	6.43E-35
GABBR2	1.3E-222	-2.005594	0.13458	0.058	0.729	4.24E-218
DDX3Y	8.1E-305	-1.989625	0.136747	0.031	0.814	2.67E-300
ABI3BP	3.1E-207	-1.960429	0.140798	0.25	0.865	1.02E-202

PSG5	4.1E-276	-1.946585	0.142761	0.798	0.993	1.34E-271
TMEM119	4.1E-217	-1.945574	0.142905	0.15	0.863	1.35E-212
CPXM2	3.58749201	-1.936372	0.144226	0.026	0.817	1.18E-306
HOXC10	2.47032822	-1.914594	0.147402	0.037	0.87	8.12762690691143e-3
NTNG1	1.5E-305	-1.88998	0.151075	0.046	0.858	4.91E-301
ACTC1	2.61E-48	-1.878666	0.152794	0.082	0.332	8.578E-44
PLPP3	6.22E-161	-1.866272	0.154699	0.366	0.872	2.05E-156
AKR1C2	6.4E-205	-1.821274	0.161819	0.137	0.81	2.11E-200
CXCL6	1.64E-70	-1.798252	0.165588	0.04	0.325	5.401E-66
PRRX2	7.3E-244	-1.790999	0.166793	0.361	0.97	2.42E-239
CDCP1	1.27E-152	-1.785239	0.167757	0.105	0.633	4.17E-148
HOXB6	2.3E-277	-1.781844	0.168328	0.036	0.796	7.57E-273
ALDH1A3	1.7E-113	-1.769325	0.170448	0.205	0.677	5.51E-109
ELN	1.11E-180	-1.763109	0.171511	0.177	0.759	3.65E-176
EIF1AY	2.27E-297	-1.760769	0.171913	0.05	0.833	7.45E-293
COL18A1	2.1E-281	-1.759989	0.172047	0.506	0.986	6.79E-277
NGF	1.08E-164	-1.753047	0.173245	0.093	0.694	3.56E-160
LPXN	1.20E-207	-1.751269	0.173554	0.271	0.886	3.95E-203
NGFR	2.6E-152	-1.71031	0.18081	0.029	0.508	8.56E-148
DNAJC15	6.6E-279	-1.696478	0.183328	0.231	0.993	2.17E-274
COLEC12	1.24E-241	-1.690631	0.184403	0.303	0.858	4.09E-237
EMB	5.3E-219	-1.680638	0.186255	0.024	0.654	1.75E-214
ITGA8	3.3E-164	-1.670764	0.188103	0.167	0.78	1.08E-159
COMP	6.87E-75	-1.649509	0.192144	0.05	0.341	2.26E-70
PTGER3	4.8E-199	-1.648865	0.192268	0.054	0.696	1.59E-194
NRG1	1.36E-162	-1.638537	0.194264	0.561	0.933	4.46E-158
PRPS1	2.3E-217	-1.629371	0.196053	0.874	0.988	7.43E-213
STEAP1	1.31E-173	-1.627403	0.196439	0.245	0.872	4.30E-169
OLFML2B	2.7E-164	-1.622154	0.197473	0.155	0.773	8.77E-160
KRTAP2-3	5.74E-66	-1.60675	0.200538	0.14	0.492	1.89E-61
PTGFR	1.2E-154	-1.604848	0.20092	0.206	0.81	3.81E-150
APCDD1	2.7E-117	-1.596482	0.202608	0.132	0.65	8.96E-113
GRP	5E-128	-1.595018	0.202905	0.021	0.434	1.63E-123
TAFA5	1.23E-207	-1.583835	0.205187	0.092	0.782	4.04E-203
TGFBR3	2.2E-176	-1.571186	0.207799	0.137	0.789	7.23E-172
NKX3-1	1.2E-124	-1.570925	0.207853	0.139	0.652	3.88E-120
CLMP	1E-289	-1.569405	0.208169	0.94	1	3.41E-285
S100A4	3.11E-265	-1.561863	0.209745	0.989	1	1.02E-260
CELF2	2.11E-202	-1.560581	0.210014	0.015	0.585	6.93E-198
LOX	4.33295750	-1.556713	0.210828	1	1	1.43E-306

ASS1	5.9E-137	-1.537526	0.214912	0.163	0.754	1.95E-132
PITX2	3E-191	-1.532175	0.216065	0.018	0.58	9.93E-187
LINC00707	3E-119	-1.525701	0.217469	0.093	0.536	1E-114
STXBP6	6.1E-172	-1.519522	0.218816	0.059	0.661	2.02E-167
HS3ST3A1	4.4E-220	-1.507849	0.221386	0.464	0.951	1.46E-215
ITGBL1	7.5E-209	-1.502274	0.222623	0.622	0.991	2.46E-204
GDNF	2.10E-145	-1.490602	0.225237	0.05	0.531	6.90E-141
ARSI	1.4E-144	-1.487119	0.226023	0.219	0.807	4.61E-140
MIR31HG	7.14E-163	-1.48075	0.227467	0.111	0.724	2.35E-158
CRYAB	1.61E-149	-1.478646	0.227946	0.349	0.91	5.31E-145
RUNX3	3.81E-206	-1.476581	0.228417	0.026	0.622	1.25E-201
IL1R1	4.7E-175	-1.47544	0.228678	0.313	0.882	1.54E-170
KCTD12	1.7E-156	-1.471945	0.229479	0.109	0.71	5.61E-152
FOSL1	6.83E-219	-1.469816	0.229968	0.805	0.991	2.25E-214
PCOLCE2	1.7E-181	-1.467514	0.230498	0.018	0.548	5.74E-177
SERPINE1	1.5E-209	-1.466916	0.230636	0.999	1	4.89E-205
TWIST1	7.37E-202	-1.462971	0.231547	0.425	0.947	2.42E-197
SEMA7A	5.77E-171	-1.460265	0.232175	0.576	0.944	1.90E-166
USP9Y	3.26E-203	-1.450477	0.234458	0.056	0.687	1.07E-198
RFX8	1.6E-206	-1.448696	0.234876	0.043	0.689	5.33E-202
METRNL	1.84E-216	-1.447382	0.235185	0.724	0.991	6.05E-212
SPHK1	1.4E-194	-1.445835	0.235549	0.558	0.984	4.48E-190
PMEPA1	6.4E-173	-1.42095	0.241484	0.082	0.701	2.12E-168
CRIP1	3.11E-181	-1.420809	0.241519	0.344	0.87	1.02E-176
WNT5B	1.3E-227	-1.40247	0.245989	0.912	0.998	4.41E-223
TFAP2C	5.49E-160	-1.398726	0.246911	0.154	0.794	1.81E-155
PNP	4.7E-189	-1.393068	0.248312	0.629	0.97	1.55E-184
MFAP5	2.17E-39	-1.392788	0.248382	0.089	0.262	7.126E-35
CXCL5	1.89E-42	-1.384052	0.250561	0.033	0.225	6.203E-38
EMP1	0	-1.380058	0.251564	1	1	0
GNG2	7.52E-152	-1.378269	0.252014	0.322	0.882	2.47E-147
DDIT4L	8.75E-55	-1.37522	0.252784	0.07	0.343	2.88E-50
COL6A1	0	-1.361635	0.256242	1	1	0
COL6A2	0.00E+00	-1.359278	0.256846	1	1	0.00E+00
IRX2	1.3E-209	-1.357745	0.25724	0.015	0.608	4.38E-205
FOXF2	6.8E-174	-1.352137	0.258687	0.015	0.524	2.22E-169
IFI44	1.10E-134	-1.351256	0.258915	0.119	0.677	3.62E-130
UAP1	5.41E-183	-1.350113	0.259211	0.924	0.995	1.78E-178
PAQR5	2.4E-149	-1.348518	0.259625	0.103	0.687	7.82E-145
LINC01615	3.02E-131	-1.344528	0.260663	0.087	0.617	9.93E-127

PGF	6.54E-61	-1.340736	0.261653	0.079	0.35	2.153E-56
NTM	1.58E-155	-1.338519	0.262234	0.434	0.928	5.19E-151
NDNF	1.4E-131	-1.33755	0.262488	0.025	0.466	4.54E-127
HOXC8	1.62E-239	-1.335519	0.263022	0.021	0.691	5.34E-235
FHOD3	4.5E-128	-1.324492	0.265938	0.101	0.629	1.48E-123
HOXC6	1.91E-224	-1.32307	0.266316	0.037	0.71	6.28E-220
ADAMTSL1	1.1E-114	-1.322847	0.266376	0.087	0.578	3.48E-110
CERS6	3.90E-152	-1.31993	0.267154	0.17	0.803	1.28E-147
MALL	6.4E-118	-1.306108	0.270872	0.646	0.942	2.09E-113
CDCA4	2.21E-143	-1.296999	0.273351	0.474	0.926	7.28E-139
KCNQ5	1.17E-104	-1.294209	0.274115	0.049	0.418	3.84E-100
OSBPL6	1.01E-161	-1.293007	0.274444	0.099	0.722	3.34E-157
FGF5	5.03E-146	-1.290991	0.274998	0.681	0.958	1.66E-141
HS3ST3B1	7.86E-174	-1.289945	0.275286	0.395	0.937	2.59E-169
FAM167A	2.1E-106	-1.2886	0.275657	0.225	0.729	6.81E-102
HSD3B7	1.99E-133	-1.288202	0.275766	0.373	0.884	6.53E-129
HOXB3	2.15E-139	-1.279342	0.27822	0.146	0.733	7.06E-135
MYBL1	4.09E-122	-1.277558	0.278717	0.321	0.842	1.34E-117
NBL1	2.47E-212	-1.274176	0.279661	0.877	0.995	8.12E-208
LINC00595	5.86E-157	-1.272372	0.280166	0.076	0.617	1.93E-152
NFASC	6.36E-169	-1.269526	0.280965	0.102	0.638	2.09E-164
CLDN4	4.99E-51	-1.268007	0.281392	0.036	0.253	1.64E-46
ADIRF	1.47E-176	-1.267399	0.281563	0.421	0.937	4.84E-172
NPTX2	1.09E-137	-1.260775	0.283434	0.013	0.429	3.58E-133
FOXD1	9.88E-183	-1.25708	0.284484	0.829	0.995	3.25E-178
AC119674.2	6.8E-121	-1.250438	0.286379	0.15	0.687	2.24E-116
AUTS2	1.95E-143	-1.244808	0.287996	0.112	0.657	6.43E-139
SERTAD2	4.4E-157	-1.24157	0.28893	0.562	0.968	1.45E-152
GBP3	9.25E-114	-1.240915	0.289119	0.289	0.796	3.04E-109
NET1	8.1E-126	-1.240261	0.289309	0.581	0.937	2.66E-121
HOMER2	1.8E-153	-1.237057	0.290237	0.094	0.626	5.76E-149
LGMN	1.55E-137	-1.233147	0.291374	0.466	0.928	5.10E-133
TBX18	2.06E-141	-1.233044	0.291404	0.079	0.636	6.76E-137
HOXC9	1.66E-191	-1.227935	0.292897	0.047	0.677	5.47E-187
XG	1.25E-148	-1.225065	0.293739	0.02	0.494	4.12E-144
FBLN2	2.89E-128	-1.221188	0.29488	0.685	0.968	9.50E-124
CDCA5	8.00E-130	-1.219336	0.295426	0.318	0.879	2.63E-125
PRKY	5.86E-143	-1.216175	0.296362	0.06	0.568	1.93E-138
CPPED1	2.82E-155	-1.215641	0.29652	0.642	0.974	9.29E-151
HOXB7	2.19E-179	-1.213198	0.297245	0.15	0.833	7.21E-175

LYPD6B	5.24E-87	-1.211627	0.297713	0.032	0.381	1.73E-82
ZFY	2.97E-169	-1.21036	0.29809	0.013	0.515	9.79E-165
UBE2C	2.38E-130	-1.204195	0.299933	0.622	0.947	7.83E-126
TXLNGY	4.87E-175	-1.195743	0.302479	0.019	0.543	1.60E-170
GPC6	1.87E-102	-1.195266	0.302623	0.143	0.643	6.16E-98
GPAT2	3.04E-71	-1.191574	0.303743	0.028	0.318	1.00E-66
MEOX2	2.95E-149	-1.187349	0.305029	0.01	0.45	9.72E-145
LOXL3	9.23E-81	-1.184601	0.305868	0.273	0.694	3.04E-76
LINC02381	5.87E-153	-1.181007	0.306969	0.027	0.517	1.93E-148
RGMB	3.62E-163	-1.180706	0.307062	0.902	1	1.19E-158
AMPH	4.36E-137	-1.177534	0.308037	0.027	0.487	1.44E-132
EPB41L3	1.73E-141	-1.175503	0.308664	0.011	0.441	5.68E-137
PKMYT1	5.62E-113	-1.174712	0.308908	0.247	0.789	1.85E-108
ZNF536	2.8E-158	-1.173728	0.309212	0.014	0.487	9.06E-154
CTSL	3.31E-204	-1.167587	0.311117	0.997	1	1.09E-199
SHOX2	2.10E-176	-1.166788	0.311366	0.017	0.552	6.92E-172
TUBB3	1.20E-164	-1.166094	0.311582	0.936	0.995	3.95E-160
RRM2	8.24E-117	-1.164686	0.312021	0.754	0.974	2.71E-112
FBXO5	2.88E-118	-1.157578	0.314246	0.5	0.919	9.46E-114
SCARA3	8.4E-112	-1.157381	0.314308	0.504	0.937	2.75E-107
HOXB5	4.04E-161	-1.148307	0.317173	0.037	0.587	1.33E-156
TGFB1	1.36E-198	-1.147512	0.317426	0.934	1	4.47E-194
SQOR	7.8E-111	-1.137264	0.320695	0.382	0.879	2.57E-106
LINC00702	1.16E-110	-1.136041	0.321088	0.133	0.613	3.82E-106
CYB561	5.54E-110	-1.133344	0.321955	0.348	0.854	1.82E-105
CXCL12	7.79E-125	-1.132155	0.322338	0.907	0.993	2.56E-120
TBX15	1.48E-154	-1.127772	0.323754	0.017	0.497	4.87E-150
PSG6	1.33E-108	-1.12767	0.323787	0.078	0.531	4.39E-104
BMPER	1.03E-112	-1.125402	0.324522	0.137	0.664	3.38E-108
C1orf21	1.43E-113	-1.125278	0.324562	0.339	0.856	4.71E-109
KRT7	2.49E-41	-1.123681	0.325081	0.515	0.74	8.18E-37
CENPQ	5.12E-112	-1.12296	0.325315	0.247	0.787	1.69E-107
CTSZ	9.80E-191	-1.122641	0.325419	0.951	1	3.22E-186
TRNP1	2.54E-113	-1.121974	0.325636	0.768	0.968	8.36E-109
EN1	3.34E-165	-1.121055	0.325936	0.015	0.513	1.10E-160
MEGF6	2.24E-125	-1.120175	0.326223	0.028	0.466	7.36E-121
MOK	8.01E-46	-1.119578	0.326418	0.37	0.638	2.64E-41
TCF19	5.38E-110	-1.111202	0.329163	0.403	0.889	1.77E-105
CDA	1.56E-86	-1.109677	0.329665	0.112	0.534	5.14E-82
MOXD1	3.36E-108	-1.106448	0.330732	0.488	0.898	1.11E-103

KIAA1549L	3.6E-105	-1.103852	0.331591	0.298	0.814	1.2E-100
LOXL1	1.11E-167	-1.103221	0.331801	0.957	1	3.65E-163
F3	8.53E-66	-1.101704	0.332304	0.813	0.954	2.81E-61
DCN	1.73E-112	-1.097899	0.333571	0.949	0.998	5.70E-108
RMI2	7.03E-106	-1.097433	0.333727	0.222	0.752	2.31E-101
LIN7A	4.32E-82	-1.094804	0.334605	0.162	0.603	1.42E-77
ORC6	2.5E-128	-1.089559	0.336365	0.653	0.94	8.18E-124
MANCR	9.89E-81	-1.087702	0.33699	0.068	0.427	3.25E-76
FABP5	2.98E-127	-1.086676	0.337336	0.441	0.907	9.79E-123
ID1	7.33E-131	-1.08578	0.337638	0.987	0.995	2.41E-126
DPT	3.68E-120	-1.084146	0.338191	0.015	0.408	1.21E-115
GALNT5	1.49E-92	-1.08101	0.339253	0.301	0.789	4.91E-88
ESM1	8.54E-74	-1.080732	0.339347	0.026	0.318	2.81E-69
BGN	7.43E-104	-1.079764	0.339676	0.697	0.97	2.45E-99
MLPH	8.87E-104	-1.074984	0.341303	0.515	0.916	2.92E-99
VEPH1	2.40E-102	-1.074319	0.34153	0.429	0.877	7.89E-98
MT2A	3.95E-166	-1.07219	0.342258	1	1	1.30E-161
CXCL3	1.53E-52	-1.071956	0.342338	0.047	0.299	5.04E-48
VIT	2.26E-117	-1.071154	0.342613	0.014	0.401	7.45E-113
AC092143.1	4.26E-169	-1.07038	0.342878	0.777	0.991	1.40E-164
AC006058.1	4.93E-111	-1.070255	0.342921	0.016	0.381	1.62E-106
RFLNA	9.01E-130	-1.069987	0.343013	0.012	0.413	2.96E-125
EMP2	8.30E-142	-1.063755	0.345157	0.742	0.986	2.73E-137
LAPTM5	5.23E-74	-1.06362	0.345204	0.014	0.274	1.72E-69
PLAUR	1.16E-148	-1.059087	0.346772	0.97	1	3.81E-144
BACE2	2.05E-114	-1.057269	0.347403	0.388	0.889	6.76E-110
HOXB2	1.04E-108	-1.055502	0.348018	0.45	0.896	3.41E-104
LINC00517	2.16E-89	-1.054986	0.348197	0.086	0.515	7.12E-85
C3orf14	5.07E-110	-1.051518	0.349407	0.356	0.863	1.67E-105
ZNF385D	6.19E-99	-1.051421	0.349441	0.337	0.691	2.04E-94
CKAP2L	4.60E-106	-1.045664	0.351458	0.453	0.9	1.51E-101
SLC43A3	1.63E-87	-1.044514	0.351863	0.298	0.787	5.36E-83
TNFRSF21	2.65E-71	-1.041571	0.3529	0.246	0.664	8.73E-67
AURKB	3.54E-109	-1.040359	0.353328	0.387	0.882	1.16E-104
ENC1	2.94E-93	-1.039981	0.353461	0.453	0.889	9.68E-89
SHISAL1	4.38E-110	-1.034618	0.355362	0.013	0.367	1.44E-105
GADD45B	2.44E-122	-1.030884	0.356692	0.824	0.984	8.02E-118
PSMC3IP	1.1E-104	-1.030197	0.356937	0.302	0.831	3.73E-100
LRATD1	7.34E-93	-1.029841	0.357064	0.041	0.425	2.41E-88
LSAMP	1.05E-73	-1.028818	0.357429	0.126	0.536	3.45E-69

EPHB1	9.15E-86	-1.028188	0.357655	0.099	0.515	3.01E-81
CBR3	6.22E-107	-1.024199	0.359084	0.607	0.949	2.05E-102
EMILIN2	3.44E-86	-1.022123	0.35983	0.461	0.854	1.13E-81
ATP10A	4.71E-96	-1.021283	0.360133	0.23	0.722	1.55E-91
FXYD5	5.26E-205	-1.021261	0.360141	0.996	1	1.73E-200
KRT34	3.43E-23	-1.010831	0.363916	0.216	0.415	1.13E-18
HOXC11	7.95E-129	-1.009555	0.364381	0.023	0.455	2.62E-124
SKA1	2.60E-100	-1.006705	0.365421	0.42	0.884	8.56E-96
AL928654.3	1.67E-135	-1.006301	0.365569	0.231	0.768	5.49E-131
DIRAS1	1.05E-109	-1.004559	0.366206	0.046	0.485	3.46E-105
KCNK2	5.09E-114	-1.003755	0.366501	0.618	0.972	1.68E-109
CCDC152	9.56E-85	-1.000093	0.367845	0.109	0.517	3.14E-80
SCG5	6.57E-71	-0.997348	0.368856	0.186	0.606	2.16E-66
DNAJC9	8.79E-121	-0.99633	0.369232	0.785	0.956	2.89E-116
CRLF1	9.28E-60	-0.995471	0.369549	0.026	0.274	3.05E-55
ENPP1	2.67E-67	-0.992846	0.370521	0.368	0.766	8.78E-63
AEBP1	2.28E-131	-0.992826	0.370528	0.915	0.998	7.49E-127
XYLT1	7.87E-87	-0.992433	0.370674	0.334	0.805	2.59E-82
DPP4	1.48E-70	-0.98809	0.372287	0.093	0.469	4.86E-66
SBSN	2.42E-109	-0.986508	0.372877	0.011	0.36	7.98E-105
CDK1	7.68E-109	-0.98148	0.374756	0.515	0.914	2.53E-104
DSN1	1.23E-97	-0.97742	0.376281	0.407	0.877	4.04E-93
HJURP	4.87E-101	-0.976111	0.376773	0.332	0.856	1.60E-96
NR1D1	4.75E-103	-0.974785	0.377273	0.394	0.896	1.56E-98
OPCML	2.85E-96	-0.973812	0.377641	0.012	0.332	9.38E-92
IL33	3.06E-14	-0.969981	0.37909	0.023	0.107	1.01E-09
HTRA3	1.12E-81	-0.96884	0.379523	0.115	0.545	3.70E-77
PMP22	9.34E-91	-0.96748	0.38004	0.694	0.944	3.07E-86
P4HA3	1.96E-76	-0.967192	0.380149	0.115	0.515	6.45E-72
SELENOP	6.43E-68	-0.966532	0.3804	0.073	0.425	2.12E-63
CDCA7L	3.55E-93	-0.964494	0.381176	0.331	0.826	1.168E-88
RAD51	8.94E-82	-0.963105	0.381706	0.267	0.745	2.94E-77
EVA1A	1.12E-108	-0.960727	0.382615	0.716	0.972	3.69E-104
CD59	0.00E+00	-0.956361	0.384289	1	1	0.00E+00
CDC45	5.25E-84	-0.955489	0.384624	0.287	0.777	1.73E-79
ACAA2	5.8E-114	-0.955324	0.384688	0.686	0.972	1.92E-109
RTEL1-TNFR	1.24E-68	-0.954881	0.384858	0.199	0.559	4.09E-64
DCK	8.75E-99	-0.954757	0.384906	0.722	0.961	2.88E-94
MMP12	1.48E-43	-0.954423	0.385034	0.037	0.251	4.86E-39
MCUB	5.32E-97	-0.952651	0.385717	0.483	0.916	1.75E-92

CAPG	1.96E-99	-0.950616	0.386503	0.768	0.974	6.46E-95
VIM	0.00E+00	-0.950302	0.386624	1	1	0.00E+00
ASF1B	2.77E-92	-0.949746	0.386839	0.362	0.852	9.10E-88
RASD2	5.70E-82	-0.941664	0.389978	0.008	0.271	1.88E-77
STEAP1B	1.65E-70	-0.940673	0.390365	0.128	0.531	5.43E-66
FEN1	2.00E-90	-0.938027	0.391399	0.424	0.884	6.59E-86
SDF2L1	3.63E-113	-0.933976	0.392988	0.825	0.986	1.19E-108
E2F7	3.13E-82	-0.932224	0.393677	0.449	0.868	1.03E-77
CRNDE	3.14E-111	-0.930405	0.394394	0.22	0.766	1.03E-106
CHST11	1.80E-78	-0.92745	0.395561	0.082	0.476	5.91E-74
HOXB-AS3	1.26E-120	-0.925679	0.396262	0.052	0.517	4.16E-116
RRAS2	4.39E-105	-0.922964	0.397339	0.927	0.998	1.44E-100
CDCA8	1.09E-99	-0.922012	0.397718	0.353	0.868	3.58E-95
PITPNC1	7.07E-66	-0.92196	0.397739	0.209	0.617	2.33E-61
CD109	1.41E-107	-0.921461	0.397937	0.893	0.998	4.63E-103
COL5A3	4.62E-129	-0.920682	0.398247	0.048	0.538	1.52E-124
NLGN4Y	3.32E-111	-0.919668	0.398651	0.008	0.353	1.09E-106
S100A10	6.66E-156	-0.918737	0.399023	0.995	1	2.19E-151
COL15A1	6.56E-69	-0.915405	0.400354	0.024	0.295	2.16E-64
EDNRA	1.18E-50	-0.914602	0.400676	0.105	0.422	3.90E-46
UBASH3B	6.13E-77	-0.91393	0.400946	0.528	0.861	2.02E-72
UBL3	3.97E-139	-0.911389	0.401966	0.99	1	1.31E-134
KIAA1217	7.26E-94	-0.908693	0.403051	0.022	0.357	2.39E-89
MARCHF4	3.48E-81	-0.907138	0.403678	0.534	0.903	1.14E-76
KIFC1	1.24E-97	-0.905151	0.404481	0.456	0.91	4.07E-93
IL17RA	2.56E-81	-0.900759	0.406261	0.429	0.845	8.42E-77
KLF13	9.07E-77	-0.899499	0.406773	0.306	0.749	2.99E-72
KLF10	4.33E-83	-0.898886	0.407023	0.85	0.984	1.42E-78
NTF3	2.69E-93	-0.898411	0.407216	0.027	0.387	8.86E-89
EHD4	4.73E-86	-0.898261	0.407278	0.623	0.935	1.56E-81
TM4SF1	2.01E-19	-0.898093	0.407346	0.529	0.654	6.60E-15
EXO1	5.50E-76	-0.898055	0.407361	0.161	0.606	1.81E-71
BNC1	7.91E-70	-0.897716	0.4075	0.205	0.64	2.60E-65
MTFR2	4.25E-82	-0.895711	0.408317	0.182	0.654	1.40E-77
MT1E	5.07E-112	-0.895538	0.408388	0.858	0.991	1.67E-107
P4HA2	1.31E-129	-0.893881	0.409065	0.975	1	4.32E-125
ADTRP	4.06E-80	-0.889316	0.410937	0.03	0.353	1.34E-75
ENPP4	1.83E-110	-0.888606	0.411229	0.004	0.327	6.01E-106
IRX5	1.45E-137	-0.886869	0.411943	0.016	0.457	4.76E-133
C12orf56	5.69E-116	-0.886534	0.412082	0.008	0.367	1.87E-111

STMN2	2.46E-27	-0.885999	0.412302	0.098	0.313	8.10E-23
KCNC4	2.12E-71	-0.885688	0.41243	0.126	0.503	6.97E-67
RAD54L	1.44E-64	-0.884078	0.413095	0.148	0.545	4.75E-60
SYNJ2	1.30E-93	-0.881867	0.414009	0.847	0.981	4.27E-89
ZNF367	2.93E-76	-0.881626	0.414109	0.144	0.582	9.65E-72
HEPH	6.18E-112	-0.880378	0.414626	0.016	0.394	2.03E-107
CDT1	2.68E-78	-0.879714	0.414901	0.292	0.759	8.82E-74
DCLRE1B	2.40E-93	-0.879009	0.415194	0.192	0.701	7.89E-89
AK4	4.45E-61	-0.878002	0.415612	0.052	0.353	1.46E-56
SRPX	1.41E-78	-0.877895	0.415657	0.548	0.914	4.65E-74
TRIP13	2.74E-84	-0.877779	0.415705	0.553	0.914	9.02E-80
PDIA5	6.25E-82	-0.877696	0.41574	0.559	0.896	2.06E-77
SLC7A8	4.89E-50	-0.877695	0.41574	0.06	0.325	1.61E-45
SCFD2	1.89E-84	-0.87701	0.416025	0.534	0.919	6.23E-80
MCM4	2.65E-87	-0.875749	0.41655	0.772	0.963	8.72E-83
PLIN2	8.59E-108	-0.874942	0.416886	0.945	1	2.83E-103
E2F1	4.37E-69	-0.874674	0.416998	0.211	0.645	1.44E-64
FLRT2	4.99E-75	-0.874368	0.417126	0.601	0.91	1.64E-70
RCN3	9.59E-141	-0.869576	0.419129	0.995	1	3.16E-136
KLF6	4.08E-112	-0.866829	0.420282	0.998	1	1.34E-107
CENPU	1.02E-87	-0.866069	0.420602	0.505	0.914	3.36E-83
PRIM1	2.00E-69	-0.865739	0.420741	0.262	0.705	6.57E-65
SKA3	2.16E-93	-0.865674	0.420768	0.462	0.91	7.10E-89
KDM5D	3.29E-107	-0.862995	0.421897	0.013	0.36	1.08E-102
DUSP2	4.42E-26	-0.862079	0.422283	0.066	0.237	1.45E-21
TGFBR2	5.91E-90	-0.852646	0.426286	0.751	0.97	1.94E-85
ANGPTL4	2.11E-51	-0.852571	0.426317	0.065	0.343	6.95E-47
RAD51AP1	8.31E-89	-0.852502	0.426347	0.367	0.861	2.74E-84
PRSS23	4.70E-128	-0.851018	0.42698	1	1	1.55E-123
PTER	1.43E-78	-0.850551	0.427179	0.117	0.545	4.71E-74
TLN2	1.25E-74	-0.848221	0.428176	0.482	0.872	4.12E-70
EBF3	1.49E-89	-0.848007	0.428268	0.008	0.295	4.91E-85
RASA1	2.48E-67	-0.846207	0.429039	0.914	0.979	8.15E-63
SLC35E4	5.89E-72	-0.843508	0.430199	0.227	0.661	1.94E-67
JAM3	9.87E-86	-0.842738	0.43053	0.635	0.942	3.25E-81
CENPO	5.24E-81	-0.841047	0.431259	0.35	0.821	1.72E-76
TMEM47	2.18E-76	-0.840987	0.431285	0.904	0.984	7.18E-72
DUSP23	1.91E-85	-0.840284	0.431588	0.182	0.661	6.27E-81
PTPN22	8.12E-57	-0.839605	0.431881	0.037	0.299	2.67E-52
SH3BP5L	1.24E-66	-0.838143	0.432513	0.497	0.845	4.08E-62

SERPINB7	6.35E-46	-0.837437	0.432818	0.877	0.947	2.09E-41
ARHGAP29	1.70E-61	-0.835391	0.433705	0.85	0.951	5.60E-57
CDCA2	6.52E-92	-0.834504	0.43409	0.355	0.858	2.14E-87
BCAR3	1.06E-74	-0.832349	0.435026	0.693	0.93	3.50E-70
MOCOS	5.08E-65	-0.831958	0.435197	0.096	0.406	1.67E-60
GPR68	2.73E-81	-0.83172	0.4353	0.01	0.288	8.97E-77
RPP25	5.39E-64	-0.830109	0.436002	0.05	0.362	1.77E-59
TNFRSF10A	2.96E-71	-0.829452	0.436288	0.132	0.531	9.75E-67
SPATC1L	6.40E-83	-0.829312	0.436349	0.159	0.626	2.11E-78
STAC	3.62E-97	-0.826469	0.437592	0.021	0.376	1.19E-92
PAX3	1.17E-86	-0.820387	0.440261	0.017	0.323	3.84E-82
INHBB	9.64E-64	-0.819908	0.440472	0.004	0.206	3.17E-59
THBD	5.09E-52	-0.815906	0.442239	0.009	0.19	1.67E-47
GEM	2.47E-45	-0.815379	0.442471	0.296	0.643	8.11E-41
PLAC9	7.97E-151	-0.811355	0.444256	0.02	0.503	2.62E-146
C6orf99	3.40E-78	-0.810497	0.444637	0.025	0.334	1.12E-73
GSTT2	1.36E-99	-0.809658	0.44501	0.142	0.647	4.49E-95
CLSPN	6.77E-68	-0.809474	0.445092	0.462	0.852	2.23E-63
SERPINE2	2.29E-66	-0.80825	0.445637	0.995	1	7.53E-62
TTK	8.87E-92	-0.807399	0.446017	0.38	0.875	2.92E-87
VRK1	5.93E-74	-0.806408	0.446459	0.456	0.863	1.95E-69
EPAS1	1.28E-74	-0.805593	0.446823	0.822	0.979	4.20E-70
SLC16A2	2.05E-64	-0.804713	0.447216	0.573	0.903	6.74E-60
ESCO2	1.60E-67	-0.80468	0.447231	0.522	0.835	5.27E-63
SLC17A9	4.33E-67	-0.804478	0.447321	0.48	0.849	1.43E-62
FAM20C	2.25E-60	-0.802649	0.44814	0.661	0.907	7.39E-56
KRBOX1	7.99E-61	-0.798996	0.44978	0.076	0.408	2.63E-56
LYPD6	5.58E-46	-0.797189	0.450594	0.176	0.508	1.84E-41
OLFM1	1.07E-66	-0.797181	0.450598	0.045	0.357	3.52E-62
SLIT2	6.20E-83	-0.796208	0.451036	0.669	0.972	2.04E-78
LINC01204	1.32E-54	-0.793953	0.452054	0.11	0.387	4.34E-50
CPEB1	4.15E-62	-0.793035	0.452469	0.382	0.724	1.37E-57
SLC35F2	2.94E-72	-0.792245	0.452827	0.151	0.573	9.67E-68
LFNG	5.44E-77	-0.790498	0.453619	0.078	0.464	1.79E-72
MT1M	1.37E-102	-0.79001	0.45384	0.068	0.52	4.51E-98
TENM4	5.93E-65	-0.789953	0.453866	0.165	0.573	1.95E-60
GTSE1	4.35E-85	-0.787092	0.455166	0.474	0.916	1.43E-80
ARMH4	7.26E-65	-0.786798	0.4553	0.275	0.701	2.39E-60
AC207056.1	3.26E-22	-0.786693	0.455348	0.172	0.362	1.07E-17
PARVB	4.86E-72	-0.785884	0.455717	0.61	0.923	1.60E-67

BCL7A	1.92E-52	-0.785097	0.456075	0.181	0.503	6.32E-48
FAM3C	6.53E-88	-0.782637	0.457199	0.864	0.981	2.15E-83
TMEM54	4.89E-63	-0.782347	0.457331	0.248	0.671	1.61E-58
MTURN	1.98E-59	-0.781921	0.457526	0.289	0.698	6.50E-55
FZD1	1.66E-61	-0.780584	0.458139	0.319	0.735	5.47E-57
PLEK2	1.57E-54	-0.779759	0.458516	0.379	0.763	5.15E-50
MELK	5.78E-81	-0.779565	0.458605	0.648	0.947	1.90E-76
CENPT	2.43E-76	-0.779284	0.458735	0.648	0.935	8.00E-72
HSPB2	5.54E-80	-0.779173	0.458785	0.114	0.545	1.82E-75
CDC25B	2.20E-72	-0.778928	0.458898	0.649	0.958	7.24E-68
BOC	1.61E-66	-0.778079	0.459288	0.244	0.675	5.30E-62
USP1	2.12E-82	-0.777237	0.459674	0.809	0.979	6.97E-78
EVL	6.54E-57	-0.775898	0.46029	0.196	0.575	2.15E-52
CRELD2	1.23E-83	-0.77561	0.460423	0.811	0.979	4.05E-79
TEX30	4.36E-70	-0.775452	0.460495	0.591	0.912	1.43E-65
HIPK2	7.03E-57	-0.775337	0.460549	0.454	0.807	2.31E-52
LASTR	2.29E-56	-0.774231	0.461058	0.069	0.374	7.53E-52
G0S2	4.82E-05	-0.773412	0.461436	0.168	0.155	1.00E+00
GLIPR2	3.66E-70	-0.77328	0.461497	0.645	0.949	1.20E-65
NCAPG	7.63E-83	-0.773226	0.461522	0.544	0.916	2.51E-78
ZWILCH	3.44E-78	-0.772557	0.461831	0.721	0.958	1.13E-73
PAQR4	6.88E-54	-0.772053	0.462063	0.134	0.487	2.27E-49
GCNT1	5.73E-58	-0.771936	0.462117	0.412	0.791	1.88E-53
STK26	2.67E-59	-0.77185	0.462157	0.086	0.425	8.79E-55
CDC6	5.88E-55	-0.771249	0.462435	0.236	0.631	1.94E-50
TOX2	6.86E-64	-0.766232	0.464761	0.249	0.677	2.26E-59
GPNMB	3.56E-76	-0.766009	0.464865	0.572	0.937	1.17E-71
GALNT15	1.23E-25	-0.765606	0.465052	0.398	0.613	4.04E-21
POLD3	3.22E-60	-0.765504	0.465099	0.373	0.777	1.058E-55
S1PR1	7.38E-42	-0.765376	0.465159	0.627	0.833	2.43E-37
RORA	5.39E-59	-0.76507	0.465301	0.076	0.397	1.77E-54
FYN	2.50E-56	-0.76353	0.466019	0.404	0.782	8.24E-52
PTGS2	3.49E-20	-0.761193	0.467109	0.013	0.107	1.15E-15
CAMKK1	1.51E-32	-0.761094	0.467155	0.222	0.492	4.96E-28
SNHG7	3.7E-70	-0.756986	0.469078	0.395	0.833	1.217E-65
SLC8A1-AS1	1.39E-40	-0.755902	0.469587	0.076	0.32	4.56E-36
ITPR3	1.24E-52	-0.755145	0.469942	0.372	0.756	4.08E-48
GRAMD1B	2.11E-74	-0.754934	0.470042	0.11	0.517	6.95E-70
KCTD15	7.54E-58	-0.75406	0.470453	0.18	0.568	2.48E-53
MANF	1.58E-87	-0.75391	0.470523	0.992	0.998	5.19E-83

DDIAS	7.47E-76	-0.753708	0.470618	0.221	0.687	2.46E-71
SPESP1	8.87E-80	-0.753166	0.470874	0.019	0.32	2.92E-75
CDC7	1.10E-80	-0.751705	0.471562	0.163	0.624	3.62E-76
C12orf75	2.28E-178	-0.750746	0.472014	1	1	7.50E-174
RFC4	4.95E-69	-0.750391	0.472182	0.392	0.831	1.63E-64
PBX3	4.01E-59	-0.749805	0.472458	0.678	0.91	1.32E-54
PRDM8	2.00E-51	-0.749354	0.472672	0.195	0.534	6.59E-47
DTL	4.86E-74	-0.748606	0.473026	0.227	0.689	1.60E-69
TIMP1	5.63E-107	-0.743838	0.475286	1	1	1.85E-102
GSTT2B	7.50E-82	-0.739117	0.477536	0.138	0.585	2.47E-77
RPL9	0.00E+00	-0.737119	0.478491	1	1	0.00E+00
ABHD5	9.52E-57	-0.73609	0.478983	0.626	0.905	3.13E-52
ARL4A	9.29E-52	-0.734959	0.479525	0.508	0.84	3.06E-47
HYLS1	2.77E-56	-0.734169	0.479904	0.351	0.747	9.11E-52
STIL	2.02E-67	-0.733483	0.480234	0.35	0.789	6.65E-63
SPRY2	3.42E-54	-0.733135	0.4804	0.579	0.882	1.12E-49
HAUS6	2.59E-62	-0.732394	0.480757	0.568	0.891	8.53E-58
SPRY4	1.23E-66	-0.726658	0.483522	0.462	0.861	4.06E-62
MASTL	5.93E-68	-0.72647	0.483613	0.442	0.858	1.95E-63
AKR1C3	3.32E-44	-0.725364	0.484148	0.037	0.265	1.09E-39
KCNN4	2.24E-63	-0.725084	0.484284	0.017	0.265	7.36E-59
FST	5.37E-22	-0.72445	0.484591	0.753	0.872	1.77E-17
CCNA2	3.23E-78	-0.723971	0.484823	0.629	0.944	1.06E-73
EML1	4.11E-52	-0.723515	0.485044	0.46	0.8	1.35E-47
RFC3	1.14E-76	-0.722733	0.485424	0.331	0.807	3.76E-72
PBK	6.53E-73	-0.722709	0.485435	0.565	0.919	2.15E-68
RFC2	1.33E-59	-0.720812	0.486357	0.607	0.9	4.39E-55
GAL	6.46E-48	-0.719733	0.486882	0.013	0.195	2.13E-43
EGR3	1.47E-18	-0.718711	0.48738	0.094	0.244	4.83E-14
APCDD1L	2.18E-66	-0.71833	0.487566	0.801	0.977	7.18E-62
KIF11	1.67E-76	-0.713446	0.489953	0.501	0.9	5.48E-72
TPX2	5.50E-75	-0.712814	0.490263	0.785	0.972	1.81E-70
ESPL1	4.91E-57	-0.712677	0.49033	0.191	0.582	1.61E-52
POLA2	1.45E-62	-0.711997	0.490663	0.374	0.798	4.77E-58
NPC1	3.40E-66	-0.711211	0.491049	0.451	0.858	1.12E-61
NMRK1	4.67E-60	-0.710914	0.491195	0.287	0.708	1.54E-55
SHCBP1	6.76E-68	-0.710873	0.491215	0.664	0.935	2.22E-63
CCNF	4.31E-67	-0.710682	0.491309	0.304	0.752	1.42E-62
SMAGP	7.86E-62	-0.710126	0.491582	0.561	0.898	2.59E-57
HM13	2.53E-106	-0.710056	0.491617	0.997	1	8.33E-102

MYBL2	6.55E-59	-0.709624	0.491829	0.432	0.828	2.16E-54
FEZ1	8.33E-54	-0.708855	0.492207	0.38	0.749	2.74E-49
CHAF1A	1.71E-67	-0.708278	0.492491	0.351	0.794	5.61E-63
PLK4	3.55E-80	-0.70792	0.492668	0.267	0.754	1.17E-75
THRB	5.36E-32	-0.707905	0.492675	0.047	0.239	1.76E-27
POLM	1.14E-36	-0.707452	0.492899	0.252	0.552	3.76E-32
MYPN	5.00E-47	-0.705311	0.493955	0.189	0.538	1.64E-42
DNAJB4	4.13E-39	-0.704701	0.494257	0.739	0.896	1.36E-34
MIR222HG	3.73E-47	-0.704363	0.494424	0.097	0.399	1.23E-42
TSPO	6.88E-120	-0.704237	0.494486	0.998	1	2.26E-115
MAPKAPK3	1.09E-42	-0.704087	0.49456	0.385	0.726	3.60E-38
STMN3	2.03E-43	-0.703899	0.494653	0.382	0.719	6.67E-39
STX1A	3.25E-56	-0.70321	0.494994	0.158	0.527	1.07E-51
RECQL4	4.34E-59	-0.702502	0.495344	0.229	0.638	1.43E-54
C18orf54	1.20E-58	-0.700754	0.496211	0.264	0.675	3.95E-54
ZNF395	2.85E-48	-0.700424	0.496375	0.348	0.724	9.38E-44
ARHGAP11A	6.84E-72	-0.699511	0.496828	0.553	0.919	2.25E-67
IRAK3	3.89E-68	-0.698063	0.497548	0.023	0.302	1.28E-63
GPER1	4.30E-60	-0.697965	0.497597	0.033	0.304	1.41E-55
C19orf48	5.97E-67	-0.696545	0.498304	0.493	0.893	1.96E-62
PLK2	1.58E-31	-0.696022	0.498564	0.829	0.903	5.21E-27
LRFN4	3.31E-53	-0.695941	0.498605	0.318	0.696	1.09E-48
PRELP	3.27E-25	-0.694488	0.49933	0.125	0.306	1.08E-20
RBL1	8.60E-54	-0.693994	0.499577	0.338	0.722	2.83E-49
OLFML3	9.44E-74	-0.693924	0.499612	0.949	1	3.10E-69
MIS18A	1.54E-73	-0.693421	0.499863	0.49	0.907	5.05E-69
PDE7B	4.49E-38	-0.693154	0.499996	0.089	0.329	1.48E-33
NDRG1	2.94E-43	-0.691986	0.500581	0.634	0.879	9.66E-39
BRCA1	5.09E-51	-0.689079	0.502038	0.529	0.849	1.67E-46
NCAPH	1.28E-83	-0.687868	0.502647	0.421	0.896	4.20E-79
GLUL	5.56E-72	-0.687231	0.502967	0.918	0.993	1.83E-67
GAS1	2.36E-41	-0.685234	0.503972	0.115	0.406	7.77E-37
GINS1	8.20E-73	-0.684742	0.504221	0.307	0.773	2.70E-68
GINS3	2.71E-47	-0.684436	0.504374	0.221	0.573	8.91E-43
GREM2	7.23E-39	-0.68439	0.504398	0.486	0.803	2.38E-34
MCM10	6.17E-53	-0.684266	0.50446	0.223	0.608	2.03E-48
CENPL	9.19E-56	-0.683751	0.50472	0.421	0.796	3.02E-51
ADCY7	2.45E-65	-0.683394	0.5049	0.417	0.838	8.05E-61
HAUS3	2.60E-57	-0.682321	0.505443	0.396	0.796	8.55E-53
TNFSF9	1.59E-89	-0.681193	0.506013	0.017	0.336	5.24E-85

SLC6A15	2.69E-57	-0.680969	0.506126	0.023	0.267	8.84E-53
ST8SIA2	6.06E-68	-0.678245	0.507507	0.006	0.225	1.99E-63
LUM	2.06E-43	-0.678225	0.507517	0.973	0.998	6.78E-39
GPAM	7.04E-43	-0.677813	0.507726	0.35	0.694	2.32E-38
CLU	7.50E-19	-0.676508	0.508389	0.615	0.777	2.47E-14
PPP2R3B	1.15E-53	-0.676414	0.508437	0.404	0.766	3.77E-49
PTGS1	1.09E-23	-0.675723	0.508789	0.328	0.575	3.57E-19
SEPTIN6	4.84E-60	-0.675713	0.508794	0.209	0.617	1.59E-55
NDC80	1.28E-64	-0.674784	0.509267	0.561	0.898	4.22E-60
FANCG	4.47E-55	-0.674217	0.509555	0.315	0.717	1.47E-50
ST3GAL4	1.35E-54	-0.672285	0.51054	0.399	0.787	4.44E-50
FRMD3	7.98E-30	-0.671612	0.510884	0.254	0.531	2.63E-25
ACVRL1	2.79E-54	-0.6701	0.511658	0.058	0.346	9.19E-50
VLDLR	5.23E-37	-0.66825	0.512605	0.226	0.524	1.72E-32
ADAM23	2.13E-52	-0.667986	0.51274	0.072	0.371	7.00E-48
MGLL	5.89E-71	-0.667872	0.512799	0.996	0.998	1.94E-66
SIRPA	1.10E-43	-0.667652	0.512911	0.425	0.749	3.61E-39
SNAI1	4.09E-23	-0.666813	0.513342	0.206	0.434	1.35E-18
NUF2	2.67E-81	-0.666606	0.513448	0.418	0.886	8.79E-77
CYSTM1	7.41E-54	-0.665411	0.514062	0.473	0.847	2.44E-49
SGO2	3.63E-77	-0.664863	0.514344	0.498	0.912	1.20E-72
HAUS8	3.88E-56	-0.664484	0.514539	0.299	0.708	1.28E-51
CCN3	2.47E-37	-0.663317	0.51514	0.057	0.285	8.12E-33
WDHD1	1.51E-56	-0.661184	0.51624	0.364	0.773	4.96E-52
LGR4	5.51E-34	-0.660936	0.516368	0.333	0.64	1.81E-29
SMTN	8.68E-58	-0.66078	0.516448	0.789	0.958	2.86E-53
C2orf69	6.68E-60	-0.66057	0.516557	0.476	0.858	2.20E-55
TRAIP	3.05E-48	-0.659533	0.517093	0.15	0.49	1.00E-43
MSX1	1.27E-47	-0.658848	0.517447	0.169	0.51	4.17E-43
CXCL2	1.00E-11	-0.658676	0.517536	0.128	0.265	3.30E-07
IL11	2.54E-25	-0.656736	0.518541	0.277	0.541	8.37E-21
IVNS1ABP	4.24E-50	-0.656651	0.518585	0.565	0.875	1.39E-45
SPC25	4.39E-77	-0.656126	0.518857	0.353	0.831	1.44E-72
CCIN	4.34E-48	-0.656025	0.51891	0.02	0.23	1.43E-43
POC1A	8.51E-61	-0.654862	0.519514	0.374	0.798	2.80E-56
ADAMTS2	5.13E-55	-0.653652	0.520143	0.708	0.942	1.69E-50
EDN1	5.84E-26	-0.65359	0.520175	0.192	0.443	1.92E-21
DOCK5	3.49E-51	-0.653553	0.520194	0.751	0.933	1.15E-46
ERCC6L	7.27E-64	-0.65302	0.520471	0.183	0.594	2.39E-59
PTGES	1.13E-33	-0.65269	0.520643	0.171	0.455	3.71E-29

RACGAP1	6.69E-71	-0.652583	0.520699	0.621	0.942	2.20E-66
ZWINT	2.70E-57	-0.652526	0.520729	0.693	0.933	8.88E-53
RTTN	1.38E-49	-0.65168	0.521169	0.28	0.647	4.55E-45
THSD1	5.02E-42	-0.651449	0.52129	0.088	0.362	1.65E-37
ID4	2.12E-43	-0.651176	0.521432	0.359	0.71	6.96E-39
SULF2	2.89E-61	-0.650832	0.521612	0.02	0.269	9.52E-57
BUB3	6.54E-75	-0.650767	0.521646	0.992	1	2.15E-70
SUV39H1	3.93E-57	-0.650642	0.521711	0.238	0.643	1.29E-52
KLF4	1.21E-28	-0.649325	0.522398	0.332	0.613	3.97E-24
FAM111B	6.46E-36	-0.64778	0.523206	0.198	0.501	2.13E-31
INCENP	2.28E-50	-0.647663	0.523267	0.222	0.596	7.51E-46
AURKA	3.07E-60	-0.647467	0.52337	0.518	0.884	1.01E-55
GAS6	2.52E-57	-0.645365	0.524471	0.924	0.991	8.28E-53
CERCAM	5.48E-67	-0.644968	0.524679	0.892	0.995	1.80E-62
NGEF	4.08E-68	-0.64393	0.525224	0.01	0.248	1.34E-63
HERC3	3.49E-52	-0.642685	0.525878	0.508	0.84	1.15E-47
TNFAIP6	9.00E-35	-0.642265	0.526099	0.22	0.527	2.96E-30
SAC3D1	2.32E-54	-0.64186	0.526313	0.379	0.773	7.63E-50
MMP14	2.61E-89	-0.640326	0.527121	0.993	1	8.60E-85
ACKR4	5.23E-44	-0.639786	0.527405	0.044	0.281	1.72E-39
MAN1A1	2.81E-48	-0.639464	0.527575	0.518	0.854	9.26E-44
PPIF	6.64E-44	-0.639447	0.527584	0.832	0.951	2.19E-39
ZNF672	2.29E-41	-0.639324	0.527649	0.314	0.661	7.53E-37
CDKN2C	7.15E-60	-0.639206	0.527711	0.561	0.905	2.35E-55
MAD2L1	8.39E-66	-0.638953	0.527845	0.679	0.944	2.76E-61
KIF2C	1.25E-74	-0.638685	0.527986	0.399	0.858	4.12E-70
UBE2T	6.28E-64	-0.638233	0.528225	0.666	0.937	2.07E-59
PCNA	2.51E-57	-0.636637	0.529069	0.948	1	8.25E-53
SPRYD3	3.42E-44	-0.635799	0.529512	0.435	0.77	1.13E-39
RXRA	6.90E-53	-0.635771	0.529527	0.622	0.912	2.27E-48
GAS7	3.03E-40	-0.63539	0.529729	0.202	0.506	9.96E-36
BRI3	1.80E-83	-0.635376	0.529736	0.951	0.991	5.91E-79
RFC5	4.43E-54	-0.634566	0.530166	0.382	0.777	1.46E-49
SMIM29	9.28E-53	-0.634467	0.530218	0.687	0.94	3.05E-48
CENPI	3.58E-48	-0.633121	0.530932	0.203	0.564	1.18E-43
ISLR	2.66E-71	-0.632124	0.531462	0.994	0.995	8.74E-67
ATAD3A	9.47E-65	-0.631813	0.531627	0.6	0.933	3.12E-60
TACC3	2.71E-61	-0.631783	0.531643	0.602	0.91	8.90E-57
RNASE4	1.26E-55	-0.631562	0.53176	0.28	0.682	4.14E-51
KIF18A	7.86E-62	-0.631378	0.531858	0.442	0.852	2.59E-57

PLXNA2	3.18E-48	-0.630874	0.532127	0.084	0.381	1.05E-43
TTLL7	3.24E-48	-0.63068	0.53223	0.496	0.831	1.07E-43
C1GALT1	1.30E-42	-0.630455	0.532349	0.581	0.861	4.29E-38
ING2	1.04E-51	-0.630293	0.532436	0.401	0.773	3.42E-47
TYMS	5.52E-54	-0.63005	0.532565	0.778	0.963	1.82E-49
FUOM	3.22E-60	-0.629603	0.532803	0.256	0.673	1.06E-55
MME	6.82E-33	-0.629297	0.532966	0.799	0.937	2.25E-28
FABP5P7	7.94E-84	-0.628686	0.533292	0.206	0.682	2.61E-79
NAGK	3.47E-64	-0.627983	0.533667	0.95	0.998	1.14E-59
SEC14L2	4.92E-40	-0.627776	0.533778	0.196	0.517	1.62E-35
LINC00511	5.68E-34	-0.626165	0.534638	0.175	0.408	1.87E-29
AL049629.2	1.22E-88	-0.625409	0.535042	0.807	0.986	4.02E-84
SAT1	1.41E-41	-0.625169	0.535171	0.894	0.981	4.63E-37
DUSP5	1.93E-46	-0.624845	0.535345	0.487	0.828	6.36E-42
MXD3	2.08E-41	-0.624715	0.535414	0.54	0.835	6.86E-37
POP7	1.54E-59	-0.624271	0.535652	0.764	0.965	5.07E-55
FAM47E-STE	3.54E-42	-0.624205	0.535687	0.234	0.575	1.17E-37
PTGIR	4.30E-42	-0.622776	0.536453	0.216	0.536	1.42E-37
TGFBR1	5.22E-49	-0.622409	0.53665	0.467	0.828	1.72E-44
PSG4	1.26E-45	-0.621988	0.536876	0.711	0.93	4.15E-41
AL139393.3	7.07E-23	-0.621801	0.536976	0.117	0.309	2.32E-18
SPDL1	2.36E-64	-0.62142	0.537181	0.85	0.981	7.75E-60
NXT1	1.26E-50	-0.621139	0.537332	0.559	0.886	4.13E-46
SCD5	1.89E-29	-0.619817	0.538043	0.249	0.529	6.20E-25
STING1	1.17E-44	-0.618741	0.538622	0.729	0.923	3.86E-40
C4orf46	6.59E-51	-0.618526	0.538738	0.43	0.805	2.17E-46
TMPO	1.94E-61	-0.618419	0.538796	0.86	0.981	6.38E-57
TNC	2.46E-40	-0.617457	0.539314	0.964	0.998	8.09E-36
CCNE2	2.48E-29	-0.616292	0.539943	0.104	0.334	8.15E-25
IFRD2	1.55E-62	-0.616004	0.540098	0.837	0.979	5.09E-58
FBXO4	1.81E-39	-0.615843	0.540185	0.33	0.666	5.96E-35
RGS3	1.74E-40	-0.615772	0.540224	0.728	0.921	5.72E-36
CMSS1	2.17E-49	-0.615374	0.540439	0.567	0.884	7.15E-45
CD274	8.20E-33	-0.615178	0.540545	0.288	0.594	2.70E-28
SDC4	5.38E-64	-0.614702	0.540802	0.924	0.998	1.77E-59
HMMR	1.60E-66	-0.61334	0.541539	0.495	0.891	5.26E-62
ZNF85	1.87E-36	-0.612381	0.542059	0.153	0.436	6.14E-32
FN1	8.87E-90	-0.611965	0.542284	1	1	2.92E-85
PLCB4	1.30E-37	-0.611161	0.542721	0.532	0.828	4.28E-33
KLHL21	3.54E-30	-0.61073	0.542954	0.507	0.754	1.16E-25

FKBP11	2.25E-66	-0.610718	0.542961	0.888	0.991	7.39E-62
SGTB	1.16E-46	-0.61005	0.543324	0.829	0.949	3.80E-42
GMNN	1.05E-53	-0.609977	0.543363	0.68	0.933	3.45E-49
EME1	5.79E-38	-0.608978	0.543907	0.134	0.42	1.90E-33
FZD8	1.34E-30	-0.608581	0.544122	0.184	0.455	4.41E-26
H2AX	3.80E-63	-0.608199	0.54433	0.824	0.963	1.25E-58
SDC1	2.84E-37	-0.607566	0.544675	0.689	0.896	9.33E-33
NETO2	2.92E-37	-0.607539	0.54469	0.568	0.842	9.62E-33
SDC2	2.38E-67	-0.607082	0.544939	0.96	1	7.84E-63
TUBB2A	2.59E-61	-0.606879	0.54505	0.966	0.991	8.52E-57
IL20RB	1.48E-31	-0.606807	0.545089	0.053	0.253	4.88E-27
ATAD2	4.44E-47	-0.606492	0.545261	0.507	0.847	1.46E-42
AC090204.1	4.92E-93	-0.606419	0.5453	0.008	0.311	1.62E-88
FAM111A	4.53E-44	-0.60448	0.546359	0.667	0.896	1.49E-39
CTSB	2.15E-129	-0.603548	0.546868	1	1	7.07E-125
PID1	8.93E-33	-0.603302	0.547003	0.152	0.425	2.94E-28
CKS2	4.76E-59	-0.601873	0.547785	0.884	0.977	1.57E-54
PRPF4	1.71E-55	-0.601434	0.548025	0.741	0.949	5.62E-51
LINC00960	5.33E-38	-0.601395	0.548047	0.271	0.599	1.75E-33
CDCA3	3.10E-48	-0.600987	0.54827	0.541	0.868	1.02E-43
ARHGAP22	2.34E-49	-0.600208	0.548697	0.681	0.933	7.68E-45
PRXL2C	2.01E-54	-0.600208	0.548697	0.595	0.907	6.61E-50
RECK	9.69E-50	-0.599638	0.54901	0.741	0.949	3.19E-45
KIF15	1.20E-64	-0.5988	0.54947	0.292	0.729	3.94E-60
LRR1	5.95E-53	-0.597368	0.550258	0.616	0.916	1.96E-48
RNF182	2.50E-39	-0.596848	0.550544	0.134	0.427	8.23E-35
HSPB7	2.02E-15	-0.596164	0.550921	0.282	0.48	6.65E-11
RNASEH2B	2.10E-54	-0.596033	0.550993	0.637	0.93	6.92E-50
KIF22	3.73E-58	-0.594822	0.55166	0.634	0.923	1.23E-53
PLXNC1	2.76E-44	-0.592881	0.552732	0.018	0.211	9.07E-40
NIPAL3	2.40E-44	-0.59259	0.552893	0.924	0.97	7.90E-40
RAB31	8.89E-54	-0.59243	0.552982	0.998	1	2.92E-49
TAGLN2	8.81E-115	-0.592365	0.553018	1	1	2.90E-110
SIM1	5.76E-69	-0.591646	0.553416	0.009	0.244	1.89E-64
GINS4	1.08E-57	-0.591023	0.55376	0.485	0.87	3.54E-53
HPCAL1	4.92E-44	-0.590587	0.554002	0.731	0.919	1.62E-39
FICD	7.47E-28	-0.590582	0.554005	0.268	0.538	2.46E-23
TEDC1	1.45E-48	-0.590483	0.554059	0.2	0.561	4.76E-44
FAM72B	6.65E-65	-0.59046	0.554072	0.209	0.636	2.19E-60
CENPK	1.25E-54	-0.589786	0.554446	0.576	0.896	4.11E-50

SH2D4A	5.12E-32	-0.589353	0.554686	0.547	0.805	1.68E-27
USP12	1.00E-46	-0.588883	0.554947	0.566	0.882	3.31E-42
PRLR	5.33E-36	-0.588383	0.555225	0.039	0.23	1.75E-31
SFR1	2.66E-51	-0.588288	0.555277	0.204	0.578	8.77E-47
RMI1	5.40E-44	-0.588105	0.555379	0.428	0.782	1.78E-39
SLC29A1	2.30E-33	-0.587742	0.55558	0.369	0.677	7.58E-29
POLE3	1.25E-49	-0.586991	0.555998	0.763	0.949	4.10E-45
CCDC77	1.08E-40	-0.586694	0.556163	0.254	0.594	3.56E-36
WDR76	8.86E-35	-0.58562	0.556761	0.342	0.659	2.91E-30
GALNT14	8.36E-47	-0.58529	0.556944	0.009	0.179	2.75E-42
CSPG4	2.23E-51	-0.58488	0.557173	0.575	0.896	7.35E-47
DBF4	5.11E-48	-0.584724	0.55726	0.52	0.852	1.68E-43
FAM83D	9.21E-57	-0.584524	0.557371	0.365	0.777	3.03E-52
GFRA1	9.48E-46	-0.584421	0.557429	0.3	0.668	3.12E-41
NCAPH2	8.78E-50	-0.584213	0.557545	0.613	0.907	2.89E-45
KLF11	6.92E-39	-0.58357	0.557903	0.13	0.42	2.28E-34
GTF2H2	1.54E-61	-0.583085	0.558174	0.87	0.974	5.08E-57
CIP2A	3.31E-57	-0.58305	0.558193	0.561	0.91	1.09E-52
BUB1B	8.67E-70	-0.582729	0.558372	0.363	0.819	2.85E-65
INSR	1.11E-33	-0.582453	0.558527	0.158	0.434	3.66E-29
MRTO4	9.76E-55	-0.582392	0.558561	0.857	0.979	3.21E-50
DOK5	4.66E-36	-0.581979	0.558792	0.003	0.118	1.53E-31
RSPO4	2.53E-86	-0.581957	0.558804	0.009	0.288	8.32E-82
RALGPS2	7.97E-47	-0.58178	0.558902	0.834	0.968	2.62E-42
CARMIL2	3.07E-40	-0.58152	0.559048	0.033	0.234	1.01E-35
VSTM4	3.41E-26	-0.579884	0.559963	0.301	0.545	1.12E-21
PGM1	2.31E-46	-0.57952	0.560167	0.425	0.789	7.59E-42
TWIST2	5.77E-46	-0.579179	0.560358	0.919	0.981	1.90E-41
TIMM21	4.08E-45	-0.57875	0.560599	0.584	0.872	1.34E-40
CD68	6.14E-51	-0.577991	0.561024	0.447	0.803	2.02E-46
LINC00327	1.01E-31	-0.577397	0.561358	0.234	0.506	3.31E-27
TUBA1C	2.71E-71	-0.576398	0.561919	1	1	8.92E-67
VKORC1	5.95E-85	-0.576168	0.562048	0.997	1	1.96E-80
PLK1	3.01E-53	-0.576166	0.562049	0.535	0.879	9.91E-49
CEP128	3.64E-40	-0.576005	0.56214	0.219	0.55	1.20E-35
NFIX	3.20E-48	-0.575918	0.562189	0.75	0.944	1.05E-43
H4C3	2.64E-52	-0.574864	0.562782	0.294	0.677	8.68E-48
TUBB2B	1.97E-60	-0.574843	0.562793	0.862	0.979	6.47E-56
AL135926.1	3.38E-77	-0.574686	0.562882	0.01	0.278	1.11E-72
C15orf39	1.52E-33	-0.574115	0.563203	0.276	0.585	5.02E-29

FANCI	5.76E-50	-0.573776	0.563394	0.592	0.907	1.89E-45
ERLIN1	1.33E-50	-0.573626	0.563479	0.818	0.958	4.36E-46
SLC9A3R1	3.07E-43	-0.57348	0.563561	0.244	0.594	1.01E-38
ARL6IP6	1.35E-41	-0.573173	0.563734	0.71	0.91	4.46E-37
GEN1	2.01E-40	-0.572231	0.564265	0.318	0.661	6.62E-36
SGO1	5.21E-77	-0.571934	0.564433	0.308	0.787	1.72E-72
RAD18	2.33E-46	-0.571687	0.564572	0.448	0.805	7.65E-42
SESTD1	5.41E-46	-0.570831	0.565055	0.364	0.733	1.78E-41
MIS12	2.08E-40	-0.57005	0.565497	0.422	0.763	6.83E-36
FAM72A	1.65E-62	-0.569698	0.565696	0.214	0.633	5.44E-58
LRRFIP2	4.17E-62	-0.569241	0.565955	0.983	0.998	1.37E-57
KCNJ15	5.03E-36	-0.568657	0.566286	0.243	0.429	1.65E-31
ZNF219	8.99E-70	-0.568229	0.566528	0.009	0.251	2.96E-65
HOXB4	1.60E-54	-0.568127	0.566586	0.045	0.32	5.26E-50
AC098847.1	1.18E-33	-0.566924	0.567267	0.012	0.13	3.88E-29
POLE2	2.37E-40	-0.56608	0.567747	0.248	0.587	7.80E-36
FANCA	4.23E-42	-0.566067	0.567754	0.252	0.599	1.39E-37
LNX1	9.83E-44	-0.564898	0.568418	0.04	0.269	3.23E-39
ZDHHC9	1.53E-40	-0.564842	0.56845	0.503	0.817	5.03E-36
STK17B	6.91E-44	-0.564315	0.568749	0.487	0.826	2.27E-39
TENT5A	3.04E-22	-0.563792	0.569047	0.651	0.838	9.99E-18
IKBIP	4.08E-78	-0.56367	0.569117	0.997	1	1.34E-73
CCN1	6.50E-62	-0.562731	0.569651	0.999	1	2.14E-57
KDSR	3.03E-49	-0.561477	0.570366	0.862	0.981	9.98E-45
TSKU	9.03E-31	-0.56115	0.570553	0.289	0.582	2.97E-26
CALHM2	1.48E-40	-0.560622	0.570854	0.582	0.875	4.86E-36
RRM1	2.33E-57	-0.560015	0.571201	0.911	0.991	7.66E-53
CGB7	1.04E-42	-0.559097	0.571725	0.024	0.216	3.44E-38
AXIN2	1.32E-58	-0.559009	0.571776	0.015	0.244	4.34E-54
TRPV2	2.94E-34	-0.55885	0.571866	0.69	0.891	9.68E-30
MYO1D	1.83E-47	-0.558603	0.572008	0.399	0.775	6.02E-43
LIN9	5.42E-49	-0.557655	0.57255	0.237	0.61	1.78E-44
CDKN2D	3.51E-32	-0.557255	0.572779	0.516	0.789	1.15E-27
ATAD3B	2.43E-48	-0.556992	0.57293	0.496	0.842	7.99E-44
MFHAS1	2.07E-37	-0.556898	0.572984	0.272	0.601	6.82E-33
GALNT6	5.14E-41	-0.556764	0.573061	0.754	0.93	1.69E-36
DKK3	2.04E-115	-0.556549	0.573184	1	1	6.72E-111
HLA-F	1.82E-43	-0.556491	0.573217	0.127	0.434	5.99E-39
HSPB2-C11o	6.72E-67	-0.556084	0.57345	0.092	0.462	2.21E-62
ARG2	3.38E-30	-0.556015	0.57349	0.086	0.309	1.11E-25

CENPP	1.63E-41	-0.555606	0.573725	0.174	0.497	5.35E-37
WDR62	4.84E-44	-0.555514	0.573778	0.252	0.601	1.59E-39
NRIP3	5.80E-34	-0.555361	0.573865	0.556	0.826	1.91E-29
RFWD3	1.05E-44	-0.55518	0.573969	0.445	0.798	3.44E-40
C3orf70	1.73E-30	-0.554639	0.57428	0.083	0.302	5.69E-26
NAV2	6.14E-54	-0.554617	0.574292	0.273	0.673	2.02E-49
AK5	6.75E-51	-0.553651	0.574847	0.942	0.986	2.22E-46
SERPINB8	8.99E-43	-0.553635	0.574856	0.737	0.942	2.96E-38
CGB5	4.38E-40	-0.553301	0.575049	0.022	0.197	1.44E-35
FRAS1	3.23E-44	-0.552996	0.575224	0.014	0.197	1.06E-39
PTPRU	2.21E-45	-0.5525	0.575509	0.051	0.302	7.27E-41
TNFAIP3	1.30E-33	-0.552133	0.57572	0.103	0.353	4.28E-29
POLR3H	2.57E-41	-0.552014	0.575789	0.61	0.891	8.45E-37
IL11RA	3.89E-40	-0.55151	0.576079	0.26	0.599	1.28E-35
KREMEN1	3.21E-32	-0.55133	0.576183	0.265	0.466	1.06E-27
HOXC4	5.94E-100	-0.551255	0.576226	0.018	0.371	1.96E-95
NR2C2AP	5.38E-38	-0.551047	0.576346	0.236	0.561	1.77E-33
PIK3CD	7.37E-31	-0.550975	0.576387	0.265	0.557	2.42E-26
RABGEF1	1.93E-41	-0.550641	0.57658	0.675	0.879	6.35E-37
YEATS4	4.75E-41	-0.550628	0.576587	0.502	0.826	1.56E-36
SLC41A2	1.71E-32	-0.550528	0.576646	0.087	0.32	5.61E-28
HROB	3.16E-38	-0.54925	0.577383	0.122	0.404	1.04E-33
RUNX1T1	1.70E-43	-0.549234	0.577392	0.425	0.775	5.59E-39
CKAP2	4.97E-47	-0.549108	0.577465	0.737	0.926	1.64E-42
GAS2L1	1.25E-34	-0.548999	0.577528	0.559	0.812	4.12E-30
TNFRSF1A	1.59E-50	-0.548855	0.577611	0.857	0.986	5.23E-46
STK10	3.21E-32	-0.547269	0.578528	0.515	0.773	1.06E-27
MARCHF3	6.98E-25	-0.547137	0.578604	0.213	0.464	2.30E-20
ANLN	1.82E-56	-0.547112	0.578618	0.77	0.956	5.99E-52
NFIB	6.03E-30	-0.546703	0.578855	0.048	0.216	1.98E-25
MT1X	1.56E-38	-0.546182	0.579157	0.455	0.754	5.14E-34
RCAN2	8.58E-39	-0.54586	0.579343	0.033	0.234	2.82E-34
NUSAP1	1.17E-57	-0.54584	0.579355	0.638	0.926	3.85E-53
FCRLB	9.24E-37	-0.545636	0.579473	0.204	0.515	3.04E-32
TMPO-AS1	8.02E-48	-0.545313	0.57966	0.125	0.445	2.64E-43
SYTL3	1.90E-31	-0.544851	0.579928	0.117	0.367	6.24E-27
TOP2A	6.07E-75	-0.543914	0.580472	0.78	0.961	2.00E-70
IRX3	2.70E-110	-0.543632	0.580636	0.009	0.355	8.89E-106
OIP5	2.08E-37	-0.543328	0.580812	0.319	0.654	6.84E-33
DLEU1	3.83E-35	-0.541626	0.581802	0.352	0.671	1.26E-30

COPZ2	3.56E-54	-0.541461	0.581897	0.946	0.993	1.17E-49
H1-0	7.04E-21	-0.540344	0.582548	0.88	0.93	2.32E-16
AUP1	4.40E-50	-0.540063	0.582712	0.957	0.993	1.45E-45
C2CD2	5.46E-30	-0.539987	0.582756	0.486	0.754	1.80E-25
NCEH1	1.79E-40	-0.539337	0.583135	0.84	0.958	5.88E-36
RPA2	1.71E-49	-0.539036	0.58331	0.564	0.896	5.62E-45
CRYL1	6.58E-40	-0.538862	0.583412	0.202	0.527	2.16E-35
POLD1	6.35E-41	-0.538398	0.583682	0.33	0.68	2.09E-36
GLI3	6.89E-34	-0.538254	0.583767	0.288	0.603	2.27E-29
MPP4	1.54E-23	-0.538178	0.583811	0.178	0.399	5.06E-19
THUMPD3-A	6.93E-34	-0.53814	0.583833	0.483	0.768	2.28E-29
UST	5.14E-39	-0.536867	0.584577	0.23	0.557	1.69E-34
SPARC	1.01E-130	-0.536501	0.584791	1	1	3.33E-126
CUBN	2.99E-42	-0.536079	0.585038	0.068	0.316	9.83E-38
HNRNPAB	2.72E-65	-0.53541	0.585429	1	1	8.95E-61
DLX2	8.89E-24	-0.535401	0.585434	0.092	0.288	2.93E-19
C5orf17	3.17E-49	-0.535207	0.585548	0.042	0.281	1.04E-44
SLC27A4	2.39E-38	-0.534837	0.585765	0.437	0.768	7.85E-34
EXOSC9	5.40E-45	-0.534824	0.585772	0.864	0.984	1.78E-40
C1orf112	7.97E-37	-0.534702	0.585844	0.222	0.538	2.62E-32
WWC1	5.19E-39	-0.534107	0.586193	0.03	0.204	1.71E-34
FOXM1	3.91E-43	-0.534048	0.586227	0.736	0.937	1.29E-38
KLF2	2.08E-26	-0.53381	0.586367	0.713	0.886	6.84E-22
FAAP24	1.77E-29	-0.533174	0.58674	0.217	0.485	5.82E-25
WDFY2	3.30E-39	-0.53281	0.586953	0.556	0.856	1.09E-34
CSMD2	4.48E-33	-0.532173	0.587328	0.106	0.346	1.48E-28
GSTCD	2.00E-39	-0.532166	0.587331	0.295	0.636	6.57E-35
FIGNL1	1.90E-34	-0.532113	0.587362	0.245	0.555	6.26E-30
SIPA1L3	7.37E-31	-0.53205	0.5874	0.312	0.603	2.43E-26
GNPNAT1	1.98E-48	-0.531558	0.587689	0.95	0.991	6.53E-44
PCBP3	1.21E-53	-0.531427	0.587765	0.013	0.218	3.99E-49
GPX7	3.26E-39	-0.53142	0.58777	0.754	0.942	1.07E-34
BCL2L12	2.45E-43	-0.531045	0.58799	0.503	0.835	8.06E-39
DSCC1	2.53E-37	-0.53088	0.588087	0.182	0.487	8.33E-33
AC087392.1	8.45E-38	-0.530643	0.588227	0.178	0.485	2.78E-33
SLC7A5	3.26E-18	-0.530198	0.588488	0.774	0.872	1.07E-13
SPAG9	1.30E-45	-0.52991	0.588658	0.893	0.974	4.28E-41
SNED1	9.20E-45	-0.528546	0.589461	0.024	0.232	3.03E-40
CHAC2	3.22E-32	-0.528154	0.589692	0.217	0.51	1.06E-27
TES	1.89E-42	-0.527297	0.590198	0.724	0.935	6.20E-38

B4GALT1	9.58E-47	-0.526836	0.59047	0.976	0.995	3.15E-42
PPIB	1.31E-144	-0.526689	0.590557	1	1	4.30E-140
CCDC15	6.37E-41	-0.526518	0.590658	0.173	0.492	2.10E-36
NUP35	2.78E-37	-0.525938	0.591001	0.339	0.673	9.14E-33
CDC25A	4.52E-36	-0.525804	0.59108	0.179	0.476	1.49E-31
DEPDC1B	1.41E-40	-0.525763	0.591104	0.205	0.531	4.65E-36
NUP85	3.93E-40	-0.525502	0.591259	0.581	0.872	1.29E-35
KPNA2	1.55E-50	-0.525273	0.591394	0.961	0.984	5.12E-46
SRD5A3	3.15E-30	-0.524834	0.591654	0.405	0.696	1.04E-25
C9orf40	1.86E-44	-0.524789	0.59168	0.488	0.828	6.13E-40
GMPPB	2.16E-39	-0.524379	0.591923	0.64	0.898	7.11E-35
GINS2	1.65E-44	-0.524243	0.592004	0.418	0.782	5.41E-40
MIR155HG	7.21E-33	-0.524011	0.592141	0.068	0.288	2.37E-28
CDK2AP2	1.27E-41	-0.523704	0.592322	0.827	0.965	4.17E-37
CCDC74B	2.11E-40	-0.523551	0.592413	0.109	0.392	6.95E-36
MMD	7.85E-30	-0.523064	0.592702	0.203	0.478	2.58E-25
NAP1L3	3.93E-37	-0.522815	0.592849	0.037	0.239	1.29E-32
AL139220.2	1.40E-25	-0.522509	0.593031	0.225	0.434	4.62E-21
MCM5	1.64E-38	-0.521941	0.593367	0.698	0.916	5.40E-34
SH3D19	1.03E-40	-0.520512	0.594216	0.666	0.926	3.37E-36
BIRC5	1.71E-44	-0.520424	0.594268	0.826	0.956	5.63E-40
MCM7	4.21E-47	-0.519996	0.594523	0.827	0.981	1.38E-42
FAM83G	1.85E-27	-0.519792	0.594644	0.289	0.555	6.09E-23
EMP3	9.00E-112	-0.519547	0.59479	1	1	2.96E-107
HAUS2	2.27E-39	-0.51937	0.594895	0.677	0.916	7.48E-35
CD47	2.21E-56	-0.51915	0.595026	0.989	1	7.27E-52
FGFRL1	1.15E-23	-0.519026	0.5951	0.511	0.747	3.80E-19
TTLL12	3.75E-35	-0.518774	0.59525	0.468	0.773	1.23E-30
PKIA	3.27E-20	-0.518216	0.595582	0.341	0.568	1.08E-15
ARL4D	1.27E-36	-0.517025	0.596292	0.322	0.654	4.17E-32
WNT16	8.43E-39	-0.516481	0.596617	0.005	0.137	2.77E-34
PAX6	1.41E-35	-0.5149	0.597561	0.033	0.2	4.64E-31
FAM110B	1.43E-29	-0.514837	0.597598	0.072	0.278	4.70E-25
AL163636.2	6.29E-48	-0.514247	0.597951	0.194	0.548	2.07E-43
NIPA1	6.79E-27	-0.514174	0.597995	0.199	0.457	2.23E-22
TEDC2	1.41E-40	-0.513984	0.598108	0.184	0.506	4.65E-36
VAMP4	1.51E-33	-0.513591	0.598343	0.488	0.773	4.98E-29
LRRC59	1.70E-76	-0.513538	0.598375	0.999	1	5.60E-72
C15orf41	1.42E-25	-0.51328	0.598529	0.384	0.65	4.68E-21
LSP1P5	1.32E-53	-0.513221	0.598565	0.845	0.979	4.35E-49

KCNS3	6.15E-34	-0.512443	0.599031	0.063	0.283	2.02E-29
ZIC1	7.42E-48	-0.512268	0.599135	0.01	0.181	2.44E-43
MAMLD1	1.29E-29	-0.511974	0.599311	0.07	0.278	4.24E-25
ERRFI1	4.57E-32	-0.511766	0.599436	0.839	0.97	1.50E-27
PLIN3	2.22E-74	-0.511759	0.59944	1	1	7.31E-70
MANEA	6.20E-44	-0.511395	0.599659	0.604	0.905	2.04E-39
FARSB	2.60E-44	-0.51123	0.599757	0.874	0.981	8.55E-40
MIR924HG	2.52E-43	-0.511147	0.599807	0.043	0.276	8.30E-39
SCIN	7.39E-17	-0.511105	0.599832	0.119	0.206	2.43E-12
ADAMTSL4	4.28E-25	-0.51063	0.600117	0.091	0.29	1.41E-20
ANO3	5.46E-29	-0.509905	0.600553	0.016	0.144	1.80E-24
HTRA2	1.25E-49	-0.509497	0.600798	0.49	0.856	4.11E-45
AC098582.1	8.86E-50	-0.508784	0.601226	0.375	0.756	2.91E-45
RCAN3	9.15E-32	-0.50825	0.601548	0.592	0.835	3.01E-27
LAMA2	2.44E-33	-0.506655	0.602508	0.336	0.652	8.03E-29
NEIL3	6.25E-61	-0.506648	0.602512	0.242	0.657	2.06E-56
DNAJC6	1.11E-28	-0.505478	0.603217	0.04	0.209	3.67E-24
MAPKAP1	2.06E-47	-0.50526	0.603349	0.949	0.993	6.76E-43
AC080038.1	3.34E-29	-0.505041	0.603481	0.125	0.367	1.10E-24
LINC01119	2.66E-21	-0.50492	0.603554	0.11	0.304	8.76E-17
AMDHD2	8.11E-32	-0.504893	0.60357	0.214	0.503	2.67E-27
AGTPBP1	3.20E-48	-0.504721	0.603674	0.054	0.318	1.05E-43
SLC25A19	2.53E-29	-0.504389	0.603874	0.146	0.399	8.32E-25
TTTY14	4.14E-41	-0.504272	0.603945	0.063	0.302	1.36E-36
PRUNE2	2.19E-39	-0.504026	0.604093	0.008	0.158	7.20E-35
POLR1E	1.05E-32	-0.503438	0.604449	0.435	0.735	3.45E-28
NSD2	3.51E-44	-0.503405	0.604469	0.817	0.979	1.16E-39
TMEM171	2.08E-25	-0.502914	0.604766	0.083	0.281	6.83E-21
GTF2F2	1.23E-40	-0.502433	0.605057	0.961	0.995	4.05E-36
SYTL2	2.66E-24	-0.502414	0.605068	0.521	0.738	8.76E-20
EIF4EBP1	3.17E-40	-0.502379	0.60509	0.969	0.995	1.04E-35
TUBB4B	2.34E-52	-0.502307	0.605133	1	1	7.71E-48
RAB11FIP5	8.61E-34	-0.502143	0.605232	0.652	0.884	2.83E-29
TUBG2	6.15E-48	-0.501099	0.605864	0.411	0.787	2.02E-43
REEP4	1.83E-37	-0.501048	0.605895	0.522	0.831	6.02E-33
ENO2	2.80E-38	-0.499269	0.606974	0.345	0.684	9.21E-34
IRX1	7.11E-56	-0.499141	0.607052	0.004	0.183	2.34E-51
PALM2AKAP	5.75E-46	-0.498513	0.607433	0.99	1	1.89E-41
TOMM5	1.45E-54	-0.498253	0.607591	0.974	0.998	4.77E-50
BTBD11	9.57E-31	-0.497802	0.607865	0.043	0.227	3.15E-26

BDKRB1	2.41E-46	-0.497788	0.607874	0.284	0.654	7.94E-42
ATL1	3.42E-20	-0.497485	0.608058	0.109	0.281	1.12E-15
SP9	5.88E-51	-0.497402	0.608108	0.007	0.186	1.93E-46
FSTL3	1.98E-29	-0.496021	0.608949	0.659	0.879	6.51E-25
JAM2	8.15E-25	-0.495259	0.609413	0.059	0.209	2.68E-20
NUP62	3.13E-40	-0.494808	0.609688	0.915	0.986	1.03E-35
SAMD1	1.42E-31	-0.49469	0.60976	0.58	0.838	4.69E-27
CGB3	2.42E-41	-0.494637	0.609792	0.017	0.193	7.97E-37
PGP	7.08E-41	-0.494445	0.609909	0.633	0.903	2.33E-36
NFATC2IP	2.81E-36	-0.494407	0.609933	0.658	0.889	9.25E-32
RETREG1	2.46E-33	-0.49419	0.610065	0.004	0.121	8.09E-29
ARNTL	4.24E-25	-0.493889	0.610248	0.22	0.476	1.40E-20
MICAL2	7.36E-43	-0.493858	0.610268	0.968	0.991	2.42E-38
XDH	3.42E-32	-0.493845	0.610275	0.04	0.213	1.12E-27
METTL27	3.03E-40	-0.49368	0.610376	0.043	0.26	9.95E-36
NUDT8	6.79E-36	-0.493388	0.610554	0.133	0.411	2.24E-31
SH3BGRL3	1.20E-141	-0.493267	0.610628	1	1	3.95E-137
TLCD3A	1.33E-30	-0.492992	0.610796	0.453	0.747	4.38E-26
NR3C1	4.30E-38	-0.49278	0.610926	0.961	0.988	1.41E-33
NPAS1	1.02E-39	-0.49267	0.610993	0.037	0.246	3.36E-35
EVI2A	1.27E-33	-0.492655	0.611002	0.066	0.288	4.18E-29
E2F8	1.07E-35	-0.492642	0.61101	0.08	0.323	3.51E-31
GRIA3	2.70E-27	-0.492497	0.611098	0.143	0.385	8.87E-23
DONSON	1.56E-31	-0.491852	0.611493	0.5	0.78	5.13E-27
ENDOD1	2.10E-36	-0.491369	0.611788	0.585	0.868	6.91E-32
NNMT	6.77E-24	-0.49117	0.61191	0.795	0.923	2.23E-19
ADARB1	1.69E-26	-0.491113	0.611945	0.183	0.436	5.56E-22
LRIG1	4.18E-20	-0.491091	0.611959	0.102	0.285	1.37E-15
SUB1	4.59E-97	-0.490957	0.61204	1	1	1.51E-92
KIF23	1.59E-50	-0.490433	0.612361	0.715	0.94	5.22E-46
CENPA	8.00E-49	-0.489619	0.61286	0.349	0.733	2.63E-44
STC1	2.17E-04	-0.489407	0.61299	0.665	0.722	1.00E+00
EFR3B	1.45E-36	-0.489367	0.613014	0.098	0.304	4.78E-32
AC087721.2	3.89E-62	-0.488558	0.613511	0.494	0.896	1.28E-57
CHAF1B	5.18E-30	-0.488336	0.613646	0.33	0.626	1.70E-25
SASS6	3.02E-35	-0.488292	0.613674	0.241	0.552	9.95E-31
VXN	1.37E-43	-0.487755	0.614003	0.01	0.179	4.50E-39
COL5A1	4.07E-42	-0.48766	0.614062	0.999	1	1.34E-37
SCML1	3.14E-34	-0.487561	0.614123	0.525	0.826	1.03E-29
PLP2	2.47E-47	-0.487483	0.61417	0.99	1	8.13E-43

MMRN2	9.63E-24	-0.487196	0.614347	0.027	0.155	3.17E-19
SOCS3	9.83E-28	-0.487093	0.61441	0.553	0.805	3.23E-23
CD58	1.65E-32	-0.48683	0.614572	0.321	0.631	5.43E-28
FURIN	2.19E-35	-0.486709	0.614646	0.741	0.93	7.21E-31
C16orf95	1.82E-38	-0.486674	0.614667	0.128	0.415	5.99E-34
TNFRSF12A	6.82E-78	-0.485921	0.61513	1	1	2.24E-73
TRAF3IP2	2.94E-31	-0.485915	0.615134	0.543	0.814	9.66E-27
NUP50	4.95E-39	-0.485868	0.615163	0.782	0.942	1.63E-34
NEMP1	1.21E-36	-0.48531	0.615506	0.316	0.647	4.00E-32
PARP2	6.04E-39	-0.48503	0.615679	0.494	0.819	1.99E-34
GIPC2	3.77E-41	-0.484927	0.615742	0.028	0.197	1.24E-36
GBP5	1.03E-22	-0.484903	0.615757	0.045	0.181	3.38E-18
CIB1	1.89E-47	-0.484736	0.61586	0.967	1	6.22E-43
YIF1A	4.14E-48	-0.483957	0.61634	0.969	0.998	1.36E-43
LINC02535	4.68E-20	-0.483905	0.616372	0.032	0.158	1.54E-15
PLPP4	3.92E-22	-0.483524	0.616607	0.339	0.592	1.29E-17
GGCT	7.43E-37	-0.483226	0.61679	0.582	0.858	2.45E-32
ITPRIP	1.28E-34	-0.482663	0.617138	0.564	0.847	4.21E-30
IFITM10	1.76E-35	-0.482274	0.617378	0.1	0.357	5.79E-31
COL1A2	1.95E-68	-0.481806	0.617667	1	1	6.40E-64
CGB1	4.86E-40	-0.481451	0.617886	0.017	0.186	1.60E-35
MAGI3	7.19E-31	-0.481007	0.61816	0.23	0.52	2.36E-26
AKR7A2	1.44E-35	-0.480289	0.618605	0.833	0.958	4.74E-31
FLI1	4.65E-22	-0.480259	0.618623	0.096	0.283	1.53E-17
RASIP1	8.61E-26	-0.480163	0.618683	0.021	0.155	2.83E-21
TFDP1	5.77E-47	-0.480103	0.61872	0.972	0.993	1.90E-42
KNTC1	5.63E-36	-0.479843	0.618881	0.357	0.687	1.85E-31
ARL2BP	3.32E-53	-0.47983	0.618889	0.993	1	1.09E-48
ITGB3BP	1.49E-42	-0.479805	0.618904	0.452	0.803	4.91E-38
BLM	6.15E-27	-0.479599	0.619031	0.224	0.487	2.02E-22
IDH2	3.00E-39	-0.478967	0.619423	0.681	0.928	9.87E-35
TUSC1	4.09E-33	-0.478768	0.619546	0.317	0.629	1.35E-28
ELK3	4.71E-39	-0.478316	0.619826	0.922	0.981	1.55E-34
SIPA1L1	2.44E-28	-0.478087	0.619968	0.534	0.798	8.02E-24
RHEBL1	1.84E-29	-0.47808	0.619973	0.04	0.216	6.05E-25
EIF5	3.21E-71	-0.477861	0.620108	1	1	1.06E-66
IGF2BP3	1.29E-17	-0.477746	0.62018	0.317	0.534	4.23E-13
NOP16	2.06E-31	-0.477586	0.620279	0.711	0.903	6.78E-27
PPP1R3C	1.89E-14	-0.477511	0.620325	0.396	0.596	6.23E-10
KIF18B	6.11E-60	-0.476786	0.620776	0.275	0.694	2.01E-55

NCAPD3	3.69E-44	-0.476699	0.620829	0.509	0.852	1.22E-39
S100A16	2.30E-53	-0.476599	0.620891	1	1	7.57E-49
GLTP	4.98E-43	-0.476582	0.620902	0.965	0.991	1.64E-38
GEMIN4	4.47E-30	-0.476561	0.620915	0.312	0.61	1.47E-25
RAD54B	2.92E-34	-0.476436	0.620992	0.237	0.545	9.60E-30
RAN	9.91E-87	-0.476214	0.621131	1	1	3.26E-82
SLC9B2	1.49E-30	-0.476009	0.621258	0.427	0.717	4.90E-26
TMEM255B	6.66E-53	-0.47537	0.621655	0.022	0.251	2.19E-48
ETV1	3.11E-24	-0.475198	0.621762	0.322	0.589	1.02E-19
RNASEH2A	6.77E-44	-0.474791	0.622015	0.704	0.935	2.23E-39
LOXL1-AS1	5.88E-24	-0.474585	0.622143	0.382	0.64	1.93E-19
SMC4	1.04E-53	-0.473925	0.622554	0.929	0.993	3.42E-49
UCK2	1.48E-36	-0.47368	0.622706	0.739	0.93	4.87E-32
MCM8	1.03E-38	-0.473546	0.62279	0.454	0.784	3.39E-34
IMP3	7.64E-37	-0.472636	0.623357	0.82	0.958	2.51E-32
FZR1	3.27E-25	-0.472272	0.623584	0.525	0.775	1.08E-20
LIN54	1.15E-37	-0.472157	0.623656	0.426	0.759	3.79E-33
SLC49A3	6.12E-44	-0.472104	0.623688	0.03	0.241	2.01E-39
CCN5	1.30E-17	-0.472002	0.623752	0.025	0.116	4.27E-13
MZT1	5.08E-43	-0.471907	0.623812	0.848	0.977	1.67E-38
TIMM13	7.27E-47	-0.471133	0.624294	0.978	0.998	2.39E-42
NFKBIA	3.58E-15	-0.470879	0.624453	0.854	0.916	1.18E-10
TOE1	5.06E-34	-0.470779	0.624516	0.275	0.589	1.66E-29
FN3KRP	1.87E-32	-0.469824	0.625112	0.594	0.856	6.15E-28
KNSTRN	8.66E-49	-0.46969	0.625196	0.486	0.852	2.85E-44
C5orf38	5.69E-85	-0.469574	0.625268	0.003	0.258	1.87E-80
CSF1	7.42E-32	-0.469469	0.625334	0.5	0.796	2.44E-27
MTSS1	2.57E-37	-0.46885	0.625722	0.026	0.206	8.46E-33
PTTG1IP	8.73E-70	-0.468675	0.625831	1	1	2.87E-65
PNRC2	1.10E-40	-0.46845	0.625972	0.945	0.988	3.61E-36
RELT	1.24E-29	-0.468434	0.625982	0.14	0.392	4.07E-25
PRR7	1.99E-23	-0.467976	0.626269	0.173	0.404	6.54E-19
STARD8	1.83E-31	-0.467537	0.626543	0.06	0.262	6.02E-27
PRADC1	4.91E-41	-0.46712	0.626805	0.608	0.903	1.61E-36
EZH2	1.28E-37	-0.466768	0.627026	0.524	0.835	4.22E-33
PTRH1	6.01E-38	-0.466716	0.627058	0.407	0.74	1.98E-33
CLEC3B	1.92E-22	-0.466664	0.627091	0.066	0.234	6.31E-18
ASL	9.29E-34	-0.466235	0.62736	0.798	0.944	3.06E-29
KIF4A	1.65E-57	-0.465874	0.627586	0.44	0.847	5.43E-53
PDP1	1.51E-28	-0.465379	0.627897	0.729	0.905	4.96E-24

AQP1	8.20E-14	-0.465245	0.627981	0.033	0.121	2.70E-09
FAR1	1.37E-35	-0.464851	0.628229	0.72	0.928	4.49E-31
PRDX4	8.55E-61	-0.464669	0.628343	0.999	1	2.81E-56
MYDGF	8.51E-103	-0.464612	0.628379	1	1	2.80E-98
ANKRD29	6.12E-43	-0.464018	0.628752	0.022	0.218	2.01E-38
STEAP2	1.29E-21	-0.463747	0.628923	0.208	0.439	4.26E-17
RPL39L	1.69E-44	-0.463284	0.629214	0.6	0.907	5.56E-40
MCM3	6.71E-29	-0.46323	0.629248	0.574	0.831	2.21E-24
SSR3	1.74E-92	-0.463052	0.62936	1	1	5.71E-88
TUBB6	6.38E-63	-0.462943	0.629429	1	1	2.10E-58
STEAP3	1.32E-27	-0.462621	0.629631	0.651	0.863	4.34E-23
XRCC3	1.77E-34	-0.461993	0.630027	0.174	0.464	5.82E-30
PRKG1	7.47E-30	-0.461792	0.630154	0.289	0.585	2.46E-25
WIPF1	9.51E-34	-0.461544	0.63031	0.697	0.919	3.13E-29
LDHA	2.63E-103	-0.461491	0.630343	1	1	8.64E-99
PTPDC1	3.65E-33	-0.461341	0.630438	0.164	0.443	1.20E-28
JPT2	1.03E-36	-0.461301	0.630463	0.848	0.968	3.40E-32
STK32B	1.40E-31	-0.461207	0.630522	0.013	0.151	4.60E-27
UTY	1.31E-51	-0.460997	0.630655	0.011	0.2	4.31E-47
CENPN	1.18E-37	-0.460238	0.631134	0.721	0.93	3.88E-33
ADA	2.30E-24	-0.459953	0.631313	0.202	0.448	7.58E-20
MACROD1	1.49E-28	-0.45988	0.631359	0.189	0.455	4.89E-24
HSPA5	2.85E-68	-0.459703	0.631471	1	1	9.36E-64
CKS1B	1.23E-44	-0.459177	0.631804	0.854	0.974	4.05E-40
GOLM1	2.91E-36	-0.459068	0.631872	0.774	0.954	9.57E-32
ATP8B2	7.18E-32	-0.458878	0.631992	0.562	0.833	2.36E-27
ENOSF1	9.51E-27	-0.458749	0.632074	0.357	0.631	3.13E-22
SPAG5	3.16E-49	-0.458488	0.632239	0.475	0.845	1.04E-44
MCM2	2.52E-42	-0.458319	0.632346	0.432	0.787	8.29E-38
RBBP8	1.03E-29	-0.458309	0.632352	0.593	0.842	3.40E-25
ANG	2.87E-25	-0.458167	0.632442	0.119	0.339	9.43E-21
ZDHHC6	3.41E-34	-0.457725	0.632721	0.661	0.896	1.12E-29
MALT1	4.22E-27	-0.45768	0.63275	0.698	0.889	1.39E-22
ME3	1.56E-24	-0.457549	0.632833	0.151	0.381	5.12E-20
TMEM50B	2.98E-29	-0.457422	0.632913	0.83	0.958	9.80E-25
TYMP	7.33E-26	-0.457138	0.633093	0.247	0.513	2.41E-21
RANBP1	8.85E-53	-0.457093	0.633122	0.998	1	2.91E-48
LSM2	2.76E-38	-0.456853	0.633274	0.856	0.984	9.07E-34
SPCS3	3.73E-50	-0.456663	0.633394	0.998	1	1.23E-45
GHR	1.09E-22	-0.456388	0.633568	0.066	0.232	3.60E-18

LRRC58	1.18E-31	-0.456117	0.63374	0.909	0.974	3.88E-27
ELFN1	1.26E-27	-0.455722	0.63399	0.051	0.23	4.14E-23
RHNO1	1.15E-34	-0.455448	0.634164	0.571	0.849	3.77E-30
BEND7	1.40E-32	-0.455442	0.634168	0.113	0.334	4.61E-28
FKBP9	7.38E-42	-0.454812	0.634567	0.982	0.993	2.43E-37
AGTR1	9.26E-36	-0.454807	0.634571	0.015	0.174	3.05E-31
EHHADH	1.22E-27	-0.454685	0.634648	0.192	0.452	4.02E-23
AGAP1	9.24E-21	-0.454629	0.634684	0.329	0.575	3.04E-16
BARD1	7.54E-34	-0.454514	0.634757	0.394	0.712	2.48E-29
RHOU	1.39E-28	-0.454335	0.63487	0.057	0.248	4.57E-24
AC124067.2	2.99E-38	-0.454271	0.634911	0.047	0.265	9.85E-34
MCF2L	7.61E-38	-0.454059	0.635045	0.046	0.244	2.50E-33
TMED3	2.47E-40	-0.453788	0.635218	0.976	0.995	8.12E-36
ZFAND2A	4.08E-33	-0.453678	0.635287	0.427	0.74	1.34E-28
CAPN5	8.03E-24	-0.453417	0.635453	0.43	0.689	2.64E-19
MTBP	7.93E-33	-0.453337	0.635504	0.169	0.448	2.61E-28
C1orf174	6.92E-28	-0.453322	0.635513	0.508	0.78	2.28E-23
FKBP1A	1.87E-74	-0.453311	0.63552	1	1	6.17E-70
HASPIN	3.59E-26	-0.452979	0.635731	0.082	0.283	1.18E-21
TRPS1	1.54E-14	-0.452816	0.635835	0.411	0.61	5.08E-10
TPCN1	1.79E-18	-0.452234	0.636205	0.207	0.418	5.88E-14
AL109918.1	2.01E-35	-0.452044	0.636326	0.2	0.499	6.63E-31
LRP3	3.53E-33	-0.451706	0.636541	0.258	0.564	1.16E-28
B3GNT2	9.68E-29	-0.451414	0.636727	0.243	0.524	3.18E-24
TMEM132B	5.75E-17	-0.451148	0.636896	0.081	0.232	1.89E-12
DOCK10	9.75E-34	-0.450993	0.636995	0.574	0.861	3.21E-29
CGB2	2.88E-44	-0.450618	0.637234	0.013	0.188	9.47E-40
EFL1	4.45E-29	-0.450402	0.637372	0.41	0.701	1.46E-24
SVIL	2.73E-13	-0.45037	0.637393	0.136	0.281	8.99E-09
CGB8	1.30E-39	-0.450288	0.637445	0.022	0.202	4.28E-35
HBEGF	5.95E-22	-0.450241	0.637474	0.284	0.534	1.96E-17
TUBG1	1.42E-39	-0.450125	0.637549	0.88	0.979	4.68E-35
NMB	9.24E-12	-0.449991	0.637634	0.308	0.478	3.04E-07
SDC3	3.44E-45	-0.449875	0.637708	0.947	0.998	1.13E-40
ADGRL2	5.17E-29	-0.449869	0.637711	0.265	0.552	1.70E-24
TNFRSF6B	3.56E-43	-0.448922	0.638316	0.053	0.292	1.17E-38
HOTAIR	8.97E-39	-0.448077	0.638856	0.014	0.179	2.95E-34
UHRF1	3.49E-32	-0.447959	0.638931	0.628	0.879	1.15E-27
C21orf58	1.10E-34	-0.447863	0.638992	0.17	0.459	3.63E-30
ARNTL2	2.40E-29	-0.447108	0.639475	0.612	0.852	7.89E-25

LAMB3	6.08E-21	-0.446429	0.639909	0.095	0.278	2.00E-16
RSRC1	2.15E-35	-0.446293	0.639996	0.631	0.889	7.08E-31
LYRM7	2.91E-27	-0.446017	0.640173	0.692	0.863	9.57E-23
SGMS1	3.54E-31	-0.445953	0.640214	0.58	0.852	1.16E-26
HLA-DMA	5.45E-46	-0.445645	0.640411	0.02	0.223	1.79E-41
NIP7	2.08E-33	-0.445026	0.640808	0.874	0.974	6.84E-29
SH3BP5	9.47E-21	-0.444736	0.640994	0.379	0.624	3.12E-16
B4GALT5	2.37E-31	-0.444332	0.641253	0.555	0.833	7.78E-27
CCDC34	1.27E-37	-0.444299	0.641274	0.49	0.814	4.16E-33
PTGFRN	1.49E-27	-0.444144	0.641373	0.041	0.209	4.91E-23
PRR11	2.69E-39	-0.443998	0.641467	0.867	0.963	8.85E-35
CERK	1.04E-27	-0.443847	0.641563	0.728	0.912	3.44E-23
BAIAP2	4.05E-19	-0.443793	0.641598	0.423	0.654	1.33E-14
CCDC18	4.80E-31	-0.443624	0.641707	0.339	0.645	1.58E-26
AKR1E2	5.17E-27	-0.443607	0.641718	0.072	0.269	1.70E-22
DCAF15	1.34E-41	-0.443348	0.641884	0.369	0.724	4.42E-37
PMAIP1	8.29E-15	-0.443059	0.642069	0.902	0.944	2.73E-10
SGCB	1.14E-48	-0.442894	0.642175	0.996	0.995	3.75E-44
B4GALT2	1.08E-34	-0.442608	0.642359	0.816	0.961	3.54E-30
PMM1	5.11E-32	-0.442388	0.6425	0.894	0.977	1.68E-27
RPS6KA4	8.08E-27	-0.442184	0.642631	0.494	0.759	2.66E-22
HSPB3	4.76E-41	-0.442056	0.642714	0.007	0.16	1.57E-36
MMS22L	1.00E-25	-0.441916	0.642804	0.62	0.833	3.30E-21
LYAR	1.70E-27	-0.441564	0.64303	0.662	0.879	5.60E-23
AC010327.4	5.96E-63	-0.441432	0.643115	0.024	0.29	1.96E-58
BCL10	1.52E-34	-0.441184	0.643274	0.829	0.965	5.01E-30
MGST3	9.35E-63	-0.441037	0.643369	0.998	1	3.08E-58
DOK1	7.74E-21	-0.441008	0.643388	0.318	0.564	2.55E-16
SERTAD1	1.14E-27	-0.440945	0.643428	0.775	0.93	3.75E-23
ADIRF-AS1	1.20E-25	-0.440503	0.643713	0.112	0.329	3.93E-21
DPM2	2.52E-32	-0.440497	0.643716	0.814	0.947	8.28E-28
RTEL1	7.27E-31	-0.440407	0.643775	0.189	0.466	2.39E-26
FAM241A	5.26E-24	-0.439737	0.644206	0.491	0.742	1.73E-19
VCPKMT	6.11E-22	-0.439367	0.644444	0.236	0.478	2.01E-17
TMED8	8.72E-25	-0.439259	0.644514	0.24	0.499	2.87E-20
SRM	3.34E-40	-0.438516	0.644993	0.961	0.991	1.10E-35
MAFF	3.19E-25	-0.438306	0.645128	0.61	0.84	1.05E-20
SUMO3	5.82E-42	-0.437836	0.645432	0.98	0.995	1.92E-37
NCLN	4.80E-39	-0.437738	0.645495	0.888	0.984	1.58E-34
CLN6	3.63E-35	-0.437216	0.645832	0.483	0.794	1.20E-30

ERI2	2.65E-32	-0.436951	0.646003	0.342	0.654	8.72E-28
ECHDC3	1.17E-20	-0.436229	0.646469	0.089	0.267	3.85E-16
CCNYL1	6.92E-27	-0.436213	0.64648	0.433	0.715	2.28E-22
ATAD5	9.71E-36	-0.435966	0.64664	0.26	0.578	3.19E-31
MIS18BP1	5.95E-33	-0.43594	0.646657	0.752	0.935	1.96E-28
AADAT	9.45E-25	-0.435856	0.646711	0.272	0.536	3.11E-20
ODC1	5.44E-31	-0.435753	0.646778	0.927	0.981	1.79E-26
SELENOK	1.07E-36	-0.43553	0.646922	0.814	0.968	3.52E-32
CNIH3	4.27E-15	-0.435393	0.64701	0.171	0.346	1.40E-10
TICRR	1.92E-22	-0.435387	0.647014	0.089	0.276	6.31E-18
YWHAQ	4.29E-91	-0.435333	0.647049	1	1	1.41E-86
RAB35	4.11E-28	-0.435168	0.647156	0.725	0.919	1.35E-23
LINC00632	3.11E-23	-0.434079	0.647861	0.638	0.831	1.02E-18
RPSAP52	7.03E-18	-0.433754	0.648071	0.185	0.387	2.31E-13
TTL	4.98E-36	-0.433153	0.648461	0.894	0.984	1.64E-31
POLQ	3.31E-36	-0.433131	0.648475	0.189	0.49	1.09E-31
AFP	5.06E-36	-0.432736	0.648732	0.034	0.227	1.67E-31
C1QTNF2	3.07E-21	-0.432594	0.648824	0.109	0.302	1.01E-16
MCOLN1	4.15E-23	-0.432256	0.649043	0.378	0.638	1.37E-18
RCC1	2.49E-29	-0.432129	0.649126	0.675	0.893	8.18E-25
TSPAN2	1.18E-19	-0.431903	0.649272	0.025	0.142	3.89E-15
MCL1	1.19E-39	-0.431748	0.649373	0.991	1	3.92E-35
SIX1	3.21E-22	-0.431735	0.649382	0.092	0.281	1.06E-17
LMF2	2.74E-34	-0.431547	0.649504	0.759	0.921	9.02E-30
SLC25A43	8.40E-39	-0.431519	0.649522	0.53	0.849	2.76E-34
ZGRF1	2.46E-25	-0.430865	0.649947	0.159	0.397	8.09E-21
GPCPD1	1.59E-30	-0.430584	0.650129	0.446	0.742	5.23E-26
MRPL39	4.40E-30	-0.430564	0.650142	0.706	0.907	1.45E-25
SH2B3	6.07E-22	-0.429936	0.650551	0.738	0.884	2.00E-17
AC135050.2	2.30E-48	-0.429724	0.650688	0.588	0.875	7.56E-44
LRP4	8.25E-26	-0.429273	0.650982	0.056	0.234	2.71E-21
UHRF2	1.98E-28	-0.428937	0.651201	0.573	0.838	6.52E-24
PLA2R1	1.09E-15	-0.428574	0.651437	0.167	0.327	3.59E-11
FANCB	3.09E-37	-0.428249	0.651649	0.246	0.568	1.02E-32
ANKRD13A	4.04E-34	-0.428106	0.651742	0.975	0.995	1.33E-29
STK32C	1.83E-34	-0.427997	0.651813	0.233	0.541	6.03E-30
PSMG3	2.73E-28	-0.427578	0.652086	0.719	0.912	8.99E-24
NCAPG2	1.08E-45	-0.42757	0.652092	0.548	0.889	3.54E-41
PGAM1	4.94E-102	-0.427398	0.652204	1	1	1.62E-97
MKI67	1.58E-49	-0.426714	0.65265	0.652	0.923	5.19E-45

ERO1A	2.03E-34	-0.426448	0.652824	0.906	0.986	6.70E-30
NRP1	1.03E-46	-0.426307	0.652916	0.995	1	3.38E-42
GPR180	3.75E-31	-0.426156	0.653015	0.561	0.838	1.23E-26
PRC1	1.56E-45	-0.42555	0.65341	0.751	0.958	5.12E-41
OAF	3.22E-22	-0.425486	0.653452	0.863	0.937	1.06E-17
ECT2	7.56E-41	-0.425458	0.65347	0.734	0.937	2.49E-36
MREG	5.85E-23	-0.42496	0.653796	0.196	0.427	1.93E-18
EXOSC2	6.77E-24	-0.424596	0.654034	0.574	0.807	2.23E-19
FEZ2	2.43E-33	-0.424408	0.654157	0.982	0.998	7.99E-29
EPDR1	1.06E-28	-0.424124	0.654342	0.564	0.835	3.48E-24
MCM6	5.11E-30	-0.423389	0.654824	0.605	0.863	1.68E-25
HABP4	5.03E-26	-0.422837	0.655185	0.561	0.814	1.66E-21
CDH11	1.91E-38	-0.422716	0.655264	0.99	0.998	6.30E-34
CEP85	2.01E-25	-0.422657	0.655303	0.222	0.478	6.63E-21
NAAA	1.48E-29	-0.422558	0.655368	0.665	0.893	4.88E-25
PLAGL1	6.61E-23	-0.421885	0.655809	0.502	0.74	2.17E-18
PIGH	7.38E-32	-0.421711	0.655924	0.299	0.606	2.43E-27
CYTH3	1.88E-29	-0.421251	0.656225	0.598	0.858	6.18E-25
UBE2G2	6.58E-34	-0.42125	0.656226	0.954	0.988	2.17E-29
ESYT3	8.98E-27	-0.420994	0.656394	0.04	0.174	2.96E-22
ELOVL6	1.75E-26	-0.420854	0.656486	0.818	0.942	5.77E-22
SIGMAR1	2.23E-36	-0.420675	0.656604	0.96	0.993	7.35E-32
PARPBP	1.17E-41	-0.420623	0.656637	0.528	0.861	3.84E-37
INIP	1.92E-28	-0.419701	0.657243	0.679	0.896	6.31E-24
FAM126A	2.07E-34	-0.419586	0.657319	0.928	0.988	6.80E-30
LINC01151	1.43E-24	-0.419572	0.657328	0.093	0.258	4.70E-20
C8orf33	1.25E-30	-0.419158	0.6576	0.736	0.933	4.12E-26
NUDT15	1.16E-27	-0.419002	0.657703	0.583	0.838	3.82E-23
KIAA1841	2.75E-27	-0.418917	0.657759	0.478	0.754	9.04E-23
CCDC93	1.49E-24	-0.418893	0.657774	0.481	0.742	4.91E-20
HCG11	4.94E-23	-0.418851	0.657802	0.359	0.622	1.63E-18
DVL1	2.36E-30	-0.418801	0.657835	0.662	0.896	7.76E-26
PLCD1	4.26E-25	-0.418711	0.657894	0.284	0.552	1.40E-20
UBE2Q2	4.06E-32	-0.418554	0.657998	0.843	0.965	1.34E-27
CEP57L1	4.73E-38	-0.418485	0.658043	0.547	0.858	1.56E-33
MRPL17	1.54E-40	-0.418188	0.658238	0.971	0.995	5.08E-36
CLGN	8.82E-20	-0.418127	0.658279	0.072	0.234	2.90E-15
TOPBP1	2.87E-28	-0.417777	0.658509	0.637	0.868	9.46E-24
CD83	8.91E-27	-0.417617	0.658615	0.01	0.128	2.93E-22
S100A3	1.44E-23	-0.417585	0.658636	0.382	0.647	4.75E-19

PKD1P6	2.04E-20	-0.417309	0.658817	0.515	0.742	6.70E-16
H1-5	6.92E-31	-0.417011	0.659014	0.07	0.278	2.28E-26
SLCO3A1	1.25E-23	-0.416716	0.659208	0.187	0.425	4.10E-19
BTG3	1.21E-27	-0.416661	0.659244	0.852	0.961	3.97E-23
TCF7	4.64E-22	-0.41653	0.659331	0.159	0.378	1.53E-17
DLST	1.09E-32	-0.416401	0.659416	0.909	0.984	3.60E-28
STXBP5-AS1	3.41E-18	-0.415937	0.659722	0.242	0.45	1.12E-13
S100A6	6.23E-129	-0.415378	0.660091	1	1	2.05E-124
MRGBP	9.25E-27	-0.415056	0.660303	0.482	0.754	3.04E-22
PGRMC1	3.35E-43	-0.414991	0.660346	0.995	1	1.10E-38
MAP3K6	1.75E-26	-0.414889	0.660414	0.255	0.527	5.77E-22
CYP2R1	3.53E-26	-0.414737	0.660514	0.047	0.218	1.16E-21
DUT	4.69E-43	-0.413288	0.661472	0.985	1	1.54E-38
SERPING1	3.75E-22	-0.413238	0.661505	0.217	0.455	1.23E-17
KNL1	6.50E-52	-0.41316	0.661557	0.471	0.852	2.14E-47
PITHD1	2.12E-30	-0.412874	0.661746	0.737	0.93	6.97E-26
ABL2	2.45E-26	-0.412423	0.662044	0.858	0.958	8.06E-22
BRI3BP	4.26E-19	-0.411415	0.662712	0.467	0.698	1.40E-14
DUS4L-BCAP	9.35E-38	-0.411214	0.662845	0.942	0.993	3.08E-33
PDIA6	3.92E-89	-0.411039	0.662961	1	1	1.29E-84
PAX8-AS1	5.16E-14	-0.410728	0.663168	0.103	0.251	1.70E-09
CBFB	1.75E-28	-0.410702	0.663184	0.872	0.974	5.76E-24
RALB	1.79E-39	-0.410508	0.663313	0.972	0.998	5.87E-35
SRSF7	6.10E-39	-0.41044	0.663358	0.995	0.998	2.01E-34
ICAM3	8.99E-27	-0.410248	0.663486	0.304	0.585	2.96E-22
PLXNA4	4.84E-32	-0.410033	0.663629	0.003	0.107	1.59E-27
DTYMK	2.12E-36	-0.4096	0.663916	0.924	0.986	6.98E-32
TRABD2A	2.70E-23	-0.409238	0.664156	0.334	0.596	8.89E-19
ZFP36L2	8.05E-21	-0.409191	0.664187	0.968	0.984	2.65E-16
CNTN3	3.38E-22	-0.40915	0.664214	0.287	0.538	1.11E-17
UMPS	3.00E-24	-0.409075	0.664264	0.613	0.842	9.88E-20
CAV1	1.05E-72	-0.408768	0.664468	1	1	3.45E-68
ANK2	2.52E-24	-0.408247	0.664815	0.068	0.251	8.297E-20
AL359397.2	9.63E-31	-0.408242	0.664818	0.031	0.2	3.17E-26
NEDD4	1.9E-24	-0.407929	0.665026	0.874	0.949	6.244E-20
KIAA1522	2.28E-24	-0.407877	0.665061	0.217	0.469	7.497E-20
TSPAN5	2.07E-31	-0.407682	0.66519	0.966	0.998	6.813E-27
CLEC11A	9.9E-35	-0.407216	0.6655	0.964	0.995	3.258E-30
CRYBG1	4.28E-16	-0.407151	0.665544	0.365	0.578	1.408E-11
GPAT4	1.94E-28	-0.406783	0.665789	0.72	0.916	6.385E-24

SUV39H2	2.76E-31	-0.406695	0.665847	0.3	0.603	9.071E-27
PSMG1	8.46E-27	-0.405642	0.666549	0.879	0.965	2.785E-22
MAFB	1.06E-13	-0.405615	0.666566	0.047	0.155	3.49E-09
ZDHHC14	1.32E-18	-0.405596	0.66658	0.143	0.334	4.34E-14

pct.1 Percent of cells expressing in the large subpopulation

pct.2 Percent of cells expressing in the small subpopulation

Supplementary Table. 3

Upregulated genes in female-origin than in male-origin from hAM-Muse cells

GeneSymbol	p-value	avg_logFC	FC	pct.1	pct.2	p_val_adj
KCNE4	0.004563	0.2681406	1.307531	0.421	0.37	1

Upregulated genes in male-origin than in female-origin from hAM-Muse cells

GeneSymbol	p-value	avg_logFC	FC	pct.1	pct.2	p_val_adj
MMP11	0.003859	-0.527299	0.590197	0.331	0.374	1
ZFP36	1.75E-31	-0.48119	0.618048	0.761	0.843	5.76E-27
CEMIP	0.000335	-0.446013	0.640176	0.225	0.233	1
JUNB	1.24E-30	-0.390984	0.676391	0.89	0.95	4.07E-26
IGFBP5	0.022173	-0.349645	0.704938	0.468	0.498	1
GPC4	1.17E-07	-0.345658	0.707755	0.096	0.167	0.003862
TXNIP	4.62E-08	-0.335597	0.714911	0.677	0.724	0.0015213
EFHD1	2.10E-05	-0.304723	0.737328	0.21	0.271	0.689697
FOS	7.11E-13	-0.302928	0.738652	0.932	0.947	2.34E-08
WNT2	0.000753	-0.291787	0.746928	0.134	0.182	1
DSP	1.18E-07	-0.274148	0.760219	0.831	0.869	0.0038937
NPR3	0.031577	-0.269227	0.76397	0.265	0.303	1
SOCS3	6.33E-10	-0.262738	0.768943	0.546	0.644	2.08E-05
CNTN4	0.00013	-0.255691	0.774381	0.077	0.115	1

pct.1 Percent of cells expressing in female-origin from hAM-Muse cells

pct.2 Percent of cells expressing in male-origin from hAM-Muse cells
